# Annotated catalogue of the Tachinidae (Insecta, Diptera) of Chile

**DOI:** 10.3897/zookeys.1064.62972

**Published:** 2021-10-21

**Authors:** James E. O’Hara, D. Monty Wood, Christian R. González

**Affiliations:** 1 Canadian National Collection of Insects, Agriculture and Agri-Food Canada, 960 Carling Avenue, Ottawa, Ontario, K1A 0C6, Canada Canadian National Collection of Insects, Agriculture and Agri-Food Canada Ottawa Canada; 2 Instituto de Entomología, Facultad de Ciencias Básicas, Universidad Metropolitana de Ciencias de la Educación, Santiago, Chile Universidad Metropolitana de Ciencias de la Educación Santiago Chile

**Keywords:** Argentina, Chile, Neotropical Region, Oestroidea, parasitoids, Patagonia

## Abstract

The Tachinidae (Diptera) of Chile are catalogued and information is given on distributions, name-bearing types, synonyms, nomenclatural issues, and pertinent literature. The history of tachinid collectors in Chile and authors who have contributed to the systematic knowledge of Chilean tachinids is extensively reviewed. The classification has been updated and 122 genera and 264 species are recognised in Chile. There is a significant amount of endemism with 28 genera and 100 species known only from Chile. There are also 113 species with distributions shared only between Chile and Argentina, particularly in the southern portions of these countries comprising Patagonia.

The catalogue is based on examination of the original descriptions of all nominal species and all other references known to us containing relevant taxonomic and distributional information, for a total of approximately 450 references. Many of the name-bearing types and other Chilean specimens housed in collections were examined. Taxa are arranged hierarchically and alphabetically under the categories of subfamily, tribe, genus, subgenus (where recognised), and species. Nomenclatural information is provided for genus-group and species-group names, including lists of synonyms (mostly restricted to Neotropical taxa) and name-bearing type data. Species distributions are recorded by country within the New World and by larger geographical divisions in the Old World. Additional information is given in the form of notes and references under valid names at the level of tribe, genus, and species. Two genera are newly recorded from Chile: *Chaetoepalpus* Vimmer & Soukup, 1940 (Tachinini) (also newly recorded from Argentina) and *Patelloa* Townsend, 1916 (Goniini). Four species are newly recorded from Chile or other countries: *Lyphaornata* Aldrich, 1934 (Chile); *Chaetoepalpuscoquilleti* Vimmer & Soukup, 1940 (Argentina and Chile); *Phytomypteraevanescens* (Cortés, 1967) (Argentina); and *Xanthobasisunicolor* Aldrich, 1934 (Chile). Eight species previously recorded from Chile are deemed to have been misidentified or misrecorded from Chile (known distributions in parentheses): *Archytasincertus* (Macquart, 1851) (Argentina, Brazil, Paraguay, Uruguay); *Archytasseminiger* (Wiedemann, 1830) (Brazil, Colombia); *Goniacrassicornis* (Fabricius, 1794) (Brazil, Peru, Venezuela, Middle America, West Indies, Nearctic); *Lespesiaandina* (Bigot, 1888) (Cuba); *Lespesiaarchippivora* (Riley, 1871) (widespread Nearctic and most of Neotropical); *Neoethillaignobilis* (van der Wulp, 1890) (Mexico, United States); Siphona (Siphona) geniculata (De Geer, 1776) (Palaearctic, Nearctic [introduced]); and *Winthemiaquadripustulata* (Fabricius, 1794) (Palaearctic, Nearctic, Oriental]. As First Reviser we fix *Paratheresiarufiventris* Townsend, 1929 as the senior homonym and *Sarcoprosenarufiventris* Townsend, 1929 as the junior homonym when the two are placed together in *Billaea* Robineau-Desvoidy, 1830; and we fix *Mayophoriniaangusta* Townsend, 1927 as the senior homonym and *Metarrhinomyiaangusta* Townsend, 1927 as the junior homonym when the two are placed together in *Myiopharus* Brauer & Bergenstamm, 1889. New replacement names are proposed for eight preoccupied names of Neotropical species (country of type locality in parentheses): *Billaearufescens* O’Hara & Wood for *Sarcoprosenarufiventris* Townsend, 1929, preoccupied in the genus *Billaea* Robineau-Desvoidy, 1830 by *Paratheresiarufiventris* Townsend, 1929 (Peru), **nom. nov.**; *Billaeatriquetrus* O’Hara & Wood for *Sarcoprosenatriangulifera* Townsend, 1927, preoccupied in the genus *Billaea* Robineau-Desvoidy, 1830 by *Dexiatriangulifera* Zetterstedt, 1844 (Peru), **nom. nov.**; *Eucelatorianudioculata* O’Hara & Wood for *Eucelatorioideanigripalpis* Thompson, 1968, preoccupied in the genus *Eucelatoria* Townsend, 1909 by *Chetolyganigripalpis* Bigot, 1889 (Trinidad), **nom. nov.**; *Eucelatoriaoblonga* O’Hara & Wood for *Urodexodeselongatum* Cortés & Campos, 1974, preoccupied in the genus *Eucelatoria* Townsend, 1909 by *Exoristaelongata* van der Wulp, 1890 (Chile), **nom. nov.**; *Lespesiathompsoni* O’Hara & Wood for *Sturmiopsoideaobscura* Thompson, 1966, preoccupied in the genus *Lespesia* Robineau-Desvoidy, 1863 by *Eurigasterobscurus* Bigot, 1857 (Cuba), **nom. nov.**; *Myiopharuscharapensis* O’Hara & Wood for *Metarrhinomyiaangusta* Townsend, 1927, preoccupied in the genus *Myiopharus* Brauer & Bergenstamm, 1889 by *Mayophoriniaangusta* Townsend, 1927 (Peru), **nom. nov.**; *Myiopharusincognitus* O’Hara & Wood for *Stenochaetaclaripalpis* Thompson, 1968, preoccupied in the genus *Myiopharus* Brauer & Bergenstamm, 1889 by *Neoxynopsoideaclaripalpis* Thompson, 1968 (Trinidad), **nom. nov.**; and *Myiopharusrufopalpus* O’Hara & Wood for *Paralispepalpalis* Townsend, 1929, preoccupied in the genus *Myiopharus* Brauer & Bergenstamm, 1889 by *Myioxynopspalpalis* Townsend, 1927 (Peru), **nom. nov.** New type species fixations are made under the provisions of Article 70.3.2 of the ICZN*Code* for three genus-group names: *Parafabricia* Brauer & Bergenstamm, 1894 (synonym of *Archytas* Jaennicke, 1867), type species newly fixed as *Parafabriciaperplexa* Townsend, 1931; *Tachinodes* Brauer & Bergenstamm, 1889 (synonym of *Archytas* Jaennicke, 1867), type species newly fixed as *Juriniametallica* Robineau-Desvoidy, 1830; and *Willistonia* Brauer & Bergenstamm, 1889 (synonym of *Belvosia* Robineau-Desvoidy, 1830), type species newly fixed as *Willistoniaaldrichi* Townsend, 1931. Lectotypes are designated for the following four nominal species, all described or possibly described from Chile: *Echinomyiapygmaea* Macquart, 1851 (a valid name in the genus *Peleteria* Robineau-Desvoidy, 1830); *Goniachilensis* Macquart, 1844 (a junior synonym of *Goniapallens* Wiedemann, 1830); *Masiceraauriceps* Macquart, 1844 (a valid name in the genus *Lespesia* Robineau-Desvoidy, 1863); and *Prosopochoetanitidiventris* Macquart, 1851 (a valid name in the genus *Prosopochaeta* Macquart, 1851). The following 27 new or revived combinations are proposed (distributions in parentheses): *Blepharipezaandina* Bigot, 1888 is moved to *Lespesia* Robineau-Desvoidy, 1863 as *L.andina*, *nomen dubium* (Cuba), **comb. nov.**; *Camposodesevanescens* Cortés, 1967 is moved to *Phytomyptera* Rondani, 1845 as *P.evanescens* (Argentina, Chile), **comb. nov.**; *Ectophasiopsisypiranga* Dios & Nihei, 2017 is moved to Trichopoda Berthold, 1827 and assigned to subgenus Galactomyia Townsend, 1908 as T. (G.) ypiranga (Argentina, Brazil), **comb. nov.**; *Embiomyiaaustralis* Aldrich, 1934 is moved to *Steleoneura* Stein, 1924 as *S.australis* (Argentina, Chile), **comb. nov.**; *Eurigastermodestus* Bigot, 1857 is moved to *Lespesia* as *L.modesta* (Cuba), **comb. nov.**; *Eurigasterobscurus* Bigot, 1857 is moved to *Lespesia* as *L.obscura* (Cuba), **comb. nov.**; *Macropatelloatanumeana* Townsend, 1931 is moved to *Patelloa* Townsend, 1916 as *P.tanumeana* (Argentina, Chile), **comb. nov.**; *Masicerainsignis* van der Wulp, 1882 is moved to *Drino* Robineau-Desvoidy, 1863 as *D.insignis* (Argentina, Chile), **comb. nov.**; *Parasetigenahichinsi* Cortés, 1967 is moved to *Chetogena* Rondani, 1856 as *C.hichinsi* (Chile), **comb. nov.**; *Parasetigenaporteri* Brèthes, 1920 and junior synonym *Stomatotachinasplendida* Townsend, 1931 are moved to *Chetogena* as *C.porteri* (Chile), both **comb. nov.**; *Phoroceracalyptrata* Aldrich, 1934 is moved to *Admontia* Brauer & Bergenstamm, 1889 as *A.calyptrata* (Argentina, Chile), **comb. nov.**; *Poliopsauratus* Campos, 1953 is moved to *Admontia* Brauer & Bergenstamm, 1889 as *A.aurata* (Chile), **comb. nov.**; *Poliopsstriatus* Aldrich, 1934 is moved to *Admontia* as *A.striata* (Argentina, Chile), **comb. nov.**; *Ruiziellafrontosa* Cortés, 1951 is moved to *Chaetoepalpus* Vimmer & Soukup, 1940 and placed in synonymy with *C.coquilleti* Vimmer & Soukup, 1940 (Argentina, Chile, Peru), **comb. nov.**; *Ruiziellaluctuosa* Cortés, 1951 is moved to *Chaetoepalpus* as *C.luctuosus* (Argentina, Chile), **comb. nov.**; *Sarcoprosenaluteola* Cortés & Campos, 1974 is moved to *Billaea* Robineau-Desvoidy, 1830 as *B.luteola* (Chile), **comb. nov.**; *Sarcoprosenarufiventris* Townsend, 1929 is moved to *Billaea* where it is a junior secondary homonym and is renamed *B.rufescens* O’Hara & Wood (Peru), **comb. nov.**; *Sarcoprosenatriangulifera* Townsend, 1927 is moved to *Billaea* where it is a junior secondary homonym and is renamed *B.triquetrus* O’Hara & Wood (Peru),**comb. nov.**; *Saundersiaaurea* Giglio-Tos, 1893 is moved to “Unplaced species of Tachinini” (Mexico), **comb. nov.**; *Schistostephanaaurifrons* Townsend, 1919 is moved to *Billaea* as *B.aurifrons* (Peru), **comb. nov.**; *Siphoactiacharapensis* Townsend, 1927 is moved to *Clausicella* Rondani, 1856 as *C.charapensis* (Peru), **comb. nov.**; *Siphoactiaperegrina* Cortés & Campos, 1971 is moved to *Clausicella* as C. *peregrina* (Chile), **comb. nov.**; *Sturmiafestiva* Cortés, 1944 is moved to *Drino* as *D.festiva* (Argentina, Chile), **comb. nov.**; *Sturmiopsoideaobscura* Thompson, 1966 is moved to *Lespesia* Robineau-Desvoidy, 1863, where it is a junior secondary homonym and is renamed *L.thompsoni* O’Hara & Wood (Trinidad), **comb. nov.**; *Trichopodaarcuata* Bigot, 1876 is returned to *Trichopoda* from Ectophasiopsis Townsend, 1915 and assigned to subgenus Galactomyia (Argentina, Chile), **comb. revived**; and *Trichopodagradata* Wiedemann, 1830 is returned to *Trichopoda* from *Ectophasiopsis* and assigned to subgenus Galactomyia (Argentina, Brazil, Uruguay), **comb. revived.** New or revived generic and specific synonymies are proposed for the following 14 names: *Camposodes* Cortés, 1967 with *Phytomyptera* Rondani, 1845, **syn. nov.**; *Ectophasiopsis* Townsend, 1915 with Trichopoda Berthold, 1827, subgenus Galactomyia Townsend, 1908, **syn. nov.**; *Embiomyia* Aldrich, 1934 with *Steleoneura* Stein, 1924, **syn. nov.**; *Fabriciaandicola* Bigot, 1888 with *Peleteriarobusta* (Wiedemann, 1830), **syn. revived**; *Macropatelloa* Townsend, 1931 with *Patelloa* Townsend, 1916, **syn. nov.**; *Peleteriainca* Curran, 1925 with *Peleteriarobusta* (Wiedemann, 1830), **syn. revived**; *Poliops* Aldrich, 1934 with *Admontia* Brauer & Bergenstamm, 1889, **syn. nov.**; *Ruiziella* Cortés, 1951 with *Chaetoepalpus* Vimmer & Soukup, 1940, **syn. nov.**; *Ruiziellafrontosa* Cortés, 1951 with *Chaetoepalpuscoquilleti* Vimmer & Soukup, 1940, **syn. nov.**; *Sarcoprosena* Townsend, 1927 with *Billaea* Robineau-Desvoidy, 1830, **syn. nov.**; *Schistostephana* Townsend, 1919 with *Billaea*, **syn. nov.**; *Siphoactia* Townsend, 1927 with *Clausicella* Rondani, 1856, **syn. nov.**; *Stomatotachina* Townsend, 1931 with *Chetogena* Rondani, 1856, **syn. nov.**; and *Sturmiopsoidea* Thompson, 1966 with *Lespesia* Robineau-Desvoidy, 1863, **syn. nov.**

“The joke goes in Chile, that God the Creator, after seven days of hard labor, setting of the world out of chaos, was indeed tired and weary, when Angels approached Him with concern to tell that there still were huge assortment of deserts, oceans, mountains, lakes, forests, volcanoes, rocks, glaciers, islands and the rest, that they didn’t know where to place. The sensible answer from the Almighty was: well, throw it away in any remote corner still available! And this, gentlemen, was the way in which Chile was supposedly built.”

– Campos (1975: 7)

## Introduction

Chile is a long slender country nestled between the Pacific Ocean and the Andes and stretching for more than 4000 kilometres from Peru to the southern tip of South America. The Atacama Desert in the north gradually transitions to the fertile Central Valley that runs through the middle of the country for over 600 kilometres. This is the agricultural heartland of Chile and is noted for its Mediterranean climate and large variety of produce that is exported to countries around the world. Farther to the south are the ecoregions of the Valdivian temperate rainforest and Magellanic subpolar rainforest. The former has an especially diverse fauna and flora with a high percentage of endemics whereas the harsher and less hospitable conditions of the latter have limited its biodiversity. The Andes Mountains stretch along the eastern edge of Chile and faunistically separate it from the rest of South America except in the more southern reaches of the continent (i.e., Patagonia). The Tachinidae fauna of Chile has not been catalogued for fifty years, since Guimarães (1971) published on the family in *A catalogue of the Diptera of the Americas south of the United States*. That work was of necessity built upon the taxonomic contributions of predecessors and was heavily influenced by the most prolific describer of New World Tachinidae, C.H.T. Townsend. The culmination of Townsend’s life’s work was a twelve-volume series entitled *Manual of Myiology* (Townsend 1934–1942) that laid out an idiosyncratic classification for tachinids and related groups and provided keys to, and descriptions of, all of the world’s genera. The *Manual of Myiology* and the many other publications of Townsend set forth a taxonomic scheme in which many genera were monotypic and the arrangement of genera into tribes and families followed ideas about relationships that have since been largely abandoned. An alternative to such restricted genera was proposed for a portion of the South American fauna in Aldrich’s (1934) treatment of Tachinidae in *Diptera of Patagonia and South Chile*, but that work did not arrange genera into higher categories. Younger taxonomists who came along near the end of Townsend’s era, like R. Cortés in Chile and E. Blanchard in Argentina, were obliged to interpret their faunas within the context of *Manual of Myiology*. As a result, as noted by Wood and Zumbado (2010: 1355): “Subsequent authors faced with this multiplicity of generic names have had little choice, when their specimens did not fit the existing narrow definitions, but to describe yet more genera”.

Our main objective here has been to catalogue the described taxa of Chile rather than revise the classification, but we have done as much of the latter as seemed appropriate given our level of understanding of the fauna. We have updated the higher categories of tribes and subfamilies (following Sabrosky 1999 for priority of family-group names), proposed new synonymies and combinations, and replaced some preoccupied names with new ones. Our placements of taxa have been aided by recent advances in our understanding of tachinid phylogeny and especially by the morphological study of Cerretti et al. (2014) and molecular study of Stireman et al. (2019). Our own DNA barcoding of Chilean tachinid specimens in the Canadian National Collection of Insects has also helped with the placement of certain taxa. Certain groups are too problematic to be easily reclassified here (e.g., the Polideini) and we have maintained the current classification of these until more thorough revisions can be undertaken.

The tachinids of Chile, as catalogued below, consist of 122 genera and 264 species, with 28 genera and 100 species which are endemic to Chile according to presently known distributions. Another 113 species are uniquely shared between Chile and Argentina, particularly in the southern portions of these countries. There is still a significant portion of the tachinid fauna of Chile that is undescribed and it is our hope that this catalogue will benefit those who pursue systematic studies of the fauna in the future.

## A historical perspective on the Tachinidae of Chile

The earliest accounts of entomological pursuits in Chile were traced back to the 1500s by Cortés and Herrera (1989) in their review of the history of entomology in Chile. It was not until the 1800s that any organised progress was made. As the fledgling country struggled towards independence from Spanish rule, an ambitious plan was developed for a comprehensive account of virtually all aspects of Chilean natural and political history, including entomology. The person chosen to lead this grand endeavour was a young French naturalist who had emigrated to Chile just a year or so earlier and was not yet fluent in Spanish. His name was Claude [or Claudio] Gay (1800–1873) and he was commissioned in 1830 to research and write what would later be entitled *Historia física y política de Chile según documentos adquiridos en esta república durante doce años de residencia en ella y publicada bajo los auspicios del supremo gobierno* [“Physical and political history of Chile according to documents acquired in this republic during twelve years of residence in it and published under the auspices of the supreme government”].

Preparing the *Historia* consumed much of the next 40+ years of Gay’s life. He travelled extensively throughout Chile gathering information and making collections of the fauna and flora before returning to France in 1842. There he commenced the writing of the *Historia* subject by subject in a long series of volumes. When finally completed the *Historia* filled ca. 30 volumes and was published over the course of 28 years, from 1844 to 1871. The cumulative effect of this extraordinary work was to define the physical characteristics of Chile and thus provide a foundation for decision-making for years to come. Its coverage of life forms was thought to be so complete that Cortés and Herrera (1989: 303) remarked: “Hasta bien entrado el siglo XX se acostumbraba a decir en Chile ‘si no está en Gay (por plantas, insectos y animales) es nuevo’” [“Until well into the 20^th^ Century it was customary to say in Chile ‘if it is not in Gay (for plants, insects and animals) it is new’.”].

Eight volumes of the *Historia* were devoted to zoology and a section in the seventh dealt with the Diptera. Gay had enlisted the aid of French zoologist C. Émile Blanchard (1819–1900) to prepare this section. No keys were included but species were arranged in a hierarchical classification of genera, tribes and families and each category was accompanied by a diagnosis and description (Blanchard 1854). Tachinids were grouped with the “Muscianos” and arranged into three tribes, but at this early stage in Chilean entomology there were few tachinid species known from the country. Blanchard recorded only seven, consisting of six described by French dipterist Pierre-Justin-Marie Macquart (1778–1855) and one described by German dipterist Christian Rudolph Wilhelm Wiedemann (1770–1840). The last species, treated as *Scotipteramelaleuca* (Wiedemann) and now considered a synonym of *Scotipteravenatoria* (Fabricius), was described from Brazil and was questionably recorded from Chile; it is no longer recognised as a Chilean species.

By the time of Blanchard (1854), specimens of Chilean Tachinidae were trickling back to Europe and making their way into institutional and private collections. The first of these to be described were collected by Captain Philip Parker King during the voyage of the English naval vessels HMS *Adventure* and HMS *Beagle* to the “Straits of Magellan” at the southern tip of South America in 1825–1830. The specimens were from “Cape Gregory” [Cabo San Gregorio] and “Port Famine” [Puerto del Hambre] in the Magallanes province of Chile. King gave the specimens to the British Museum (Natural History) in London (now the Natural History Museum, NHMUK) where they were later examined and described by English entomologist Francis Walker (1809–1874) in a paper devoted to King’s insects (Walker 1836). Walker was a general entomologist who described insects of all kinds with no special talent for taxonomy or descriptions. He described five tachinid species from King’s material and placed them all in the genus *Tachina*. Subsequent study of the specimens led to their reassignment to two subfamilies and three tribes, with one of Walker’s species having been described twice under different names (see catalogue below for further details).

Walker (1849) later described another Chilean species, this one simply from “Chili” and collected by Hugh Cuming (1791–1865), an Englishman who settled in Chile and became a prosperous businessman and amateur naturalist. Walker also assigned this species to *Tachina* but it was later synonymised with an earlier Macquart name and is now placed in the common genus *Archytas*.

None of the species described by Walker was included by Blanchard in the *Historia*. We have been unable to determine the reason for this but it was likely an intentional act given the care with which the insect chapters were prepared and the presumed availability of the Walker publications at the time.

Species described from Chile by Macquart (1844, 1851) and listed in the *Historia* by Blanchard (1854) were based on specimens in the Muséum National d’Histoire Naturelle (the “Paris Museum”, MNHN) received from French collectors residing in or visiting Chile during the first half of the 19^th^ Century. These collectors were frequently prominent and adventurous men better known for their non-entomological achievements. Macquart gave credit to them by naming the collector at the end of each species description. They were among the first to collect Tachinidae in Chile and are listed here in recognition of the important role they played in making these insects available to others who could describe them (collectors arranged by year of birth):

1. “Du Brésil ou du Chili. M. Gaudichand.” Charles Gaudichand-Beaupré (1789–1854), naturalist. Two species, as Masicera auriceps [= Lespesia auriceps] and Gonia virescens; neither included in the Historia and both to this day of uncertain provenance.
2. “Du Chili; de la Conception. M. Dumont-Durville.” Jules Dumont d’Urville (1790–1842), second in command on the French frigate La Coquille, which reached Concepción in January 1823. Macquart named Trichoprosopus durvillei in his honour.
3. “Du Chili. M. Gay.” Claude [or Claudio] Gay (1800–1873), author of Historia física y política de Chile… (see above for further details about Gay). Four species, as Jurinia scutellata [= Archytas scutellatus], Gonia chilensis [= Gonia pallens Wiedemann], Prosopochaeta nitidiventris and Hyalomyia chilensis [= Phasia chilensis].
4. “De la Patagonie. M. d’Orbigny.” Alcide Charles Victor Dessalines d’Orbigny (1802–1857), naturalist. One species, Gonia lineata; not included in the Historia presumably because of the uncertain provenance; current distribution as Argentina, Chile and Peru. This collector should not be confused with younger brother Charles Henry Dessalines d’Orbigny (1806–1876), author of Dictionnaire Universel d’Histoire Naturelle.
5. “Du Chili. M. Pissis.” Pierre Joseph Aimé Pissis (1812–1889), geographer. One species, as Echinomyia pygmaea [= Peleteria pygmaea]. Monte Pissis in Argentina, one of the highest mountains in South America, was named in his honour.

This practice of collecting insects in Chile and sending them back to Europe to be preserved in collections and described by specialists continued throughout the second half of the 19^th^ Century. Tachinids seem not to have been the most popular of insects to send back to Europe during this time judging from the few that fell into the hands of the leading dipterists. The dipterists involved in describing them are reviewed below.

French dipterist Jacques-Marie-Frangille Bigot (1818–1893) described his first Chilean tachinid in 1857. The description was based on material received from Philibert Germain (1827–1913), a French entomologist who had emigrated to Chile in 1850. Germain was a man of considerable talents; over the course of the next 50 years he held important positions in Chilean entomology and collected throughout the country (see review of Germain in Cortés and Herrera 1989). Bigot described an additional 12 Chilean tachinids in six papers between 1876 and 1888. These species were all from “Chili” and caught by unnamed collectors except for two described in Bigot’s (1888a) section on Diptera in the multi-volume report *Mission scientifique du Cap Horn, 1882–1883*. These tachinids and other natural history specimens were collected by naval physician Paul Daniel Jules Hyades (1847–1919) in the vicinity of “Orange Bay” [Isla Hoste, Bahía Orange] during the voyage to Cape Horn of the French frigate *La Romanche*. Bigot (1888a: 26) dedicated the genus *Hyadesimyia* to Hyades.

French dipterist André-Jean-Baptiste Robineau-Desvoidy (1799–1857) achieved notoriety in part because of his prodigious output: nearly 600 generic names and over 3000 specific names (Evenhuis et al. 2010). These names were mostly of European schizophoran Diptera and a sizable portion of them later became junior synonyms. He named just one Chilean tachinid, *Juriniaandana* [= *Archytasscutellatus* (Macquart)], from “Chili” (Robineau-Desvoidy 1863a). The collector was not given and the type(s) is lost (Cortés 1963).

The eminent Italian dipterist Camillo Rondani (1808–1879) described five species from Chile based on material collected by Rudolph [or Rodulfo] Amandus Philippi (1808–1904). Philippi was born in Germany and received his higher education in Berlin, where coincidentally he was taught “Physische Geographie” by the famed naturalist and South American explorer Alexander von Humboldt (1769–1859) (Kabat and Coan 2017). Philippi emigrated to Chile in 1851 after revolutions in Germany forced him to leave for his personal safety. He settled first in Valdivia in southern Chile but moved to Santiago in 1853 when the President of Chile appointed him director of the Museo de Historia Natural at the Universidad de Chile. He continued as director when the museum was relocated to another part of Santiago and renamed Museo Nacional de Historia Natural. He relinquished the position in 1897 at the age of 88. Philippi was well respected within the scientific community during his tenure as director and became one of the most influential voices for the natural sciences in Chile. He was also a prolific author of taxonomic papers on a wide variety of organisms throughout the animal kingdom (Cortés and Herrera 1989; Kabat and Coan 2017). His *Aufzählung der chilenischen Dipteren*, published in 1865, stands out as his most impressive contribution to dipterology with descriptions of 424 species of Diptera, but no Tachinidae (Camousseight 2005).

Three of the five species described by Rondani (1863) based on Philippi material are still recognised as valid. One of them was named *Spathipalpusphilippii* in honour of Philippi and was based on material from Valdivia. Philippi had a farm there and much of the material described in his *Aufzählung der chilenischen Dipteren* was collected from that area.

German amateur entomologist Johann Friedrich Jaennicke (1831–1907) published a significant paper entitled *Neue exotische Dipteren* in 1867 that included 18 new species of Tachinidae from Indonesia, Ethiopia, Cuba, Mexico, Panama, Venezuela and Chile. The single Chilean species was described as *Demoticusratzeburgii* [= *Deopalpuspruinosus* (Rondani)] and was presumably named in honour of Julius Theodor Christian Ratzeburg (1801–1871), a prominent German professor famous for his pioneering work in forest entomology. The type(s) of *D.ratzeburgii* was from “Chile”; the collector “Bayrhoffer” is unknown to us. Jaennicke (1867) was also responsible for describing the common New World genus *Archytas*.

The circumnavigation of the world by the Austrian frigate SMS *Novara* in 1857–1859 was the largest naval expedition ever undertaken by Austria. It was primarily a voyage of scientific discovery and its bountiful harvest of natural history specimens would greatly increase the holdings and global status of the natural history collection in Vienna. The principal zoologist responsible for collecting insects during the *Novara* voyage was Georg Ritter von Frauenfeld (1807–1873), a curator at the natural history museum in Vienna. The task of describing the expedition’s Diptera went to Ignatz Rudolph Schiner (1813–1873 [first initials often as J.R. on his publications]), a talented Viennese dipterist well-known for authoring the Diptera section of *Fauna Austriaca* (published in parts between 1860 and 1864). Schiner (1868) described 37 species of Tachinidae from the *Novara* expedition but only three were specifically from Chile; another 15 were simply from “Süd-Amerika”. Twenty years after these *Novara* tachinids were described the vast natural history collection in Vienna was moved into the new Naturhistorisches Museum Wien (or “Vienna Museum”, NHMW) along the *Ringstrasse*, where it could be displayed in regal splendour and the scientific staff could more easily study its specimens.

The *Novara* was preceded in its circumnavigation of the world by the Swedish frigate HSwMS *Eugenie* during the years 1851 to 1853. A huge number of insects was collected during the expedition and various Swedish experts published on the newly discovered species. The Diptera were described by Carl Gustaf Thomson (1824–1899) and comprised over 300 new species (Thomson 1869). Only one was a tachinid, *Degeeriaantarctica* [= *Admontiaantarctica*]. The locality was given as “Patagonia” but was probably “Port Famine” [Puerto del Hambre] according to the review of the *Eugenie* localities by Persson (1971: 168). The collector was likely the ship’s physician and zoologist, Johan Gustaf Hjalmar Kinberg (1820–1908). Although the voyage of the *Eugenie* preceded that of the *Novara*, the Diptera species described by Schiner (1868) have priority over those of Thomson (1869).

Dutch dipterist Frederik Maurits van der Wulp (1818–1900) published extensively on world Diptera, with an emphasis on Tachinidae. He described close to 500 tachinid species, including over 400 from Mexico and mostly published in the remarkable *Biologia Centrali-Americana* (e.g., van der Wulp 1890a–e). Three tachinids were described from Chile in van der Wulp (1882) and were based on specimens in the “Leyden Museum” (Leiden, RMNH) received from or collected by entomologist and beetle specialist Carl August Dohrn (1806–1892). Dohrn held the position of president of the Entomological Society of Stettin (Entomologischer Verein zu Stettin) for over 40 years. [Stettin, now Szczecin, has been part of Poland since the end of World War II.]

The first catalogue of Chilean Diptera after Blanchard’s (1854) treatment in Gay’s *Historia* was published in 1888 by Edwyn Charles Reed (1841–1910). Reed, an English naturalist, emigrated to Chile in 1869 and held various positions in entomology and natural history within the country, including director of the Museo de Historia Natural de Concepción at the time of his death (Cortés and Herrera 1989; Etcheverry 1993; Edmundson 2009). The catalogue was a minimalistic listing of 716 species of Diptera. Twelve species of Tachinidae are listed in the main body of the text and they comprise the species in the *Historia*, the three Schiner species mentioned above, and two species originally described from Brazil by Wiedemann (details above). The Chilean species of Bigot were originally overlooked but were added at the end of the list prior to *Anales de la Universidad de Chile* going to press. The Chilean species described by Rondani (1863), Robineau-Desvoidy (1863a) and Jaennicke (1867), along with the species described from the southern tip of Chile not in the *Historia* (to which can be added the single species described by Thomson in 1869), were not included in Reed’s (1888) catalogue. Aldrich (1928b) named the tachinid genus *Reedia* after Reed and then renamed it *Edwynia* (Aldrich 1930) when *Reedia* Aldrich was discovered to be a junior homonym of *Reedia* Ashmead, 1904.

Austrian zoologist Friedrich Moritz Brauer (1832–1904) was employed at the Naturhistorisches Museum Wien (NHMW) as a curator for many years (of Mollusca and then Insecta) and then as director. He engaged the assistance of Vienna dipterist Julius Edler von Bergenstamm (1837–1896) to prepare a monumental work entitled *Vorarbeiten zu einer Monographie der Muscaria schizometopa* (*exclusive Anthomyidae*), published in four parts between 1889 and 1894 (see O’Hara (2013a) for more about these authors, their monographs, and the NHMW). Brauer and Bergenstamm (1891) and Brauer (1898) each described one tachinid species from Chile. The first was collected by Philippi (in 1870 according to a label quoted by Aldrich (1925: 459)) and the other came from the Bigot collection (collector unknown). Both names are now junior synonyms of other names.

By the end of the 19^th^ Century there were ca. 40 species of Tachinidae described from Chile and a few more described from elsewhere and later recorded from Chile. The specimens upon which they were based had been collected over many years in different parts of the country by a variety of collectors, but all had something in common: they were described by European dipterists who had never been to Chile. The collectors were usually adventurous naturalists and the describers were prominent figures in European scientific institutions. Both of these groups were essential to the early knowledge that was being generated on Chilean Tachinidae.

One of the sons of Edwyn Charles Reed (see above), Carlos Samuel Reed (1888–1949), pursued a career in zoology and was most active in entomology and ornithology (Etcheverry 1993). He became an unintentional author of the tachinid name *Tachinaporteri* when he published on the biology of this undescribed species (Reed 1907). He wrote that his father had set aside specimens of it in the Museo de Historia Natural de Concepción under the “nombre MS. de [manuscript name of] *TachinaPorteri*, Reed”. By publishing this name and providing a partial description of the species, Reed made the name nomenclaturally available. The senior Reed had chosen the name to honour Carlos Emilio Porter (1867–1942), a director of the Museo de Historia Natural de Valparaíso. *Tachinaporteri* Reed, 1907 was the first tachinid described from Chile by a Chilean-born author.

A more formal but brief description of *Tachinaporteri* Reed was given by Brèthes (1910) with name and authorship as “*Exoristaporteri* (Reed) Brèthes”. Specimens of this species have not been located in recent times and Cortés and Hichins (1969: 90) treated it as *incertae sedis*. Jean [or Juan] Brèthes (1871–1928) was a French-born entomologist and professor in Argentina. From 1902 until his death, Brèthes was the curator in charge of the entomological collection in the Museo Argentino de Ciencias Naturales “Bernardino Rivadavia” in Buenos Aires (MACN). He described more than 1100 species of insects, mostly from Argentina, Chile, and other South American countries (Dallas 1928; Mulieri et al. 2013; Rossi Belgrano and Rossi Belgrano 2018). Approximately 20 species belonged to the Tachinidae including five from Chile. Brèthes’ name-bearing types in MACN were discussed in Mulieri et al. (2013).

The American-born dipterist Charles Henry Tyler Townsend (1863–1944) began his taxonomic studies of Tachinidae in North America in the 1890s with short papers of a regional nature and concluded his career decades later with his huge *Manual of Myiology in Twelve Parts*, 1934–1942. During the course of his career he described ca. 1500 genera and nearly 1600 species (Arnaud 1958; Evenhuis et al. 2015), most belonging to the Tachinidae. In his *Manual of Myiology* he gave an overview of world Tachinidae and other related flies with keys to, and diagnoses of, all tribes and genera. Townsend was a well-disciplined taxonomist with an intimate knowledge of the literature and a remarkable ability to recognise species, but his legacy has been tarnished by his propensity for monotypic genera and adoption of a higher classification that now appears to be overly artificial (O’Hara 2013b). He has left behind so many genera, especially in South America, that modern systematists are still struggling to determine how best to reduce and reorganise them into more natural and manageable tribes.

Townsend was an avid and adventurous collector who caught many of the New World specimens that he described as new species and genera. He spent much of the latter half of his life in South America, collecting and describing tachinids and ultimately preparing his *Manual of Myiology*. He lived off and on in Peru before permanently settling in Itaquaquecetuba in the São Paulo province of Brazil in 1929 on a property he had purchased about ten years earlier (Hansen and Toma 2004; Evenhuis et al. 2015). Only once did Townsend visit Chile, in 1927 on a journey that took him all the way from Punta Arenas in the south to the bordering country of Peru in the north (Cortés 1944a) where he was currently employed. That trip resulted in the description of four tachinid species and one new genus from Chile (Townsend 1928b). Townsend described a total of 16 species from Chile between 1915 and 1931, ten of which are currently valid. Besides by himself, the type specimens of his new species were collected by Anastasio Pirión (1888–1959; six species including patronyms *Pirionimyiaparadoxa* and *Dolichocypterapirioni*), E.C. Reed (see above; two species), Paul [or Pablo] Herbst (1861–1927; two species) and persons unknown (two species). The three known collectors were discussed or mentioned by Cortés and Herrera (1989). Twenty-nine species described by Townsend are currently recognised as valid in Chile and they comprise the aforementioned ten species described from Chile, 15 from Peru, two from Argentina, and one each from Bolivia and Brazil. Twenty-seven Townsend genera are recognised in Chile and 123 of his generic names are listed as junior generic synonyms.

There is no account of Townsend’s journey through Chile in 1927 but we know from the dates of collection of his Chilean specimens that he was in Punta Arenas on February 5^th^ and Valparaíso on February 15^th^. He likely travelled southward through Argentinian Patagonia and northward through Chilean Patagonia. Either known or unknown to him, he had just missed a major Diptera expedition to Patagonia by barely a month. The results of that expedition would lead to a profound advance in tachinid taxonomy in both Chile and Argentina.

The Patagonian expedition was conceived by English dipterist, Frederick Wallace Edwards (1888–1940) and had the backing of his employer, the British Museum (Natural History) (NHMUK). His partner in the expedition was American dipterist Raymond Corbett Shannon (1894–1945), who had worked as an assistant at the Bureau of Entomology, United States Department of Agriculture, in Washington but was currently working in Argentina for the Argentine Government on insects of public health concern (McAtee and Wade 1951). Their plan was to collect a broad range of Diptera in the southern temperate forest of Chile and Argentina at a latitude of ca. 41°S, corresponding to northern Patagonia. The small party of Edwards, Shannon and their wives started their collecting expedition near the mouth of the Río Negro in Argentina in late October, 1926 and finished at Concepción in Chile on 27 December (Edwards 1929). A total of 40,000 insects was collected including 30,000 Diptera.

The Edwards-Shannon expedition was part of a grander plan. The Diptera were sent to specialists to serve as a foundation for family treatments in a monographic series called *Diptera of Patagonia and South Chile*. Authors were expected to incorporate previously known species from Patagonia into their treatments and describe the new species they discovered. When they were done, the Edwards material went back to NHMUK and the Shannon material was later donated to the Smithsonian Institution (USNM).

John Merton Aldrich (1866–1934), a well-respected American dipterist at USNM, assumed responsibility for working up the Tachinidae for *Diptera of Patagonia and South Chile*. He was renowned for his catalogue of North American Diptera (Aldrich 1905) and his revisionary work on a number of Diptera families including Tachinidae. His monograph on Patagonian Tachinidae (Aldrich 1934) was an impressive achievement and the first comprehensive revision of a regional tachinid fauna in Chile. It included keys to genera and species, descriptions or diagnoses of genera and species, synonymy, illustrations and notes, but no arrangement into tribes and subfamilies. The number of genera and species was summarised in the introduction: “There are found to be 138 species and 2 varieties, of which 90 species and the 2 varieties are described as new; the total number of genera is 70, of which 28 are new” (Aldrich 1934: 1). Of the 90 new species that Aldrich described from Chile and/or Argentina in that work, 78 are recognised as valid in our catalogue below; another eleven species from earlier Aldrich papers (particularly Aldrich 1928b) are also recognised from Chile. Among these are species named in honour of Edwards (*Callotroxisedwardsi* and *Lyphaedwardsi*), Shannon (*Metopomuscopteryx* [= *Alexogloblinia*] *shannoni*), Pirión (discussed above; *Pirionafasciculata* and *Myiopharuspirioni*) and English naturalist Charles Darwin (1809–1882) (*Pelycopsdarwini*). The holotype of the last was collected by Darwin from “Port Famine” [Puerto del Hambre] during the second voyage of HMS *Beagle*, likely in February 1834—almost exactly 100 years before Aldrich’s monograph was published on 24 March 1934. Sadly, Aldrich passed away unexpectedly two months later on 27 May 1934. The new genera of Aldrich (1934) were included in Townsend’s *Manual of Myiology*. Guimarães (1971) named the genus *Aldrichiopa* after Aldrich and Cortés (1944e) named a species after him, as *Cylindromyiaaldrichi*.

Canadian-born Charles Howard Curran (1894–1972) was hired in 1922 as the first dipterist at the Canadian National Collection of Insects (CNC) in Ottawa but moved to New York for a position at the American Museum of Natural History (AMNH) in 1928. He rose to prominence as a general dipterist with many taxonomic revisions to his credit but is best remembered for his masterful coverage of flies in *The Families and Genera of North American Diptera* (Curran 1934). Only a few of the tachinid genera and species described by Curran are relevant to the Chilean fauna. Three of his generic names (proposed for species in Jamaica and Panama) and three of his species names (two named for Chilean specimens and one for Peruvian specimens) are junior synonyms. The only valid species is *Archytasperuanus*, described from Peru and since recorded from Chile.

A contemporary of Curran was American entomologist Henry Jonathan Reinhard (1892–1976). Reinhard spent most of his career in College Station, first as a general entomologist at the Texas Agricultural Experiment Station and then as a professor at Texas A&M University (Burke 1977). In addition to his professional responsibilities he had an unwavering passion for tachinids and sarcophagids and published frequently on them throughout his adult life. He was instrumental in describing the rich but little-known fauna of his part of Texas but also wrote generic revisions and described species from more distant parts of the New World. Reinhard, like Curran, was not significantly involved with Chilean tachinids. Several of his generic revisions are helpful from a Chilean perspective (e.g., *Pseudochaeta*, *Winthemia*, *Leucostoma*) and one genus that he described from the United States, *Clastoneuriopsis*, has a species in Chile. Two of Reinhard’s generic names (proposed for species in United States and Chile) and two of his species names (both based on Chilean specimens) are junior synonyms. One species described from Argentina, *Winthemiasingularis*, is valid and has been recorded from Chile. Reinhard’s personal collection was purchased in 1968 by the CNC and his name-bearing types located there are listed in Cooper and O’Hara (1996).

Everardo Eels Blanchard (1899–1971) was born in Buenos Aires and received training in entomology at the University of Maine in United States (Pirán 1972). After returning to Argentina he embarked on a long and illustrious career that encompassed nearly all aspects of entomology. He worked for the Ministerio de Agricultura and in time became director of the Instituto de Sanidad Vegetal. Among other pursuits and responsibilities, Blanchard found time to describe Argentine insects across a broad range of families and orders, with a general focus on those of agricultural importance. The Tachinidae ranked high among the insects he studied and his new taxa comprised ca. 50 genera and 125 species, all described from Argentina. Nearly all of the species names continue to be recognised as valid and six species have been recorded from Chile. Blanchard, like Townsend, had a restricted view of tachinid genera and only half of the ones he described are still considered valid. Three of his genera are recognised in Chile and another 18 generic names are listed in the catalogue below as junior synonyms. Cortés (1973b) wrote an obituary for Blanchard and later (Cortés 1979) named a Chilean species after him (*Ateloglutusblanchardi*). Blanchard has received two other patronyms in Tachinidae, one by Guimarães (1971: 44, replacement name *Peleteriablanchardi*) and another by Toma and Guimarães (2002: 45, Ecuadorian species *Leschenaultiablanchardi*). Blanchard’s name-bearing types in MACN were discussed in Mulieri et al. (2013).

We come next to the central figure in Chilean tachinidology, Raúl Eduardo Cortés (1915–2001). Cortés was born in the coastal city of Coquimbo in northern Chile. He was educated at the Universidad de Chile in Santiago and spent a couple of years at Harvard University in the United States. His study of tachinid taxonomy began early while he was an entomologist in the Sección Zoología Agrícola of the Departamento de Sanidad Vegetal in the Ministerio de Agricultura, and professor at the Universidad Católica de Chile (both in Santiago). His first taxonomic papers on Tachinidae appeared in 1944, coincidentally the same year as the death of the patriarch of tachinidology at the time, C.H.T. Townsend. Cortés (1944a) published a short biography of Townsend outlining his major achievements. That same year, Cortés (1944b) published a concise review of the history of tachinid studies in Chile that provides a good companion to our treatment of this subject here.

A milestone for dipterology in Chile was reached with the 1946 publication of *Catálogo de los dípteros de Chile* under the leadership of Carlos Stuardo [Ortíz] (1895–1962). Just a year before, Cortés (1945e) had dedicated one of his first tachinid genera, *Stuardomyia*, to his senior colleague. The tachinid section of the catalogue was prepared by the young Cortés (1946). Chilean Tachinidae were still relatively little known in the 1940s. Early European authors had described a spattering of species that they had generally assigned to new genera or Old World genera that were familiar to them. Aldrich brought some order to these early names but his emphasis was on the fauna of southern (Patagonian) Chile and he provided no higher classification beyond genus. Townsend then rearranged the entire Tachinidae of the world in his remarkable but idiosyncratic *Manual of Myiology*. Cortés (1946) had to make a choice of whom to follow and he sided with Aldrich:

“*Al preparar esta lista de los Tachinidae de Chile, el autor ha querido conservar esencialmente el criterio sistemático con que el Dr. J. M. Aldrich tratara las especies patagonianas en Diptera (7, 1:1–170, 1934). Se han introducido, sin embargo, las modificaciones que obvia y naturalmente había que hacer, especialmente en aquellos géneros y grupos que el autor ha podido estudiar con más abundante y representativo material.*

*Al adoptar este criterio*—*a pesar de que según Townsend prácticamente ninguna de nuestras especies fué correctamente ubicada por el Dr. Aldrich*—*el autor ha preferido continuar la línea sistemática por la cual el estudio de nuestros Tachinidae hasta ahora se ha guiado. Un cambio del criterio conservador de Aldrich al concepto extremadamente radical de Townsend, seguramente traería más confusión que beneficios para el estudio futuro de esta familia.*”

[In preparing this list of the Tachinidae of Chile, the author wanted to essentially preserve the systematic criterion with which Dr. J. M. Aldrich treated the Patagonian species in *Diptera of Patagonia and South Chile* (7, 1: 1–170, 1934). However, the modifications that obviously and naturally had to be made have been introduced, especially in those genera and groups that the author has been able to study with more abundant and representative material.

By adopting this criterion—although according to Townsend practically none of our species was correctly placed by Dr. Aldrich—the author has preferred to continue the systematic line by which the study of our Tachinidae has so far been guided. A shift from the conservative approach of Aldrich to the extremely radical concept of Townsend would surely bring more confusion than benefits for the future study of this family.] (Cortés 1946: 172.)

Cortés (1946) arranged the known Tachinidae of Chile into 72 genera and 121 species (plus an additional 13 names listed as “Species *incertae sedis*”) and, following the style of the catalogue as a whole, provided no higher classification. The compilation of this catalogue and the earlier review of the history of Chilean Tachinidae studies provided Cortés with a solid understanding of the state of tachinid taxonomy in Chile at this early point in his life-long dedication to the family. Cortés published ca. 50 papers on mostly Chilean tachinids over a span of 50+ years (González 2001) and during that time described 30 genera (24 currently valid) and 73 species (70 currently valid) of Tachinidae.

Most papers published by Cortés were straightforward taxonomic treatments with descriptions of species and/or genera, keys if appropriate, and notes about types and synonymy. Tachinid taxonomy in Chile advanced incrementally in this fashion for a number of years until the fauna as a whole was reviewed in a comprehensive monograph by Cortés and Hichins (1969) entitled *Distribución Geográfica y Huéspedes Conocidos de los Taquínidos de Chile* [Geographic Distribution and Known Hosts of Chilean Tachinids]. It was the summation of 25 years of collection-building on the part of the senior author and his examination of other collections within and outside (Cortés 1963) the country. The northernmost desert regions of Chile were excluded because only one species was known from there at the time. A careful compilation of distributions and hosts was given for 135 species and another 21 species were listed that were essentially known only from their type specimens and type localities. A further ten names were listed as *incertae sedis* following Cortés (1946). No new taxa were described. The monograph concluded with an interpretative discussion of the biogeographic patterns of tachinid distributions in Chile.

The arid northern portion of Chile was treated a couple of years later when Cortés and Campos (1971) published their findings on *Taquínidos de Tarapacá y Antofagasta*. This monograph recorded 33 genera and 53 species in these regions and described two genera and 15 species as new. A key to genera and a biogeographic discussion were included. One new species was named *Trichophoropsis* [= *Andicesa*] *sabroskyi* in recognition of Curtis Williams Sabrosky (1910–1997), a dipterist with the United States Department of Agriculture (at USNM) who had kindly provided them with information about types and answered their other questions for more than 15 years.

An addendum to Cortés and Campos (1971) was published by Cortés and Campos (1974) and added seven genera to the previous list (including one new genus) and nine species (including four new). A revised key to the genera of these northern regions was given. One new genus was named *Caltagironea* and dedicated to Leopoldo Enrique Caltagirone (1927–present) of the University of California Berkeley. Caltagirone (1953) had studied the life history and biological control potential of *Incamyiachilensis*. Caltagirone (1966) named a new species, *Opsophaguscortesi*, in honour of Cortés.

A second addendum to *Taquínidos de Tarapacá y Antofagasta* by Cortés and Hichins (1979) added new data on a number of previously reported species and also recorded two genera as new to the regions (one a new record for Chile). A third and last addendum was published by Cortés (1984). It brought the number of genera known from the regions to 47 and number of species to 70. One new genus and two new species were described and a new key to genera was included. In 2007, the Government of Chile divided the region of Tarapacá *sensu*Cortés and Hichins (1969, 1979) and Cortés and Campos (1971) into two regions, Arica & Parinacota and Tarapacá.

Chilean entomologist Nelson Hichins published on tachinids twice with Cortés (see above) and once in a sole-authored paper about a survey he conducted near Maipú, just west of Santiago (Hichins 1969). The specimens collected during that survey were identified by Cortés and 68 species were recorded. A few years earlier, Louis Marnef had described the new genus and species *Lafuentemyiayanezi* based on specimens collected east of Valparaíso by Hichins (Marnef 1965). Cortés (1967b) dedicated the species *Parasetigena* [= *Chetogena*] *hichinsi* to Hichins, who had collected the type series.

Luciano Elliot Campos (1927–1989) was an agricultural entomologist and later dean of the Facultad de Ciencias Agronómicas, Universidad de Chile, Santiago. He coauthored twice with Cortés in the 1970s (see above) but he had also published a paper on tachinids 20 years earlier (Campos 1953) that contained notes on species and the description of a new species, *Poliopsauratus*. Cortés (1967a) named the genus *Camposodes* in Campos’ honour. An informative and entertaining oral presentation by Campos entitled *Insects – Men and Environment in Chile* was reproduced in *Revista Chilena de Entomología* (Campos 1975).

Two American dipterists who ran Malaise traps throughout Chile in the 1960s and made their material available to specialists were honoured with generic patronyms by Cortés. *Irwinia*Cortés (1967a) [= *Phytomyptera*] was named for Michael Edward Irwin (1940–present) and *Schlingermyia*Cortés (1967b) was named for Evert Irving Schlinger (1928–2004).

An intriguing suggestion was made by Cortés (1983) that the “Trichoprosopini” (the members of which are now included in the Megaprosopini, Tachininae) are the sister group to the New Zealand “Occisorini” [= Proscissionini] based on shared morphological features and host associations. This would represent the first indication of a transantarctic relationship in the Tachinidae. More substantial morphological evidence has not been forthcoming to support this hypothesis and recent molecular evidence contradicts it (Stireman et al. 2019), as does the inferred timeline for tachinid diversification (Cerretti et al. 2017). Although this transantarctic relationship has not been substantiated, the formal transfer of the “Trichoprosopini” from Dexiinae (Guimarães 1971, as “Proseninae”) to Tachininae by Cortés (1986) has been accepted and followed by later authors.

This last mentioned work of Cortés (1986) was another significant regional study reminiscent of *Taquínidos de Tarapacá y Antofagasta* (Cortés and Campos 1971). This one dealt with the southern regions under the title *Taquínidos de Aysén (XI Region) y Magallanes* (*XII Region*) *Chile*. It contributed new information about the tachinids of these regions and was based in large part on material accumulated or examined since the monograph of Cortés and Hichins (1969). The fauna comprised 51 genera and 71 species, including four new genera and eight new species.

The size of the Chilean tachinid fauna was cited as 125 genera and 250 species in a short paper about “non-generic characters in Chilean tachinid flies” (Cortés 1989). That same year, Cortés and Herrera (1989) published a detailed history of key figures in Chilean entomology since earliest times and some of the people are discussed above because of their involvement, large or small, with tachinids.

One of the last papers published by Cortés was a review of Chilean Tachinidae and was co-authored by a young graduate student, Christian Raúl González (1963–present) (Cortés and González 1989). This student is now, more than 30 years later, a co-author on this catalogue of Chilean Tachinidae. González (2001) wrote a tribute to “Professor Raúl E. Cortés Peña” after his death that year. Cortés has been honoured with the following (and possibly more) patronyms: *Opsophagus* [= *Cyrtophloeba*] *cortesi*Caltagirone (1966; Chile), *Raulcortesia* Artigas & Papavero (1991; Chile, Asilidae), *Leschenaultiacortesi* Toma & Guimarães (2002; Venezuela), *Chaetocnephaliacortesi* González *in* González & Vergés (2004; Chile) and *Dasyuromyiacortesi*Gramajo (2011; Argentina).

William Robin Thompson (1887–1972) was a Canadian entomologist with a strong background in biological control. He was appointed director of the Farnham House Laboratory of the Imperial Institute of Entomology in England in 1928 and continued to head the Institute as it went through changes in organisation, name, and headquarters. By the time Thompson retired in 1958, it was known as the Commonwealth Institute of Biological Control and headquartered in Ottawa. Thompson then took up a new vocation and spent the next ten years (throughout his 70s) working on the eight volumes of *The Tachinids of Trinidad* (Thompson 1961–1968). The series was unique in incorporating features of larval tachinids for many species and was well illustrated thanks to the artistic talents of his wife Mary.

Thompson’s higher classification of Tachinidae was influenced more by the great contemporary masters of European Tachinidae, Louis-Paul Mesnil (1904–1986) and Benno Wilhelm Herting (1923–2004), than by Townsend’s *Manual of Myiology*. Nevertheless, he tended towards restricted genera in *The Tachinids of Trinidad* and described 41 from the island. Only 18 are still valid and not all of them have been re-evaluated. Most of the synonymy was proposed in Wood’s (1985) conspectus of the Blondeliini and as a result seven of Thompson’s generic names are listed in the catalogue below as synonyms of *Eucelatoria* and *Myiopharus*.

The Brazilian José Henrique Guimarães (1937–2008) was educated at agricultural schools in Rio de Janeiro before beginning the study of Tachinidae as an intern at the Laboratório de Zoologia Médica e Parasitologia da Escola Nacional de Veterinária and the Instituto Oswaldo Cruz, in Rio (Lamas et al. 2009). A series of five revisionary papers on *Archytas* resulted from these studies (Guimarães 1960, 1961a, b, 1963a, b) in addition to several papers on other tachinine tachinids. In 1963 he was hired as a biologist in São Paulo at what is now the Museu de Zoologia, Universidade de São Paulo (MZSP). A scholarship permitted Guimarães to spend 1967 at the Smithsonian Institution (USNM) to work on a much-needed catalogue of Neotropical Tachinidae. Not only were the resources of the Smithsonian available to Guimarães at this time, in particular the collection (including the majority of Townsend’s types) and library, but the taxonomic and nomenclatural advice of resident USDA dipterist Curtis Sabrosky (details above) was indispensable. The catalogue was completed over the next several years and published in the series *Catalogue of the Diptera of the Americas South of the United States* (Guimarães 1971). Guimarães left Washington for California to start a revision of North American *Winthemia* at the University of California Riverside for a Master’s degree. With that completed he returned to Brazil in 1970 to resume his former position and to begin study of the Mesembrinellidae for a Ph.D. degree, completed in 1973. Guimarães remained at the Museu de Zoologia until 1985 and then moved on to other positions before retiring in 1993 (Lamas et al. 2009). His sole-authored papers on Tachinidae ended in 1983 but he assisted with other tachinid papers up to 2002. Guimarães did not describe any Chilean Tachinidae but his revisions helped people like Cortés sort out the Chilean species of the groups that were revised.

Guimarães’ most influential and enduring contribution to tachinidology was his 1971 tachinid catalogue. Sabrosky and Arnaud (1965) had recently published on the Tachinidae in *A Catalog of the Diptera of America North of Mexico* and a companion catalogue for the Tachinidae of the southern portion of the Americas was sorely needed. This was not an easy task because the number of species was much greater and the existing classification (due in large measure to Townsend) was more bewildering. His result was a progressive catalogue, to a point. As Guimarães (1971: 3) conceded, the “catalogue arrangement leaves much to be desired”. This is true but the result must be viewed in the context of the task at hand, which was a compilation of data on a difficult fauna of close to 3000 species and ca. 950 genera. After 50 years this catalogue is still the chief resource for anyone studying Neotropical Tachinidae.

The Peruvian entomologist Luis A. Valencia (1945–present) has also contributed to Chilean Tachinidae. He co-authored with Cortés on a partial revision of the genus *Ateloglutus* Aldrich (Cortés and Valencia 1972), described the new genus and species *Velardemyiaica* (Valencia 1972a) and described a new species of *Winthemia* [*W.roblesi*, since synonymised with *W.singularis* Reinhard] (Valencia 1972b).

Argentine entomologist María Cecilia Gramajo (1973–present) has published several papers on the Tachinidae of Argentina. In a preliminary list of Tachinidae of Patagonian Argentina, Gramajo (1998) recorded 80 species of which ca. 20 were originally described from Chile and were newly recorded from Argentina. A paper on *Dasyuromyia* (Gramajo 2011) has a key to species that includes all but one of the Chilean species.

Xuekui Sun (1963–present) emigrated from China to Canada and completed a Ph.D. thesis on *Phasia* under the supervision of dipterist Stephen Archer Marshall (1954–present) at the University of Guelph, Ontario. The *Phasia* revision treated the species of the world except for the Neotropical ones. Seven generic names based on New World species were newly synonymised with *Phasia* in Sun and Marshall (2003) and are listed in the catalogue below.

The Brazilian tachinid specialist Silvio Shigueo Nihei (1976–present) at the Universidade de São Paulo has published primarily on the Brazilian fauna. A few of his publications are relevant to the Chilean fauna or to the list of generic synonyms in the catalogue below (Nihei 2015, 2016; Nihei and Dios 2016). A former student of Nihei’s, Brazilian Rodrigo de Vilhena Perez Dios (1987–present), published a revision of *Ectophasiopsis* that includes the single species known from Chile (Dios and Nihei 2017).

A prominent figure among Chilean entomologists was Luis Enrique Peña (1921–1995), a great explorer and professional collector with a vast knowledge of the country and its fauna and flora, particularly its insects. He worked for a short time at the Universidad de Chile in Santiago and elsewhere, but his true calling was travelling throughout Chile and neighbouring countries in search of insects, often assisting specialists from abroad who valued his expertise in the field. More than 400 species bear his name and he described more than 100 species of Tenebrionidae (Coleoptera). He did not name any tachinids but he collected many of them and his specimens are in Chilean collections, CNC and elsewhere. He collected the type series of both *Caltagironeavera* (Cortés and Campos 1974) and *Enchomyiapenai* [= *Comopsruficornis*] (Cortés 1967b) and the latter species was named in his honour.

The authors of this present catalogue have been involved with the Chilean Tachinidae to a greater or lesser degree as reviewed below.

Donald Montgomery [“Monty”] Wood (1933–2020) completed a Ph.D. degree on black flies (Simuliidae) at McMaster University in Hamilton, Ontario, in 1963 and was hired at CNC the following year to work on black flies and other families. Wood had been interested in tachinids for several years and the family came to dominate his research time and collecting activities. He soon realised that the New World classifications of Sabrosky and Arnaud (1965) and Guimarães (1971) were still overly influenced by Townsend’s *Manual of Myiology* and saw much promise in the restructuring of European Tachinidae that was underway by Mesnil and Herting (see above). The result was a great pruning of generic names in his *Taxonomic Conspectus of the Blondeliini of North and Central America and the West Indies* (Wood 1985) and tachinid chapter in *Manual of Nearctic Diptera* (Wood 1987; synonymy reviewed in O’Hara and Wood 1998). Together, these two works proposed close to 400 new generic synonyms for New World genera with species north of continental South America. Included among these were quite a few generic names that are listed as synonyms in the catalogue below. A later chapter on Tachinidae and key to genera in *Manual of Central American Diptera* (Wood and Zumbado 2010) only provides a small measure of assistance with the Chilean genera because a significant number of the genera are not included. There are a few generic names mentioned as synonyms for the first time in Wood and Zumbado (2010) and also the occasional inflation of species numbers that reflect unstated generic synonymies.

Wood retired in 1986 and was an honorary research associate with CNC until his death a few months before this manuscript was completed. He continued to collect and build his knowledge of New World Tachinidae after retirement and was working towards a revised generic classification of the entire fauna until the final months of his life. He collected extensively throughout the Neotropics and built a large and significantly curated private collection. He augmented his own collecting efforts with specimens purchased from professional collectors Fritz Plaumann (Brazil) and Luis Peña (Chile). Wood and wife Grace collected in Chile and Argentina with Luis Peña in late 1993 to early 1994 and returned to Ottawa with a broad assortment of beautifully-mounted tachinids that were mostly sorted and identified before being donated to the CNC during the past few years. These tachinids from Peña and M. and G. Wood were helpful in understanding the Chilean fauna during the preparation of this paper.

James Edward O’Hara (1952–present) first became interested in Tachinidae as a summer student at CNC in 1977 while pursuing a B.Sc. degree at nearby Carleton University. There he was influenced by Monty Wood to undertake a revision of the North American species of *Siphona* for a Master’s degree, which was completed at the University of Alberta, Edmonton, under the supervision of coleopterist George E. Ball in 1981. The *Siphona* revision included a new generic synonym (*Phantasiosiphona*) listed in the catalogue below and a cautionary note that the European species *Siphonageniculata* is likely misidentified from Chile and elsewhere in South America (O’Hara 1983). The tribe Siphonini was revised for a Ph.D. degree, also under the supervision of Ball and completed in 1987 (O’Hara 1989). In that revision, Chilean species formerly in *Actia* were moved to *Ceromya* and subgenera of *Siphona* were recognised. O’Hara was hired into his present position with CNC in 1989. A later revision of North American Polideini and a reinterpretation of the tribe is followed here (O’Hara 2002). Specimens collected in Chile by O’Hara in late 2015 (Stireman et al. 2016) helped with the preparation of this paper. The most recent version of the *Preliminary Checklist of the Tachinidae of the World* (O’Hara et al. 2020) includes the current names and distributions of Chilean Tachinidae.

Christian Raúl González (1963–present) (also see above) became interested in Tachinidae as a student at the Universidad Metropolitana de Ciencias de la Educación (UMCE) in Santiago in 1986. There, he was influenced by Raúl Cortés to prepare a *List of Tachinidae from Chile* for an undergraduate thesis, which was completed under the supervision of Cortés in 1988. González then worked on Tabanidae for a Master’s degree at the Universidad Metropolitana under the supervision of Sixto Coscarón. In 1989, González was hired into his present position at the same university. That same year, Cortés and González (1989) published a review of the Voriini of Chile, recognising nine genera and 16 species including a new genus with one new species (*Nothovoriapraestans*). Other single-authored contributions on the Chilean Tachinidae consisted of reviews of the genus *Ateloglutus* (González 1989) and former tribe Cuphocerini (now Polideini and Tachinini) (González 1992a), and a survey of the tachinids of Reserva Nacional de Río Clarillo near Santiago (González 1992b). Collaborations resulted in reviews of the Chilean species of *Incamyia* (González and Henry 1992) and the Chilean Goniini (González and Vergés 2004). The single new species described in the latter was named *Chaetocnephaliacortesi* in honour of González’s mentor.

## Materials and methods

### Format

This catalogue is arranged and formatted in a similar manner to the Tachinidae of the Afrotropical Region by O’Hara and Cerretti (2016). The sections here under Format are similar to the same sections in that work but are repeated here as a convenient guide and have been modified to apply to the Chilean Tachinidae. Any changes in format or interpretation of nomenclatural matters as compared to O’Hara and Cerretti (2016) are noted.

#### General

This catalogue cites all the species of Chile in their valid and original combinations, provides details about the name-bearing types of all nominal species, and gives known distributions. It is based on the examination of virtually all of the approximately 450 publications listed in the References.

Valid names are arranged hierarchically and alphabetically according to the categories of subfamily, tribe, genus, subgenus, and species. Synonyms are given for valid names of genera, subgenera, and species, and are listed chronologically. Synonymic lists comprise taxa described from south of the United States, synonyms that have been used as valid names in the literature on Chilean Tachinidae, and (where known and listed last) misidentifications and incorrect spellings.

Each genus-group name is listed with the following information: genus name in italics and capital letters (and additionally in bold if valid, unless misidentified from Chile; e.g., *Neoethilla* Cerretti et al.), author, year (with letter if applicable), page, note in parentheses if applicable (e.g., junior homonym or proposed as subgenus), type species with author and date, form of type fixation, and country (or region, such as Europe, if country unknown) of the type locality of the type species in square brackets (the last not given for all generic names in O’Hara and Cerretti 2016). Each type species is cited in its original binomen (Recommendation 67B of the *Code*, ICZN 1999), and if that name is a synonym then it is followed by the valid name of the species in parentheses. We have invoked Article 70.3.2 of the *Code* (ICZN 1999) to fix the intended species as the type species for generic names that were based on misidentified type species. This maintains the concepts of these generic names as currently accepted and in prevailing usage. The genera so affected are listed below under “Summary of new taxonomic and nomenclatural changes”.

Type species were fixed by original designation, monotypy, subsequent designation, or in a few instances subsequent monotypy, except for type species newly fixed here for nominal genera based on misidentified type species. Fixation by original designation requires an explicit designation of a type species (Article 68.2 of the *Code*, ICZN 1999), so a new genus “proposed for” or “erected for” a single species has its type species fixed by monotypy. A new genus proposed before 1931 for a single species and accompanied by the expression “gen. n., sp. n.” or an equivalent also has its type species fixed by monotypy (Article 68.2.1). If, on the other hand, the new genus is proposed for more than one new species and the expression “gen. n., sp. n.” or an equivalent is applied to only one of the new species, then that species is fixed as type species by original designation (Article 68.2.1).

Species are listed by valid name followed by the available name(s) associated with it; i.e., the available name of the valid name plus synonyms. The valid name is represented by the valid specific epithet in bold and italics (in italics only if questionably recorded or misidentified from Chile; e.g., *Archytasincertus* (Macquart)) followed by the author, date (no letter suffix), and known distribution. Author and date are enclosed in parentheses if the species has moved from its original genus. The distribution is given first for the Neotropical Region and then for other regions as explained under “Geographic divisions” and “Distributional data”. Each available name is given in italics in its original combination and spelling followed by author, year (with letter suffix if applicable to match a publication listed in the References), page, and a note in parentheses if applicable (e.g., junior homonym or subsequent spelling). A questionable synonym is preceded by a question mark (e.g., “? *Spathipalpusflavifrons* Rondani”). Given next is name-bearing type information consisting of status (holotype, lectotype, neotype, or syntypes), sex (of single type, or number and sex of syntypes), type depository (in parentheses), and type locality. If a neotype or lectotype was designated then a citation is given to the designation. Additional information may be given in parentheses with the type depository to cite the number and sex of syntypes existing in a collection if that number is different from the information given in the original description, or if the original description did not provide details about the type series; also, a reference may be cited wherein information can be found about the name-bearing type.

A subsequent spelling of a generic or specific name can be an incorrect subsequent spelling (which is not an available name) or an unjustified emendation (which is an available name with its own author and date). Incorrect subsequent spellings are cited where known to us but others surely exist. An unjustified emendation is cited with an author and date following O’Hara and Cerretti (2016); a name only was given in O’Hara and Wood (2004) and O’Hara et al. (2009) except in rare instances.

Notes and/or references are often given after genus and species entries. Notes provide explanations of some sort; e.g., priority of names, composition of type series, justification for a new combination or new name. References have a standardised format consisting of a source followed by the information provided therein; e.g., first records from countries (as explained under “Distributional data”), redescriptions, keys, figures, type notes. These references attempt to trace the history of name usage and synonymy but do not cite every occurrence of a name in species lists (unless it is a first record from a country).

The following abbreviations are used:

***Code****International Code of Zoological Nomenclature*, specifically the fourth edition published by the International Commission on Zoological Nomenclature in 1999; cited as ICZN 1999.

**ICZN** International Commission on Zoological Nomenclature.

**JEOH** James E. O’Hara.

**DMW** D. Monty Wood.

**CRG** Christian R. González.

#### Name-bearing types

We follow the same method developed by O’Hara et al. (2009) and followed by O’Hara and Cerretti (2016) for citing name-bearing type information for species described without a holotype designation in the original publication or without a subsequent lectotype or neotype designation. Details are provided about name-bearing types based on the content of the original descriptions and are not biased by existing type material in collections (that information being given in parentheses with the type depository). Our format for citing published data on name-bearing types other than a designated holotype, lectotype or neotype is as follows:

Type(s), male: One or more males. This citation is used for a species described from the male sex without indication of whether a single male (i.e., a holotype) or more than one male (i.e., syntypes) composed the type series.

Type(s), female: One or more females. See “Type(s), male”.

Type(s), unspecified sex: One or more specimens with no indication of sex.

Syntypes, [number] male[s] and [number] female[s] (e.g., “Syntypes, 3 males and 2 females”): Species described from an indicated number of males and females.

Syntypes, males and females: Species described from both sexes but the number of each sex was not given. A number in front of “males” with no number in front of “females” refers to the total number of males and females.

Syntypes, males: Species described from more than one male but without indication of the number of males.

Syntypes, females: Species described from more than one female but without indication of the number of females.

Syntypes, unspecified number and sex: Species described from more than one specimen but without indication of sex or number of specimens.

#### Avoidance of assumption of holotype

In following the foregoing format we have complied with Recommendation 73F of the *Code* (ICZN 1999), “Avoidance of assumption of holotype”, which states: “Where no holotype or syntype was fixed for a nominal species-group taxon established before 2000, and when it is possible that the nominal species-group taxon was based on more than one specimen, an author should proceed as though syntypes may exist and, where appropriate, should designate a lectotype rather than assume a holotype (see also Article 74.6)”. See O’Hara et al. (2009: 9–10) for a further discussion of this issue.

By following Recommendation 73F of the *Code*, assumed holotypes take on the status of syntypes. The recommendation favours “where appropriate” the designation of lectotypes. We have combined the spirit of Recommendation 73F and the provisions of Article 74.5 of the *Code* (ICZN 1999) to recognise certain published statements (as discussed in the next section) about assumed holotypes as lectotype fixations. This follows O’Hara et al. (2009) and O’Hara and Cerretti (2016) and is in our opinion the best way to reconcile assumed holotypes with the modern rules of nomenclature, while also giving credit of lectotype fixations to the authors who assumed holotypes.

#### Lectotypifications

There are two types of lectotypification in zoological nomenclature, explicit and implicit. In the former, a single syntype in a type series is designated as lectotype; in the latter, there is some form of statement that can be construed as the selection of a single name-bearing type. We follow O’Hara et al. (2009) in using the term “lectotype designation” for an explicit lectotypification and “lectotype fixation” for an implicit lectotypification. There is good reason to distinguish between the two because implicit lectotypifications are open to some interpretation, especially with respect to Article 74.5 of the *Code* (ICZN 1999: 82–83) that deals in part (see also Article 74.6) with lectotype designations before 2000:

“In a lectotype designation made before 2000, either the term ‘lectotype’, or an exact translation or equivalent expression (e.g. ‘the type’), must have been used or the author must have unambiguously selected a particular syntype to act as the unique name-bearing type of the taxon. When the original work reveals that the taxon had been based on more than one specimen, a subsequent use of the term ‘holotype’ does not constitute a valid lectotype designation unless the author, when wrongly using that term, explicitly indicated that he or she was selecting from the type series that particular specimen to serve as the name-bearing type”.

What constitutes a valid lectotypification (or lectotype fixation in our terminology) in the foregoing is largely dependent on how one interprets the passage about an author explicitly indicating “that he or she was selecting from the type series that particular specimen to serve as the name-bearing type”. At one end of the spectrum is the mere mention of a “holotype” or “type” by a subsequent author when the original type series clearly consisted of two or more syntypes. This statement does not constitute a lectotype fixation because the “holotype” is not distinguishable from other syntypes. At the other end of the spectrum is the mention of a “holotype” or “type” with accompanying details about its labelling, features, damage, etc. that clearly distinguishes that specimen from other syntypes; or perhaps there is only one type specimen in a collection and it is an “assumed holotype” (see section above) for a species described from an unspecified number of specimens. We considered these latter statements about a single type to qualify as lectotype fixations under Article 74.5 because they contain an explicit indication that an author accepted the cited “holotype” or “type” as the name-bearing type and restricted the term to a single recognisable specimen in a collection.

O’Hara and Cerretti (2016) recognised lectotype fixations in Townsend’s *Manual of Myiology* (Parts I–XII, 1934–1942), reversing the practice of O’Hara et al. (2009). We follow the former authors in recognising lectotype fixations in *Manual of Myiology* if there is a strong possibility of the lectotype being recognised in the stated collection. See O’Hara and Cerretti (2016: 11–12) for a more detailed discussion of this subject.

#### Type localities

Type localities are cited first by country and then by location within the country from larger to smaller geographic area or place. Spellings of geographic areas and places follow *The Times Comprehensive Atlas of the World* (Times Books 2007), if found in that work. Modern names and spellings are given where these have been determined. Country and higher administrative subdivisions (i.e., regions and provinces of Chile, provinces of Argentina, states of Brazil, regions of Peru, etc.) are given only in their modern equivalents. For locality names that have changed since they were first published, the modern spelling is given first followed by the original spelling in square brackets and quotes; e.g., Puerto del Hambre [as “Port Famine”]. Elevations are cited in metres (m) or feet (ft) as given by the author. Coordinates given in an original publication are cited in parentheses after the type locality; e.g., Chile, Arica y Parinacota, Parinacota, Putre, 3530 m (18°12′S, 69°35′W). Coordinates are included for many type localities that we had difficulty locating and these are given in square brackets after the locality to distinguish them from coordinates provided by an author; e.g., Chile, Valparaíso, Marga Marga, Bosque Los Perales [as “Perales”, ca. 33°9′S, 71°18′W]. Criteria for citing type localities in Sweden are explained in O’Hara et al. (2009: 11). The few localities we could not find are given in quotes; e.g., Trinidad, “Legerville Mt.”. A variety of resources were used to locate type localities not found in *The Times Comprehensive Atlas of the World* including other atlases, maps, literature, and Internet searches (often for the locality and/or collector).

Type localities in Chile are preceded by region and province (e.g., Valparaíso, Marga Marga). Those in other countries are preceded by province (Argentina, Ecuador, etc.), state (Brazil, Mexico, etc.) or department (Peru, Uruguay, etc.), or by no higher administrative division (e.g., countries of the West Indies).

### Collections housing name-bearing types

The location of the name-bearing type (holotype, lectotype, neotype, or syntypes) is cited for each nominal species, where known. The collections housing these name-bearing types are listed below with the abbreviations used in the text. We largely accepted as accurate the statements about the deposition of name-bearing types given in the original literature unless we had reason to doubt the information given (e.g., types known to have been relocated or presumed lost).

The abbreviations of collections cited in this work are as follows:

**AMNH** American Museum of Natural History, New York, USA.

**CAS** California Academy of Sciences, San Francisco, California, USA.

**CNC** Canadian National Collection of Insects, Arachnids and Nematodes, Agriculture and Agri-Food Canada, Ottawa, Canada.

**CUIC** Cornell University Insect Collection, Department of Entomology, Cornell University, Ithaca, New York, USA.

**EEAM** Estación Experimental Agronómica, Universidad de Chile, Maipú, Santiago, Chile. Cited as CEA in publications of R. Cortés.

**EESC** Estación Experimental San Camilo [formerly Estación Experimental Agrícola], Ica, Peru.

**INLA** INIA Subestación Experimental Control Biológico La Cruz, La Cruz, Chile. Cited as CENE [Estación Nacional de Entomología de La Cruz] in Cortés (1967b), Cortés and Hichins (1969) and Cortés and Campos (1971).

**INTA** Instituto Nacional de Tecnología Agropecuaria, Castelar, Argentina.

**MACN** Museo Argentino de Ciencias Naturales “Bernardino Rivadavia”, Buenos Aires, Argentina. Cited as MAHN in Cortés (1963).

**MCZ** Museum of Comparative Zoology, Harvard University, Cambridge, Massachusetts, USA.

**MEUC** Museo Entomológico Luis Peña del Departamento de Sanidad Vegetal de la Facultad de Ciencias Agronómicas, Universidad de Chile, La Pintana, Santiago, Chile. Cited as DSV [Departamento de Sanidad Vegetal] in Cortés (1945c–e) and as CFA [Colección de la Facultad de Agronomía] in later publications of R. Cortés.

**MLPA** Museo de La Plata, Universidad Nacional de La Plata, La Plata, Argentina.

**MNHN** Muséum National d’Histoire Naturelle, Paris, France.

**MNNC** Museo Nacional de Historia Natural, Santiago, Chile. Cited as CNI [Colección Nacional de Insectos, Ministerio de Agricultura, Santiago] in Cortés (1951b) and Campos (1953).

**MZSP** Museu de Zoologia, Universidade de São Paulo, São Paulo, Brazil.

**MZUF** Museo Zoologico “La Specola”, Firenze [Florence], Italy.

**MZUT** Museo e Istituto di Zoologia Sistematica dell’Università di Torino, Turin, Italy.

**NHMUK** Natural History Museum, London, United Kingdom. Frequently cited as BMNH [British Museum (Natural History)] in previous publications.

**NHMW** Naturhistorisches Museum Wien, Wien [Vienna], Austria.

**NHRS** Naturhistoriska Riksmuseet [Swedish Museum of Natural History], Stockholm, Sweden.

**NMPC** National Museum, Natural History Museum, Prague, Czech Republic.

**RBINS** Royal Belgian Institute of Natural Sciences, Bruxelles [Brussels], Belgium. Frequently cited as IRSNB [Institut Royal des Sciences Naturelles de Belgique] in former publications.

**RMNH** Naturalis Biodiversity Center, Leiden, Netherlands [formerly Nationaal Natuurhistorisch Museum and before that Rijksmuseum van Natuurlijke Historie]. The Zoölogisch Museum of the University of Amsterdam [ZMAN] has closed and its collections were merged with those of RMNH.

**SDEI** Senckenberg Deutsches Entomologisches Institut, Leibniz-Zentrums für Agrarlandschaftsforschung, Müncheberg, Germany.

**SEMC** Snow Entomological Museum Collection, KU Biodiversity Institute, University of Kansas, Lawrence, Kansas, USA.

**SENASA** Laboratorio de Sanidad Vegetal, Servicio Nacional de Sanidad Agraria, Lima, Peru.

**SMF** Forschungsinstitut und Naturmuseum Senckenberg, Frankfurt am Main, Germany.

**UMCE** Universidad Metropolitana de Ciencias de la Educación, Santiago, Chile.

**USNM** National Museum of Natural History [formerly United States National Museum], Smithsonian Institution, Washington, USA.

**UVVC** Facultad de Ciencias, Universidad de Valparaíso, Valparaíso, Chile. Cited as “Instituto de Biología, Universidad de Chile, Valparaíso” in Marnef (1965) and as CDCV [Colección del Departamento de Ciencias, Universidad de Chile, Valparaíso] in Cortés and Hichins (1969).

### Imaging of specimens

Habitus images of *Billaea* Robineau-Desvoidy name-bearing types (Fig. 3a–d) were taken by DMW during a visit to USNM in 2011. The equipment used was not recorded and specimens were not measured.

Specimens shown in Figs 4–6 belong to CNC and were imaged using a Canon EOS 70D Digital SLR camera body mounted on a Kaiser RS1 copy stand. A Canon EF 100 mm f/2.8 macro lens was used to image the medium to large specimens and a Canon MP-E 65 mm 1–5× macro lens was used for small specimens. Helicon Remote software was used to tether the camera to a computer to capture images remotely and control lens functions (shutter, shutter speed, aperture, and focus). Lighting was provided by a ring light comprising 80 LEDs with a specimen holder in the middle. A dome cover with a reflective white coating was placed over the ring light and a hole in the centre of the dome permitted images to be taken of the specimen within. A series of images were captured using the Helicon Remote software paired with a Stackshot Macro Rail hardware package by Cognisys. The series of images was stacked using Zerene Stacker and final images were prepared in Adobe Photoshop Creative Cloud 2018. Specimen measurements given in the captions to Figs 4–6 refer to body length from the pedicel of the antenna to the tip of the abdomen, excluding setae.

### Geographic divisions

The known distribution of each tachinid species recorded from Chile is given next to the valid name in the following order: Neotropical Region, Nearctic Region, Palaearctic Region, Afrotropical Region, Oriental Region, and Australasian and Oceanian regions. These regions are delimited and mapped in O’Hara et al. (2020: 8–26). Each region is subdivided according to the scheme explained below, with the Neotropical Region subdivided more finely than the other regions. Geographical names of countries, political divisions within countries, places, and topographical features follow *The Times Comprehensive Atlas of the World* (Times Books 2007), if given therein. The abbreviations and names given below are those used for the distributions given in the catalogue.

#### Neotropical Region (Figs 1, 2)

Greater Antilles (part of the West Indies).

Bahamas; Cayman Islands (United Kingdom Overseas Territory); Cuba; Dominican Republic; Haiti; Jamaica; Puerto Rico; Turks & Caicos (United Kingdom Overseas Territory).

eastern Lesser Antilles (Leeward and Windward islands in the Lesser Antilles of the West Indies).

Anguilla (United Kingdom Overseas Territory); Antigua [Antigua & Barbuda] (including Redonda); Barbados; Dominica; Grenada; Guadeloupe (including Marie-Galante, La Désirade, Îles des Saintes) (France); Martinique (France); Montserrat (United Kingdom Overseas Territory); Saba (Netherlands); Saint-Barthélemy (France); Saint Kitts [Saint Kitts and Nevis]; Saint Lucia; Saint-Martin (comprising Saint Martin [France] and Sint Maarten [Netherlands]); Saint Vincent [Saint Vincent and The Grenadines]; Sint Eustatius (Netherlands); Virgin Islands (including the United States islands of Saint Thomas, Saint John, and Saint Croix, and the British Virgin Islands of Tortola, Virgin Gorda, Anegada, and Jost Van Dyke).

southern Lesser Antilles (islands north of the Venezuelan coast in the Lesser Antilles of the West Indies).

Aruba (Netherlands); Blanquilla (Venezuela); Bonaire (Netherlands); Curaçao (Netherlands); Los Roques Archipelago (Venezuela); Los Testigos (Venezuela); Margarita (including smaller neighbouring islands, principally La Tortuga, Coche, and Cubagua; all comprising Nueva Esparta state, Venezuela); Trinidad & Tobago.

Middle America (mainland Middle America).

Belize; Costa Rica; El Salvador; Guatemala; Honduras; Mexico; Nicaragua; Panama.

South America. [Cited as South America when more detailed distributional data is not available.]

Argentina; Bolivia; Brazil; Chile (excluding Juan Fernández Islands); Colombia; Ecuador (excluding Galápagos Islands); Falkland Islands (disputed United Kingdom Overseas Territory); French Guiana (France); Juan Fernández Islands (Chile); Galápagos Islands (Ecuador); Guyana; Paraguay; Peru; South Georgia (including the South Sandwich Islands; disputed United Kingdom Overseas Territory); Suriname; Uruguay; Venezuela.

**Figure 1.**
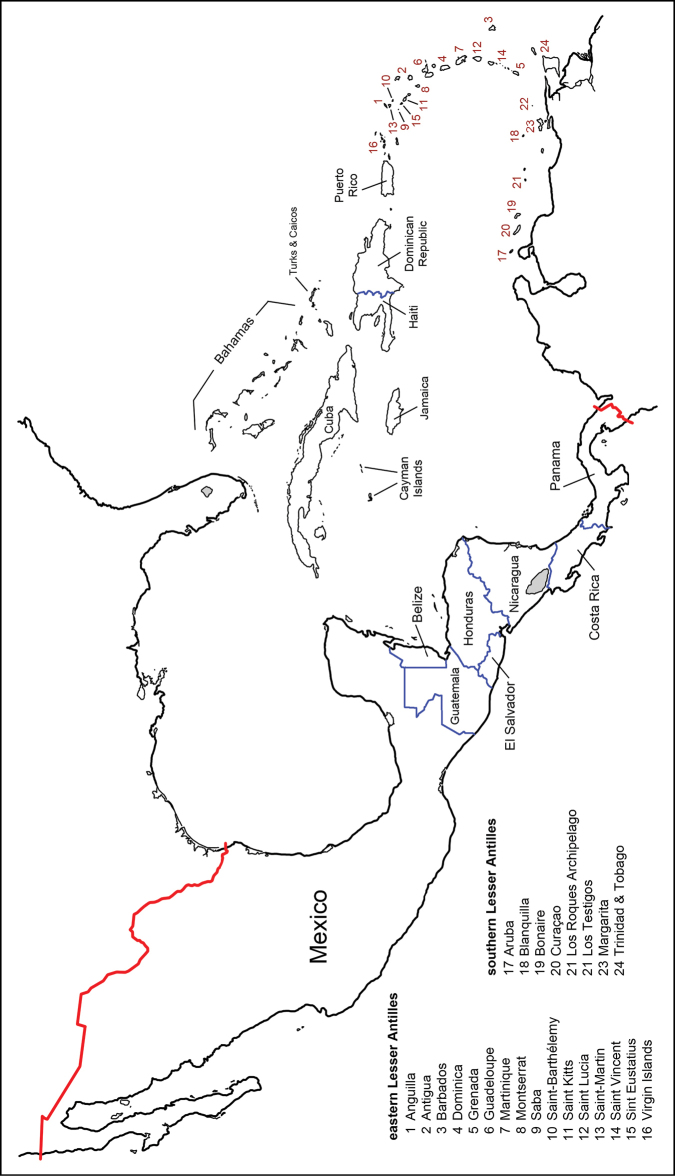
Countries and major islands of the Middle American portion of the Neotropical Region.

**Figure 2.**
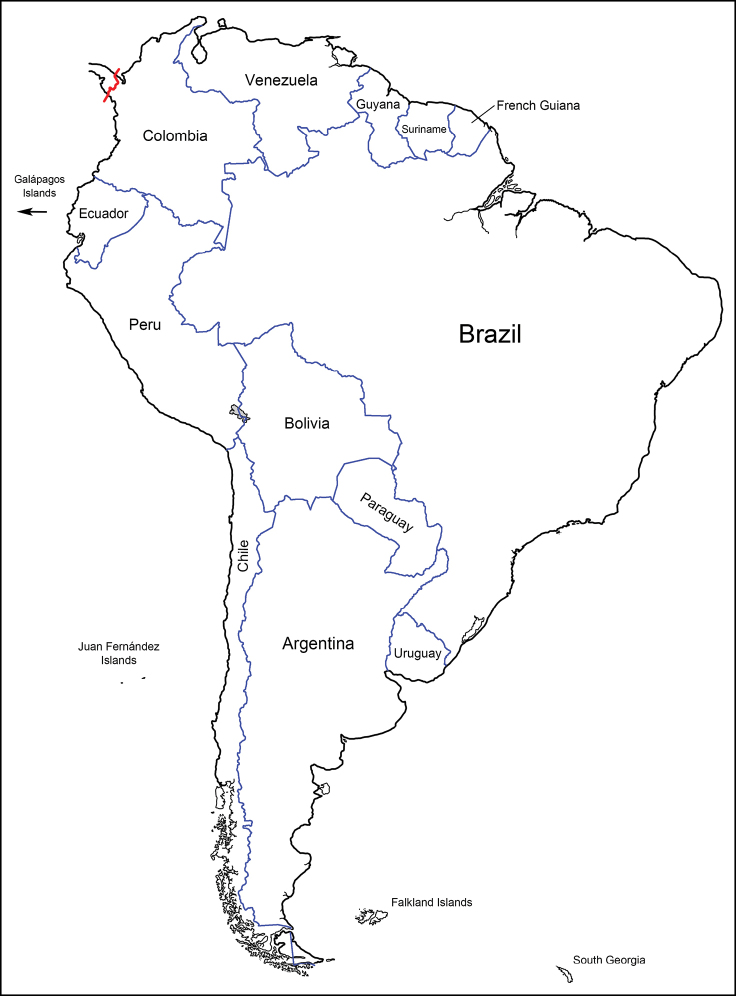
Countries and major islands of the South American portion of the Neotropical Region.

#### Nearctic Region

The limits of the Nearctic Region follow O’Hara et al. (2020: 8, 18 [map 1]) and include the following subdivisions: Bermuda (United Kingdom Overseas Territory); Canada; Greenland (Denmark); United States [United States of America, as “USA” for type localities; Hawaii as part of Australasian and Oceanian regions].

#### Palaearctic Region

See O’Hara et al. (2020: 11, 21 [map 4], 22 [map 5]) for the countries included in the broader subdivisions recognised here: Central Asia; China (Palaearctic part, *sensu*O’Hara et al. 2020); Europe; Japan (excluding Ryukyu Islands); Kazakhstan; Korean Peninsula; Middle East; Mongolia; North Africa; Russia; Transcaucasia.

#### Afrotropical Region

This region is subdivided by country, as explained and mapped in O’Hara and Cerretti (2016: 15, 16 [fig. 1]) and O’Hara et al. (2020: 13, 23 [map 6]).

#### Oriental Region

The Oriental Region is bounded on the north by the Palaearctic Region (O’Hara et al. 2020) and on the south by Weber’s Line (Evenhuis 1989: 31). See O’Hara et al. (2020: 15, 22 [map 5], 24 [map 7], 25 [map 8]) for a list of countries/subdivisions and maps.

#### Australasian and Oceanian regions

The Australasian and Oceanian regions are bounded on the north by Weber’s Line (Evenhuis 1989: 31). See O’Hara et al. (2020: 16, 26 [map 9]) for a list of countries/subdivisions and maps.

#### Sample distribution

A species recorded from all regions and subdivisions recognised here would be cited with the following distribution:

Neotropical: Greater Antilles (Bahamas, Cayman Islands, Cuba, Dominican Republic, Haiti, Jamaica, Puerto Rico, Turks & Caicos), eastern Lesser Antilles (Anguilla, Antigua, Barbados, Dominica, Grenada, Guadeloupe, Martinique, Montserrat, Saba, Saint-Barthélemy, Saint Kitts, Saint Lucia, Saint-Martin, Saint Vincent, Sint Eustatius, Virgin Islands), southern Lesser Antilles (Aruba, Blanquilla, Bonaire, Curaçao, Los Roques Archipelago, Los Testigos, Margarita, Trinidad & Tobago), Middle America (Belize, Costa Rica, El Salvador, Guatemala, Honduras, Mexico, Nicaragua, Panama), South America (Argentina, Bolivia, Brazil, Chile, Colombia, Ecuador, Falkland Islands, French Guiana, Juan Fernández Islands, Galápagos Islands, Guyana, Paraguay, Peru, South Georgia, Suriname, Uruguay, Venezuela). Nearctic: Bermuda, Canada, Greenland, United States. Palaearctic: Central Asia, China [Pal.], Europe, Japan, Kazakhstan, Korean Peninsula, Middle East, Mongolia, North Africa, Russia, Transcaucasia. Oriental: Andaman & Nicobar Islands, Bangladesh, Brunei, Bhutan, Cambodia, China [Orien.], Christmas & Cocos Islands, India, Indonesia [Orien.], Japan [Ryukyu Islands], Laos, Malaysia, Maldives etc., Myanmar, Nepal, Pakistan, Philippines, Singapore, Sri Lanka, Taiwan, Thailand, Vietnam. Australasian & Oceanian: Australia, Hawaii, Indonesia [Aust.], Melanesia, Micronesia, New Zealand, Papua New Guinea, Polynesia.

### Distributional data

#### Distributions within the Neotropical Region

Distributions are cited at the country level within the Neotropical Region (as listed in Geographic Divisions section) for each species based on published records and our examination of specimens in CNC and UMCE. The principal sources for published records were Aldrich (1934) and the publications of Cortés (and co-authors, 1944–1992, mainly Chile) and Blanchard (1935–1966, mainly Argentina). The CNC has more than 3000 Chilean specimens but few have been reported in the literature. These mostly originated from the following sources: nearly 2000 collected by the esteemed Chilean entomologist Luis E. Peña [Guzmán] from 1959–1980 and 1994–1995 (privately sold to DMW over a period of years and recently donated to CNC); ca. 1000 collected by DMW and wife Grace between December 1993 and February 1994 (recently donated to CNC and including ca. 150 specimens from Argentina); and over 200 collected by JEOH in December 2015 (see Stireman et al. 2016).

A reference is cited, if known, for the first record of a species from countries different from the one(s) from which the species was described. Subsequent records from the same country are not generally given unless significant in some way. The first record is considered the most important because it is sometimes the source for later records even if it was based on a misidentification.

## Classification

### Summary of new taxonomic and nomenclatural changes

#### Genera newly recorded from Chile

Two genera are newly recorded from Chile (one also newly recorded from Argentina).

*Chaetoepalpus* Vimmer & Soukup, 1940 (based on new records of *Chaetoepalpuscoquilleti* Vimmer & Soukup, 1940). New records from Argentina and Chile.

*Patelloa* Townsend, 1916 (based on new synonymy of *Macropatelloa* Townsend, 1931 with *Patelloa*). New record from Chile.

#### Species newly recorded from Chile

The following species are newly recorded from Chile or other countries.

*Lyphaornata* Aldrich, 1934. New record from Chile.

*Chaetoepalpuscoquilleti* Vimmer & Soukup, 1940. New records from Argentina and Chile.

*Phytomypteraevanescens* (Cortés, 1967). New record from Argentina.

*Xanthobasisunicolor* Aldrich, 1934. New record from Chile.

#### Species misidentified or misrecorded from Chile

Species newly recognised as misidentified or misrecorded from Chile are listed here. The reasons for not recognising them from Chile are given under each name in the catalogue.

*Archytasincertus* (Macquart, 1851).–Not Chile [Argentina, Brazil, Paraguay, Uruguay].

*Archytasseminiger* (Wiedemann, 1830).–Not Chile [Brazil, Colombia].

*Goniacrassicornis* (Fabricius, 1794).–Not Chile [Brazil, Peru, Venezuela; also Middle America, West Indies and Nearctic].

*Lespesiaandina* (Bigot, 1888), *nomen dubium*.–Not Chile [Cuba].

*Lespesiaarchippivora* (Riley, 1871).–Not Chile [widespread throughout the Nearctic Region and most of Middle and South America].

*Neoethillaignobilis* (van der Wulp, 1890).–Not Chile [Mexico; United States].

Siphona (Siphona) geniculata (De Geer, 1776).–Not Chile [Nearctic (introduced), Palaearctic].

*Winthemiaquadripustulata* (Fabricius, 1794).–Not Chile [Palaearctic; also Nearctic and Oriental].

#### First Reviser actions

*Billaea* Robineau-Desvoidy, 1830

*Paratheresiarufiventris* Townsend, 1929 and *Sarcoprosenarufiventris* Townsend, 1929 are secondary homonyms when placed together in *Billaea*. As the First Reviser (Article 24.2.2 of the *Code*, ICZN 1999), we fix *Paratheresiarufiventris* as the senior homonym.

*Myiopharus* Brauer & Bergenstamm, 1889

*Mayophoriniaangusta* Townsend, 1927 and *Metarrhinomyiaangusta* Townsend, 1927 are secondary homonyms when placed together in *Myiopharus*. As the First Reviser (Article 24.2.2 of the *Code*, ICZN 1999), we fix *Mayophoriniaangusta* as the senior homonym.

#### New replacement names

Eight new names are proposed for preoccupied names that came to our attention during the preparation of this catalogue and belong to genera recorded from Chile. The preoccupied names do not concern Chilean species except for one but are renamed to avoid the confusion of having two Neotropical species with the same name in the same genus. The etymology of each new name is given in the catalogue. The country of the type locality of each preoccupied species name is given at the end of each entry.

*Billaearufescens* O’Hara & Wood is proposed as a *nomen novum* for *Sarcoprosenarufiventris* Townsend, 1929, a name preoccupied in the genus *Billaea* Robineau-Desvoidy, 1830 by *Paratheresiarufiventris* Townsend, 1929 [Peru]. **Nom. nov.**

*Billaeatriquetrus* O’Hara & Wood is proposed as a *nomen novum* for *Sarcoprosenatriangulifera* Townsend, 1927, a name preoccupied in the genus *Billaea* Robineau-Desvoidy, 1830 by *Dexiatriangulifera* Zetterstedt, 1844 [Peru]. **Nom. nov.**

*Eucelatorianudioculata* O’Hara & Wood is proposed as a *nomen novum* for *Eucelatorioideanigripalpis* Thompson, 1968, a name preoccupied in the genus *Eucelatoria* Townsend, 1909 by *Chetolyganigripalpis* Bigot, 1889 [Trinidad]. **Nom. nov.**

*Eucelatoriaoblonga* O’Hara & Wood is proposed as a *nomen novum* for *Urodexodeselongatum* Cortés & Campos, 1974, a name preoccupied in the genus *Eucelatoria* Townsend, 1909 by *Exoristaelongata* van der Wulp, 1890 [Chile]. **Nom. nov.**

*Lespesiathompsoni* O’Hara & Wood is proposed as a *nomen novum* for *Sturmiopsoideaobscura* Thompson, 1966, a name preoccupied in the genus *Lespesia* Robineau-Desvoidy, 1863 by *Eurigasterobscurus* Bigot, 1857 [Cuba]. **Nom. nov.**

*Myiopharuscharapensis* O’Hara & Wood is proposed as a *nomen novum* for *Metarrhinomyiaangusta* Townsend, 1927, a name preoccupied in the genus *Myiopharus* Brauer & Bergenstamm, 1889 by *Mayophoriniaangusta* Townsend, 1927 [Peru]. **Nom. nov.**

*Myiopharusincognitus* O’Hara & Wood is proposed as a *nomen novum* for *Stenochaetaclaripalpis* Thompson, 1968, a name preoccupied in the genus *Myiopharus* Brauer & Bergenstamm, 1889 by *Neoxynopsoideaclaripalpis* Thompson, 1968 [Trinidad]. **Nom. nov.**

*Myiopharusrufopalpus* O’Hara & Wood is proposed as a *nomen novum* for *Paralispepalpalis* Townsend, 1929, a name preoccupied in the genus *Myiopharus* Brauer & Bergenstamm, 1889 by *Myioxynopspalpalis* Townsend, 1927 [Peru]. **Nom. nov.**

#### New type species fixations

Article 70.3.2 of the *Code* (ICZN 1999) allows the type species of a nominal genus to be fixed as the species intended by the original author if the type species designated by that author was misidentified. We have invoked Article 70.3.2 for the three instances of misidentified type species in this catalogue that had not been dealt with previously (e.g., O’Hara and Wood 2004) to preserve the current concepts of the genera involved. Type species are fixed for the following nominal genera (see catalogue for further details).

*Parafabricia* Brauer & Bergenstamm, 1894: 612 [also 1895: 76]. Type species newly fixed as *Parafabriciaperplexa* Townsend, 1931. Synonym of *Archytas* Jaennicke, 1867.

*Tachinodes* Brauer & Bergenstamm, 1889: 133 [also 1889: 65]. Type species newly fixed as *Juriniametallica* Robineau-Desvoidy, 1830. Synonym of *Archytas* Jaennicke, 1867.

*Willistonia* Brauer & Bergenstamm, 1889: 97 [also 1890: 29]. Type species newly fixed as *Willistoniaaldrichi* Townsend, 1931. Synonym of *Belvosia* Robineau-Desvoidy, 1830.

#### Lectotype designations

Lectotypes are designated for four nominal species (see Lectotype Designations section).

*Echinomyiapygmaea* Macquart, 1851. This is a valid name in the genus *Peleteria* Robineau-Desvoidy, 1830, as *Peleteriapygmaea* (Macquart).

*Goniachilensis* Macquart, 1844. This is a junior synonym in the genus *Gonia* Meigen, 1803. The valid name of the species is *Goniapallens* Wiedemann, 1830.

*Masiceraauriceps* Macquart, 1844. This is a valid name in the genus *Lespesia* Robineau-Desvoidy, 1863, as *Lespesiaauriceps* (Macquart).

*Prosopochoetanitidiventris* Macquart, 1851. This is a valid name in the genus *Prosopochaeta* Macquart, 1851.

#### New and revived combinations

New and revived combinations proposed in this work are listed below. These are based

on the study of type material, authoritatively identified specimens, and/or descriptions

and figures in the literature, mostly by DMW.

*Blepharipezaandina* Bigot, 1888 is moved from an unplaced species in “Sturmiini” or Tachinidae to *Lespesia* Robineau-Desvoidy, 1863 as a *nomen dubium*. Distribution: Cuba (not Chile as published). **Comb. nov.**

*Camposodesevanescens* Cortés, 1967 is moved from its original placement in *Camposodes* Cortés, 1967 to *Phytomyptera* Rondani, 1845. Distribution: Argentina, Chile. **Comb. nov.**

*Ectophasiopsisypiranga* Dios & Nihei, 2017 is moved from its original placement in *Ectophasiopsis* Townsend, 1915 to *Trichopoda* Berthold, 1827 (and assigned to subgenus Galactomyia Townsend, 1908). Distribution: Argentina, Brazil. **Comb. nov.**

*Embiomyiaaustralis* Aldrich, 1934 (type species of *Embiomyia* Aldrich, 1934) is moved from its original placement in *Embiomyia* to *Steleoneura* Stein, 1924 (with *Embiomyia* in synonymy). Distribution: Argentina, Chile. **Comb. nov.**

*Eurigastermodestus* Bigot, 1857 is moved from its position in unplaced species of Exoristinae (as “Goniinae”) by Guimarães (1971: 215) to *Lespesia* Robineau-Desvoidy, 1863. Distribution: Cuba. **Comb. nov.**

*Eurigasterobscurus* Bigot, 1857 is moved from its position in unplaced species of Exoristinae (as “Goniinae”) by Guimarães (1971: 215) to *Lespesia* Robineau-Desvoidy, 1863. Distribution: Cuba. **Comb. nov.**

*Macropatelloatanumeana* Townsend, 1931 (type species of *Macropatelloa* Townsend, 1931) is moved from its original placement in *Macropatelloa* to *Patelloa* Townsend, 1916 (with *Macropatelloa* in synonymy). Distribution: Argentina, Chile. **Comb. nov.**

*Masicerainsignis* van der Wulp, 1882 is moved from its placement in *Sturmia* Robineau-Desvoidy, 1830 by previous authors (e.g., Cortés and Hichins 1969: 59; Guimarães 1971: 192; Henry 1987: 200) to *Drino* Robineau-Desvoidy, 1863. Distribution: Argentina, Chile. **Comb. nov.**

*Parasetigenahichinsi* Cortés, 1967 is moved from its original placement in *Parasetigena* Brauer & Bergenstamm, 1891 to *Chetogena* Rondani, 1856. Distribution: Chile. **Comb. nov.**

*Parasetigenaporteri* Brèthes, 1920 is moved from its placement in *Stomatotachina* Townsend, 1931 by previous authors (e.g., Guimarães 1971: 160; Mulieri et al. 2013: 169) to *Chetogena* Rondani, 1856. Distribution: Chile. **Comb. nov.**

*Phoroceracalyptrata* Aldrich, 1934 is moved from uncertain placements by Guimarães (1971: 152, unplaced in Blondeliini), Henry (1987: 206, in *Phorocera* Robineau-Desvoidy, 1830 but genus unplaced in Tachinidae) and González (1992b: 183, in *Phorocera* but genus unplaced in Exoristinae [as “Goniinae”]) to *Admontia* Brauer & Bergenstamm, 1889. Distribution: Argentina, Chile. **Comb. nov.**

*Poliopsauratus* Campos, 1953 is moved from its original placement in *Poliops* Aldrich, 1934 to *Admontia* Brauer & Bergenstamm, 1889. Distribution: Chile. **Comb. nov.**

*Poliopsstriatus* Aldrich, 1934 is moved from its original placement in *Poliops* Aldrich, 1934 to *Admontia* Brauer & Bergenstamm, 1889. Distribution: Argentina, Chile. **Comb. nov.**

*Ruiziellafrontosa* Cortés, 1951 is moved from its original placement in *Ruiziella* Cortés, 1951 to *Chaetoepalpus* Vimmer & Soukup, 1940, where it is placed in synonymy with *C.coquilleti* Vimmer & Soukup, 1940, **syn. nov.** Distribution of *C.coquilleti*: Argentina, Chile, Peru. **Comb. nov.**

*Ruiziellaluctuosa* Cortés, 1951 is moved from its original placement in *Ruiziella* Cortés, 1951 to *Chaetoepalpus* Vimmer & Soukup, 1940. Distribution: Argentina, Chile. **Comb. nov.**

*Sarcoprosenaluteola* Cortés & Campos, 1974 is moved from its original placement in *Sarcoprosena* Townsend, 1927 to *Billaea* Robineau-Desvoidy, 1830. Distribution: Chile. **Comb. nov.**

*Sarcoprosenarufiventris* Townsend, 1929 is moved from its original placement in *Sarcoprosena* Townsend, 1927 to *Billaea* Robineau-Desvoidy, 1830. **Comb. nov.** The name *S.rufiventris* is a junior secondary homonym of *Paratheresiarufiventris* Townsend, 1929 when placed in *Billaea* and is renamed herein as *Billaearufescens* O’Hara & Wood, **nom. nov.** Distribution: Peru.

*Sarcoprosenatriangulifera* Townsend, 1927 (type species of *Sarcoprosena* Townsend, 1927) is moved from its original placement in *Sarcoprosena* to *Billaea* Robineau-Desvoidy, 1830 (with *Sarcoprosena* in synonymy). **Comb. nov.** The name *S.triangulifera* is a junior secondary homonym of *Dexiatriangulifera* Zetterstedt, 1844 when placed in *Billaea* and is renamed herein as *Billaeatriquetrus* O’Hara & Wood, **nom. nov.** Distribution: Peru.

*Saundersiaaurea* Giglio-Tos, 1893 is moved from its placement in *Epalpus* Rondani, 1850 by Guimarães (1971: 64) to “Unplaced species of Tachinini”. Distribution: Mexico. **Comb. nov.**

*Schistostephanaaurifrons* Townsend, 1919 (type species of *Schistostephana* Townsend, 1919) is moved from its original placement in *Schistostephana* to *Billaea* Robineau-Desvoidy, 1830 (with *Schistostephana* in synonymy). Distribution: Peru. **Comb. nov.**

*Siphoactiacharapensis* Townsend, 1927 (type species of *Siphoactia* Townsend, 1927) is moved from its original placement in *Siphoactia* to *Clausicella* Rondani, 1856 (with *Siphoactia* in synonymy). Distribution: Peru. **Comb. nov.**

*Siphoactiaperegrina* Cortés & Campos, 1971 is moved from its original placement in *Siphoactia* Townsend, 1927 to *Clausicella* Rondani, 1856. Distribution: Chile. **Comb. nov.**

*Stomatotachinasplendida* Townsend, 1931 (type species of *Stomatotachina* Townsend, 1931) is moved from its original placement in *Stomatotachina* to *Chetogena* Rondani, 1856 (with *Stomatotachina* in synonymy). *Stomatotachinasplendida* continues to be treated as a junior subjective synonym of *Parasetigenaporteri* Brèthes, 1920 (see above). **Comb. nov.**

*Sturmiafestiva* Cortés, 1944 is moved from its original placement in *Sturmia* Robineau-Desvoidy, 1830 to *Drino* Robineau-Desvoidy, 1863. Distribution: Argentina, Chile. **Comb. nov.**

*Sturmiopsoideaobscura* Thompson, 1966 (type species of *Sturmiopsoidea* Thompson, 1966) is moved from its original placement in *Sturmiopsoidea* to *Lespesia* Robineau-Desvoidy, 1863 (with *Sturmiopsoidea* in synonymy). **Comb. nov.** The name *S.obscura* is a junior secondary homonym of *Eurigasterobscurus* Bigot, 1857 when placed in *Lespesia* and is renamed herein as *Lespesiathompsoni* O’Hara & Wood, **nom. nov.** Distribution: Trinidad.

*Trichopodaarcuata* Bigot, 1876 is returned to *Trichopoda* Berthold, 1827 (and assigned to subgenus Galactomyia Townsend, 1908) from its placement in *Ectophasiopsis* Townsend, 1915 by previous authors (e.g., Aldrich 1934: 12; Guimarães 1971: 12; Dios and Nihei 2017: 5). Distribution: Argentina, Chile. **Comb. revived.**

*Trichopodagradata* Wiedemann, 1830 is returned to *Trichopoda* Berthold, 1827 (and assigned to subgenus Galactomyia Townsend, 1908) from its placement in *Ectophasiopsis* Townsend, 1915 by Dios and Nihei (2017: 10). Distribution: Argentina, Brazil, Uruguay. **Comb. revived.**

#### New and revived synonymies

New and revived generic and specific synonymies are proposed for the names below. As with the new and revived combinations listed above, they result from the study of type material, authoritatively identified specimens, and/or descriptions and figures in the literature, mostly by DMW.

*Camposodes* Cortés, 1967, formerly treated as a genus (e.g., Guimarães 1971: 166; Cortés 1984: 378), is synonymised with *Phytomyptera* Rondani, 1845. **Syn. nov.**

*Ectophasiopsis* Townsend, 1915, formerly treated as a genus (e.g., Aldrich 1934: 11; Guimarães 1971: 12; Dios and Nihei 2017: 4), is synonymised with Trichopoda Berthold, 1827, subgenus Galactomyia Townsend, 1908. **Syn. nov.**

*Embiomyia* Aldrich, 1934, formerly treated as a genus (e.g., Cortés and Hichins 1969: 32; Guimarães 1971: 85), is synonymised with *Steleoneura* Stein, 1924. **Syn. nov.**

*Fabriciaandicola* Bigot, 1888, treated as a junior synonym of *Peleteriafilipalpis* (Rondani, 1863) by Guimarães (1971: 44), is returned to synonymy with *Peleteriarobusta* (Wiedemann, 1830) as proposed earlier by Guimarães (1962: 484). **Syn. revived.**

*Macropatelloa* Townsend, 1931, formerly treated as a genus (e.g., Cortés 1986: 144, 158), González (1992b: 179), is synonymised with *Patelloa* Townsend, 1916. **Syn. nov.**

*Peleteriainca* Curran, 1925, treated as a junior synonym of *Peleteriafilipalpis* (Rondani, 1863) by Guimarães (1971: 44), is returned to synonymy with *Peleteriarobusta* (Wiedemann, 1830) as proposed earlier by Guimarães (1962: 484). **Syn. revived.**

*Poliops* Aldrich, 1934, formerly treated as a genus (e.g., Guimarães 1971: 169; Cortés 1979: 81), is synonymised with *Admontia* Brauer & Bergenstamm, 1889. **Syn. nov.**

*Ruiziella* Cortés, 1951, formerly treated as a genus (e.g., Guimarães 1971: 45; Cortés and Campos 1974: 116), is synonymised with *Chaetoepalpus* Vimmer & Soukup, 1940. **Syn. nov.**

*Ruiziellafrontosa* Cortés, 1951, formerly treated as a valid species of *Ruiziella* (e.g., Guimarães 1971: 45), is synonymised with *Chaetoepalpuscoquilleti* Vimmer & Soukup, 1940 in the genus *Chaetoepalpus* Vimmer & Soukup, 1940. **Comb. nov., syn. nov.**

*Sarcoprosena* Townsend, 1927, formerly treated as a genus (e.g., Guimarães 1971: 38; Cortés 1984: 381), is synonymised with *Billaea* Robineau-Desvoidy, 1830. **Syn. nov.**

*Schistostephana* Townsend, 1919, formerly treated as a genus (e.g., Guimarães 1971: 38), is synonymised with *Billaea* Robineau-Desvoidy, 1830. **Syn. nov.**

*Siphoactia* Townsend, 1927, formerly treated as a genus (e.g., Guimarães 1971: 170; Cortés 1984: 381), is synonymised with *Clausicella* Rondani, 1856. **Syn. nov.**

*Stomatotachina* Townsend, 1931, formerly treated as a genus (e.g., Guimarães 1971: 160; Mulieri et al. 2013: 169; Nihei 2015: 1), is synonymised with *Chetogena* Rondani, 1856. **Syn. nov.**

*Sturmiopsoidea* Thompson, 1966, formerly treated as a genus (e.g., Guimarães 1971: 192), is synonymised with *Lespesia* Robineau-Desvoidy, 1863. **Syn. nov.**

## Catalogue

### Subfamily DEXIINAE

#### Tribe DEXIINI

##### Genus *BILLAEA* Robineau-Desvoidy, 1830

*THERESIA* Robineau-Desvoidy, 1830: 325. Type species: *Theresiatandrec* Robineau-Desvoidy, 1830 (= *Muscarutilans* Fabricius, 1781), by monotypy [United States].

***BILLAEA*** Robineau-Desvoidy, 1830: 328. Type species: *Billaeagrisea* Robineau-Desvoidy, 1830 (= *Dexiapectinata* Meigen, 1826), by monotypy [France].

*EUTHERESIA* Townsend, 1911: 149. *Nomen nudum* (named for “Coquillett’s *Theresiaanalis*”, itself a *nomen nudum*).

*EUTHERESIA* Townsend, 1912a: 117. Type species: *Eutheresiamonohammi* Townsend, 1912, by monotypy [United States].

*PARATHERESIA* Townsend, 1915c: 65. Type species: *Paratheresiasignifera* Townsend, 1915 (= *Sarcophagaclaripalpis* van der Wulp, 1895), by original designation [Peru].

*SCHISTOSTEPHANA* Townsend, 1919b: 551. Type species: *Schistostephanaaurifrons* Townsend, 1919, by original designation [Peru]. **Syn. nov.**

*SARCOPROSENA* Townsend, 1927a: 228. Type species: *Sarcoprosenatriangulifera* Townsend, 1927 (junior secondary homonym of *Dexiatriangulifera* Zetterstedt, 1844; = *Billaeatriquetrus* O’Hara & Wood, **nom. nov.**, see below), by original designation [Peru]. **Syn. nov.**

*BATHYTHERESIA* Townsend, 1928a: 146. Type species: *Bathytheresiabassleri* Townsend, 1928 (= *Sarcophagaclaripalpis* van der Wulp, 1895), by original designation [Peru].

*PARABILLAEA* Blanchard, 1937: 44. Type species: *Parabillaearhynchophorae* Blanchard, 1937, by original designation [Argentina].

*PARABILAEA*. Incorrect subsequent spelling of *Parabillaea* Blanchard, 1937 (Guimarães 1977b: 269).

Notes: The concept of *Billaea* Robineau-Desvoidy adopted here is similar to that of *Theresia* Robineau-Desvoidy *sensu*Aldrich (1934: 106) for the Patagonian fauna and *Billaeasensu*Wood (1987: 1248) for the Nearctic fauna, but is expanded geographically to include variation within the lineage throughout the Neotropics. The restricted generic concepts of Townsend (1936b: 142), and to a lesser degree Guimarães (1971: 36, 1977b), were based on what we believe to be morphological differences within a larger clade that constitutes our concept of *Billaea*. This genus is characterised in part by a plumose arista, bare eye and parafacial, face with not more than a small carina dividing antennae, bare prosternum, haired proepisternum, and often three black vittae on the scutum resembling the common pattern seen in *Sarcophaga* Meigen, 1826 (Sarcophagidae). The species formerly assigned to *Sarcoprosena* differ from the other species in having a narrower parafacial and gena.

The relative priority of *Billaea* Robineau-Desvoidy, 1830 and *Theresia* Robineau-Desvoidy, 1830, when the two are treated as synonyms, was established by Wood (1987: 1248), as the First Reviser (Article 24.2.2 of the *Code*, ICZN 1999).

References: Coquillett (1910: 614), type species of *Theresia*; Aldrich (1934: 5, 106), in key to Patagonian genera (as *Theresia*), synonymy of *Eutheresia*, *Paratheresia* and *Schistostephana* with *Theresia*, taxonomic notes; Townsend (1936b: 142), diagnosis of adults and immatures of Theresiini and key to genera (including *Bathytheresia*, *Billaea*, *Eutheresia*, *Paratheresia*, *Sarcoprosena*, *Schistostephana* and *Theresia*); Townsend (1938: 391, 392, 397, 402, 403, 404, 405), redescriptions of *Bathytheresia*, *Billaea*, *Eutheresia*, *Paratheresia*, *Sarcoprosena*, *Schistostephana* and *Theresia*; van Emden (1949: 507), synonymy of *Bathytheresia* and *Parabillaea* with *Paratheresia*; Guimarães (1971: 37, 38), *Paratheresia*, *Schistostephana*, *Sarcoprosena* and *Theresia* recognised as valid genera; Cortés and Campos (1974: 115), *Sarcoprosena* in key to tachinid genera of Tarapacá and Antofagasta regions; Guimarães (1977b), revision of *Paratheresia*; Cortés (1984: 381), *Sarcoprosena* in key to tachinid genera of Tarapacá and Antofagasta regions; Wood (1987: 1248), synonymy of *Eutheresia*, *Paratheresia* and *Theresia* with *Billaea*; O’Hara and Wood (1998: 756, 759), review of synonymy of Wood (1987).

*aurifrons* (Townsend, 1919).—Not Chile [Peru]. **Comb. nov.**

**Figure 3.**
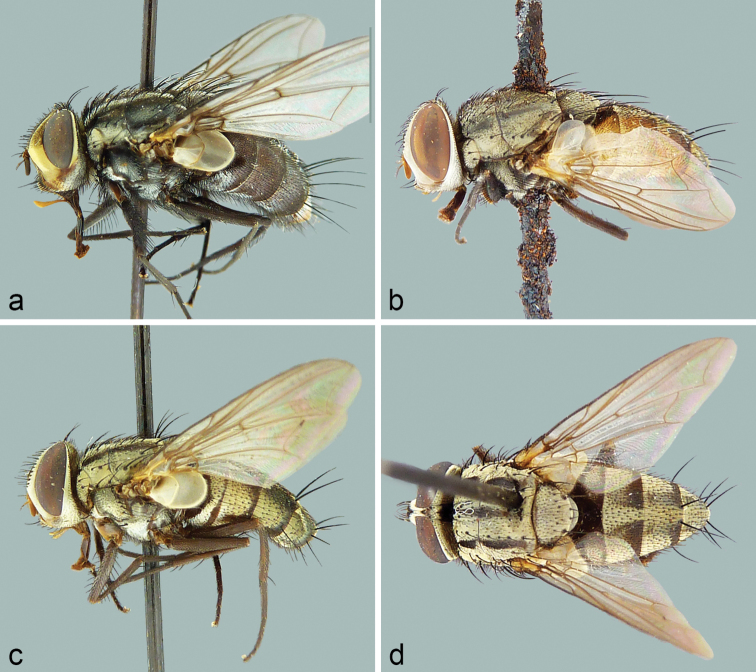
*Billaea* species (Dexiinae, Dexiini), habitus images **a***B.aurifrons* (Townsend), comb. nov. ♂ (Peru) [holotype] **b***B.rufescens* O’Hara & Wood, nom. nov., comb. nov. ♂ (Peru) [syntype] **c, d***B.triquetrus* O’Hara & Wood, nom. nov., comb. nov. ♂ (Peru) [holotype].

*Schistostephanaaurifrons* Townsend, 1919b: 552. Holotype male (USNM, examined by DMW, Fig. 3a). Type locality: Peru, Cajamarca, Río Charapi [as “Rio Charape”, ca. 5°25′S, 78°59′W], 4500 ft.

References: Aldrich (1934: 107), as species of *Theresia*; Townsend (1936b: 147, 1938: 404), as species of *Schistostephana*; Guimarães (1971: 38), as species of *Schistostephana*.

***erecta*** (Aldrich, 1934).—Neotropical: South America (Argentina, Chile).

*Theresiaerecta* Aldrich, 1934: 107. Holotype male (NHMUK). Type locality: Chile, Los Lagos, Llanquihue, Peulla.

References: Guimarães (1971: 37), as sole species of *Theresia* in America south of United States; Gramajo (1998: 93), first record from Argentina.

***luteola*** (Cortés & Campos, 1974).—Neotropical: South America (Chile). **Comb. nov.**

*Sarcoprosenaluteola* Cortés & Campos, 1974: 122. Holotype female (MEUC). Type locality: Chile, Arica y Parinacota, Arica, Valle de Lluta, km 41/42, Mollepampa [ca. 18°24′S, 70°2′W].

*rufescens* O’Hara & Wood, **nom. nov.**—Not Chile [Peru].

*Sarcoprosenarufiventris* Townsend, 1929: 367 (junior secondary homonym of *Paratheresiarufiventris* Townsend, 1929, by First Reviser action below). Syntypes, 1 male and 1 female (USNM, examined by DMW, Fig. 3b). Type locality: Peru, Río Ushpayacu, 1300 ft. **Comb. nov.**

*Billaearufescens* O’Hara & Wood, **nom. nov.** for *Sarcoprosenarufiventris* Townsend, 1929.

Note: *Paratheresiarufiventris* Townsend, 1929 and *Sarcoprosenarufiventris* Townsend, 1929, both from Peru, were described in the same publication on the same page (Townsend 1929: 367) and are secondary homonyms when placed together in *Billaea*. As the First Reviser (Article 24.2.2 of the *Code*, ICZN 1999), we hereby fix *Paratheresiarufiventris* as the senior homonym. We propose the new name *Billaearufescens* to replace the name of the junior homonym *Sarcoprosenarufiventris*. The same type material applies to the new name. The specific epithet *rufescens* is formed in part from *rufus*, Latin for red, alluding to the underlying reddish tinge of the abdomen that likely suggested the original name *rufiventris*.

Reference: Guimarães (1971: 37), as *Sarcoprosenarufiventris*.

*triquetrus* O’Hara & Wood, **nom. nov.**—Not Chile [Peru].

*Sarcoprosenatriangulifera* Townsend, 1927a: 356 (junior secondary homonym of *Dexiatriangulifera* Zetterstedt, 1844). Holotype male (USNM, examined by DMW, Fig. 3c, d). Type locality: Peru, Arequipa, Yahuarmayo. **Comb. nov.**

*Billaeatriquetrus* O’Hara & Wood, **nom. nov.** for *Sarcoprosenatriangulifera* Townsend, 1927.

Note: *Sarcoprosenatriangulifera* Townsend, 1927, when moved to *Billaea*, is a junior secondary homonym of *Dexiatriangulifera* Zetterstedt, 1844, the valid name of a *Billaea* species in the Palaearctic Region (O’Hara et al. 2009: 28). We hereby propose the new name *Billaeatriquetrus* to replace the name of the junior homonym *Sarcoprosenatriangulifera*. The same type material applies to the new name. The specific epithet *triquetrus* is Latin for three-cornered or triangular, referring to the triangular markings on the abdomen and particularly tergite 3, which presumably inspired Townsend’s name *triangulifera*.

Reference: Guimarães (1971: 37), as *Sarcoprosenatriangulifera*.

##### Genus *CALLOTROXIS* Aldrich, 1929

***CALLOTROXIS*** Aldrich, 1929b: 7. Type species: *Callotroxisedwardsi* Aldrich, 1929, by original designation [Chile].

References: Aldrich (1934: 4, 80), in key to Patagonian genera, taxonomic notes; Townsend (1936b: 131), diagnosis of adults and immatures of Prosenini and key to genera (including *Callotroxis*); Townsend (1938: 321), redescription.

***edwardsi*** Aldrich, 1929.—Neotropical: South America (Chile).

*Callotroxisedwardsi* Aldrich, 1929b: 8. Holotype male (USNM). Type locality: Chile, Araucanía, Malleco, Angol.

Reference: Aldrich (1934: 81), redescription, first description of female.

##### Genus *DASYUROMYIA* Bigot, 1885

*DASYUROMYIA* Bigot, 1885a: 237. *Nomen nudum*.

***DASYUROMYIA*** Bigot, 1885c: liv [also 1885c: liv, *Bull. Soc. Ent. France*]. Type species: *Dasyuromyiapenicillata* Bigot, 1885 (= *Tachinainornata* Walker, 1836), by monotypy [Chile].

*SELENOMYIA* Brauer & Bergenstamm, 1891: 361 [also 1891: 57]. Type species: *Selenomyiabrevicornis* Brauer & Bergenstamm, 1891 (= *Hyadesimyiasarcophagidea* Bigot, 1888), by monotypy [Chile].

*MESEMBRIOPHYTO* Townsend, 1916e: 301. Type species: *Mesembriophytomagellana* Townsend, 1916 (= *Tachinainornata* Walker, 1836), by original designation [Chile].

References: Aldrich (1934: 6, 156), in key to Patagonian genera, synonymy of *Mesembriophyto* and *Selenomyia* with *Dasyuromyia*, redescription, key to six Patagonian species; Townsend (1936c: 13), diagnosis of adults and immatures of Macquartiini and key to genera (including *Dasyuromyia*); Townsend (1939c: 32), redescription of *Dasyuromyia*; Dugdale (1969: 624), figure of terminal segments of first instar larva of *Dasyuromyia* sp.; Cortés (1986: 143), in key to tachinid genera of Aysén and Magallanes regions; Gramajo (2011: 175), key to species of Patagonian Argentina including all species listed below except for *Dasyuromyianervosa* (known only from Chile).

***aperta*** Aldrich, 1934.—Neotropical: South America (Argentina, Chile).

*Dasyuromyiaaperta* Aldrich, 1934: 161. Holotype male (USNM). Type locality: Argentina, Río Negro, Lago Gutiérrez.

Reference: Cortés and Hichins (1969: 29), first record from Chile.

***inornata*** (Walker, 1836).—Neotropical: South America (Argentina, Chile).

*Tachinainornata* Walker, 1836: 349. Lectotype male (NHMUK), by fixation of Austen (1907: 329) (examination of male “type” from Cape Gregory in NHMUK is regarded as a lectotype fixation). Type locality: Chile, Magallanes y de la Antártica Chilena, Magallanes, Cabo San Gregorio [as “Cape Gregory”, ca. 52°39′S, 70°13′W].

*Dasyuromyiapenicillata* Bigot, 1885c: lv [also 1885c: lv, *Bull. Soc. Ent. France*]. Lectotype male (NHMUK), by fixation of Townsend (1931a: 93) (examination of “male Ht” from Chile in NHMUK [as “Newmarket”] is regarded as a lectotype fixation). Type locality: Chile.

*Mesembriophytomagellana* Townsend, 1916e: 301. Holotype female (USNM). Type locality: Chile, Magallanes y de la Antártica Chilena, Magallanes, Punta Arenas [as “Sandy Point”].

References: Aldrich (1934: 157), synonymy, redescription, figures of male abdomen, first record from Argentina (as “Southern Patagonia”, which is interpreted here as Argentina based on the travels of the collector of the specimen, paleontologist Barnum Brown); Cortés (1963: 244), notes on name-bearing type of *Tachinainornata* in NHMUK.

***nervosa*** (Walker, 1836).—Neotropical: South America (Chile).

*Tachinanervosa* Walker, 1836: 349. Lectotype male (NHMUK), by fixation of Austen (1907: 329) (examination of male “type” from Port Famine in NHMUK is regarded as a lectotype fixation). Type locality: Chile, Magallanes y de la Antártica Chilena, Magallanes, Puerto del Hambre [as “Port Famine”].

Reference: Cortés (1963: 245), notes on name-bearing type in NHMUK.

***nigriceps*** Aldrich, 1934.—Neotropical: South America (Argentina, Chile).

*Dasyuromyianigriceps* Aldrich, 1934: 162. Holotype female (NHMUK). Type locality: Chile, Los Lagos, Llanquihue, Casa Pangue.

References: Cortés (1963: 244), notes on holotype in NHMUK; Gramajo (2011: 174), first record from Argentina.

***sarcophagidea*** (Bigot, 1888).—Neotropical: South America (Argentina, Chile).

*Hyadesimyiasarcophagidea* Bigot, 1888a: 28. Syntypes, 6 males and females (MNHN, see note). Type locality: Chile, Magallanes y de la Antártica Chilena, Antártica Chilena, Isla Hoste, Bahía Orange area [ca. 55°31′S, 68°6′W].

*Selenomyiabrevicornis* Brauer & Bergenstamm, 1891: 361 [also 1891: 57] (as “*S.brevicornis* Phil.”). Lectotype male (NHMW), by fixation of Townsend (1939c: 32) (mention of “Male Ht” from Chile in NHMW is regarded as a lectotype fixation). Type locality: Chile.

Notes: Bigot (1888a: 28) described *Hyadesimyiasarcophagidea* from six specimens of both sexes from the Bahía Orange area of Magallanes, Chile. The MNHN database records ten possible syntypes in the Macquart collection with numbers MNHN-ED-ED10217 to MNHN-ED-ED10226.

Brauer and Bergenstamm (1891: 361) described *Selenomyiabrevicornis* from one or more males collected from Chile by Philippi. Aldrich (1925: 459) partially redescribed one male borrowed from NHMW and labelled “Philippi, Chili, 1870”. This specimen is undoubtedly a name-bearing type of *S.brevicornis* and is assumed to be the “Ht male” of Townsend (1939c: 32).

Reference: Aldrich (1934: 159), synonymy, redescription, first record from Argentina.

***sternalis*** Aldrich, 1934.—Neotropical: South America (Argentina, Chile).

*Dasyuromyiasternalis* Aldrich, 1934: 160. Holotype male (USNM). Type locality: Chile, Araucanía, Malleco, Angol.

Reference: Cortés (1979: 79), first record from Argentina.

***tarsalis*** Aldrich, 1934.—Neotropical: South America (Argentina, Chile).

*Dasyuromyiatarsalis* Aldrich, 1934: 160. Holotype male (NHMUK). Type locality: Argentina, Río Negro, Lago Gutiérrez.

References: Cortés (1963: 245), notes on holotype in NHMUK; Cortés (1986: 146), first record from Chile.

##### Genus *HYADESIMYIA* Bigot, 1888

***HYADESIMYIA*** Bigot, 1888a: 26. Type species: *Hyadesimyiaclausa* Bigot, 1888, by subsequent designation of Bigot (1891: cxxxvi) [Chile].

References: Coquillett (1910: 553), type species (given as “*Hyadesimyiaclausa* Bigot, the first species, by present designation”); Aldrich (1934: 4, 84), in key to Patagonian genera, taxonomic notes; Townsend (1936b: 112), diagnosis of adults and immatures of Aulacephalini and key to genera (including *Hyadesimyia*); Townsend (1938: 259), redescription; Cortés (1986: 143), in key to tachinid genera of Aysén and Magallanes regions.

***clausa*** Bigot, 1888.—Neotropical: South America (Argentina, Chile).

*Hyadesimyiaclausa* Bigot, 1888a: 27. Holotype male (MNHN, number MNHN-ED-ED10216). Type locality: Chile, Magallanes y de la Antártica Chilena, Antártica Chilena, Isla Hoste, Bahía Orange area [ca. 55°31′S, 68°6′W].

References: Aldrich (1934: 85), redescription, first record from Argentina; Cortés (1973a: 101), partial redescription, head and wing figures.

##### Genus *HYOSOMA* Aldrich, 1934

***HYOSOMA*** Aldrich, 1934: 139. Type species: *Hyosomalimbisquama* Aldrich, 1934, by original designation [Argentina].

References: Townsend (1936c: 13), diagnosis of adults and immatures of Macquartiini and key to genera (including *Hyosoma*); Townsend (1939c: 41), redescription; Cortés (1986: 143), in key to tachinid genera of Aysén and Magallanes regions.

***limbisquama*** Aldrich, 1934.—Neotropical: South America (Argentina, Chile).

*Hyosomalimbisquama* Aldrich, 1934: 140. Holotype male (NHMUK). Type locality: Argentina, Río Negro, Lago Nahuel Huapí, Puerto Blest.

Note: *Hyosomalimbisquama* was recorded from both Argentina and Chile in the original description.

Reference: Cortés (1963: 244), notes on type series in NHMUK.

##### Genus *MORPHODEXIA* Townsend, 1931

***MORPHODEXIA*** Townsend, 1931c: 342. Type species: *Morphodexiamicrophthalmoides* Townsend, 1931 (= *Camaronabarrosi* Brèthes, 1920), by original designation [Chile].

References: Aldrich (1934: 6, 146), in key to Patagonian genera, redescription, key to five Patagonian species; Townsend (1936b: 116), diagnosis of adults and immatures of Dexillini and key to genera (including *Morphodexia*); Townsend (1938: 288), redescription; Cortés (1986: 142), in key to tachinid genera of Aysén and Magallanes regions.

***barrosi*** (Brèthes, 1920).—Neotropical: South America (Argentina, Chile).

*Camaronabarrosi* Brèthes, 1920a: 42. Lectotype male (MACN), by fixation of Cortés (1963: 250–251) (examination of male “tipo” from Río Blanco in MACN is regarded as a lectotype fixation). Type locality: Chile, Los Andes, Río Blanco.

*Morphodexiamicrophthalmoides* Townsend, 1931c: 343. Holotype female (USNM). Type locality: Chile, Valparaíso, Marga Marga, Bosque Los Perales [as “Perales”, ca. 33°9′S, 71°18′W].

References: Aldrich (1934: 147), redescription, head figure (as *Morphodexiamicrophthalmoides*); Verbeke (1962: 97), description of male terminalia; Cortés (1963: 250), notes and partial redescription of name-bearing type of *Camaronabarrosi* in MACN; Cortés (1967b: 13), synonymy of *Morphodexiamicrophthalmoides* with *Camaronabarrosi*; Cortés (1979: 80), first record from Argentina; Mulieri et al. (2013: 160), notes on name-bearing type (as syntype) of *C.barrosi* in MACN.

***clausa*** Aldrich, 1934.—Neotropical: South America (Argentina, Chile).

*Morphodexiaclausa* Aldrich, 1934: 149. Holotype male (USNM). Type locality: Argentina, Río Negro, Lago Nahuel Huapí, Puerto Blest.

Reference: Cortés and Hichins (1969: 43), first record from Chile.

***facialis*** (Aldrich, 1928).—Neotropical: South America (Argentina, Chile).

*Selenomyiafacialis* Aldrich, 1928b: 23. Holotype female (USNM). Type locality: Chile, Valparaíso, Marga Marga, Bosque Los Perales [as “Perales”, ca. 33°9′S, 71°18′W].

References: Aldrich (1934: 150), redescription, first description of male; Gramajo (1998: 93), first record from Argentina.

***nigra*** Aldrich, 1934.—Neotropical: South America (Argentina, Chile).

*Morphodexianigra* Aldrich, 1934: 149. Holotype male (NHMUK). Type locality: Chile, Los Lagos, Chiloé, Ancud.

Reference: Cortés (1979: 80), first record from Argentina.

***palpalis*** Aldrich, 1934.—Neotropical: South America (Chile).

*Morphodexiapalpalis* Aldrich, 1934: 150. Holotype male (USNM). Type locality: Chile, Araucanía, Malleco, Angol.

***subaenea*** Aldrich, 1934.—Neotropical: South America (Argentina, Chile).

*Morphodexianigrasubaenea* Aldrich, 1934: 149. Holotype male (USNM). Type locality: Argentina, Río Negro, Lago Gutiérrez.

References: Cortés and Hichins (1969: 44), first record from Chile (as Morphodexianigravar.subaenea); Guimarães (1971: 24), raised from subspecies to *Morphodexiasubaenea*.

##### Genus *MYIODEXIA* Cortés & Campos, 1971

***MYIODEXIA*** Cortés & Campos, 1971: 36. Type species: *Myiodexiadeserticola* Cortés & Campos, 1971, by original designation [Chile].

References: Cortés and Campos (1971: 21, 1974: 112) and Cortés (1984: 378), in keys to tachinid genera of Tarapacá and Antofagasta regions.

***deserticola*** Cortés & Campos, 1971.—Neotropical: South America (Chile).

*Myiodexiadeserticola* Cortés & Campos, 1971: 38. Holotype male (EEAM). Type locality: Chile, Tarapacá, Tamarugal, 15 km south of Pozo Almonte, Junoy, 1200 m (20°18′S, 69°48′W) (coordinates and elevation given on p. 11).

##### Genus *NOTODYTES* Aldrich, 1934

***NOTODYTES*** Aldrich, 1934: 163. Type species: *Notodytesvariabilis* Aldrich, 1934, by original designation [Argentina].

References: Aldrich (1934: 163), key to species; Townsend (1936c: 13), diagnosis of adults and immatures of Macquartiini and key to genera (including *Notodytes*); Townsend (1939c: 51), redescription; Cortés (1986: 143), in key to tachinid genera of Aysén and Magallanes regions.

***aurea*** Aldrich, 1934.—Neotropical: South America (Argentina, Chile).

*Notodytesaurea* Aldrich, 1934: 165. Holotype female (USNM). Type locality: Chile, Los Lagos, Llanquihue, Ensenada.

References: Cortés (1973a: 100), taxonomic notes; Cortés (1979: 80), first record from Argentina.

***major*** Aldrich, 1934.—Neotropical: South America (Argentina, Chile).

*Notodytesmajor* Aldrich, 1934: 165. Syntypes, 14 females (NHMUK, USNM). Type locality: Chile, Araucanía, Malleco, Angol.

References: Cortés (1963: 245), notes on two syntypes in NHMUK; Cortés (1979: 80), first record from Argentina.

***variabilis*** Aldrich, 1934.—Neotropical: South America (Argentina, Chile).

*Notodytesvariabilis* Aldrich, 1934: 164. Holotype male (NHMUK). Type locality: Argentina, Río Negro, San Carlos de Bariloche [as “Bariloche”].

Note: *Notodytesvariabilis* was recorded from both Argentina and Chile in the original description.

References: Cortés (1963: 245), notes on type series in NHMUK; Dugdale (1969: 624), figure of first instar larva; Cortés (1973a: 100), taxonomic notes.

##### Genus *OLIGOOESTRUS* Townsend, 1932

***OLIGOOESTRUS*** Townsend, 1932c: 1. Type species: *Oligooestrusoestroideus* Townsend, 1932, by original designation [Argentina].

*OLIGOESTRUS*. Incorrect subsequent spelling of *Oligooestrus* Townsend, 1932 (Guimarães 1971: 216, 302).

**Figure 4.**
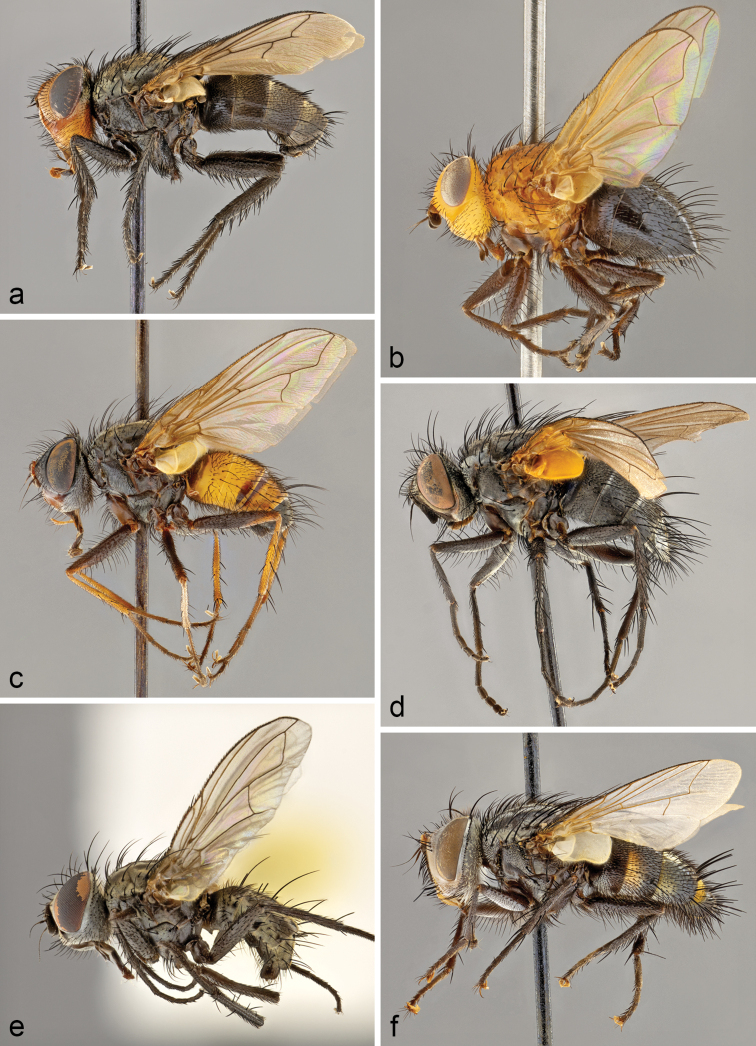
Habitus images **a***Oligooestrusoestroideus* Townsend ♂ (Dexiinae, Dexiini) (Chile) [CNC487480], 6.0 mm **b***Gonzalezodoriagonioides* Cortés ♀ (Dexiinae, Dufouriini) (Chile) [CNC1546958], 4.9 mm **c***Xanthobasisrufescens* (Blanchard) ♂ (Dexiinae, Eutrixini) (Chile) [CNC1142102], 7.0 mm **d***Admontiacalyptrata* (Aldrich) ♀ (Exoristinae, Blondeliini) (Chile) [CNC1143224], 7.5 mm **e***Steleoneuraaustralis* (Aldrich) ♀ (Exoristinae, Blondeliini) (Chile) [CNC487608], 2.5 mm **f***Chetogenahichinsi* (Cortés) ♂ (Exoristinae, Exoristini) (Chile) [CNC1546959], 9.1 mm.

Note: *Oligooestrusoestroideus*, the single known species of *Oligooestrus*, is an unusual-looking tachinid with a round yellow head in frontal view, thorax and abdomen dark, and body length of ca. 5 mm. It has a suite of distinctive features (see descriptions and figures in Townsend 1932: 2 and Aldrich 1934: 6) including tiny antenna, arista micropubescent, vibrissae closely approximated and very high (closer to antenna than to oral cavity), vein M_1_ petiolate and ending in wing margin far from wing tip, and one katepisternal seta. Townsend (1932: 1) regarded the species as “the most important oestromuscoid discovery of the twentieth century from the taxonomic point of view”, a form he believed supported his view that “the oestriform tachinids or Tachino-Oestridae of Villeneuve (Aulacephalini, Ormiini, Trixodini, Trixini, Palpostomatini, Paratrixini, Glaurocarini, and Myiotrixini) all belong in the same family with the Oestrini”. Townsend assigned *Oligooestrus* to a narrowly interpreted Oestrini within the Oestridae (see also Townsend 1936b: 108). *Oligooestrus* was later listed as an unplaced genus of Tachinidae in Guimarães (1971: 216). It was recorded from Chile for the first time in Stireman et al. (2016: 30) as “Dexiinae … ?Dufouriini: Oligooestrus?oestroideus Townsend”. The identity of that specimen as *O.oestroideus* has since been confirmed and a second specimen of *O.oestroideus* collected on the same trip and identified later bears the following data: Chile, Araucanía Region, road R-955, south of Punta Negra, 1090 m, 38°34.96′S, 71°26.35′W, 15.xii.2015, J.E. O’Hara (CNC487480)]. The recent molecular phylogeny of Tachinidae places *Oligooestrus* in the tribe Dexiini of the Dexiinae (Stireman et al. 2019: 9 [fig. 4], 30) and we follow this placement.

References: Aldrich (1934: 2, 6), in key to Patagonian genera, taxonomic notes; Townsend (1936b: 108), diagnosis of adults and immatures of Oestrini and key to genera (including *Oligooestrus*); Townsend (1938: 252), redescription; Guimarães (1971: 216), listed as an unplaced genus of Tachinidae.

***oestroideus*** Townsend, 1932.—Neotropical: South America (Argentina, Chile). **New record from Chile.** (Fig. 4a)

*Oligooestrusoestroideus* Townsend, 1932c: 4. Holotype male (USNM). Type locality: Argentina, Río Negro, Lago Nahuel Huapí, San Carlos de Bariloche [as “Bariloche”].

Note: *Oligooestrusoestroideus* was tentatively recorded from Chile (Parque Nacional Nahuelbuta, Araucanía) in a trip report by Stireman et al. (2016: 30). It is here confirmed from Chile based on a male specimen in CNC collected by JEOH during the same trip but identified later. The specimen bears the following data: Araucanía Region, road R-955, south of Punta Negra, 1090 m, 38°34.96′S, 71°26.35′W, 15.xii.2015, J.E. O’Hara (CNC487480).

Reference: Aldrich (1934: 7), redescription, taxonomic notes, head and wing figures.

##### Genus *PELYCOPS* Aldrich, 1934

***PELYCOPS*** Aldrich, 1934: 168. Type species: *Pelycopsdarwini* Aldrich, 1934, by original designation [Chile].

References: Townsend (1936c: 13), diagnosis of adults and immatures of Macquartiini and key to genera (including *Pelycops*); Townsend (1939c: 56), redescription; Cortés (1986: 143), in key to tachinid genera of Aysén and Magallanes regions.

***darwini*** Aldrich, 1934.—Neotropical: South America (Argentina, Chile).

*Pelycopsdarwini* Aldrich, 1934: 169. Holotype female (NHMUK). Type locality: Chile, Magallanes y de la Antártica Chilena, Magallanes, Puerto del Hambre [as “Port Famine”].

Notes: *Pelycopsdarwini* was recorded from both Argentina and Chile in the original description. There is variation in the amount of orange setation on the abdomen and there could be more than one species.

References: Verbeke (1962: 98, pl. V fig. 6), description and figure of male terminalia; Cortés (1963: 245), notes on holotype in NHMUK; Cortés (1986: 147), taxonomic notes; Stireman et al. (2016: 37), habitus images of P.“nr.darwini”.

##### Genus *PIRIONIMYIA* Townsend, 1931

***PIRIONIMYIA*** Townsend, 1931c: 343. Type species: *Pirionimyiaparadoxa* Townsend, 1931, by original designation [Chile].

*PIRIONOMYIA*. Incorrect subsequent spelling of *Pirionimyia* Townsend, 1931 (Aldrich 1934: 5, 105).

References: Aldrich (1934: 5, 105), in key to Patagonian genera, taxonomic notes (as “*Pirionomyia*”); Townsend (1936b: 131), diagnosis of adults and immatures of Prosenini and key to genera (including *Pirionimyia*); Townsend (1938: 361), redescription.

***paradoxa*** Townsend, 1931.—Neotropical: South America (Chile).

*Pirionimyiaparadoxa* Townsend, 1931c: 344. Holotype female (USNM). Type locality: Chile, Valparaíso, Marga Marga, Bosque Los Perales [as “Perales”, ca. 33°9′S, 71°18′W].

Reference: Aldrich (1934: 105), redescription, head figure.

##### Genus *PSECACERA* Bigot, 1880

***PSECACERA*** Bigot, 1880: 69 [also 1880: liii]. Type species: *Psecacerachiliensis* Bigot, 1880, by monotypy [Chile].

*TRIXODOPSIS* Townsend, 1933: 527. Type species: *Trixodopsisfacialis* Townsend, 1933, by monotypy (not by original designation as cited by Evenhuis et al. 2015: 270) [Chile].

References: Aldrich (1934: 6, 151), in key to Patagonian genera, synonymy, taxonomic notes, key to six species; Townsend (1936b: 121), diagnosis of Trichoprosopini and key to genera (including *Psecacera*); Townsend (1938: 299), redescription of *Psecacera*; Cortés (1986: 141, 142), synonymy of *Trixodopsis* with *Psecacera*, in key to tachinid genera of Aysén and Magallanes regions.

***atriventris*** Aldrich, 1934.—Neotropical: South America (Chile).

*Psecaceraatriventris* Aldrich, 1934: 154. Holotype male (USNM). Type locality: Chile, Los Lagos, Llanquihue, Ensenada.

***chiliensis*** Bigot, 1880.—Neotropical: South America (Argentina, Chile).

*Psecacerachiliensis* Bigot, 1880: 70 [also 1880: liii]. Syntypes, 2 specimens of unspecified sex [2 males, examined by DMW] (NHMUK). Type locality: Chile.

*Selenomyiaplena* Aldrich, 1928b: 23. Holotype male (USNM). Type locality: Chile, Araucanía, Malleco, Angol.

*chilensis*. Incorrect subsequent spelling of *chiliensis* Bigot, 1880 (Stireman et al. 2016: 29, 30).

Note: Aldrich (1928b: 23) described *Selenomyiaplena* from two males and two females collected from four localities in Argentina and Chile. Aldrich cited a male “Type” in USNM with “Cat. No. 41383” but did not give the type locality. The holotype in USNM, examined by DMW, is from Angol in Chile.

References: Brauer (1898: 494), taxonomic notes on *Psecacerachiliensis*; Aldrich (1934: 152), synonymy, redescription, figure of male surstylus; Verbeke (1962: 96), description of male terminalia.

***facialis*** (Townsend, 1933).—Neotropical: South America (Chile).

*Trixodopsisfacialis* Townsend, 1933: 527 (named for *Psecacerachiliensis* of Townsend, 1931a, not Bigot, 1880). Holotype male (NHMW). Type locality: Chile.

*Psecacerachiliensis* of Townsend (1931a: 98), not Bigot, 1880. Misidentification (Townsend 1933: 527).

***latiforceps*** Aldrich, 1934.—Neotropical: South America (Argentina, Chile).

*Psecaceralatiforceps* Aldrich, 1934: 155. Holotype male (USNM). Type locality: Argentina, Río Negro, Lago Gutiérrez.

Reference: Henry (1987: 194), first record from Chile.

***robusta*** Aldrich, 1934.—Neotropical: South America (Argentina, Chile).

*Psecacerarobusta* Aldrich, 1934: 154. Syntypes, 2 males (NHMUK, USNM). Type locality: Chile, Biobío, Concepción, San Rosendo.

Note: Aldrich (1934: 154) described *Psecacerarobusta* from two male “cotypes” (i.e., syntypes) but did not name the type depository. There appears to be three specimens labelled as types in collections, one in NHMUK and two in USNM (all examined by DMW).

Reference: Gramajo (1998: 93), first record from Argentina.

***tibialis*** Aldrich, 1934.—Neotropical: South America (Chile).

*Psecaceratibialis* Aldrich, 1934: 154. Holotype male (USNM). Type locality: Chile, Los Lagos, Llanquihue, Casa Pangue.

***virens*** (Aldrich, 1928).—Neotropical: South America (Chile).

*Selenomyiavirens* Aldrich, 1928b: 22. Holotype male (USNM). Type locality: Chile, Valparaíso, Marga Marga, Bosque Los Perales [as “Perales, Chile, near Santiago”, ca. 33°9′S, 71°18′W].

##### Genus *SETOLESTES* Aldrich, 1934

***SETOLESTES*** Aldrich, 1934: 142. Type species: *Setolestesgenalis* Aldrich, 1934, by original designation [Chile].

References: Townsend (1936c: 13), diagnosis of adults and immatures of Macquartiini and key to genera (including *Setolestes*); Townsend (1939c: 65), redescription; Cortés (1986: 143), in key to tachinid genera of Aysén and Magallanes regions.

***genalis*** Aldrich, 1934.—Neotropical: South America (Chile).

*Setolestesgenalis* Aldrich, 1934: 142. Holotype male (NHMUK). Type locality: Chile, Los Lagos, Chiloé, Castro.

References: Cortés (1963: 244), notes on holotype in NHMUK; Cortés (1973a: 100), taxonomic notes.

### Tribe DUFOURIINI

#### Genus *GONZALEZODORIA* Cortés, 1967

***GONZALEZODORIA*** Cortés, 1967b: 18. Type species: *Gonzalezodoriagonioides* Cortés, 1967, by original designation [Chile].

Notes: *Gonzalezodoriagonioides* is a small dark tachinid with a globous abdomen that Cortés (1967b: 18) noted would run to Dexiinae in the keys of Mesnil (1939), or to the Prosenidae in the family key of Townsend (1936b). In Aldrich’s (1934) key to Tachinidae of Patagonia and South Chile it runs to *Myiophasia* Brauer & Bergenstamm, 1891 (now a synonym of *Gnadochaeta* Macquart, 1851) but “con el cual no tiene ningun parecido” [“with which it has no resemblance”] according to Cortés (1967b: 20). Guimarães (1971: 26) did not agree and placed *Gonzalezodoria* in the Myiophasiini along with a dozen species of *Myiophasia* and five monotypic genera. We have examined five females of *G.gonioides* in CNC with the following data: Chile, Los Lagos, Volcán Osorno, La Picada, 600 m, 1980, L. Peña (CNC1546958, CNC1546966–CNC1546969). They clearly belong to the “Oestrophasiini” *sensu*Guimarães (1977a), differing from the genera treated therein in having setae on the parafacial that are continuous with the orbitals on the fronto-orbital plate (as shown in figs 1–2 in Cortés 1967b: 21) and lacking banding on the wing. Guimarães (1977a: 216) remarked that the male terminalia of Oestrophasiini “are very similar to those of the Old World Dufouriini … but it will be many years before the interrelationships of this difficult group … becomes clear”. The recent molecular phylogeny of Tachinidae of Stireman et al. (2019: 9 [fig. 4]) places *Oestrophasia* in the Dufouriini and *Gonzalezodoria* belongs there as well.

The male of *G.gonioides* has not been described but a single male in CNC with the following data might be the male of this species: Santiago Metro. Region, Mirador de Los Tres Valles, 1820 m, 7.xii.2015, J.E. O’Hara [CNC487604]. This male is a good match morphologically with the aforementioned females but the colouration is different. The females have a yellow head and thorax and a black abdomen; the male has a yellow head except for black underlying the continuous orbital and parafacial setae, black thorax except for yellow scutellum, and yellow abdomen except for black syntergite 1+2, median vitta and bands posteriorly on tergites 3–5.

***gonioides*** Cortés, 1967.—Neotropical: South America (Chile). (Fig. 4b)

*Gonzalezodoriagonioides* Cortés, 1967b: 19. Holotype female (EEAM). Type locality: Chile, Coquimbo, Limarí, 15 km southwest of Pachingo, near Parque Nacional Bosque Fray Jorge, 110–250 m.

### Tribe EUTRIXINI

The South American genus *Xanthobasis* appears to belong to one of the most basal lineages of the Dexiinae according to the recent molecular phylogeny of the Tachinidae of Stireman et al. (2019: 9). In the same clade is the small North American genus *Eutrixa* Coquillett, 1897. The extant tachinids of early lineages like this one are often difficult to classify morphologically and molecular evidence is helping with their phylogenetic, and hence taxonomic, placements. Historically, Guimarães (1971: 110) followed Townsend (1936c: 49, 1939c: 163) in placing *Xanthobasis* in the tribe Ebeniini, subfamily Dexiinae (“Dexiidae” *sensu* Townsend). Sabrosky and Arnaud (1965: 981) followed Townsend (1936b: 115, 1938: 258) in placing *Eutrixa* in the tribe Aulacephalini, with the former authors assigning the tribe to subfamily Proseninae (“Dexiinae of authors”) in contrast to Townsend’s placement in the family Oestridae. O’Hara and Wood (2004: 45) transferred *Eutrixa* to the Palpostomatini as the first New World member of the tribe. They were influenced by such similarities as a weak genal dilation, weakly developed postscutellum, lower calypter strongly diverging from scutellum, and shared parasitism of scarab beetles. These are traits of *Xanthobasis* as well. However, the molecular phylogenetic evidence of Stireman et al. (2019: 9) suggests that the Palpostomatini are an Old World lineage and the name Eutrixini therefore applies to *Xanthobasis* and allies (*Eutrixa*, *Isidotus* Reinhard, 1962, and genera named below in the *Xanthobasis* note).

#### Genus *XANTHOBASIS* Aldrich, 1934

***XANTHOBASIS*** Aldrich, 1934: 110. Type species: *Xanthobasisangustifrons* Aldrich, 1934, by original designation [Argentina].

*PROXANTHOBASIS* Blanchard, 1966b: 219. Type species: *Proxanthobasisrufipes* Blanchard, 1966, by original designation [Argentina].

Note: Cortés (1973a: 101) synonymised *Proxanthobasis* with *Xanthobasis* and Cortés (1986: 147) later commented that two additional monotypic genera described from Argentina, *Neoxanthobasis* Blanchard, 1966 and *Paraxanthobasis* Blanchard, 1966, may also be synonyms. We did not examine specimens of these last two nominal genera and cannot comment on the merits of this proposition but we do include all three nominal genera in the Eutrixini based on their presumed close relationship.

References: Aldrich (1934: 110), key to three Patagonian species; Townsend (1936c: 47), diagnosis of Ebeniini and key to genera (including *Xanthobasis*); Townsend (1939c: 163), redescription of *Xanthobasis*; Cortés (1973a: 101), synonymy of *Proxanthobasis* with *Xanthobasis*; Cortés (1986: 143, 147), in key to tachinid genera of Aysén and Magallanes regions, comments on likely additional generic synonymy.

***angustifrons*** Aldrich, 1934.—Neotropical: South America (Argentina, Chile).

*Xanthobasisangustifrons* Aldrich, 1934: 111. Holotype male (USNM). Type locality: Argentina, Río Negro, Lago Gutiérrez.

Note: *Xanthobasisangustifrons* was recorded from both Argentina and Chile in the original description.

References: Blanchard (1966b: 225), taxonomic notes; Cortés (1973a: 101), taxonomic notes.

***rufescens*** (Blanchard, 1966).—Neotropical: South America (Argentina, Chile). (Fig. 4c)

*Proxanthobasisrufescens* Blanchard, 1966b: 222. Holotype male (not located). Type locality: Argentina, Río Negro, San Carlos de Bariloche [as “Bariloche”].

Reference: Cortés (1973a: 101), moved to *Xanthobasis*, first record from Chile.

***unicolor*** Aldrich, 1934.—Neotropical: South America (Argentina, Chile). **New record from Chile.**

*Xanthobasisunicolor* Aldrich, 1934: 112. Holotype male (NHMUK). Type locality: Argentina, Río Negro, Lago Gutiérrez.

Note: *Xanthobasisunicolor* is recorded from Chile for the first time based on material from Rofuco [in Los Ríos Region, Valdivia Province] in MZSP identified by R. Cortés (ex. unpublished notes of Cortés in UMCE examined by CRG).

References: Blanchard (1966b: 218), redescription and wing figure, assigned to *Paraxanthobasis* Blanchard, 1966; Guimarães (1971: 110), as *Paraxanthobasisunicolor*; Cortés (1986: 147), as *Xanthobasisunicolor*.

### Tribe VORIINI

#### Genus *ACTINOPLAGIA* Blanchard, 1940

***ACTINOPLAGIA*** Blanchard, 1940: 234. Type species: *Actinoplagiakoehleri* Blanchard, 1940, by original designation [Argentina].

Reference: Cortés (1967b: 12), key to separate *Actinoplagia* Blanchard and *Chaetodemoticus* Brauer & Bergenstamm.

***koehleri*** Blanchard, 1940.—Neotropical: South America (Argentina, Chile, Uruguay).

*Actinoplagiakoehleri* Blanchard, 1940: 234. Holotype male (MACN). Type locality: Argentina, Buenos Aires, Arrecifes.

References: Parker et al. (1951 [pages unknown], also 1953: 53, 66), first record from Uruguay; Parker (1953: 62), figures of first instar larva and puparium; Blanchard (1963: 170), redescription, head figures; Cortés (1967b: 12), first record from Chile; Mulieri et al. (2013: 165), notes on holotype in MACN.

#### Genus *ALDRICHIOPA* Guimarães, 1971

*APHELOGASTER* Aldrich, 1934: 22 (junior homonym of *Aphelogaster* Kolbe, 1897). Type species: *Aphelogastercoracella* Aldrich, 1934, by original designation [Argentina].

***ALDRICHIOPA*** Guimarães, 1971: 165 (*nomen novum* for *Aphelogaster* Aldrich, 1934).

References: Townsend (1936c: 129), diagnosis of adults and immatures of Actiini and key to genera (including *Aphelogaster*); Townsend (1940a: 192), redescription of *Aphelogaster*.

***coracella*** (Aldrich, 1934).—Neotropical: South America (Argentina, Chile).

*Aphelogastercoracella* Aldrich, 1934: 23. Holotype male (NHMUK). Type locality: Argentina, Río Negro, Lago Gutiérrez.

Reference: Townsend (1940a: 192), first record from Chile.

#### Genus *ALEXOGLOBLINIA* Cortés, 1945

***ALEXOGLOBLINIA*** Cortés, 1945b: 256. Type species: *Metopomuscopteryxshannoni* Aldrich, 1934, by original designation [Argentina].

***shannoni*** (Aldrich, 1934).—Neotropical: South America (Argentina, Chile).

*Metopomuscopteryxshannoni* Aldrich, 1934: 46. Holotype male (USNM). Type locality: Argentina, Río Negro, Lago Nahuel Huapí.

Reference: Stireman et al. (2016: 34), first record from Chile (Araucanía Region, Parque Nacional Conguillío, Laguna Conguillío), single female in Stireman collection.

#### Genus *ALPINOPLAGIA* Townsend, 1931

***ALPINOPLAGIA*** Townsend, 1931d: 475. Type species: *Alpinoplagiaboliviana* Townsend, 1931, by original designation [Bolivia].

References: Townsend (1936b: 232), diagnosis of adults and immatures of Voriini and key to genera (including *Alpinoplagia*); Townsend (1939a: 373), redescription; Cortés and Campos (1971: 23, 1974: 114) and Cortés (1984: 380), in keys to tachinid genera of Tarapacá and Antofagasta regions; Cortés and González (1989: 116), in key to genera of Chilean Voriini.

***boliviana*** Townsend, 1931.—Neotropical: South America (Bolivia, Chile).

*Alpinoplagiaboliviana* Townsend, 1931d: 476. Holotype female (NHMW). Type locality: Bolivia, La Paz, Cerro Sillutincara [as “Cuesta de Cillutincara”, ca. 16°17′S, 67°53′W], 11,000 ft.

Reference: Cortés and Campos (1971: 46), first record from Chile, redescription, head figure.

#### Genus *ATELOGLUTUS* Aldrich, 1934

References: Cortés and Valencia (1972: 66), key to the two subgenera of *Ateloglutus* and the three species of new subgenus Proteloglutus; Cortés (1984: 378), in key to tachinid genera of Tarapacá and Antofagasta regions; Cortés (1986: 143), in key to tachinid genera of Aysén and Magallanes regions; González (1989), review, key to Chilean species; Cortés and González (1989: 116), in key to genera of Chilean Voriini.

### Subgenus ATELOGLUTUS Aldrich, 1934

***ATELOGLUTUS*** Aldrich, 1934: 24. Type species: *Ateloglutusruficornis* Aldrich, 1934, by original designation [Argentina].

References: Townsend (1936c: 129), diagnosis of adults and immatures of Actiini and key to genera (including *Ateloglutus*); Townsend (1940a: 192), redescription of *Ateloglutus*.

***blanchardi*** Cortés, 1979.—Neotropical: South America (Argentina, Chile).

Ateloglutus (Ateloglutus) blanchardi Cortés, 1979: 77. Holotype female (MLPA). Type locality: Argentina, Santa Cruz, Caleta Olivia, 5 km northwest of Piedrabuena, 130 m.

Reference: González (1989: 226, 227), first description of male, head figure, first record from Chile.

***lanfrancoi*** Cortés, 1986.—Neotropical: South America (Chile).

Ateloglutus (Ateloglutus) lanfrancoi Cortés, 1986: 147. Holotype male (MEUC). Type locality: Chile, Magallanes y de la Antártica Chilena, Última Esperanza, Sierra de Los Baguales, 600 m [ca. 50°47′S, 72°24′W].

***ruficornis*** Aldrich, 1934.—Neotropical: South America (Argentina, Chile).

*Ateloglutusruficornis* Aldrich, 1934: 25. Holotype male (NHMUK). Type locality: Argentina, Río Negro, Lago Nahuel Huapí.

Note: *Ateloglutusruficornis* was recorded from both Argentina and Chile in the original description.

### Subgenus PROTELOGLUTUS Cortés & Valencia, 1972

*PROTELOGLUTUS* Cortés & Valencia, 1972: 66. Type species: *Phorichaetachilensis* Brèthes, 1920, by original designation [Chile].

***chilensis*** (Brèthes, 1920).—Neotropical: South America (Argentina, Chile).

*Phorichaetachilensis* Brèthes, 1920a: 42. Type(s), unspecified sex (1 female in MACN, Mulieri et al. 2013: 162). Type locality: Chile, Los Andes, Río Blanco.

References: Aldrich (1934: 25), redescription, first record from Argentina; Cortés (1963: 251), notes on a female with data of name-bearing type in MACN (with no mention of “type” and hence not a lectotype fixation); Cortés and Valencia (1972: 66), in key, taxonomic notes; Mulieri et al. (2013: 162), notes on syntype in MACN.

***nitens*** Aldrich, 1934.—Neotropical: South America (Argentina, Chile).

*Ateloglutusnitens* Aldrich, 1934: 26. Holotype female (NHMUK). Type locality: Argentina, Río Negro, eastern end of Lago Nahuel Huapí.

References: Cortés and Valencia (1972: 66), in key, first record from Chile; Cortés (1979: 77), taxonomic notes.

***velardei*** Cortés & Valencia, 1972.—Neotropical: South America (Argentina, Chile, Peru).

Ateloglutus (Proteloglutus) velardei Cortés & Valencia, 1972: 67. Holotype male (EESC). Type locality: Peru, Ica, Hacienda Paraya.

Note: *Ateloglutusvelardei* was recorded from both Peru and Chile in the original description.

Reference: González (1989: 226, 227), wing figure, first record from Argentina.

#### Genus *CHAETODEMOTICUS* Brauer & Bergenstamm, 1891

***CHAETODEMOTICUS*** Brauer & Bergenstamm, 1891: 385 [also 1891: 81]. Type species: *Demoticuschilensis* Schiner, 1868, by monotypy [Chile].

References: Townsend (1936b: 218), diagnosis of adults and immatures of Germariini and key to genera (including *Chaetodemoticus*); Townsend (1939a: 319), redescription; Cortés (1967b: 12), key to separate *Chaetodemoticus* and *Actinoplagia* Blanchard; Cortés and Campos (1971: 25, 1974: 115) and Cortés (1984: 380), in keys to tachinid genera of Tarapacá and Antofagasta regions.

***chilensis*** (Schiner, 1868).—Neotropical: South America (Chile).

*Demoticuschilensis* Schiner, 1868: 324. Holotype male [not female as published, Aldrich 1927b: 5] (NHMW). Type locality: Chile.

References: Aldrich (1927b: 5), redescription of holotype; Cortés (1945c: 24), redescription; Cortés and Campos (1971: 56, 61), notes, head figure.

#### Genus *CHILOCLISTA* Townsend, 1931

***CHILOCLISTA*** Townsend, 1931c: 334. Type species: *Chiloclistabicolor* Townsend, 1931, by original designation [Chile].

References: Townsend (1936c: 13), diagnosis of adults and immatures of Macquartiini and key to genera (including *Chiloclista*); Townsend (1939c: 30), redescription.

***bicolor*** Townsend, 1931.—Neotropical: South America (Chile).

*Chiloclistabicolor* Townsend, 1931c: 334. Holotype male (USNM). Type locality: Chile, O’Higgins, Cardenal Caro, Tanumé [ca. 34°13′S, 71°55′W].

Reference: Stireman et al. (2016: 37), habitus images.

#### Genus *CORACOMYIA* Aldrich, 1934

***CORACOMYIA*** Aldrich, 1934: 21. Type species: *Coracomyiacrassicornis* Aldrich, 1934, by original designation [Argentina].

References: Townsend (1936c: 129), diagnosis of adults and immatures of Actiini and key to genera (including *Coracomyia*); Townsend (1940a: 206), redescription; Cortés (1986: 143), in key to tachinid genera of Aysén and Magallanes regions; Cortés and González (1989: 116), in key to genera of Chilean Voriini.

***crassicornis*** Aldrich, 1934.—Neotropical: South America (Argentina, Chile).

*Coracomyiacrassicornis* Aldrich, 1934: 22. Holotype male (NHMUK). Type locality: Argentina, Río Negro, Lago Nahuel Huapí.

Reference: Cortés (1976: 8), partial redescription including first description of female, first record from Chile.

***woodi*** Cortés, 1976.—Neotropical: South America (Chile).

*Coracomyiawoodi* Cortés, 1976: 8. Holotype male (MEUC). Type locality: Chile, Los Lagos, Osorno, Parque Nacional Puyehue, Paso Cardenal Antonio Samoré [as “Paso Puyehue”], 1200 m [ca. 40°42′S, 71°57′W].

#### Genus *CYRTOPHLOEBA* Rondani, 1856

***CYRTOPHLOEBA*** Rondani, 1856: 207. Type species: *Tachinaruricola* Meigen, 1824, by original designation [Europe].

*EUCYRTOPHLOEBA* Townsend, 1916e: 316. Type species: *Eucyrtophloebarhois* Townsend, 1916, by original designation [Mexico].

*OPSOPHAGUS* Aldrich, 1926a: 15. Type species: *Opsophagusornatus* Aldrich, 1926, by original designation [Peru].

*CYRTHOPHLEBA*. Incorrect subsequent spelling of *Cyrtophloeba* Rondani, 1856 (Rondani 1857: 13) (see O’Hara et al. 2011: 68).

*CYRTOPHLEBA*. Incorrect original spelling of *Cyrtophloeba* Rondani, 1856 (Rondani 1856: 68) (see O’Hara et al. 2011: 69).

References: Coquillett (1910: 530), type species of *Cyrtophloeba* (as “*Cyrtophleba*”); Aldrich (1934: 3, 32), in key to Patagonian genera, taxonomic notes (as *Opsophagus*); Townsend (1936b: 232), diagnosis of adults and immatures of Voriini and key to genera (including *Cyrtophloeba* [as “*Cyrtophleba*”], *Eucyrtophloeba* and *Opsophagus*); Townsend (1939a: 378, 379, 393), redescriptions of *Cyrtophloeba* (as “*Cyrtophleba*”), *Eucyrtophloeba* and *Opsophagus*; Caltagirone (1966: 63), key to Neotropical species (as *Opsophagus*); Cortés (1986: 143), in key to tachinid genera of Aysén and Magallanes regions (as *Opsophagus*); Cortés and González (1989: 116), in key to genera of Chilean Voriini (as *Opsophagus*); Wood and Zumbado (2010: 1402), synonymy of *Opsophagus* with *Cyrtophloeba* (as “*Cyrtophleba*”).

***cortesi*** (Caltagirone, 1966).—Neotropical: South America (Argentina, Chile).

*Opsophaguscortesi* Caltagirone, 1966: 64. Holotype male (INLA). Type locality: Chile, Maule, Talca, Gualleco.

References: Cortés (1967b: 12), taxonomic notes; Gramajo (1998: 95), first record from Argentina.

***nigripalpis*** (Aldrich, 1926).—Neotropical: South America (Argentina, Chile, Ecuador).

*Opsophagusnigripalpis* Aldrich, 1926a: 16. Holotype male (USNM). Type locality: Chile, Valparaíso, Marga Marga, Bosque Los Perales [as “Perales”, ca. 33°9′S, 71°18′W].

References: Aldrich (1934: 33), redescription, taxonomic notes, first record from Argentina; Cortés (1979: 80), taxonomic notes; Cortés (1980: 106), first record from Ecuador.

#### Genus *DISCHOTRICHIA* Cortés, 1944

***DISCHOTRICHIA*** Cortés, 1944f: 54. Type species: *Dischotrichiacaelibata* Cortés, 1944, by original designation [Chile].

Reference: Cortés (1975: 36), in key to related genera.

***caelibata*** Cortés, 1944.—Neotropical: South America (Chile).

*Dischotrichiacaelibata* Cortés, 1944f: 56. Holotype male (USNM). Type locality: Chile, Valparaíso, Marga Marga.

Reference: Campos (1953: 25), first description of female.

#### Genus *GANOPLEURON* Aldrich, 1934

***GANOPLEURON*** Aldrich, 1934: 118. Type species: *Ganopleurondivergens* Aldrich, 1934, by original designation [Chile].

***divergens*** Aldrich, 1934.—Neotropical: South America (Argentina, Chile).

*Ganopleurondivergens* Aldrich, 1934: 119. Holotype female (NHMUK). Type locality: Chile, Los Lagos, Chiloé, Castro.

References: Townsend (1936c: 13), diagnosis of adults and immatures of Macquartiini and key to genera (including *Ganopleuron*); Townsend (1939c: 39), redescription; Cortés (1963: 243), notes on holotype in NHMUK; Cortés (1973a: 98), first description of male; Gramajo (1998: 94), first record from Argentina.

#### Genus *LAFUENTEMYIA* Marnef, 1965

***LAFUENTEMYIA*** Marnef, 1965: 243. Type species: *Lafuentemyiayanezi* Marnef, 1965, by original designation [Chile].

Reference: Cortés (1975: 36), in key to genera with a modified hind femur in male.

***yanezi*** Marnef, 1965.—Neotropical: South America (Chile).

*Lafuentemyiayanezi* Marnef, 1965: 246. Holotype male (UVVC). Type locality: Chile, Valparaíso, Valparaíso, Reserva Nacional Lago Peñuelas.

Reference: Cortés (1975: 36), taxonomic notes.

#### Genus *MYIOCHAETA* Cortés, 1967

***MYIOCHAETA*** Cortés, 1967b: 24. Type species: *Myiochaetamarnefi* Cortés, 1967, by original designation [Chile].

Reference: Cortés and González (1989: 116), in key to genera of Chilean Voriini.

***marnefi*** Cortés, 1967.—Neotropical: South America (Chile).

*Myiochaetamarnefi* Cortés, 1967b: 25. Holotype male (EEAM). Type locality: Chile, Metropolitana de Santiago, Santiago, Maipú, Rinconada, Quebrada de La Plata, 510 m.

#### Genus *NEOCHAETOPLAGIA* Blanchard, 1963

***NEOCHAETOPLAGIA*** Blanchard, 1963: 173. Type species: *Neochaetoplagiapastranai* Blanchard, 1963, by original designation [Argentina].

Reference: Cortés and González (1989: 116), in key to genera of Chilean Voriini.

***pastranai*** Blanchard, 1963.—Neotropical: South America (Argentina, Chile).

*Neochaetoplagiapastranai* Blanchard, 1963: 173. Holotype male (presumed lost, Mulieri et al. 2013: 168). Type locality: Argentina, Buenos Aires, Buenos Aires [as “Capital Federal”].

References: Cortés and González (1989: 118), first record from Chile; Mulieri et al. (2013: 168), notes on type series.

#### Genus *NOTHOVORIA* Cortés & González, 1989

***NOTHOVORIA*** Cortés & González, 1989: 120. Type species: *Nothovoriapraestans* Cortés & González, 1989, by original designation [Chile].

Reference: Cortés and González (1989: 116), in key to genera of Chilean Voriini.

***praestans*** Cortés & González, 1989.—Neotropical: South America (Chile).

*Nothovoriapraestans* Cortés & González, 1989: 120. Holotype female (UMCE). Type locality: Chile, Tarapacá, Iquique, 40 km from Iquique, Pampa del Tamarugal, Estación Refresco, 1200 m.

#### Genus *PHAEODEMA* Aldrich, 1934

***PHAEODEMA*** Aldrich, 1934: 145. Type species: *Phaeodemamystacina* Aldrich, 1934, by original designation [Chile].

References: Townsend (1936c: 47), diagnosis of Ebeniini and key to genera (including *Phaeodema*); Townsend (1939c: 160), redescription; Cortés (1986: 143), in key to tachinid genera of Aysén and Magallanes regions.

***mystacina*** Aldrich, 1934.—Neotropical: South America (Argentina, Chile).

*Phaeodemamystacina* Aldrich, 1934: 145. Holotype male (NHMUK). Type locality: Chile, Los Lagos, Llanquihue, Puerto Montt.

Reference: Gramajo (1998: 94), first record from Argentina.

#### Genus *PIRIONA* Aldrich, 1928

***PIRIONA*** Aldrich, 1928b: 24. Type species: *Pirionafasciculata* Aldrich, 1928, by original designation [Chile].

References: Aldrich (1934: 3, 44), in key to Patagonian genera, taxonomic notes; Townsend (1936c: 13), diagnosis of adults and immatures of Macquartiini and key to genera (including *Piriona*); Townsend (1939c: 58), redescription; Cortés (1975: 36), in key to genera with a modified hind femur in male.

***fasciculata*** Aldrich, 1928.—Neotropical: South America (Argentina, Chile).

*Pirionafasciculata* Aldrich, 1928b: 24. Holotype male (USNM). Type locality: Chile, Valparaíso, Marga Marga.

Note: Aldrich (1928b: 25) described *Pirionafasciculata* from five males and one female collected from three localities in Argentina and Chile. Aldrich cited a male “Type” in USNM with “Cat. No. 41385” but did not give the type locality. The holotype in USNM was examined by DMW and is from Marga Marga Province in Chile (see also Aldrich 1934: 45).

References: Aldrich (1934: 45), redescription; Cortés (1963: 243), notes on specimens in NHMUK; Cortés (1975: 36), taxonomic notes.

#### Genus *PROSOPOCHAETA* Macquart, 1851

***PROSOPOCHAETA*** Macquart, 1851: 183 [also 1851: 210] (as “*Prosopochoeta*”, see note). Type species: *Prosopochaetanitidiventris* Macquart, 1851, by original designation [Chile].

*PUNACLISTA* Townsend, 1915e: 406. Type species: *Punaclistasetosa* Townsend, 1915, by original designation [Peru].

*PROSOPOCHOETA*. Incorrect original spelling of *Prosopochaeta* Macquart, 1851 (Macquart 1851: 183, see note).

Notes: The name *Prosopochaeta* Macquart, 1851 was originally published as *Prosopochoeta* but subsequent authors (e.g., Aldrich 1934; Cortés and Hichins 1969; Guimarães 1971; González 1992b) used the spelling *Prosopochaeta*. This changed spelling would normally be considered an incorrect subsequent spelling but because it is in prevailing usage and is attributed to Macquart (1851), it is deemed to be the correct original spelling (Article 33.3.1 of the *Code*, ICZN 1999).

Macquart (1851: 184 [also 1851: 211]) noted about his new genus *Prosopochaeta* (as *Prosopochoeta*), “Le type de ce genre est du Chili” [“The type of this genus is from Chile”]. This statement is accepted as a type species designation for *Prosopochaeta* of the single included species, *Prosopochaetanitidiventris* Macquart, from Chile.

References: Aldrich (1934: 5, 115), in key to Patagonian genera, synonymy of *Punaclista* with *Prosopochaeta*, redescription; Townsend (1936b: 121), diagnosis of Trichoprosopini and key to genera (including *Prosopochaeta*); Townsend (1936c: 13), diagnosis of adults and immatures of Macquartiini and key to genera (including *Punaclista*); Townsend (1938: 299), redescription of *Prosopochaeta*; Townsend (1939c: 62), redescription of *Punaclista*; Parker (1953: 66), figures of puparium of *Prosopochaeta* sp.; Cortés and Campos (1971: 45), taxonomic notes including reinstating synonymy of *Punaclista* with *Prosopochaeta*; Cortés and Campos (1971: 26, 1974: 116) and Cortés (1984: 381), in keys to tachinid genera of Tarapacá and Antofagasta regions; Cortés (1986: 142), in key to tachinid genera of Aysén and Magallanes regions.

***anomala*** Aldrich, 1934.—Neotropical: South America (Argentina, Chile).

*Prosopochaetaanomala* Aldrich, 1934: 118. Holotype male (USNM). Type locality: Argentina, Río Negro, eastern end of Lago Nahuel Huapí, Jones Estancia.

Note: *Prosopochaetaanomala* was recorded from both Argentina and Chile in the original description.

***caliginosa*** Cortés & Campos, 1971.—Neotropical: South America (Argentina, Chile).

*Prosopochaetacaliginosa* Cortés & Campos, 1971: 43. Holotype male (EEAM). Type locality: Chile, Antofagasta, Antofagasta, north of Quebrada de Paposo, 200–1000 m (25°03′S, 70°25′W) (coordinates and elevation given on p. 12).

Reference: Cortés (1979: 81), first record from Argentina.

***nitidiventris*** Macquart, 1851.—Neotropical: South America (Argentina, Chile).

*Prosopochoetanitidiventris* Macquart, 1851: 184 [also 1851: 211]. Lectotype male (MNHN), by designation herein (see Lectotype Designations section). Type locality: Chile (“Coquimbo, etc.” according to Blanchard 1854: 424).

Notes: *Punaclistasetosa* Townsend, 1915 from Peru was treated as a synonym of *Prosopochoetanitidiventris* Macquart by Aldrich (1934: 116) but is currently recognised as a valid species of *Prosopochaeta* (e.g., Cortés and Campos 1971: 46; Guimarães 1971: 100).

References: Aldrich (1934: 116), redescription, head figure, first record from Argentina; Verbeke (1962: 103), description of male terminalia.

#### Genus *TRICHODISCHIA* Bigot, 1885

*TRICHODISCHIA* Bigot, 1885a: 237. *Nomen nudum*.

***TRICHODISCHIA*** Bigot, 1885b: xlv [also 1885b: xlv, *Bull. Soc. Ent. France*]. Type species: *Trichodischiasoror* Bigot, 1885, by subsequent designation of Townsend (1916b: 9) [Argentina].

*TRICHORAEA* Cortés, 1975: 37. Type species: *Trichodischiacaerulea* Bigot, 1885, by original designation [Argentina].

*TRICODISCHIA*. Incorrect subsequent spelling of *Trichodischia* Bigot, 1885 (Henry 1987: 194).

Note: The two Bigot species *Trichodischiacaerulea* and *T.soror* were treated as generically different by Cortés (1975: 37) and the former was assigned to new genus *Trichoraea* Cortés. Subsequent authors have continued to treat the original combination *Trichodischiacaerulea* as valid (e.g., Guimarães 1977c: 76; Henry 1987: 195; Molina-Ochoa et al. 2003: 269) and this classification is followed here, but without further study.

References: Townsend (1936c: 13), diagnosis of adults and immatures of Macquartiini and key to genera (including *Trichodischia*); Townsend (1939c: 68), redescription of *Trichodischia*; Parker (1953: 66), figures of first instar larva and puparium of *Trichodischia* sp., from Argentina; Cortés (1969: 98), key to separate the two species; Cortés (1975: 36), *Trichodischia* and *Trichoraea* in key to genera with a modified hind femur in male.

***caerulea*** Bigot, 1885.—Neotropical: South America (Argentina, Chile, Uruguay).

*Trichodischiacaerulea* Bigot, 1885b: xlv [also 1885b: xlv, *Bull. Soc. Ent. France*]. Syntypes, 3 specimens as “♀?” (2 males and 1 female in NHMUK, examined by DMW). Type locality: Argentina, Buenos Aires, Buenos Aires.

*caerulai*. Incorrect subsequent spelling of *caerulea* Bigot, 1885 (Blanchard 1963: 184).

*caerulia*. Incorrect subsequent spelling of *caerulea* Bigot, 1885 (Blanchard 1963: 185).

*coerulea*. Incorrect subsequent spelling of *caerulea* Bigot, 1885 (Brauer 1899: 498).

References: Cortés (1969: 98), separation from *Trichodischiasoror*, first record from Chile; Cortés (1975: 36, 37), first record from Uruguay (distribution given for *Trichoraea* in key, a monotypic genus based on *T.caerulea*), taxonomic notes.

***soror*** Bigot, 1885.—Neotropical: South America (Argentina, Brazil, Chile, Uruguay).

*Trichodischiasoror* Bigot, 1885b: xlvi [also 1885b: xlvi, *Bull. Soc. Ent. France*]. Holotype male (NHMUK). Type locality: Argentina, Buenos Aires, Buenos Aires.

*Trichodischiacaerulea* of Cortés (1944f: 51, 1946: 175) and Blanchard (1963: 184), not Bigot, 1885. Misidentification (Cortés 1969: 97, 1975: 37).

References: Cortés (1944f: 54), first records from Chile and Uruguay, misidentified as *Trichodischiacaerulea*; Cortés (1969: 98), separation from *T.caerulea*; Cortés (1975: 37), taxonomic notes; Cortés (1980: 105), first record from Brazil.

#### Genus *VELARDEMYIA* Valencia, 1972

***VELARDEMYIA*** Valencia, 1972a: 364. Type species: *Velardemyiaica* Valencia, 1972, by original designation [Peru].

Reference: Cortés and González (1989: 116), in key to genera of Chilean Voriini.

***ica*** Valencia, 1972.—Neotropical: South America (Chile, Peru).

*Velardemyiaica* Valencia, 1972a: 364. Holotype male (SENASA, Lozada et al. 2005: 460). Type locality: Peru, Ica, Arrabales.

Reference: Cortés and González (1989: 122), first record from Chile.

#### Genus *VORIA* Robineau-Desvoidy, 1830

***VORIA*** Robineau-Desvoidy, 1830: 195. Type species: *Vorialatifrons* Robineau-Desvoidy, 1830 (= *Tachinaruralis* Fallén, 1810), monotypy [France].

*PLAGIA* Meigen, 1838: 201. Type species: *Tachinaverticalis* Meigen, 1824 (= *Tachinaruralis* Fallén, 1810), by subsequent designation of Rondani (1856: 69) [Europe].

*XENOPLAGIA* Townsend, 1914a: 13. Type species: *Xenoplagiasetosa* Townsend, 1914, by original designation [Peru].

*ITAVORIA* Townsend, 1931d: 474. Type species: *Itavoriaaurescens* Townsend, 1931, by original designation [Brazil].

References: Coquillett (1910: 591, 619), type species of *Plagia* and *Voria* (with *Plagia* [and others] in synonymy with *Voria*); Townsend (1936b: 232), diagnosis of adults and immatures of Voriini and key to genera (including *Itavoria*, *Voria* and *Xenoplagia*); Townsend (1936c: 280), *Plagia* as synonym of *Voria*; Townsend (1939a: 385, 402, 403), redescriptions of *Itavoria*, *Voria* (with *Plagia* in synonymy) and *Xenoplagia*; Mesnil (1974: 1261), redescription (with *Plagia* in synonymy), taxonomic notes; Cortés and Campos (1974: 113) and Cortés (1984: 379), *Voria* in keys to tachinid genera of Tarapacá and Antofagasta regions; Cortés and González (1989: 116), *Voria* in key to genera of Chilean Voriini; Fleming et al. (2017: 7), synonymy of *Itavoria* and *Xenoplagia* with *Voria* [also the synonymy with *Voria* of the Afrotropical and Oriental genus *Hystricovoria* Townsend, 1928 and its synonyms *Afrovoria* Curran, 1938 and *Anavoria* Mesnil, 1953, but the present authors follow O’Hara and Cerretti (2016: 56) in recognising *Hystricovoria* as a valid genus].

***ruralis*** (Fallén, 1810).—Neotropical: southern Lesser Antilles (Trinidad & Tobago), Middle America (Mexico, Nicaragua), South America (Argentina, Brazil, Chile, Colombia, Peru, Uruguay, Venezuela). Nearctic: Canada, United States. Palaearctic: Central Asia, China [Pal.], Europe, Japan, Korean Peninsula, Middle East, Mongolia, Russia, Transcaucasia. Afrotropical: Kenya to South Africa, Yemen. Oriental: China (Yunnan), India, Nepal, Pakistan, Taiwan. Australasian & Oceanian: Australia, Papua New Guinea.

*Tachinaruralis* Fallén, 1810: 265. Lectotype male (NHRS), by designation of Crosskey (1973: 163). Type locality: Sweden, Skåne, Äsperöd [as “Esperöd”].

*Plagiaamericana* van der Wulp, 1890c: 102. Syntypes, males and females (NHMUK). Type localities: Mexico, Veracruz (Orizaba), Guerrero (Venta del Zopilote [ca. 17°46′N, 99°32′W], 2800 ft; Xocomanatlán [as “Xucumanatlan”, ca. 17°34′N, 99°37′W], 7000 ft; Omiltemi [as “Omilteme”, ca. 17°33′N, 99°41′W], 8000 ft), and Tabasco (Teapa).

*Plagiamexicana* Giglio-Tos, 1893: 5. Type(s), female (MZUT). Type locality: Mexico.

*Voriabrasiliana* Townsend, 1929: 380. Syntypes, “many males and females” (USNM). Type locality: Brazil, São Paulo, Itaquaquecetuba.

*Voriaayerzai* Blanchard, 1937: 47 (as “*Voriaayerzai*, (Brethes)”). *Nomen nudum*.

*Voriaayerzai* Blanchard, 1943c: 157 (as “*Plagiaayerzai*, Brèthes in lit.”). Syntypes, 3 males and females (MLPA). Type locality: Argentina, Buenos Aires [province or city].

Notes: The mention of a “Ht” for *Tachinaruralis* from Sweden in NHRS by Townsend (1939a: 402) is not accepted as a lectotype fixation because the specimen in question is not distinguishable from the other specimens in the type series.

*Voriaruralis* as here interpreted is almost certainly a species complex (see also Fleming et al. 2017). This complex in the New World may not include the true *Voriaruralis* described from the Palaearctic Region by Fallén (1810). Some of the names listed here in synonymy may represent distinct species.

References: Aldrich (1926a: 14), synonymy of *Plagiaamericana* with *Tachinaruralis*; Cortés (1944d: 142), *Voriabrasiliana* and *Voriaayerzai* as possible synonyms of *Tachinaruralis*, first record from Chile; Parker (1953: 64), figures of first instar larva and puparium; Thompson (1961: 28), synonymy including *Plagiamexicana* as a questionable synonym of *Tachinaruralis*, redescription, first record from Trinidad; Blanchard (1963: 169), taxonomic notes, record from Argentina; Guimarães (1971: 93), synonymy; Nihei (2016: 911), first record from Colombia.

### Unplaced genus of Dexiinae

#### Genus *SCHLINGERMYIA* Cortés, 1967

***SCHLINGERMYIA*** Cortés, 1967b: 20. Type species: *Schlingermyiavenusta* Cortés, 1967, by original designation [Chile].

Note: This genus was not placed beyond subfamily Dexiinae by the original author, Cortés (1967: 20). Guimarães (1971: 100) listed *Schlingermyia* under subfamily Dexiinae, tribe Macquartiini, following Townsend’s (1936c: 13) concept of the tribe. Most of the Neotropical genera then assigned to the Macquartiini are currently placed in the dexiine tribes Dexiini and Voriini.

***venusta*** Cortés, 1967.—Neotropical: South America (Chile).

*Schlingermyiavenusta* Cortés, 1967b: 22. Holotype male (EEAM). Type locality: Chile, Valparaíso, Marga Marga, Bosque Los Perales [as “Los Perales”, ca. 33°9′S, 71°18′W].

### Subfamily EXORISTINAE

#### Tribe ACEMYINI

##### Genus *CERACIA* Rondani, 1865

***CERACIA*** Rondani, 1865: 221. Type species: *Ceraciamucronifera* Rondani, 1865, by monotypy [Italy].

*MYOTHYRIA* van der Wulp, 1890a: 44, in key [1890e: 208, description]. Type species: *Myothyriamajorina* van der Wulp, 1890, by subsequent designation of Coquillett (1910: 573) (see O’Hara and Cerretti 2016: 63) [Mexico].

*ACEMYIOPSIS* Townsend, 1915e: 433. Type species: *Acemyiopsispunensis* Townsend, 1915, by original designation [Peru].

*CLYTHOPSIS* Townsend, 1927a: 276. Type species: *Clythopsisconfundens* Townsend, 1927 (= *Myobiabrachyptera* Thomson, 1869), by original designation [Brazil].

References: Coquillett (1910: 573), type species of *Myothyria*; Aldrich (1934: 5, 136), in key to Patagonian genera, synonymy of *Acemyiopsis*, *Clythopsis* and *Myothyria* with *Ceracia*, taxonomic notes; Townsend (1936c: 71, 270, 273, 278), diagnosis of adults and immatures of Acemyini and key to genera (including *Ceracia*), synonymy; Townsend (1939c: 255), redescription of *Ceracia* (with *Acemyiopsis*, *Clythopsis* and *Myothyria* in synonymy).

***dentata*** (Coquillett, 1895).—Neotropical: Middle America (Mexico), South America (Chile). Nearctic: Canada, United States.

*Acemyiadentata* Coquillett, 1895a: 311. Syntypes, 4 females (2 females in USNM [one with abdomen missing] and 2 females in MCZ). Type localities: USA, Florida (Georgetown), Alabama (Mobile), and California (Los Angeles County).

Reference: Aldrich (1934: 137), redescription, first record from Chile.

***subandina*** Blanchard, 1943.—Neotropical: South America (Argentina, ?Chile).

*Ceraciasubandina* Blanchard, 1943b: 19. Holotype male (INTA, Patitucci et al. 2015: 567). Type locality: Argentina, Río Negro, Comallo.

Note: Guimarães (1971: 123) recorded *Ceraciasubandina* from three places in Chile (“Angol, Concepción, Santiago”) but we have not found any of these records in the Chilean literature and suspect that they were listed for *C.subandina* in error.

### Tribe BLONDELIINI

References: Sabrosky (1981: 3), key to genera of Blondeliini in which females possess an abdominal keel and sharp, curved piercer; Wood (1985), revision of the Blondeliini of North and Central America and the West Indies.

#### Genus *ADMONTIA* Brauer & Bergenstamm, 1889

*GRAVENHORSTIA* Robineau-Desvoidy, 1863a: 924 (junior homonym of *Gravenhorstia* Boie, 1836). Type species: *Gravenhorstialongicornis* Robineau-Desvoidy, 1863 (= *Tachinagrandicornis* Zetterstedt, 1849), by original designation [France].

***ADMONTIA*** Brauer & Bergenstamm, 1889: 104 [also 1890: 36]. Type species: *Admontiapodomyia* Brauer & Bergenstamm, 1889, by monotypy [Austria, Germany, Italy, Poland, Germany and Czech Republic].

*TRICHOPAREIA* Brauer & Bergenstamm, 1889: 103 [also 1890: 35]. Type species: *Tachinaseria* Meigen, 1824, by monotypy [Germany].

*AUSTROSTAUROCHAETA* Townsend, 1931d: 476. Type species: *Degeeriaantarctica* Thomson, 1869, by original designation [probably Chile].

*POLIOPS* Aldrich, 1934: 94. Type species: *Poliopsstriatus* Aldrich, 1934, by original designation [Argentina]. **Syn. nov.**

Notes: The relative priority of *Admontia* Brauer & Bergenstamm, 1889 and *Trichopareia* Brauer & Bergenstamm, 1889, when the two are treated as synonyms, was established by Strobl (1910: 137), as the First Reviser (Article 24.2.2 of the *Code*, ICZN 1999).

The new synonymy of *Poliops* with *Admontia* is explained below under *Admontiastriata*.

References: Coquillett (1910: 503), type species of *Admontia* (as synonym of *Hyperecteina* Schiner, 1861); Aldrich (1934: 4, 94, 95), *Admontia* and *Poliops* in key to Patagonian genera, synonymy including *Trichopareia* and *Austrostaurochaeta* with *Admontia*, taxonomic notes, key to six Patagonian species of *Admontia*; Townsend (1936c: 129, 154), diagnosis of adults and immatures of Actiini and key to genera (including *Austrostaurochaeta* and *Poliops*), diagnosis of adults and immatures of Trichopareiini and key to genera (including *Admontia* and *Trichopareia*); Townsend (1940a: 193, 254, 301, 304), redescriptions of *Admontia*, *Austrostaurochaeta*, *Poliops* and *Trichopareia*; Cortés (1973a: 100), separation of *Admontia* and *Austrostaurochaeta* from *Notomanes* Aldrich; Wood (1985: 14, 17), in key to the Blondeliini of North and Central America and the West Indies, synonymy including *Austrostaurochaeta* with *Admontia*, diagnosis, taxonomic notes; Cortés (1986: 144), in key to tachinid genera of Aysén and Magallanes regions.

***antarctica*** (Thomson, 1869).—Neotropical: South America (Argentina, Chile).

*Degeeriaantarctica* Thomson, 1869: 527. Lectotype male (NHRS), by fixation of Townsend (1931b: 183) (examination of “Male Ht” from Patagonia in NHRS is regarded as a lectotype fixation). Type locality: “Patagonia” (most likely Chile, Magallanes y de la Antártica Chilena, Magallanes, Puerto del Hambre [frequently as “Port Famine”], based on localities where insects were collected during the voyage of the Swedish frigate *Eugenie*, including the lectotype of *Degeeriaantarctica*; see Persson 1971: 168).

References: Aldrich (1934: 97), redescription, first record from Argentina; Cortés (1973a: 97), taxonomic notes.

***aurata*** (Campos, 1953).—Neotropical: South America (Chile). **Comb. nov.**

*Poliopsauratus* Campos, 1953: 27. Holotype male (MNNC). Type locality: Chile, Biobío, Concepción, Tomé.

Note: *Poliopsauratus* is assumed to have the same generic features as the type species of *Poliops*, *P.striatus*, and for this reason is transferred to *Admontia*. The transfer of *P.striatus* to *Admontia* is discussed below under *A.striata*.

***calyptrata*** (Aldrich, 1934).—Neotropical: South America (Argentina, Chile). **Comb. nov.** (Fig. 4d)

*Phoroceracalyptrata* Aldrich, 1934: 73. Holotype male (USNM). Type locality: Argentina, Río Negro, Lago Correntoso.

Note: *Phoroceracalyptrata* Aldrich has the *Admontia* features of a setose facial ridge, haired parafacial and tiny fore claws in the female but the eye has scattered long hairs rather than the usual bare condition. We interpret the species as an aberrant member of the *Admontia* lineage and move it here to *Admontia* from its prior indefinite placements in Tachinidae (see References below).

References: Aldrich (1934: 69), in key to Patagonian species of *Phorocera* Robineau-Desvoidy, 1830 (*s. lato*); Cortés (1945d: 158), in key to Chilean species of *Phorocera* Robineau-Desvoidy, 1830 (*s. lato*) and *Parasetigena* Brauer & Bergenstamm, 1891, known from Argentina and not Chile; Guimarães (1971: 152), as unplaced species of Blondeliini; Henry (1987: 206), first record from Chile (in *Phorocera* but genus unplaced in Tachinidae); González (1992b: 183), record from Chile, as *Phoroceracalyptrata* (*sensu* previous authors).

***communis*** Aldrich, 1934.—Neotropical: South America (Argentina, Chile).

*Admontiacommunis* Aldrich, 1934: 99. Holotype, unspecified sex (NHMUK). Type locality: Argentina, Río Negro, eastern end of Lago Nahuel Huapí.

*Admontiacommunisalbescens* Aldrich, 1934: 100. Syntypes, 5 males (NHMUK). Type localities: Argentina, Río Negro, eastern end of Lago Nahuel Huapí and San Carlos de Bariloche [as “Bariloche”].

Notes: *Admontiacommunis* was recorded from both Argentina and Chile in the original description. Cortés and Hichins (1969: 16) gave the depository for the holotype of *A.communis* as USNM, in error.

***debilis*** Aldrich, 1934.—Neotropical: South America (Argentina, Chile).

*Admontiadebilis* Aldrich, 1934: 102. Holotype male (NHMUK). Type locality: Chile, Los Lagos, Llanquihue, Casa Pangue.

***finisterrae*** Cortés, 1986.—Neotropical: South America (Chile).

*Admontiafinisterrae* Cortés, 1986: 155. Holotype male (MEUC). Type locality: Chile, Magallanes y de la Antártica Chilena, Antártica Chilena, Islas Hermite, Isla Deceit, Caleta Toledo.

***flavibasis*** Aldrich, 1934.—Neotropical: South America (Argentina, Chile).

*Admontiaflavibasis* Aldrich, 1934: 103. Holotype female (USNM). Type locality: Argentina, Río Negro, Lago Gutiérrez.

Reference: González (1992b: 178), first record from Chile.

***pictiventris*** Aldrich, 1934.—Neotropical: South America (Argentina, Chile).

*Admontiapictiventris* Aldrich, 1934: 100. Holotype male (NHMUK). Type locality: Chile, Los Lagos, Llanquihue, Peulla.

Reference: Gramajo (1998: 96), first record from Argentina.

***striata*** (Aldrich, 1934).—Neotropical: South America (Argentina, Chile). **Comb. nov.**

*Poliopsstriatus* Aldrich, 1934: 94. Holotype male (NHMUK). Type locality: Argentina, Río Negro, eastern end of Lago Nahuel Huapí.

Notes: *Poliopsstriatus* was recorded from both Argentina and Chile in the original description.

Aldrich (1934: 94) described *Poliops* as a monotypic genus based on the new species *P.striatus*, noting: “Very similar to *Admontia*, but costal spine about as long as distance between auxiliary and first vein on costa, and third vein at base with a single setula of about the same length”. A male homotype of *P.striatus* in CNC that DMW compared to the holotype in NHMUK possesses the usual features of the Blondeliini and diagnostic characters of *Admontia* (Wood 1985: 18), including bare eye, setose facial ridge, and a few hairs on the upper parafacial below lowest frontal seta. A single setula at the base of wing vein R_4+5_ is not unique in *Admontia* to the two species originally described in *Poliops* and was even given in the original description as a characteristic of *A.finisterrae* Cortés, 1986.

#### Genus *EUCELATORIA* Townsend, 1909

***EUCELATORIA*** Townsend, 1909: 249. Type species: *Tachinaarmigera* Coquillett, 1889, by monotypy [United States].

*SPATHIMYIA* Townsend, 1912b: 318. Type species: *Spathimyiaferox* Townsend, 1912, by original designation [Peru].

*XIPHOMYIA* Townsend, 1917: 125. Type species: *Xiphomyiagladiatrix* Townsend, 1917, by original designation [Panama].

*URODEXODES* Townsend, 1919b: 572. Type species: *Urodexodescharapensis* Townsend, 1919, by original designation [Peru].

*MACHAIROMASICERA* Townsend, 1919b: 577. Type species: *Machairomasiceracarinata* Townsend, 1919, by original designation [Ecuador].

*LIXINIA* Curran, 1926: 108. Type species: *Lixiniajamaicensis* Curran, 1926, by original designation [Jamaica].

*TINALYDELLA* Townsend, 1927a: 265. Type species: *Tinalydellatinensis* Townsend, 1927, by original designation [Peru].

*OROPHOROCERA* Townsend, 1927a: 267. Type species: *Orophoroceraocellaris* Townsend, 1927, by original designation [Peru].

*HYPOMYOTHYRIA* Townsend, 1927a: 276. Type species: *Hypomyothyriahypodermica* Townsend, 1927, by original designation [Brazil].

*EUCELATORIOPSIS* Townsend, 1927a: 276. Type species: *Eucelatoriopsisteffeensis* Townsend, 1927, by original designation [Brazil].

*HELIOLYDELLA* Townsend, 1927a: 277. Type species: *Heliolydellaaurata* Townsend, 1927, by original designation [Brazil].

*TACHINOPHYTOPSIS* Townsend, 1927a: 277. Type species: *Tachinophytopsiscarinata* Townsend, 1927 (junior secondary homonym of *Machairomasiceracarinata* Townsend, 1919; = *Eucelatoriaparacarinata* Nihei & Dios, 2016), by original designation [Brazil].

*HEMILYDELLA* Townsend, 1927a: 278. Type species: *Hemilydellafasciata* Townsend, 1927, by original designation [Peru].

*LYDELLOHOUGHIA* Townsend, 1927a: 280. Type species: *Lydellohoughianana* Townsend, 1927, by original designation [Brazil].

*EUPTILODEGEERIA* Townsend, 1931d: 465. Type species: *Hypostenaobumbrata* van der Wulp, 1890, by original designation [Mexico].

*COROZALIA* Curran, 1934: 465. Type species: *Corozalialongula* Curran, 1934, by original designation [Panama].

*CELATORIOPSIS* Blanchard, 1963: 228. Type species: *Celatoriopsiseucelatorioides* Blanchard, 1963, by original designation [Argentina].

*EUCELATORIOIDEA* Thompson, 1968: 176. Type species: *Eucelatorioideanigripalpis* Thompson, 1968 (junior secondary homonym of *Chetolyganigripalpis* Bigot, 1889; = *Eucelatorianudioculata* O’Hara & Wood, **nom. nov.**, see below), by original designation [Trinidad & Tobago].

*DEXODIMYIA* Thompson, 1968: 181. Type species: *Dexodimyiadiscalis* Thompson, 1968, by original designation [Trinidad & Tobago].

*PSEUDOCELATORIA* Thompson, 1968: 190. Type species: *Pseudocelatoriarobusta* Thompson, 1968, by original designation [Trinidad & Tobago].

*HELIODEXODES* Thompson, 1968: 197. Type species: *Heliodexodesargenteus* Thompson, 1968, by original designation [Trinidad & Tobago].

*DEXODIOPSIS* Thompson, 1968: 202. Type species: *Dexodiopsisaurea* Thompson, 1968, by original designation [Trinidad & Tobago].

Notes: The relative priority of *Eucelatorioidea* Thompson, 1968, *Dexodimyia* Thompson, 1968, *Pseudocelatoria* Thompson, 1968, *Heliodexodes* Thompson, 1968 and *Dexodiopsis* Thompson, 1968, when the five are treated as synonyms, has not been established and is not of concern while all are junior synonyms of *Eucelatoria* Townsend, 1909 (as proposed by Wood 1985: 40).

References: Townsend (1936c: 86, 237), diagnosis of adults and immatures of Compsilurini and key to genera (including *Eucelatoria*, *Eucelatoriopsis*, *Euptilodegeeria*, *Heliolydella*, *Hemilydella*, *Hypomyothyria*, *Machairomasicera*, *Orophorocera*, *Spathimyia*, *Tachinophytopsis*, *Tinalydella* and *Urodexodes*), diagnosis of adults and immatures of Trypherini and key to genera (including *Corozalia*, *Lixinia*, *Lydellohoughia* and *Xiphomyia*); Townsend (1940a: 48, 50, 53, 54, 55, 56, 63, 76, 94, 97, 98, 100), redescriptions of *Eucelatoria*, *Eucelatoriopsis*, *Euptilodegeeria*, *Heliolydella*, *Hemilydella*, *Hypomyothyria*, *Machairomasicera*, *Orophorocera*, *Spathimyia*, *Tachinophytopsis*, *Tinalydella* and *Urodexodes*; Townsend (1941: 258, 279, 281, 327), redescriptions of *Corozalia*, *Lixinia*, *Lydellohoughia* and *Xiphomyia*; Thompson (1968: 174, 176), revision of *Eucelatoria* species of Trinidad, as nine genera (five new) collectively termed the “Trinidad compsilurines”; Cortés and Campos (1971: 26, 1974: 115, 116), *Hemilydella* and *Eucelatoria* in keys to tachinid genera of Tarapacá and Antofagasta regions; Sabrosky (1981: 3), in key to genera of Blondeliini in which females possess an abdominal keel and sharp, curved piercer (key also including the following generic names later synonymised with *Eucelatoria*: *Eucelatoriopsis*, *Heliodexodes*, *Heliolydella*, *Hemilydella*, *Lydellohoughia*, *Machairomasicera*, *Spathimyia*, *Tinalydella*, *Urodexodes* and *Xiphomyia*), synonymy of *Celatoriopsis* with *Eucelatoria*; Cortés (1984: 381, 382), *Eucelatoria*, *Hemilydella* and *Urodexodes* in key to tachinid genera of Tarapacá and Antofagasta regions; Wood (1985: 13, 40), in key to the Blondeliini of North and Central America and the West Indies, new synonymy of all generic names listed above with *Eucelatoria* (with the exception of the previously synonymised *Celatoriopsis*), diagnosis, taxonomic notes.

***australis*** Townsend, 1911.—Neotropical: eastern Lesser Antilles (Saint Vincent), southern Lesser Antilles (Trinidad & Tobago), South America (Brazil, Chile, Peru).

*Eucelatoriaaustralis* Townsend, 1911: 140, based on female reproductive system [1912b: 315, adult description]. Lectotype female (USNM), by fixation of Townsend (1912b: 316) (description of female “Type” [dissection TD 4025] from Piura in USNM is regarded as a lectotype fixation). Type locality: Peru, Piura, Piura.

Note: Aldrich (1927a: 19) synonymised *Compsiluraoppugnator* Walton, 1914 from Puerto Rico with *Eucelatoriaaustralis* Townsend and this synonymy was followed by Guimarães (1971: 133). Sabrosky (1981) recognised *Eucelatoriaoppugnator* as valid and Wood (1985: 44) did also but with the note: “[? = *australis* (c/f Aldrich 1927a: 19)]”.

References: Aldrich (1927a: 19), first record from St. Vincent; Sauer (1946: 21), first record from Brazil; Thompson (1968: 200), redescription, first record from Trinidad; Cortés and Campos (1971: 81), first record from Chile; Cortés (1984: 386), taxonomic notes; Vergara de Sánchez (1987: 10), redescription, figures of male and female terminalia.

***digitata*** Sabrosky, 1981.—Neotropical: South America (Chile, Peru).

*Eucelatoriadigitata* Sabrosky, 1981: 11. Holotype male (USNM). Type locality: Peru, Lima, San Diego.

Note: *Eucelatoriadigitata* was recorded from both Chile and Peru in the original description.

References: Cortés (1984: 386), taxonomic notes; Vergara de Sánchez (1987: 10, 11), redescription, figures of male and female terminalia.

***fasciata*** (Townsend, 1927).—Neotropical: southern Lesser Antilles (Trinidad & Tobago), South America (Chile, Peru).

*Hemilydellafasciata* Townsend, 1927a: 315. Holotype male (USNM). Type locality: Peru, Piura, Río Macará, La Tina, on border with Ecuador, 1370 ft.

*nudioculata* O’Hara & Wood, **nom. nov.**—Not Chile [Trinidad].

*Eucelatorioideanigripalpis* Thompson, 1968: 177 (junior secondary homonym of *Chetolyganigripalpis* Bigot, 1889). Holotype female (CNC). Type locality: Trinidad.

*Eucelatorianudioculata* O’Hara & Wood, **nom. nov.** for *Eucelatorioideanigripalpis* Thompson, 1968.

Note: *Eucelatorioideanigripalpis* Thompson, 1968 from Trinidad, the type species of *Eucelatorioidea* Thompson, 1968, became a junior secondary homonym of *Chetolyganigripalpis* Bigot, 1889 from Mexico when transferred to *Eucelatoria* Townsend, 1909 by Wood (1985: 44). Both names were treated as valid in that work and the junior homonym was not renamed “pending a revision of the genus” (Wood 1985: 44). This situation has continued to the present and both names are listed as valid in the most recent version of the checklist of world Tachinidae (O’Hara et al. 2020: 225). In the interests of nomenclatural stability, we hereby propose the new name *Eucelatorianudioculata* to replace the preoccupied name *Eucelatorioideanigripalpis* Thompson. The same type material applies to the new name. The specific epithet *nudioculata* refers to the bare eye that was noted by Thompson (1968: 176) as a characteristic of his new genus *Eucelatorioidea*, for which *E.nigripalpis* was designated type species.

***oblonga*** O’Hara & Wood, **nom. nov.**—Neotropical: South America (Chile).

*Urodexodeselongatum* Cortés & Campos, 1974: 124 (junior secondary homonym of *Exoristaelongata* van der Wulp, 1890). Holotype male (MEUC). Type locality: Chile, Arica y Parinacota, Parinacota, Belén, 3500 m.

*Eucelatoriaoblonga* O’Hara & Wood, **nom. nov.** for *Urodexodeselongatum* Cortés & Campos, 1974.

Note: *Urodexodeselongatum* Cortés & Campos, 1974, from Chile, is a junior secondary homonym of *Exoristaelongata* van der Wulp, 1890, the valid name of a Costa Rican species of *Eucelatoria* (Wood 1985: 43). The two species names are listed as valid in the most recent version of the checklist of world Tachinidae (O’Hara et al. 2020: 224). In the interests of nomenclatural stability, we hereby propose the new name *Eucelatoriaoblonga* to replace the preoccupied name *Urodexodeselongatum* Cortés & Campos. The same type material applies to the new name. The specific epithet *oblonga*, Latin for longer than broad, refers to the elongated appearance of the species.

***parkeri*** (Sabrosky, 1952).—Neotropical: South America (Argentina, Brazil, Chile, Uruguay).

*Eucelatoriopsisparkeri* Sabrosky, 1952: 325. Holotype male (USNM). Type locality: Uruguay, Montevideo, Montevideo.

References: Cortés (1967b: 12), first record from Chile; Guimarães (1977c: 35), first record from Brazil; Boldt et al. (1991: 843), first record from Argentina.

#### Genus *EUHALIDAYA* Walton, 1914

***EUHALIDAYA*** Walton, 1914: 130. Type species: *Euhallidayaseverinii* Walton, 1914 (= *Biomyiagenalis* Coquillett, 1897), by original designation [United States].

*OOMEIGENIA* Townsend, 1915e: 434 (as “*Oömeigenia*”). Type species: *Oomeigeniachosica* Townsend, 1915, by original designation [Peru].

*CLYTHOXYNOPS* Townsend, 1927a: 272. Type species: *Clythoxynopsorbitalis* Townsend, 1927, by original designation [Brazil].

*BACULOCAPTUS* Cortés, 1968a: 106. Type species: *Baculocaptusvalparadisi* Cortés, 1968, by original designation [Chile].

*EUHALLIDAYA*. Incorrect original spelling of *Euhalidaya* Walton, 1914 (Walton 1914: 130) (see note).

*CLITHOXYNOPS*. Incorrect original spelling of *Clythoxynops* Townsend, 1927 (Townsend 1927a: 272).

Notes: The genus name *Euhalidaya* Walton was originally proposed as *Euhallidaya* but subsequent authors (e.g., Curran 1934: 460) changed the spelling to *Euhalidaya*. This changed spelling would normally be considered an incorrect subsequent spelling but because it is in prevailing usage and is attributed to Walton (1914), it is deemed to be the correct original spelling (Article 33.3.1 of the *Code*, ICZN 1999).

There are two original spellings of *Clythoxynops* in Townsend (1927a): *Clithoxynops* (p. 272) and *Clythoxynops* (p. 299). The correct original spelling was selected as *Clythoxynops* by Townsend (1927b, see entry for “page 272, line 17 [from] top” in the unpaginated errata of Townsend 1927a), as the First Reviser (Article 24.2.3 of the *Code*, ICZN 1999).

References: Townsend (1936c: 71, 237), diagnosis of adults and immatures of Acemyini and key to genera (including *Euhalidaya*), diagnosis of adults and immatures of Trypherini and key to genera (including *Clythoxynops* and *Oomeigenia*); Townsend (1939c: 259), redescription of *Euhalidaya*; Townsend (1941: 257, 296), redescriptions of *Clythoxynops* and *Oomeigenia*; Cortés (1976: 3), difference between *Clythoxynops* and *Baculocaptus*; Wood (1985: 14, 45), in key to the Blondeliini of North and Central America and the West Indies, synonymy of *Baculocaptus*, *Clythoxynops* and *Oomeigenia* with *Euhalidaya*, diagnosis, taxonomic notes.

***valparadisi*** (Cortés, 1968).—Neotropical: South America (Chile).

*Baculocaptusvalparadisi* Cortés, 1968a: 108. Holotype male (EEAM). Type locality: Chile, Valparaíso, Valparaíso, Viña del Mar.

#### Genus *INCAMYIA* Townsend, 1912

***INCAMYIA*** Townsend, 1912b: 317. Type species: *Incamyiacuzcensis* Townsend, 1912, by original designation [Peru].

*SPHALLOGLANDULUS* Townsend, 1915e: 438. Type species: *Sphalloglandulusunicus* Townsend, 1915, by original designation [Peru].

*PROPHRYNOPSIS* Townsend, 1927a: 273. Type species: *Prophrynopsisperuviana* Townsend, 1927, by original designation [Peru].

References: Aldrich (1928b: 14), synonymy of *Sphalloglandulus* with *Incamyia*, key to four species; Aldrich (1934: 4, 65), in key to Patagonian genera, *Sphalloglandulus* in synonymy, taxonomic notes, key to three Patagonian species; Townsend (1936c: 86, 190, 282), diagnosis of adults and immatures of Compsilurini and key to genera (including *Incamyia*), diagnosis of adults and immatures of Sturmiini and key to genera (including *Prophrynopsis*), synonymy; Townsend (1940a: 560), redescription of *Incamyia* (with *Sphalloglandulus* in synonymy); Townsend (1941: 124), redescription of *Prophrynopsis*; Parker (1953: 68), figures of first instar larva and puparium; Cortés (1968b: 18), key to four species; Cortés and Campos (1971: 25, 87), in key to tachinid genera of Tarapacá and Antofagasta regions, key to six species of these provinces; Guimarães (1971: 136), synonymy of *Prophrynopsis* with *Incamyia*; Cortés and Campos (1974: 114) and Cortés (1984: 380), in keys to tachinid genera of Tarapacá and Antofagasta regions; Sabrosky (1981: 3), in key to genera of Blondeliini in which females possess an abdominal keel and sharp, curved piercer; Cortés (1986: 144), in key to tachinid genera of Aysén and Magallanes regions; González and Henry (1992: 36), key to the ten Chilean species.

***charlini*** Cortés, 1968.—Neotropical: South America (Chile).

*Incamyiacharlini* Cortés, 1968b: 19. Holotype male (EEAM). Type locality: Chile, Metropolitana de Santiago, Santiago, Maipú, Rinconada.

Reference: Cortés (1986: 158), taxonomic notes.

***chilensis*** Aldrich, 1928.—Neotropical: South America (Argentina, Chile, Uruguay).

*Incamyiachilensis* Aldrich, 1928b: 16. Holotype male (USNM). Type locality: Chile, Araucanía, Malleco, Angol.

References: Aldrich (1934: 66), redescription, first record from Argentina; Parker et al. (1951 [pages unknown], also 1953: 55, 58, 68), first record from Uruguay; Cortés (1952: 109), first record from Juan Fernández Islands with note on the possible subspecific status of the island population; Caltagirone (1953: 90, 92), description and figure of first instar larva; Blanchard (1963: 203), redescription, wing figure; Molina-Ochoa et al. (2003: 259), record from Brazil attributed to Guimarães (1977c) but *Incamyiachilensis* not recorded from Brazil in that work.

***cinerea*** Cortés & Campos, 1971.—Neotropical: South America (Chile).

*Incamyiacinerea* Cortés & Campos, 1971: 88. Holotype male (EEAM). Type locality: Chile, Tarapacá, Tamarugal, Mamiña, 2600 m (20°06′S, 69°16′W) (coordinates and elevation given on p. 11).

***cuzcensis*** Townsend, 1912.—Neotropical: South America (Chile, Peru).

*Incamyiacuzcensis* Townsend, 1912b: 317. Holotype female (USNM). Type locality: Peru, Cusco [region or city, as “Cuzco”].

*cuzcoensis*. Incorrect subsequent spelling of *cuzcensis* Townsend, 1912 (Vimmer and Soukup 1940b: 361).

References: Aldrich (1928b: 14), synonymy of *Sphalloglandulusunicus* Townsend, 1915 from Peru with *Incamyiacuzcensis*, but this synonymy overlooked or not followed by later authors (except Aldrich 1934: 65); Cortés and Campos (1971: 89), first record from Chile.

***nuda*** Aldrich, 1934.—Neotropical: South America (Argentina, Chile).

*Incamyianuda* Aldrich, 1934: 66. Syntypes, 6 males (USNM). Type locality: Argentina, Río Negro, Lago Nahuel Huapí, San Carlos de Bariloche [as “Bariloche”].

Reference: González and Henry (1992: 36), first record from Chile.

***perezi*** Cortés & Campos, 1971.—Neotropical: South America (Chile).

*Incamyiaperezi* Cortés & Campos, 1971: 89. Holotype male (EEAM). Type locality: Chile, Arica y Parinacota, Parinacota, Putre, 3530 m (18°12′S, 69°35′W) (coordinates and elevation given on p. 11).

***picta*** Cortés, 1976.—Neotropical: South America (Chile).

*Incamyiapicta* Cortés, 1976: 5. Holotype male (MEUC). Type locality: Chile, Coquimbo, Elqui, Baños El Toro, 3300–4000 m [ca. 29°50′S, 70°1′W].

***sandovali*** Cortés & Campos, 1971.—Neotropical: South America (Chile).

*Incamyiasandovali* Cortés & Campos, 1971: 90. Holotype male (EEAM). Type locality: Chile, Arica y Parinacota, Parinacota, Putre, 3530 m (18°12′S, 69°35′W) (coordinates and elevation given on p. 11).

***spinicosta*** Aldrich, 1928.—Neotropical: South America (Argentina, Chile).

*Incamyiaspinicosta* Aldrich, 1928b: 15. Holotype male (USNM). Type locality: Chile, Valparaíso, Marga Marga, Bosque Los Perales [as “Perales”, ca. 33°9′S, 71°18′W].

Reference: Aldrich (1934: 67), first description of female, first record from Argentina.

***striata*** Aldrich, 1928.—Neotropical: South America (Chile, Peru).

*Incamyiastriata* Aldrich, 1928b: 16. Holotype male (USNM). Type locality: Peru, Junín, La Oroya.

Reference: Cortés and Campos (1971: 91), first record from Chile.

#### Genus *MYIOPHARUS* Brauer & Bergenstamm, 1889

***MYIOPHARUS*** Brauer & Bergenstamm, 1889: 161 [also 1890: 93]. Type species: *Myiopharusmetopia* Brauer & Bergenstamm, 1889, by monotypy [Mexico].

*DIDYMA* van der Wulp, 1890a: 43, in key [1890e: 156, description]. Type species: *Didymaalbomicans* van der Wulp, 1890, by subsequent designation of Townsend *in*Williston (1908: 379, as “*albombicans*”) [Mexico].

*PARALISPE* Brauer & Bergenstamm, 1891: 337 [also 1891: 33]. Type species: *Paralispebrasiliana* Brauer & Bergenstamm, 1891, by monotypy [Brazil].

*PARADORIA* Brauer & Bergenstamm, 1891: 339 [also 1891: 35]. Type species: *Paradorianigra* Brauer & Bergenstamm, 1891, by monotypy [Venezuela].

*MESOCHAETA* Brauer & Bergenstamm, 1891: 341 [also 1891: 37]. Type species: *Didymacommixta* van der Wulp, 1890 (= *Phorocerabarbata* Bigot, 1889; *commixta* cited as “*connexa*” by Brauer & Bergenstamm 1891: 341, in error), by monotypy [Mexico].

*METADORIA* Brauer & Bergenstamm, 1893: 29 [also 1893: 117]. Type species: *Metadoriamexicana* Brauer & Bergenstamm, 1893 (= *Phorocerabarbata* Bigot, 1889), by monotypy [Mexico].

*HEMIARGYRA* Townsend, 1908: 88. Type species: *Hemiargyranigra* Townsend, 1908 (junior secondary homonym of *Paradorianigra* Brauer & Bergenstamm, 1891; = *Phoroceranigrita* van der Wulp, 1890), by original designation [Costa Rica].

*MUSCINOTHELAIRA* Townsend, 1916e: 310. Type species: *Muscinothelairalutzi* Townsend, 1916, by original designation [Brazil].

*AUSTROLYDELLA* Townsend, 1919b: 573. Type species: *Austrolydellaassimilis* Townsend, 1919, by original designation [Peru].

*GYMNODORIA* Townsend, 1927a: 260. Type species: *Gymnodoriacapitata* Townsend, 1927, by original designation [Peru].

*EUHEMIARGYRA* Townsend, 1927a: 260. Type species: *Euhemiargyraparva* Townsend, 1927, by original designation [Brazil].

*HEMIARGYROPSIS* Townsend, 1927a: 260. Type species: *Hemiargyropsisfrontalis* Townsend, 1927, by original designation [Peru].

*DACTYLODIDYMA* Townsend, 1927a: 260. Type species: *Dactylodidymadubia* Townsend, 1927, by original designation [Brazil].

*THELYPHAENOPSIS* Townsend, 1927a: 262. Type species: *Thelyphaenopsisatra* Townsend, 1927, by original designation [Brazil].

*BOLODORIA* Townsend, 1927a: 262. Type species: *Bolodoriayahuarmayana* Townsend, 1927, by original designation [Peru].

*DIDYMOPS* Townsend, 1927a: 262 (junior homonym of *Didymops* Rambur, 1842 and *Didymops* Szilády, 1922). Type species: *Didymopsyahuarmayensis* Townsend, 1927, by original designation [Peru].

*MAYOPHORINIA* Townsend, 1927a: 263. Type species: *Mayophoriniaangusta* Townsend, 1927, by original designation (see note) [Peru].

*ARGYRODORIA* Townsend, 1927a: 265. Type species: *Argyrodoriahemiargyroides* Townsend, 1927, by original designation [Brazil].

*NEARGYROPHYLAX* Townsend, 1927a: 265. Type species: *Neargyrophylaxargentescens* Townsend, 1927, by original designation [Brazil].

*HEMIARGYROPHYLAX* Townsend, 1927a: 265. Type species: *Hemiargyrophylaxpunctilucis* Townsend, 1927, by original designation [Peru].

*OXYNOPSIS* Townsend, 1927a: 270. Type species: *Oxynopsisbrasiliensis* Townsend, 1927, by original designation [Brazil].

*MYIOXYNOPS* Townsend, 1927a: 278. Type species: *Myioxynopspalpalis* Townsend, 1927, by original designation [Peru].

*HYPOPHORINIA* Townsend, 1927a: 279. Type species: *Hypophoriniahyphena* Townsend, 1927, by original designation [Brazil].

*METARRHINOMYIA* Townsend, 1927a: 279. Type species: *Metarrhinomyiaangusta* Townsend, 1927 (junior secondary homonym of *Mayophoriniaangusta* Townsend, 1927; = *Myiopharuscharapensis* O’Hara & Wood, **nom. nov.**, see below), by original designation (see note) [Peru].

*MELANODORIA* Townsend, 1927a: 280. Type species: *Melanodorianigrisquamis* Townsend, 1927, by original designation [Peru].

*NEOXYNOPS* Townsend, 1934b: 403. Type species: *Neoxynopsnana* Townsend, 1934, by original designation [Brazil].

*OXYNOPSALIA* Curran, 1934: 467. Type species: *Oxynopsalianitida* Curran, 1934, by original designation [Panama].

*ANOXYNOPSELLA* Townsend, 1935: 226. Type species: *Anoxynopsellaargentescens* Townsend, 1935 (junior secondary homonym of *Neargyrophylaxargentescens* Townsend, 1927; = *Myiopharusargentata* Nihei & Dios, 2016), by original designation [Brazil].

*NEOXYNOPSOIDEA* Thompson, 1968: 149. Type species: *Neoxynopsoideaclaripalpis* Thompson, 1968, by original designation [Trinidad & Tobago].

*STENOCHAETA* Thompson, 1968: 159. Type species: *Stenochaetaclaripalpis* Thompson, 1968 (junior secondary homonym of *Neoxynopsoideaclaripalpis* Thompson, 1968; = *Myiopharusincognitus* O’Hara & Wood, **nom. nov.**, see below), by original designation [Trinidad & Tobago].

*NEOARGYROPHYLAX*. Incorrect subsequent spelling of *Neargyrophylax* Townsend, 1927 (Guimarães 1971: 142, 297; Toma and Nihei 2006: 242, 249).

References: Coquillett (1910: 533, 550, 568, 572), type species of *Didyma*, *Hemiargyra*, *Metadoria* and *Myiopharus* (with *Hemiargyra* in synonymy with *Metadoria*); Aldrich (1924: 216), synonymy of *Hemiargyra* with *Myiopharus*; Aldrich (1934: 4, 62), in key to Patagonian genera, synonymy (*Hemiargyra* with *Myiopharus*), taxonomic notes; Townsend (1936c: 78, 82, 86, 204, 237, 273, 275), diagnosis of adults and immatures of Anacamptomyiini and key to genera (including *Gymnodoria*), diagnosis of adults and immatures of Elodiini and key to genera (including *Metarrhinomyia*), diagnosis of adults and immatures of Compsilurini and key to genera (including *Anoxynopsella*), diagnosis of adults and immatures of Carceliini and key to genera (including *Myioxynops*), diagnosis of adults and immatures of Trypherini and key to genera (including *Argyrodoria*, *Austrolydella*, *Bolodoria*, *Didyma*, *Didymops*, *Hemiargyra*, *Hemiargyrophylax*, *Hemiargyropsis*, *Hypophorinia*, *Mayophorinia*, *Melanodoria*, *Mesochaeta*, *Metadoria*, *Muscinothelaira*, *Myiopharus*, *Neargyrophylax*, *Neoxynops*, *Oxynopsalia*, *Oxynopsis*, *Paradoria*, *Paralispe* and *Thelyphaenopsis*), *Dactylodidyma* and *Euhemiargyra* as synonyms of *Paradoria*; Townsend (1940a: 9, 21, 32), redescriptions of *Gymnodoria*, *Metarrhinomyia* and *Anoxynopsella*; Townsend (1941: 154, 239–322), redescriptions of *Myioxynops* and the aforementioned genera of Trypherini (with *Dactylodidyma* and *Euhemiargyra* in synonymy with *Paradoria*); Guimarães (1971: 141), *Hemiargyra* in synonymy with *Myiopharus* (following Aldrich 1924, 1934, not Townsend 1941); Wood (1985: 13, 14, 15, 60), in key to the Blondeliini of North and Central America and the West Indies, synonymy (including many of the names above as new generic synonyms), diagnosis, taxonomic notes.

*charapensis* O’Hara & Wood, **nom. nov.**—Not Chile [Peru].

*Metarrhinomyiaangusta* Townsend, 1927a: 329 (junior secondary homonym of *Mayophoriniaangusta* Townsend, 1927, by First Reviser action below). Holotype female (USNM). Type locality: Peru, Cajamarca, Río Charapi [as “Rio Charape”, ca. 5°25′S, 78°59′W].

*Myiopharuscharapensis* O’Hara & Wood, **nom. nov.** for *Metarrhinomyiaangusta* Townsend, 1927.

Note: *Mayophoriniaangusta* Townsend, 1927 (type species of *Mayophorinia*) and *Metarrhinomyiaangusta* Townsend, 1927 (type species of *Metarrhinomyia*), both from Peru, were described in the same publication (Townsend 1927a: 326, 329) and became secondary homonyms when the generic names were synonymised with *Myiopharus* by Wood (1985: 61, 62). The two species names are listed as valid in the most recent version of the checklist of world Tachinidae (O’Hara et al. 2020: 257). As the First Reviser (Article 24.2.2 of the *Code*, ICZN 1999), we hereby fix *Mayophoriniaangusta* as the senior homonym. In the interests of nomenclatural stability, we propose the new name *Myiopharuscharapensis* to replace the name of the junior homonym *Metarrhinomyiaangusta*. The same type material applies to the new name. The specific epithet *charapensis* is based on the type locality of Río Charapi.

*incognitus* O’Hara & Wood, **nom. nov.**—Not Chile [Trinidad].

*Stenochaetaclaripalpis* Thompson, 1968: 159 (junior secondary homonym of *Neoxynopsoideaclaripalpis*Thompson 1968). Holotype male (CNC). Type locality: Trinidad, “Legerville Mt.” [not located].

*Myiopharusincognitus* O’Hara & Wood, **nom. nov.** for *Stenochaetaclaripalpis* Thompson, 1968.

Note: *Neoxynopsoidea* and *Stenochaeta* were described from Trinidad in the same work by Thompson (1968), along with their type species *Neoxynopsoideaclaripalpis* and *Stenochaetaclaripalpis*. The names of the two type species became secondary homonyms when the generic names were transferred to *Myiopharus* Brauer & Bergenstamm by Wood (1985: 62) and both were listed under “Included species” of *Myiopharus* (p. 64). The relative priority of *Neoxynopsoideaclaripalpis* and *Stenochaetaclaripalpis*, when both are placed in *Myiopharus*, was established by Wood (1985: 64) as the First Reviser (Article 24.2.2 of the *Code*, ICZN 1999) when he noted under the latter: “Although a secondary homonym of *claripalpis* (Thompson) 1968: 149, this species is not renamed, pending a revision of the genus”. This situation has continued to the present and both names are listed as valid in the most recent version of the checklist of world Tachinidae (O’Hara et al. 2020: 258). In the interests of nomenclatural stability, we hereby propose the new name *Myiopharusincognitus* to replace the name of the junior homonym *Stenochaetaclaripalpis*. The same type material applies to the new name. The specific epithet *incognitus* was inspired by the type locality of “Legerville Mt.” that we have been unable to locate.

***pirioni*** Aldrich, 1934.—Neotropical: South America (Chile).

*Myiopharuspirioni* Aldrich, 1934: 64. Holotype male (USNM). Type locality: Chile, Valparaíso, Marga Marga, Bosque Los Perales [as “Perales”, ca. 33°9′S, 71°18′W].

Note: Marshall et al. (2008) observed a female of *Myiopharuspirioni* Aldrich feeding on the regurgitate of a leaf beetle larva (*Procalus* Clark, Chrysomelidae).

*rufopalpus* O’Hara & Wood, **nom. nov.**—Not Chile [Brazil].

*Paralispepalpalis* Townsend, 1929: 376 (junior secondary homonym of *Myioxynopspalpalis* Townsend, 1927). Holotype female (USNM). Type locality: Brazil, São Paulo, Itaquaquecetuba.

*Myiopharusrufopalpus* O’Hara & Wood, **nom. nov.** for *Paralispepalpalis* Townsend, 1929.

Note: *Myioxynopspalpalis* Townsend, 1927 (type species of *Myioxynops* Townsend, 1927) from Peru and *Paralispepalpalis* Townsend, 1929 from Brazil became secondary homonyms when the genera to which they belonged, *Myioxynops* Townsend, 1927 and *Paralispe* Brauer & Bergenstamm, 1891, were synonymised with *Myiopharus* Brauer & Bergenstamm, 1889 by Wood (1985: 60, 62). The two species names are listed as valid in the most recent version of the checklist of world Tachinidae (O’Hara et al. 2020: 260). In the interests of nomenclatural stability, we hereby propose the new name *Myiopharusrufopalpus* to replace the name of the junior homonym *Paralispepalpalis*. The same type material applies to the new name. The specific epithet *rufopalpus* refers to the colour of the palpus, described by Townsend (1929: 376) as “light rufous on swollen portion”.

***subaeneus*** Aldrich, 1934.—Neotropical: South America (Chile).

*Myiopharussubaeneus* Aldrich, 1934: 63. Holotype male (USNM). Type locality: Chile, Valparaíso, Marga Marga, Bosque Los Perales [as “Perales”, ca. 33°9′S, 71°18′W].

#### Genus *NOTOMANES* Aldrich, 1934

***NOTOMANES*** Aldrich, 1934: 93. Type species: *Tachinamaura* Walker, 1836 (= *Tachinabasalis* Walker, 1836), by original designation [Chile].

Reference: Cortés (1986: 144), in key to tachinid genera of Aysén and Magallanes regions.

***basalis*** (Walker, 1836).—Neotropical: South America (Chile).

*Tachinabasalis* Walker, 1836: 351. Lectotype female (NHMUK), by fixation of Aldrich (1934: 94) (examination of “type female” from Port Famine in NHMUK is regarded as a lectotype fixation). Type locality: Chile, Magallanes y de la Antártica Chilena, Magallanes, Puerto del Hambre [as “Port Famine”].

*Tachinamaura* Walker, 1836: 352. Lectotype male (NHMUK), by fixation of Aldrich (1934: 94) (examination of “type male” from Port Famine in NHMUK is regarded as a lectotype fixation). Type locality: Chile, Magallanes y de la Antártica Chilena, Magallanes, Puerto del Hambre [as “Port Famine”].

Note: The relative priority of *Tachinabasalis* Walker, 1836 and *Tachinamaura* Walker, 1836, when the two are treated as synonyms, was established by Austen (1907: 330), as the First Reviser (Article 24.2.2 of the *Code*, ICZN 1999). Aldrich (1934: 93), Cortés (1963: 243, 1986: 158) and Cortés and Hichins (1969: 66) treated *maura* as the valid name but Guimarães (1971: 99) gave priority to *basalis* based on the First Reviser action of Austen (1907). Cortés (1973a: 99) argued that *maura* should have priority because it was chosen over *basalis* as the type species of *Notomanes* Aldrich, 1934 and was based on a male type in better condition than the female type of *basalis*, but the nomenclatural action of the First Reviser cannot be set aside on these grounds. Cortés (1986: 158) continued use of the name *Notomanesmaura*.

References: Aldrich (1934: 93), taxonomic notes; Cortés (1963: 243), notes on name-bearing types of *Tachinabasalis* and *Tachinamaura* in NHMUK; Cortés (1973a: 99), comparison of recent Chilean specimens with the redescription of Aldrich (1934: 93, as *Notomanesmaura*).

#### Genus *PHASMOPHAGA* Townsend, 1909

***PHASMOPHAGA*** Townsend, 1909: 243. Type species: *Phasmophagaantennalis* Townsend, 1909, by original designation [United States].

*PHASMOVORA* Cortés, 1968a: 102. Type species: *Phasmovoraphasmophagae* Cortés, 1968, by original designation [Chile].

References: Townsend (1936c: 129), diagnosis of adults and immatures of Actiini and key to genera (including *Phasmophaga*); Townsend (1940a: 246), redescription of *Phasmophaga*; Cortés (1976: 4), difference between *Gilvella* Mesnil, 1960 (a synonym of *Anisia* van der Wulp, 1890) and *Phasmovora*; Wood (1985: 14, 72), in key to the Blondeliini of North and Central America and the West Indies, synonymy of *Phasmovora* with *Phasmophaga*, diagnosis, taxonomic notes.

***phasmophagae*** (Cortés, 1968).—Neotropical: South America (Chile).

*Phasmovoraphasmophagae* Cortés, 1968a: 105. Holotype male (EEAM). Type locality: Chile, Maule, Curicó, Cajón del Río Claro, 15 km east of Los Queñes, 900 m.

#### Genus *STELEONEURA* Stein, 1924

***STELEONEURA*** Stein, 1924: 151. Type species: *Steleoneuraczernyi* Stein, 1924, by monotypy [Spain].

*EMBIOMYIA* Aldrich, 1934: 29. Type species: *Embiomyiaaustralis* Aldrich, 1934, by original designation [Argentina]. **Syn. nov.**

Notes: *Steleoneura* was most recently characterised by Wood (1985: 80) and was included in the keys to tachinid genera of America north of Mexico (Wood 1987: 110) and Central America (Wood and Zumbado 2010: 1380). A similarity between *Steleoneura* and Chilean *Embiomyia* (and South African genus *Pararondania* Villeneuve, 1916) was noted by Wood (1985: 81) based on the shared possession of “medially separated antennae, elongate pedicel, short first flagellomere and bulbous-based arista, long straight prosternal setae, absence of lateral scutellar bristles, 2 postpronotal bristles, and vein M ending in R_4+5_”. Wood and Zumbado (2010: 1412) further noted that *Embiomyia* “may be congeneric with *Steleoneura*”. These previous authors were reluctant to synonymise *Embiomyia* with *Steleoneura* because they had not seen a female of the single known species, *E.australis*. The female abdomen in *Steleoneura* is distinctive: “globular, ovipositor telescopic, extending ventrally from apex of abdomen (fig. 50)” (Wood 1985: 81). Aldrich (1934: 31) had simply described the shape of the female abdomen as “bluntly pointed”. We have examined three females of *E.australis* in CNC collected in Chile by JEOH in 2015 (and unidentified until after the trip report published by Stireman et al. 2016) and they possess the same peculiar ventrally-directed ovipositor that characterises *Steleoneura* species. Based on this finding and the other similarities between *Embiomyia* and *Steleoneura* noted above [but not “medially separated antennae”], we here synonymise the two generic names. *Steleoneura* is a genus with unusual features for a blondeliine and it could be misplaced here.

References: (*Steleoneura* Stein, 1924 is inexplicably missing from Townsend’s comprehensive *Manual of Myiology*); Townsend (1936c: 106), diagnosis of adults and immatures of Phoroceratini and key to genera (including *Embiomyia*); Townsend (1940a: 121), redescription of *Embiomyia*.

***australis*** (Aldrich, 1934).—Neotropical: South America (Argentina, Chile). **Comb. nov.** (Fig. 4e)

*Embiomyiaaustralis* Aldrich, 1934: 30. Holotype male (NHMUK). Type locality: Argentina, Río Negro, eastern end of Lago Nahuel Huapí.

Note: *Embiomyiaaustralis* was recorded from both Argentina and Chile in the original description.

References: Wood (1985: 81, 86) and Wood and Zumbado (2010: 1412), taxonomic notes.

### Tribe ERYCIINI

#### Genus *CARCELIA* Robineau-Desvoidy, 1830

***CARCELIA*** Robineau-Desvoidy, 1830: 176. Type species: *Carceliabombylans* Robineau-Desvoidy, 1830, by subsequent designation of Coquillett (1910: 518) (see Evenhuis et al. 2010: 52) [France].

References: Coquillett (1910: 518), type species (given as “*bombylans* … by designation of Desvoidy … vol. 1, 1863, p. 220”); Townsend (1936c: 204), diagnosis of adults and immatures of Carceliini and key to genera (including *Carcelia*); Townsend (1941: 143), redescription of *Carcelia*; Cortés (1986: 144), in key to tachinid genera of Aysén and Magallanes regions.

***halliana*** Cortés, 1945.—Neotropical: South America (Argentina, Chile).

*Carceliahalliana* Cortés, 1945c: 27. Holotype male (USNM). Type locality: Chile, Araucanía, Malleco, Angol.

Note: This species has not been assigned to a *Carcelia* subgenus.

Reference: Gramajo (1998: 97), first record from Argentina.

#### Genus *DRINO* Robineau-Desvoidy, 1863

***DRINO*** Robineau-Desvoidy, 1863a: 250. Type species: *Drinovolucris* Robineau-Desvoidy, 1863 (= *Tachinalota* Meigen, 1824), by original designation [France].

Note: The more notable diagnostic features of *Drino* within the Erycini are the short or absent ocellar setae, bare parafacial, facial ridge bare above lowest third, postpronotal setae more or less in line, four katepistemal setae, single setula at the base of wing vein R_4+5_, and row of even and closely spaced anterodorsal setae on the hind tibia (Wood 1987: 1215; Wood and Zumbado 2010: 1366). The two species moved here to *Drino* share these characteristics.

References: Aldrich (1934: 5, 138), in key to Patagonian genera, taxonomic notes (as *Sturmia* Robineau-Desvoidy, 1830); Cortés (1944g: 161), key to Chilean species (as *Sturmia*); Thompson (1966: 391, 393), key to Trinidad species (as *Drino*), taxonomic notes; Cortés and Campos (1974: 115) and Cortés (1984: 381), in keys to tachinid genera of Tarapacá and Antofagasta regions (as *Sturmia*); ICZN (2012: 242), ruling to conserve current usage of generic names *Sturmia* and *Drino*.

***festiva*** (Cortés, 1944).—Neotropical: South America (Argentina, Chile). **Comb. nov.**

*Sturmiafestiva* Cortés, 1944g: 163. Holotype male (USNM). Type locality: Chile, Valparaíso, Marga Marga, Bosque Los Perales [as “Perales, prov. de Valparaíso”, ca. 33°9′S, 71°18′W].

Note: *Sturmiafestiva* was treated as a species of *Sturmia* Robineau-Desvoidy, 1830 by previous authors (e.g., Guimarães 1971: 192; Cortés and Hichins 1979: 115; Liljesthröm 1980: 135; Henry 1987: 200). It is moved here to *Drino* Robineau-Desvoidy and resembles the widespread *Drinorhoeo* (Walker, 1849) in possessing a bright yellow abdominal tergite 5 that contrasts with the gray pruinose colour of the previous segments. *Drinorhoeo*, if correctly identified throughout its range, occurs from Canada (O’Hara and Wood 2004: 120) to Argentina (Blanchard and De Santis 1975: 34), including Costa Rica (Smith et al. 2007: 4968).

References: Campos (1953: 25), first description of female; Cortés and Hichins (1979: 115), redescription of female; Liljesthröm (1980: 135), first record from Argentina.

***insignis*** (van der Wulp, 1882).—Neotropical: South America (Argentina, Chile). **Comb. nov.**

*Masicerainsignis* van der Wulp, 1882: 85. Syntypes, 1 male and 1 unknown (abdomen missing at time of description) (RMNH). Type locality: Chile.

Note: *Masicerainsignis* was treated as a species of *Sturmia* Robineau-Desvoidy, 1830 by previous authors (e.g., Cortés and Hichins 1969: 59; Guimarães 1971: 192; Henry 1987: 200) but is moved here to *Drino* Robineau-Desvoidy (see note under genus for generic characters).

Reference: Cortés (1944g: 166), redescription, first record from Argentina.

***piceiventris*** (Walker, 1836).—Neotropical: South America (Chile).

*Tachinapiceiventris* Walker, 1836: 350. Lectotype female (NHMUK), by fixation of Aldrich (1934: 139) (examination of female “type” in NHMUK is regarded as a lectotype fixation). Type locality: not given; somewhere along the South American coast “from St. Paul’s [São Paulo, see Thompson (1974: 2)] in Brazil to Valparaiso [Chile]” according to Curtis (1836: 315), here interpreted as Chile based on the known distribution of the species.

Note: *Tachinapiceiventris* was treated as a species of *Sturmia* Robineau-Desvoidy, 1830 by most previous authors (e.g., Aldrich 1934: 139; Cortés 1944g: 161; Cortés 1963: 242; Cortés and Hichins 1969: 59; Henry 1987: 200) but was moved to *Drino* Robineau-Desvoidy by Guimarães (1971: 189).

References: Aldrich (1934: 139), redescription; Cortés (1944g: 161), redescription; Campos (1953: 24), partial redescription; Cortés (1963: 242), notes on name-bearing type in NHMUK.

#### Genus *LESPESIA* Robineau-Desvoidy, 1863

***LESPESIA*** Robineau-Desvoidy, 1863a: 567. Type species: *Achaetoneuraanisotae* Webber, 1930, by designation under the Plenary Powers of ICZN (1983: 97) [United States].

*ACHAETONEURA* Brauer & Bergenstamm, 1891: 334 [also 1891: 30]. Type species: *Achaetoneurahesperus* Brauer & Bergenstamm, 1891 (= *Masicerafrenchii* Williston, 1889), by subsequent designation of Townsend (1908: 88) [North America].

*PARAFRONTINA* Brauer & Bergenstamm, 1893: 27 [also 1893: 115]. Type species: *Parafrontinaapicalis* Brauer & Bergenstamm, 1893 (= *Tachinaarchippivora* Riley, 1871), by monotypy [United States].

*ZYGOFRONTINA* Townsend, 1915e: 427. Type species: *Zygofrontinacapitis* Townsend, 1915 (= *Tachinaarchippivora* Riley, 1871), by original designation [Peru].

*MASICEROPSIS* Townsend, 1916c: 178. Type species: *Masicerapauciseta* Coquillett, 1897 (= *Tachinaarchippivora* Riley, 1871), by original designation [United States].

*YPOPHAEMYIA* Townsend, 1916d: 75. Type species: *Ypophaemyiamalacosomae* Townsend, 1916 (= *Tachinaarchippivora* Riley, 1871), by original designation [United States].

*EUPARAFRONTINA* Brèthes, 1917: 17. Type species: *Euparafrontinamartinezi* Brèthes, 1917, by monotypy [Peru].

*PROPHRYNO* Townsend, 1927a: 262. Type species: *Prophrynoaurulans* Townsend, 1927 (= *Tachinalata* Wiedemann, 1830), by original designation [Brazil].

*ACHAETONEUROPSIS* Townsend, 1927a: 272. Type species: *Achaetoneuropsisaffinis* Townsend, 1927, by original designation [Brazil].

*MYIOSTURMIA* Townsend, 1927a: 272. Type species: *Myiosturmiamixta* Townsend, 1927, by original designation [Brazil].

*ZYGOFRONTINOPSIS* Blanchard, 1959: 173. Type species: *Zygofrontinopsiswilliamsoni* Blanchard, 1959, by original designation [Argentina].

*STURMIOPSOIDEA* Thompson, 1966: 359. Type species: *Sturmiopsoideaobscura* Thompson, 1966 (junior secondary homonym of *Eurigasterobscurus* Bigot, 1857; = *Lespesiathompsoni* O’Hara & Wood, **nom. nov.**, see below), by monotypy [Trinidad & Tobago]. **Syn. nov.**

*ACHATONEURA*. Incorrect subsequent spelling of *Achaetoneura* Brauer & Bergenstamm, 1891 (Townsend 1927a: 268 [not p. 230 as cited by Evenhuis et al. 2015: 38], subsequently corrected to *Achaetoneura* in Townsend 1927b, see entry for “page 268, line 7 [from] bottom” in the unpaginated errata of Townsend 1927a).

*ZYGOFRONTINIOPSIS*. Incorrect subsequent spelling of *Zygofrontinopsis* Blanchard, 1959 (Guimarães 1983: 14, etc.; Toma 2010: 166; Nihei 2016: 929).

Note: The new synonymy of *Sturmiopsoidea* with *Lespesia* is explained below under *Lespesiathompsoni* O’Hara & Wood, which is a new replacement name for type species *Sturmiopsoideaobscura* Thompson, 1966 (a junior homonym of *Eurigasterobscurus* Bigot, 1857 when the two names are placed together in *Lespesia*).

References: Coquillett (1910: 502, 545), type species of *Achaetoneura* and *Parafrontina* (both as synonyms of *Frontina* Meigen, 1838); Webber (1930: 1), synonymy of *Masiceropsis*, *Parafrontina* and *Ypophaemyia* with *Achaetoneura*, revision of North American species; Aldrich (1934: 4, 91), in key to Patagonian genera, synonymy of *Achaetoneuropsis*, *Euparafrontina* and *Zygofrontina* with *Achaetoneura*, taxonomic notes; Townsend (1936c: 190, 218, 227, 277), diagnosis of adults and immatures of Sturmiini and key to genera (including *Myiosturmia*), diagnosis of adults and immatures of Lydellini and key to genera (including *Masiceropsis*); diagnosis of adults and immatures of Phrynoini and key to genera (including *Achaetoneura*, *Achaetoneuropsis*, *Euparafrontina*, *Parafrontina*, *Prophryno*, *Ypophaemyia* and *Zygofrontina*), *Lespesia* as synonym of *Istocheta* Rondani, 1859; Townsend (1941: 109, 192, 203–227), redescriptions of *Myiosturmia*, *Masiceropsis*, and the aforementioned genera of Phrynoini; Mesnil (1950: 109), synonymy of *Achaetoneura* and *Prophryno* with *Lespesia*; Beneway (1963), revision of North American species, synonymy following non-Townsend authors; Thompson (1966: 371), revision of three Trinidad species, taxonomic notes; Cortés and Campos (1971: 25, 1974: 115) and Cortés (1984: 381), in keys to tachinid genera of Tarapacá and Antofagasta regions; Sabrosky (1980: 65), revised key to Nearctic species; Guimarães (1983: 12), synonymy of *Myiosturmia* and *Zygofrontinopsis* with *Lespesia*, revision of Brazilian species; Toma (2010), revision of Venezuelan species.

*archippivora* (Riley, 1871).—Not Chile [widespread throughout the Nearctic Region and most of Middle and South America].

*Tachinaarchippivora* Riley, 1871: 150 (name not authored by Williston as cited by some early authors).

Note: *Lespesiaarchippivora* was recorded from Chile by Molina-Ochoa et al. (2003: 262), citing Etcheverry (1957) as the source. There is no mention of *L.archippivora* in Etcheverry (1957) and the Chilean record of this species in Molina-Ochoa et al. (2003) is assumed to be in error.

***auriceps*** (Macquart, 1844).—Neotropical: South America. Distribution not known beyond the imprecise type locality of Brazil or Chile.

*Masiceraauriceps* Macquart, 1844: 59 [also 1844: 216]. Lectotype male (MNHN), by designation herein (see Lectotype Designations section). Type locality: Brazil or Chile.

References: Guimarães (1971: 209), as recognised species of *Lespesia*; Guimarães (1983: 23), as unrecognised species of *Lespesia*.

*modesta* (Bigot, 1857).—Not Chile [Cuba]. **Comb. nov.**

*Eurigastermodestus* Bigot, 1857b: 341. Type(s), unspecified sex (2 syntypes in MNHN, see note). Type locality: Cuba.

Note: The online MNHN database records two syntypes in the Guérin-Meneville in Macquart collection for *Eurigastermodestus*. One is a female based on the presence of proclinate orbital setae on the head (number MNHN-ED-ED10017) and the other is of undetermined sex (number MNHN-ED-ED10018, head missing). Bigot (1857b: 341) did not specify the sex of the name-bearing type but female is suggested by his comment under *Eurigasterobscurus* that this might be the male of *Eurigastermodestus*. The syntypes of *E.modestus* were examined by DMW and determined to belong to *Lespesia*.

Reference: Guimarães (1971: 215), as unplaced species of Exoristinae (as “Goniinae”).

*obscura* (Bigot, 1857).—Not Chile [Cuba]. **Comb. nov.**

*Eurigasterobscurus* Bigot, 1857b: 341. Type(s), male (1 male in MNHN, see note). Type locality: Cuba.

Note: The online MNHN database records a male holotype in the Guérin-Meneville in Macquart collection for *Eurigasterobscurus* (number MNHN-ED-ED10015) based on a holotype determination label that Paul Arnaud, Jr. attached to the specimen in 1972. However, Bigot did not restrict the name-bearing type to a single specimen and the “holotype” in MNHN is technically a syntype [see Recommendation 73F of the *Code* (ICZN 1999), “Avoidance of assumption of holotype”]. This specimen was examined by DMW and determined to be a species of *Lespesia* (see characters of the genus under *L.thompsoni*) This new combination is recorded here because the name is currently valid and a senior secondary homonym of *Sturmiopsoideaobscura* Thompson, 1966.

Reference: Guimarães (1971: 215), as unplaced species of Exoristinae (as “Goniinae”).

*thompsoni* O’Hara & Wood, **nom. nov.**—Not Chile [Trinidad].

*Sturmiopsoideaobscura* Thompson, 1966: 359 (junior secondary homonym of *Eurigasterobscurus* Bigot, 1857). Holotype male (CNC). Type locality: Trinidad, North Coast Road [as “American Road”], “Mauvan Hill” [not located]. **Comb. nov.**

*Lespesiathompsoni* O’Hara & Wood, **nom. nov.** for *Sturmiopsoideaobscura* Thompson, 1966.

Notes: *Sturmiopsoideaobscura* Thompson, 1966, from Trinidad, is a junior secondary homonym of *Eurigasterobscurus* Bigot, 1857, the valid name of a Cuban species that we transfer above to *Lespesia*. In the interests of nomenclatural stability, we hereby propose the new name *Lespesiathompsoni* to replace the name of the junior homonym *Sturmiopsoideaobscura*. The same type material applies to the new name. The specific epithet *thompsoni* is based on the surname of the describer of *S.obscura*, W.R. Thompson.

We have examined the holotype of *S.obscura* in CNC and it has the usual characteristics of Erycini and *Lespesia*, and runs to *Lespesia* in the keys of Wood (1987: 1211) and Wood and Zumbado (2010: 1363). Among the more diagnostic features of *Lespesia* are a setose facial ridge and four katepisternal setae. The eye can be haired or bare (haired in *S.obscura*) and this character splits *Lespesia* into two exit points in both of the aforementioned keys. Thompson (1966: 355) restricted *Lespesia* to species with a bare eye and an otherwise similar species (*obscura*) with a haired eye was assigned to new genus *Sturmiopsoidea*.

Reference: Guimarães (1971: 192), as *Sturmiopsoideaobscura*.

##### *Nomen dubium* of *LESPESIA* Robineau-Desvoidy, 1863

*andina* (Bigot, 1888).—Not Chile [Cuba]. **Comb. nov.**

*Blepharipezaandina* Bigot, 1888b: 90. Holotype male (NHMUK). Type locality: Cuba (as “Chili” in error, see note).

Note: The holotype of *Blepharipezaandina* in NHMUK was examined by DMW. It is a male (published as “♂?”) of *Lespesia*, possibly near *L.aletiae* (Riley, 1879). The label indicates that it is from Cuba, not Chile as published and as subsequently interpreted. It is moved here to *Lespesia* as a *nomen dubium* from various uncertain placements (see references below).

References: Cortés (1946: 184), listed under “Species *incertae sedis*” at end of Tachinidae; Cortés and Hichins (1969: 90), listed under “Especies excluidas de la lista (*incertae sedis*)”; Guimarães (1971: 193), listed as an unplaced species of Sturmiini.

#### Genus *RCORTESIA* Koçak & Kemal, 2010

*HYPSOMYIA* Cortés, 1984: 382 (junior homonym of *Hypsomyia* McAlpine, 1965). Type species: *Hypsomyiahispida* Cortés, 1983, by original designation [Chile].

***RCORTESIA*** Koçak & Kemal, 2010: 159 (*nomen novum* for *Hypsomyia* Cortés, 1983).

Reference: Cortés (1984: 380), in key to tachinid genera of Tarapacá and Antofagasta regions.

***hispida*** (Cortés, 1983).—Neotropical: South America (Chile).

*Hypsomyiahispida* Cortés, 1984: 383. Holotype male (MEUC). Type locality: Chile, Arica y Parinacota, Arica, Las Cuevas, Parque Nacional Lauca, 4800 m.

#### Genus *TELONOTOMYIA* Cortés, 1986

***TELONOTOMYIA*** Cortés, 1986: 151. Type species: *Telonotomyiaremota* Cortés, 1986, by original designation [Chile].

Reference: Cortés (1986: 143), in key to tachinid genera of Aysén and Magallanes regions.

***remota*** Cortés, 1986.—Neotropical: South America (Chile).

*Telonotomyiaremota* Cortés, 1986: 152. Holotype male (MEUC). Type locality: Chile, Magallanes y de la Antártica Chilena, Magallanes, Río Seco [as “Los Robles”, ca. 53°5′S, 70°53′W].

#### Unplaced species of Eryciini

The genus *Phorocera* Robineau-Desvoidy, 1830 *sensu*Aldrich (1934: 69) and Cortés (1945d: 158, 1950: 7) was cosmopolitan in distribution and consisted of many species. The main characters were given by Aldrich (1934: 69) as “hairy eyes, receding face, and bristly facial ridges”. None of the six Chilean species described in *Phorocera* by Aldrich and Cortés belong in the genus as defined by Wood (1972). We are only able to place one of these species to genus (*Phoroceracalyptrata* Aldrich, 1934 is a species of *Admontia* Brauer & Bergenstamm) and the others are left unplaced to genus (two here in Eryciini, one in Goniini, and one in Winthemiini) or subfamily (one unplaced species of Tachinidae).

**Figure 5.**
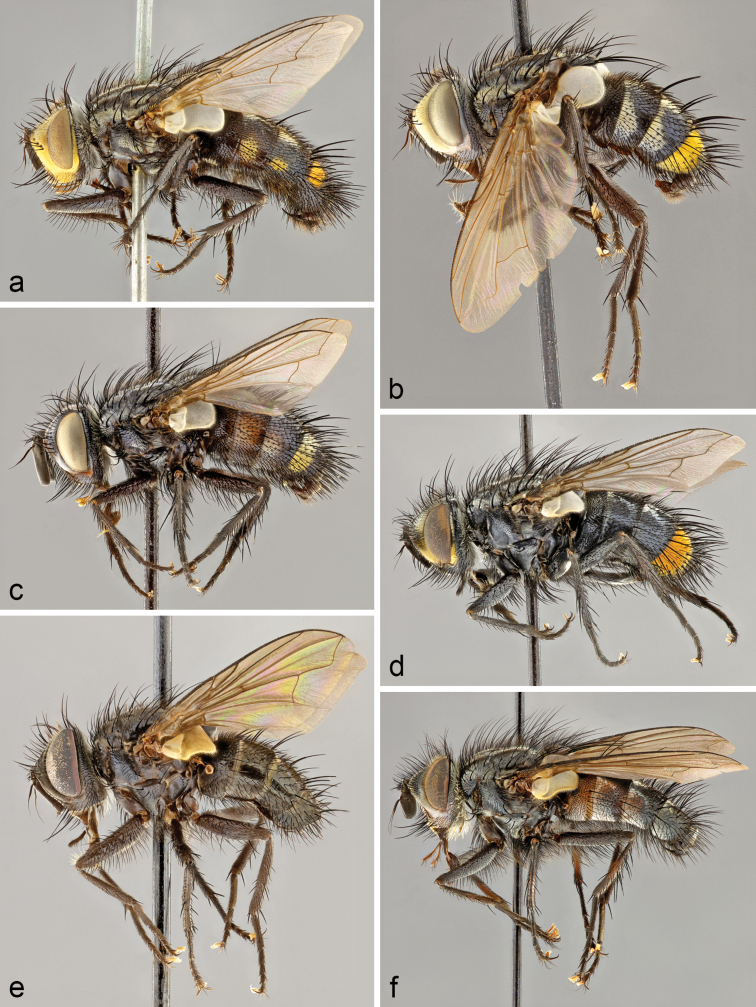
Habitus images **a***Chetogenaporteri* (Brèthes), comb. nov. ♂ (Exoristinae, Exoristini) (Chile) [CNC1546960], 11.0 mm **b** “*Phorocerachilensis* Cortés” ♂ (Exoristinae, unplaced species of Eryciini) (Chile) [CNC1546961], 6.7 mm **c** “*Phoroceraelisae* Cortés” ♂ (Exoristinae, unplaced species of Eryciini) (Chile) [CNC1143104], 8.3 mm **d***Patelloatanumeana* (Townsend), comb. nov. ♂ (Exoristinae, Goniini) (Chile) [CNC487488], 10.9 mm **e** “*Phoroceranegrensis* Aldrich” ♂ (Exoristinae, unplaced species of Goniini) (Argentina) [CNC1546962], 4.9 mm **f** “*Phorocerabullocki* Aldrich” ♂ (Exoristinae, unplaced species of Winthemiini) (Chile) [CNC1143251], 12.5 mm.

***chilensis*** Cortés, 1950.—Neotropical: South America (Chile). (Fig. 5b)

*Phorocerachilensis* Cortés, 1950: 7. Holotype male (INLA). Type locality: Chile, Coquimbo, Elqui, Gualliguaica.

Note: Cortés and Hichins (1969: 27) placed *Phorocerachilensis* in the Old World genus *Clemelis* Robineau-Desvoidy, 1863 (Goniini) and this was followed by Henry (1987: 196) and González (1992b: 183). We follow Guimarães (1971: 214) in treating this species as unplaced in the Eryciini.

Reference: Cortés (1950: 10), in key to Chilean species of *Phorocera* Robineau-Desvoidy, 1830 (*s. lato*).

***elisae*** Cortés, 1945.—Neotropical: South America (Chile). (Fig. 5c)

*Phoroceraelisae* Cortés, 1945d: 162. Holotype female (USNM). Type locality: Chile, Araucanía, Malleco, Angol.

Note: Guimarães (1971: 161) listed *Phoroceraelisae* as an unrecognised species of Exoristini. We treat it as a recognised but unplaced species of Eryciini based on the examination of the holotype by DMW.

References: Cortés (1945d: 159), in key to Chilean species of *Phorocera* Robineau-Desvoidy, 1830 (*s. lato*) and *Parasetigena* Brauer & Bergenstamm, 1891; Cortés (1950: 10), in key to Chilean species of *Phorocera* (*s. lato*); Cortés (1951a: 65), first description of male; Henry (1987: 206), in *Phorocera* but genus unplaced in Tachinidae.

### Tribe ETHILLINI

#### Genus *NEOETHILLA* Cerretti, Wood & O’Hara, 2012

*NEOETHILLA* Cerretti, Wood & O’Hara, 2012: 28. Type species: *Exoristaignobilis* van der Wulp, 1890, by original designation [Mexico].

*ignobilis* (van der Wulp, 1890).—Not Chile [Mexico, United States].

*Exoristaignobilis* van der Wulp, 1890b: 71.

Note: *Exoristaignobilis* was assigned to *Winthemia* Robineau-Desvoidy by Reinhard (1931: 16) and stayed in this genus until recently recognised as the sole New World member of the Ethillini and placed in the new genus *Neoethilla* by Cerretti et al. (2012). Its distribution is thought to be limited to United States and Mexico (Cerretti et al. 2012) and reports of the species from South America in the following works are likely based on misidentifications of one or more *Winthemia* species: Reinhard (1931: 17, Argentina, Chile), Aldrich (1934: 44, Argentina, Chile), Cortés (1946: 175, Chile), Cortés (1948: 124, Chile), Cortés and Hichins (1969: 63, Chile), Henry (1987: 200, Chile), Coelho et al. (1989: 275, as “*ignobillis*”, Argentina, Bolivia, Brazil, Chile, Colombia, Equador, Peru, Venezuela), Vergara de Sánchez and Raven (1990: 100, Chile) and González (1992b: 180, 183, Chile). Cerretti et al. (2012) suspected that the species called *W.ignobilis* in Chile and Argentina might be *Winthemiareliqua* Cortés & Campos, 1971, which Coelho et al. (1989: 281) has treated as a synonym of *Winthemiatrinitatis* Thompson, 1963.

### Tribe EXORISTINI

#### Genus *CHETOGENA* Rondani, 1856

*SALIA* Robineau-Desvoidy, 1830: 108 (junior homonym of *Salia* Hübner, 1818). Type species: *Saliaechinura* Robineau-Desvoidy, 1830 (= *Tachinaobliquata* Fallén, 1810), by subsequent designation of Robineau-Desvoidy (1863a: 553) [France].

***CHETOGENA*** Rondani, 1856: 68. Type species: *Saliarondaniana* Villeneuve, 1931, by fixation of O’Hara and Wood (2004: 145) under Article 70.3.2 of the *Code* (ICZN 1999), misidentified as *Tachinagramma* Meigen, 1824 in the original designation by Rondani (1856) (see O’Hara et al. 2011: 54) [France].

*SPOGGOSIA* Rondani, 1859: 182. Type species: *Spoggosiaocclusa* Rondani, 1859 (= *Tachinaobliquata* Fallén, 1810), by monotypy [Italy and Malta].

*STOMATOMYIA* Brauer & Bergenstamm, 1889: 98 [also 1890: 30]. Type species: *Chetogenafilipalpis* Rondani, 1859, by subsequent designation of Brauer (1893: 483) [Italy].

*TETRAGRAPHA* Brauer & Bergenstamm, 1891: 351 [also 1891: 47]. Type species: *Tetragraphatessellata* Brauer & Bergenstamm, 1891, by monotypy [Cuba].

*EUPHOROCERA* Townsend, 1892b: 112. Type species: *Euphoroceratachinomoides* Townsend, 1892, by original designation [United States].

*PLAGIPROSPHERYSA* Townsend, 1892b: 113. Type species: *Plagiprospherysavalida* Townsend, 1892 (= *Prospherysaparvipalpis* van der Wulp, 1890), by original designation [United States].

*TACHINOPSIS* Coquillett, 1897: 38, 120. Type species: *Tachinopsismentalis* Coquillett, 1897 (= *Prospherysaparvipalpis* van der Wulp, 1890), by original designation [United States].

*CHAETOGENA* Bezzi & Stein, 1907: 315. Unjustified emendation of *Chetogena* Rondani, 1856 (see O’Hara et al. 2011: 54, 259).

*PLAGIOTACHINA* Townsend, 1927a: 261. Type species: *Plagiotachinaperuviana* Townsend, 1927 (junior secondary homonym of *Euphoroceraperuviana* Townsend, 1912; = *Euphoroceratownsendi* Guimarães, 1971), by original designation [Peru].

*STOMATOTACHINA* Townsend, 1931d: 464. Type species: *Stomatotachinasplendida* Townsend, 1931 (= *Parasetigenaporteri* Brèthes, 1920), by original designation [Chile]. **Syn. nov.**

*EPIPLAGIOPS* Blanchard, 1943a: 450. Type species: *Epiplagiopslittoralis* Blanchard, 1943 (junior secondary homonym of *Plagiopslittoralis* Townsend, 1911; = *Plagiprospherysafloridensis* Townsend, 1892), by original designation [Argentina].

Note: *Parasetigena* Brauer & Bergenstamm, 1891 is an Old World genus with four species and a native distribution throughout the Palaearctic Region and northern portion of the Oriental Region (southern China). One species, *P.silvestris* (Robineau-Desvoidy, 1863), was successfully introduced into eastern North America for biological control purposes and has become established. The assignment of South American species to this Old World genus have been the result of a misunderstanding of the difference between *Parasetigena* and *Chetogena* Rondani. The latter currently has 71 species and is worldwide in distribution (O’Hara et al. 2020: 385). The two genera have the typical features of the Exoristini (principally prosternum haired, first postsutural supra-alar seta short and bend of vein M_1_ right-angled) and a setose facial ridge, but in *Parasetigena* the setae on the facial ridge are weak and decumbent and in *Chetogena* they are strong and erect (see Wood 1987: 1209, cf. head figs 35 [*Chetogenatachinomoides* (Townsend, 1892)] and 36 [*Exoristalarvarum* (Linnaeus, 1758), illustrative of *Parasetigena*] and pp. 1220–1221, key couplets 107–111). Based on this interpretation of *Chetogena*, we transfer two Chilean species originally described as *Parasetigenaporteri* Brèthes (currently in *Stomatotachina* Townsend with type species of that genus in synonymy) and *Parasetigenahichinsi* Cortés to *Chetogena*.

References: Coquillett (1910: 522, 542, 589, 591, 602, 608, 611, 613), type species of *Chetogena*, *Euphorocera*, *Salia*, *Spoggosia* (all four as synonyms of *Phorocera* Robineau-Desvoidy, 1830), *Plagiprospherysa*, *Tachinopsis* and *Tetragrapha*; Aldrich (1926a: 14), synonymy of *Tachinopsis* with *Plagiprospherysa*; Aldrich (1934: 3, 4, 34, 69), in key to Patagonian genera, synonymy, taxonomic notes (as *Plagiprospherysa* and *Phorocera* [in part]); Townsend (1936c: 116, 123, 273, 281), diagnosis of adults and immatures of Exoristini and key to genera (including *Euphorocera*, *Plagiotachina*, *Spoggosia* and *Tetragrapha*), diagnosis of adults and immatures of Phoriniini and key to genera (including *Plagiprospherysa*, *Stomatomyia*, *Stomatotachina* and *Tachinopsis*), *Chetogena* as synonym of *Phorocera*, *Spoggosia* as valid name for *Salia*; Townsend (1940a: 160, 167, 170, 171, 182, 184, 185, 186), redescriptions of *Euphorocera*, *Plagiotachina*, *Spoggosia* (with *Salia* in synonymy), *Tetragrapha*, *Plagiprospherysa*, *Stomatomyia*, *Stomatotachina* and *Tachinopsis*; Mesnil (1946: 42), synonymy of *Plagiotachina* with *Euphorocera*; Cortés and Campos (1971: 23, 24, 1974: 113, 114) and Cortés (1984: 379, 380), in keys to tachinid genera of Tarapacá and Antofagasta regions (as *Plagiprospherysa* and *Euphorocera*); Cortés (1986: 144), in key to tachinid genera of Aysén and Magallanes regions (as *Plagiprospherysa*); Wood (1987: 1221), synonymy of *Euphorocera*, *Spoggosia* and *Stomatomyia* with *Chetogena*; O’Hara and Wood (1998: 755, 759), review of synonymy of Wood (1987) (*Spoggosia* and *Stomatomyia* overlooked); Nihei (2015: 1, 2), synonymy of the monotypic genera *Epiplagiops* and *Tetragrapha* with *Chetogena*.

***hichinsi*** (Cortés, 1967).—Neotropical: South America (Chile). **Comb. nov.** (Fig. 4f)

*Parasetigenahichinsi* Cortés, 1967b: 13. Holotype male (EEAM). Type locality: Chile, Metropolitana de Santiago, Santiago, Maipú, Rinconada.

Note: We examined specimens of *P.hichinsi* in CNC and have determined that it belongs to *Chetogena* according to the criteria given above in genus note.

References: Guimarães (1971: 159), as sole species of *Parasetigena* Brauer & Bergenstamm in America south of United States; Henry (1987: 199), in *Parasetigena*.

***parvipalpis*** (van der Wulp, 1890).—Neotropical: Middle America (Mexico), South America (Argentina, Chile). Nearctic: Canada, United States.

*Prospherysaparvipalpis* van der Wulp, 1890d: 124. Syntypes, 3 males and 1 female (NHMUK). Type localities: Mexico, northern Sonora, Guerrero (Tepetlapa [ca. 18°3′N, 99°10′W], 3000 ft; Omiltemi [as “Omilteme”, ca. 17°33′N, 99°41′W], 8000 ft), and Sinaloa (Villa Unión [as “Presidio”, ca. 23°11′N, 106°13′W]).

*Plagiprospherysavalida* Townsend, 1892b: 113. Holotype male (SEMC, Byers et al. 1962: 176). Type locality: USA, New Mexico, Las Cruces.

*Tachinopsismentalis* Coquillett, 1897: 120. Holotype male (USNM). Type locality: USA, Washington [state].

References: Aldrich (1934: 34), synonymy, redescription, taxonomic notes, first record from Argentina (as “Southern Patagonia”, which is interpreted here as Argentina based on the travels of the collector, paleontologist Barnum Brown); Cortés and Hichins (1969: 53), first record from Chile.

***peruviana*** (Townsend, 1912).—Neotropical: South America (Chile, Peru).

*Euphoroceraperuviana* Townsend, 1912b: 303. Holotype female (USNM). Type locality: Peru, Piura, Piura.

Reference: Cortés and Campos (1971: 81), first record from Chile.

***porteri*** (Brèthes, 1920).—Neotropical: South America (Chile). **Comb. nov.** (Fig. 5a)

*Parasetigenaporteri* Brèthes, 1920b: 12. Lectotype, unspecified sex [female according to Mulieri et al. 2013: 169] (MACN), by fixation of Cortés (1963: 251) (examination of “Type” from Santiago in MACN is regarded as a lectotype fixation). Type locality: Chile, Metropolitana de Santiago, Santiago, Santiago.

*Stomatotachinasplendida* Townsend, 1931d: 464. Holotype female (SDEI, Rohlfien and Ewald 1974: 144). Type locality: Chile, Biobío, Concepción, Concepción. **Comb. nov.**

Notes: We examined specimens of *P.porteri* in CNC, including a male identified by R. Cortés, and have determined that it belongs to *Chetogena* according to the criteria given above in genus note.

The type locality of *Parasetigenaporteri* was given as “Santiago” and that of *Stomatotachinasplendida* as “Concepcion”, both of which could be interpreted as either the city or province of those names. Cortés and Hichins (1969: 46) cited the cities as the type localities, as “Santiago (Santiago)” and “Concepción (Concepción)”, and we follow this interpretation.

References: Cortés (1945d: 158, 159), in key to Chilean species of *Phorocera* Robineau-Desvoidy, 1830 (*s. lato*) and *Parasetigena* Brauer & Bergenstamm, 1891, first description of male, synonymy of *Stomatotachinasplendida* with *Parasetigenaporteri*, synonymy of *Stomatotachina* with *Parasetigena*; Cortés (1963: 251), notes on name-bearing type of *P.porteri* in MACN; Guimarães (1971: 160), *Stomatotachina* revived as valid genus name for *P.porteri*; Mulieri et al. (2013: 169), notes on name-bearing type (as syntype) of *P.porteri* in MACN, as *Stomatotachinaporteri*.

### Tribe GONIINI

Some of the genera recognised as valid below are almost certainly synonymous with the widespread New World genus *Spallanzania* Robineau-Desvoidy, 1830. Wood and Zumbado (2010: 1412) noted: “*Spallanzania* has six North American species and ca. 20 nominal species (presently assigned to nearly as many genera) at high elevations in the Andes and at high latitudes in Patagonia”. We hesitate to formally propose any synonymy here because the diversity of morphological forms in the *Spallanzania* lineage is best left for a more detailed study before the limits of the genus are revised. Chilean genera to be considered in such a study include *Chaetocnephalia* Townsend, *Chaetocraniopsis* Townsend *Coscaronia* Cortés and *Dolichocnephalia* Townsend.

Reference: González and Vergés (2004), revision of ten (of the 14) genera of Chilean Goniini (not included were *Belvosia* Robineau-Desvoidy, *Leschenaultia* Robineau-Desvoidy, *Patelloa* Townsend [formerly as *Macropatelloa* Townsend] and *Pseudochaeta* Coquillett).

#### Genus *ARAUCOGONIA* Cortés, 1976

***ARAUCOGONIA*** Cortés, 1976: 10. Type species: *Araucogoniaspeciosa* Cortés, 1976, by original designation [Chile].

References: Cortés (1986: 144), in key to tachinid genera of Aysén and Magallanes regions; González and Vergés (2004: 41, 42), in key to Chilean genera of Goniini, redescription.

***speciosa*** Cortés, 1976.—Neotropical: South America (Chile).

*Araucogoniaspeciosa* Cortés, 1976: 11. Holotype male (MEUC). Type locality: Chile, Araucanía, Malleco, Pehuenco Chico, Marimenuco, 1000 m [ca. 38°43′S, 71°7′W].

Reference: González and Vergés (2004: 42), redescription.

#### Genus *ARAUCOSIMUS* Aldrich, 1934

***ARAUCOSIMUS*** Aldrich, 1934: 88. Type species: *Araucosimusbullocki* Aldrich, 1934, by original designation [Chile].

References: Townsend (1936c: 169), diagnosis of adults and immatures of Goniini and key to genera (including *Araucosimus*); Townsend (1941: 10), redescription; Cortés and Campos (1971: 22, 1974: 113) and Cortés (1984: 379), in keys to tachinid genera of Tarapacá and Antofagasta regions; González and Vergés (2004: 41, 43), in key to Chilean genera of Goniini, redescription, key to species.

***bullocki*** Aldrich, 1934.—Neotropical: South America (Argentina, Chile).

*Araucosimusbullocki* Aldrich, 1934: 88. Holotype male (USNM). Type locality: Chile, Araucanía, Malleco, Angol.

References: Cortés (1979: 76), taxonomic notes, first record from Argentina; González and Vergés (2004: 43), redescription.

***orfilanus*** Cortés, 1979.—Neotropical: South America (Argentina, ?Chile).

*Araucosimusorfilanus* Cortés, 1979: 76. Holotype male (MLPA). Type locality: Argentina, Mendoza, Mendoza.

Note: Cortés (1979: 76) described *Araucosimusorfilanus* from Argentina but also identified a female from Chile (from El Melocotón near Santiago) as tentatively belonging to this species.

Reference: González and Vergés (2004: 44), redescription, tentatively recorded from Chile.

***superbus*** Cortés, 1945.—Neotropical: South America (Chile).

*Araucosimussuperbus* Cortés, 1945a: 122. Holotype male (USNM). Type locality: Chile, Valparaíso, Marga Marga, Bosque Los Perales [as “Perales, prov. de Valparaíso”, ca. 33°9′S, 71°18′W].

References: Campos (1953: 26), first description of female; González and Vergés (2004: 44), redescription.

#### Genus *BELVOSIA* Robineau-Desvoidy, 1830

***BELVOSIA*** Robineau-Desvoidy, 1830: 103. Type species: *Belvosiabicincta* Robineau-Desvoidy, 1830, by monotypy [Unites States and West Indies].

*LATREILLIA* Robineau-Desvoidy, 1830: 104 (junior homonym of *Latreillia* Roux, 1830; priority established by ruling of ICZN 1964: 343, see Evenhuis et al. 2010: 96). Type species: *Muscabifasciata* Fabricius, 1775, by subsequent designation of Coquillett (1910: 558) (see Evenhuis et al. 2010: 96) [America, probably West Indies].

*WILLISTONIA* Brauer & Bergenstamm, 1889: 97 [also 1890: 29]. Type species: hereby fixed under Article 70.3.2 of the *Code* (ICZN 1999) as *Willistoniaaldrichi* Townsend, 1931, misidentified as *Muscaesuriens* Fabricius, 1805 in the fixation by monotypy of Brauer and Bergenstamm (1889) [Brazil].

*LATREILLIMYIA* Townsend, 1908: 105 (*nomen novum* for *Latreillia* Robineau-Desvoidy, 1830).

*GONIOMIMA* Townsend, 1908: 105. Type species: *Belvosialuteola* Coquillett, 1900, by monotypy [Puerto Rico].

*TRIACHORA* Townsend, 1908: 105. Type species: *Latreilliaunifasciata* Robineau-Desvoidy, 1830, by monotypy [America, probably West Indies].

*BELVOSIOMIMA* Townsend, 1915e: 413. Type species: *Belvosiomimafosteri* Townsend, 1915, by original designation [Paraguay].

*BELVOSIOPSIS* Townsend, 1927a: 248. Type species: *Belvosiopsisbrasiliensis* Townsend, 1927 (= *Belvosiaweyenberghiana* van der Wulp, 1883), by original designation [Brazil].

*PSEUDOBELVOSIA* Blanchard, 1954: 8. Type species: *Pseudobelvosialugubris* Blanchard, 1954, by original designation [Argentina].

*PARABELVOSIA* Blanchard, 1954: 12. Type species: *Parabelvosiatibialis* Blanchard, 1954, by original designation [Argentina].

*EUBELVOSIOPSIS* Blanchard, 1954: 15. Type species: *Eubelvosiopsisformosana* Blanchard, 1954, by original designation [Argentina].

*NEOBELVOSIOPSIS* Blanchard, 1954: 20. Type species: *Neobelvosiopsisbosqi* Blanchard, 1954, by original designation [Argentina].

References: Williston (1893: 240), synonymy of *Latreillia* and *Willistonia* with *Belvosia*; Coquillett (1910: 513, 547, 558, 615, 619), type species of *Belvosia*, *Goniomima*, *Latreillia*, *Latreillimyia*, *Triachora* and *Willistonia* (with *Latreillia*, *Latreillimyia* and *Willistonia* in synonymy with *Belvosia*, and *Triachora* in synonymy with *Goniomima*); Aldrich (1928a: 1), synonymy of *Belvosiomima*, *Belvosiopsis*, *Goniomima*, *Latreillimyia* and *Triachora* with *Belvosia*; Townsend (1936c: 180, 277), diagnosis of adults and immatures of Belvosiini and key to genera (including *Belvosia*, *Belvosiomima*, *Belvosiopsis*, *Goniomima*, *Latreillimyia*, *Triachora* and *Willistonia*), *Latreillimyia* as valid name for *Latreillia*; Townsend (1941: 57, 58, 60, 66, 67, 74, 76), redescriptions of *Belvosia*, *Belvosiomima*, *Belvosiopsis*, *Goniomima*, *Latreillimyia* (with *Latreillia* in synonymy), *Triachora* and *Willistonia*; Cortés and Campos (1971: 27, 1974: 116) and (1984: 382), *Triachora* in keys to tachinid genera of Tarapacá and Antofagasta regions; Guimarães (1971: 181), synonymy of *Eubelvosiopsis*, *Neobelvosiopsis*, *Parabelvosia* and *Pseudobelvosia* with *Belvosia*; Wood (1987: 1214), synonymy of *Triachora* with *Belvosia*; O’Hara and Wood (1998: 757, 759), review of synonymy of Wood (1987).

***barbosai*** (Cortés & Campos, 1971).—Neotropical: South America (Chile).

*Triachorabarbosai* Cortés & Campos, 1971: 98. Holotype female (EEAM). Type locality: Chile, Arica y Parinacota, Arica, Codpa, 2109 m (18°50′S, 69°47′W) (coordinates and elevation given on p. 10; longitude given as “70°47′W”, a location in the Pacific Ocean and likely an error for 69°47′W).

Reference: Cortés and Campos (1974: 123), first description of male.

#### Genus *CHAETOCNEPHALIA* Townsend, 1915

***CHAETOCNEPHALIA*** Townsend, 1915d: 63. Type species: *Chaetocnephaliaalpina* Townsend, 1915, by original designation [Peru].

References: Aldrich (1934: 4, 89), in key to Patagonian genera, taxonomic notes; Townsend (1936c: 169), diagnosis of adults and immatures of Goniini and key to genera (including *Chaetocnephalia*); Townsend (1941: 13), redescription; Cortés and Campos (1971: 22, 1974: 113) and Cortés (1984: 379), in keys to tachinid genera of Tarapacá and Antofagasta regions; Cortés (1986: 144), in key to tachinid genera of Aysén and Magallanes regions; González and Vergés (2004: 41, 45), in key to Chilean genera of Goniini, redescription, key to species.

***americana*** (Schiner, 1868).—Neotropical: South America (Argentina, Chile).

*Cnephaliaamericana* Schiner, 1868: 327. Holotype female (NHMW). Type locality: Chile.

References: Aldrich (1927b: 31), redescription of holotype; Aldrich (1934: 89), redescription; Cortés (1979: 78), first record from Argentina; González and Vergés (2004: 46), redescription.

***andina*** Cortés & Campos, 1971.—Neotropical: South America (Argentina, Bolivia, Chile).

*Chaetocnephaliaandina* Cortés & Campos, 1971: 76. Holotype male (EEAM). Type locality: Chile, Antofagasta, El Loa, Ojo Hécar, 4500 m (23°11′S, 68°01′W) (coordinates and elevation given on p. 12, locality as “Ojo Hécar (Láscar)”).

References: Cortés (1980: 107), first records from Argentina and Bolivia; González and Vergés (2004: 46), redescription.

***cortesi*** González, 2004.—Neotropical: South America (Chile).

*Chaetocnephaliacortesi* González in González and Vergés, 2004: 47. Holotype male (UMCE). Type locality: Chile, Tarapacá, Tamarugal, Mamiña, 2800 m [ca. 20°4′S, 69°13′W].

***innupta*** Cortés, 1945.—Neotropical: South America (Argentina, Chile).

*Chaetocnephaliainnupta* Cortés, 1945a: 120. Holotype female (USNM). Type locality: Chile, Metropolitana de Santiago, Santiago, Las Condes.

References: Campos (1953: 26), first description of male; Gramajo (1998: 97), first record from Argentina; González and Vergés (2004: 48), redescription.

#### Genus *CHAETOCRANIOPSIS* Townsend, 1915

***CHAETOCRANIOPSIS*** Townsend, 1915d: 68. Type species: *Chaetocraniopsischilensis* Townsend, 1915, by original designation [Chile].

*VALPOGONIA* Townsend, 1928b: 163. Type species: *Valpogoniachilensis* Townsend, 1928 (junior secondary homonym of *Chaetocraniopsischilensis* Townsend, 1915; = *Chaetocraniopsisargenticeps* Aldrich, 1928), by original designation [Chile].

References: Aldrich (1928b: 19), taxonomic notes on *Chaetocraniopsis*, key to two species; Townsend (1936c: 169), diagnosis of adults and immatures of Goniini and key to genera (including *Chaetocraniopsis* and *Valpogonia*); Townsend (1941: 14, 51), redescriptions of *Chaetocraniopsis* and *Valpogonia*; Cortés (1945a: 116), synonymy of *Valpogonia* with *Chaetocraniopsis*, key to Chilean species; Cortés (1980: 107), description of a species from Argentina (*C.transandinum* Cortés); Cortés and Campos (1971: 22, 1974: 113) and Cortés (1984: 379), in keys to tachinid genera of Tarapacá and Antofagasta regions; Cortés (1986: 144), in key to tachinid genera of Aysén and Magallanes regions; González and Vergés (2004: 41, 49), in key to Chilean genera of Goniini, redescription, key to species.

***argenticeps*** Aldrich, 1928.—Neotropical: South America (Argentina, Chile).

*Chaetocraniopsisargenticeps* Aldrich, 1928b: 20. Holotype male (USNM). Type locality: Chile, Valparaíso, Marga Marga, Bosque Los Perales [as “Perales”, ca. 33°9′S, 71°18′W].

*Valpogoniachilensis* Townsend, 1928b: 163 (junior secondary homonym of *Chaetocraniopsischilensis* Townsend, 1915). Holotype female (USNM). Type locality: Chile, Valparaíso, Valparaíso, “hills back of Valparaíso”.

Note: *Valpogoniachilensis* Townsend (published in “early 1928” according to Evenhuis et al. 2015: 352) has priority over *Chaetocraniopsisargenticeps* Aldrich (published on 1 December 1928 according to the Table of Contents of the journal volume) when the two are treated as synonyms, but *Valpogoniachilensis* is a junior secondary homonym of *Chaetocraniopsischilensis* Townsend, 1915 and thus invalid.

References: Cortés (1945a: 119), synonymy, taxonomic notes; Cortés (1980: 107), first record from Argentina; González and Vergés (2004: 50), redescription; Stireman et al. (2016: 38), habitus images.

***chilensis*** Townsend, 1915.—Neotropical: South America (Chile).

*Chaetocraniopsischilensis* Townsend, 1915d: 69. Holotype male (USNM). Type locality: Chile.

References: Aldrich (1928b: 20), taxonomic notes; Cortés (1945a: 117), taxonomic notes; González and Vergés (2004: 50), redescription.

***obliteratus*** Cortés, 1945.—Neotropical: South America (Chile).

*Chaetocraniopsisobliteratus* Cortés, 1945a: 117. Holotype male (USNM). Type locality: Chile, Valparaíso, Marga Marga, Bosque Los Perales [as “Perales, prov. de Valparaíso”, ca. 33°9′S, 71°18′W].

Reference: González and Vergés (2004: 51), redescription.

***similis*** (Townsend, 1928).—Neotropical: South America (Chile).

*Valpogoniasimilis* Townsend, 1928b: 163. Holotype female (USNM). Type locality: Chile, Valparaíso, Valparaíso, “hills back of Valparaíso”.

References: Cortés (1945a: 120), first description of male; González and Vergés (2004: 52), redescription.

#### Genus *COSCARONIA* Cortés, 1979

***COSCARONIA*** Cortés, 1979: 77. Type species: *Coscaroniaatrogonia* Cortés, 1979, by original designation [Argentina].

*COSCARCONIA*. Incorrect original spelling of *Coscaronia* Cortés, 1979 (Cortés 1979: 78, see note).

Note: There are two original spellings for *Coscaronia* in Cortés (1979): *Coscaronia* (pp. 77, 78) and *Coscarconia* (p. 78). There is clear evidence in the work itself that the spelling *Coscarconia* is an inadvertent error because the genus-group name is dedicated to dipterist S. Coscarón. Therefore, the spelling *Coscaronia* is deemed to be the correct original spelling (Article 32.5.1 of the *Code*, ICZN 1999).

References: Cortés (1986: 144), in key to tachinid genera of Aysén and Magallanes regions; González and Vergés (2004: 41, 52), in key to Chilean genera of Goniini, redescription.

***antennalis*** Cortés, 1986.—Neotropical: South America (Chile).

*Coscaroniaantennalis* Cortés, 1986: 157. Holotype male (MEUC). Type locality: Chile, Magallanes y de la Antártica Chilena, Tierra del Fuego, Isla Grande de Tierra del Fuego, Puerto Espora.

Reference: González and Vergés (2004: 53), redescription.

***propinqua*** Cortés, 1979.—Neotropical: South America (Argentina, Chile).

*Coscaroniapropinqua* Cortés, 1979: 78. Holotype male (MEUC). Type locality: Chile, Aysén, General Carrera, Chile Chico.

References: Gramajo (1998: 97), first record from Argentina; González and Vergés (2004: 53), redescription.

#### Genus *DOLICHOCNEPHALIA* Townsend, 1915

***DOLICHOCNEPHALIA*** Townsend, 1915d: 64. Type species: *Dolichocnephaliapuna* Townsend, 1915, by original designation [Peru].

References: Townsend (1936c: 169), diagnosis of adults and immatures of Goniini and key to genera (including *Dolichocnephalia*); Townsend (1941: 21), redescription; Cortés and Campos (1971: 21, 1974: 113) and Cortés (1984: 379), in keys to tachinid genera of Tarapacá and Antofagasta regions; González and Vergés (2004: 41, 53), in key to Chilean genera of Goniini, redescription.

***puna*** Townsend, 1915.—Neotropical: South America (Chile, Peru).

*Dolichocnephaliapuna* Townsend, 1915d: 66. Holotype female (USNM). Type locality: Peru, Junín, La Oroya, Valle del Río Mantaro, higher than 12,000 ft.

References: Cortés and Campos (1971: 80), first record from Chile; González and Vergés (2004: 53), redescription.

#### Genus *ENCHOMYIA* Aldrich, 1934

***ENCHOMYIA*** Aldrich, 1934: 42. Type species: *Goniaerythrocera* Bigot, 1888, by original designation [Chile].

References: Townsend (1936c: 169), diagnosis of adults and immatures of Goniini and key to genera (including *Enchomyia*); Townsend (1941: 23), redescription; Cortés (1986: 144), in key to tachinid genera of Aysén and Magallanes regions; González and Vergés (2004: 41, 54), in key to Chilean genera of Goniini, redescription.

***erythrocera*** (Bigot, 1888).—Neotropical: South America (Chile).

*Goniaerythrocera* Bigot, 1888b: 86. Holotype female (NHMUK). Type locality: Chile.

References: Aldrich (1934: 42), diagnosis, taxonomic notes; Cortés (1951a: 60), first description of male; González and Vergés (2004: 54), redescription.

***shewelli*** Cortés, 1976.—Neotropical: South America (Chile).

*Enchomyiashewelli* Cortés, 1976: 5. Holotype male (CNC). Type locality: Chile, Coquimbo, Elqui, Baños El Toro, 3300–4000 m [ca. 29°50′S, 70°1′W].

Reference: González and Vergés (2004: 54), redescription.

#### Genus *GONIA* Meigen, 1803

*SALMACIA* Meigen, 1800: 38. Meigen (1800) suppressed by ICZN (1963: 339).

***GONIA*** Meigen, 1803: 280. Type species: *Goniabimaculata* Wiedemann, 1819, by subsequent designation of Sabrosky and Arnaud (1965: 1075) [South Africa].

*SALMACIA* Meigen *in* Hendel, 1908: 65. First usage of *Salmacia* (*sensu* Meigen, 1800) as a valid name after Meigen, 1800; no type species designated originally or subsequently (see note).

*PHOSOCOCEPHALOPS* Townsend, 1927a: 237. Type species: *Phosococephalopsfulvus* Townsend, 1927 (= *Goniapallens* Wiedemann, 1830), by original designation [Brazil].

Note: The name *Salmacia* Meigen, 1800 became unavailable when the pamphlet of Meigen (1800) was suppressed by ICZN (1963: 339). *Salmacia* became available later when given by Meigen *in* Hendel (1908: 65), as explained in Evenhuis and Pape (2017: 51). This last work cited the type species of *Salmacia* Meigen *in* Hendel, 1908 as *Muscacapitata* De Geer, 1776 by designation of Coquillett (1910: 602) but this is incorrect; Coquillett (1910) designated a type species for *Salmacia* Meigen, 1800 (at the time an available name) not *Salmacia* Meigen *in* Hendel, 1908.

References: Coquillett (1910: 547, 602), type species of *Gonia* and *Salmacia* Meigen, 1800 (with *Gonia* [and others] in synonymy with *Salmacia*); Townsend (1931b: 177), synonymy of *Phosococephalops* with *Gonia*; Aldrich (1934: 4, 86), in key to Patagonian genera, synonymy, taxonomic notes, key to two Patagonian species; Townsend (1936c: 169, 281), diagnosis of adults and immatures of Goniini and key to genera (including *Gonia* and *Phosococephalops*), *Salmacia* as synonym of *Gonia*; Townsend (1941: 31, 42), redescriptions of *Gonia* (with *Salmacia* in synonymy) and *Phosococephalops*; Cortés and Campos (1971: 84), in key to tachinid genera of Tarapacá and Antofagasta regions, key to separate several species of *Gonia*; Cortés and Campos (1974: 113) and Cortés (1984: 379), in keys to tachinid genera of Tarapacá and Antofagasta regions; Cortés (1986: 144), in key to tachinid genera of Aysén and Magallanes regions; González and Vergés (2004: 41, 55), in key to Chilean genera of Goniini, redescription.

*crassicornis* (Fabricius, 1794).—Not Chile [Brazil, Peru, Venezuela; also Middle America, West Indies and Nearctic].

*Muscacrassicornis* Fabricius, 1794: 328.

Note: *Goniacrassicornis* was recorded from Chile and Puerto Rico by Ashley (1979), citing Jones (1913) and Van Dine (1913) as sources. Neither of these last two papers record *G.crassicornis* from Chile and Ashley’s (1979) record is assumed to be in error.

***lineata*** Macquart, 1851.—Neotropical: South America (Argentina, Chile, Peru).

*Gonialineata* Macquart, 1851: 151 [also 1851: 178]. Lectotype male (MNHN, see note), by fixation of Aldrich (1934: 87) (examination of “type” in MNHN is regarded as a lectotype fixation). Type locality: “Patagonie” (i.e., Argentina or Chile).

*Goniachiliensis* of Blanchard (1854: 422), not Macquart, 1844. Misidentification (Aldrich 1934: 87).

Note: The online MNHN database records a male holotype in the Macquart collection for *Gonialineata* (number MNHN-ED-ED8338) based on a holotype determination label that DMW attached to the specimen in 1982. Macquart did not restrict the name-bearing type to a single specimen and the lectotype fixation of Aldrich (1934: 87) is accepted here [see Recommendation 73F of the *Code* (ICZN 1999), “Avoidance of assumption of holotype”].

References: Aldrich (1934: 87), taxonomic notes, recorded from Argentina, Chile and Peru; Cortés (1963: 249), notes on name-bearing type in MNHN; Cortés (1979: 79), separation of *Gonialineata* and *Goniapallens*; González and Vergés (2004: 55), redescription.

***pallens*** Wiedemann, 1830.—Neotropical: Greater Antilles (Cuba, Jamaica), eastern Lesser Antilles (Saint Vincent), Middle America (Mexico), South America (Argentina, Brazil, Chile, Ecuador, Paraguay, Peru).

*Goniapallens* Wiedemann, 1830: 346. Lectotype, unspecified sex (NHMW), by fixation of Townsend (1931b: 177) (examination of “Ht” from Brazil in NHMW is regarded as a lectotype fixation). Type locality: Brazil.

*Goniachilensis* Macquart, 1844: 50 [also 1844: 207]. Lectotype female (MNHN), by designation herein (see Lectotype Designations section). Type locality: Chile or Cuba.

*Phosococephalopsfulvus* Townsend, 1927a: 347 (as “*fulva*” on p. 237). Lectotype female (USNM), by fixation of Townsend (1931b: 177) (examination of “female Ht” from São Paulo in USNM [as “Lima” but later changed to “Washington” in Townsend 1941: 42] is regarded as a lectotype fixation for the single female in the type series). Type locality: Brazil, São Paulo, Itaquaquecetuba.

Notes: Cortés (1963: 248) questioned whether *Goniapallens* as interpreted here is a single species but we tentatively accept the synonymy and distribution above following Aldrich (1934: 87), Guimarães (1971: 176), González and Vergés (2004: 57) and others.

References: Van der Wulp (1888: 39), distribution as Brazil, Chile, Argentina, Mexico, Cuba and Jamaica; Williston (1896: 354), record from Saint Vincent, taxonomic notes; Townsend (1931b: 177), synonymy of *Phosococephalopsfulvus* with *Goniapallens*; Aldrich (1934: 87), synonymy, partial redescription, distribution as Argentina, Brazil, Chile, Ecuador, Paraguay and Peru; Blanchard (1963: 226), redescription, wing figure; Cortés (1963: 248), notes on type series in MNHN; Cortés (1979: 79), separation of *Goniapallens* and *Gonialineata*; González and Vergés (2004: 57), redescription.

##### *Nomen dubium* of *GONIA* Meigen, 1803

***virescens*** Macquart, 1844.—Neotropical: South America. Distribution not known beyond the imprecise type locality of Brazil or Chile.

*Goniavirescens* Macquart, 1844: 50 [also 1844: 207]. Type(s), female (1 female in MNHN, see note). Type locality: Brazil or Chile.

Note: The online MNHN database records a holotype for *Goniavirescens* Robineau-Desvoidy, 1863 (number MNHN-ED-ED6825) in the Macquart collection. This record is in error; Robineau-Desvoidy (1863a: 741) did not describe a new species but instead cited the earlier Macquart species as “*Goniavirescens*: Macq. Coll. du Muséum” and assigned it to the genus *Reaumuria* Robineau-Desvoidy, 1830. He gave the provenance as Egypt, whereas Macquart had cited the species from “Brésil ou du Chili”. Perhaps Egypt was given in error and the “holotype” of “*Goniavirescens* Robineau-Desvoidy, 1863” is an overlooked type of *Goniavirescens* Macquart, 1844.

Reference: Guimarães (1971: 176), unrecognised species of *Gonia*.

#### Genus *LESCHENAULTIA* Robineau-Desvoidy, 1830

***LESCHENAULTIA*** Robineau-Desvoidy, 1830: 324. Type species: *Leschenaultiacilipes* Robineau-Desvoidy, 1830, by subsequent designation of Townsend (1916b: 7) (see Evenhuis et al. 2010: 97) [Suriname].

*BLEPHARIPEZA* Macquart, 1844: 54 [also 1844: 211]. Type species: *Blepharipezarufipalpis* Macquart, 1844 (= *Leschenaultiacilipes* Robineau-Desvoidy, 1830), by monotypy [Mexico].

*ECHINOMASICERA* Townsend, 1915e: 413. Type species: *Echinomasicerahystrix* Townsend, 1915, by original designation [Peru].

*HARRISIOPSIS* Townsend, 1927a: 247. Type species: *Harrisiopsisspinosa* Townsend, 1927 (= *Leschenaultiacilipes* Robineau-Desvoidy, 1830), by original designation [Brazil].

*PARACHAETOPSIS* Blanchard, 1959: 163. Type species: *Parachaetopsisproseni* Blanchard, 1959 (= *Blepharipezabicolor* Macquart, 1846), by original designation [Argentina].

*BLEPHRARIPEZA*. Incorrect subsequent spelling of *Blepharipeza* Macquart, 1844 (Vimmer and Soukup 1940a: 217).

References: Coquillett (1910: 514), type species of *Blepharipeza*; Townsend (1931b: 175), synonymy of *Blepharipeza* and *Harrisiopsis* with *Leschenaultia*; Townsend (1936c: 186, 272, 276), diagnosis of adults and immatures of Harrisiini and key to genera (including *Echinomasicera* and *Leschenaultia*), synonymy; Townsend (1941: 77, 79), redescriptions of *Echinomasicera* and *Leschenaultia* (with *Blepharipeza* and *Harrisiopsis* in synonymy); Cortés (1984: 380), *Echinomasicera* in key to tachinid genera of Tarapacá and Antofagasta regions; Toma and Guimarães (2002), revision, synonymy including *Echinomasicera* and *Parachaetopsis* with *Leschenaultia*.

***hystrix*** (Townsend, 1915).—Neotropical: South America (Chile, Peru).

*Echinomasicerahystrix* Townsend, 1915e: 413. Holotype male (USNM). Type locality: Peru, Lima, Matucana, ca. 8000 ft.

References: Cortés (1984: 386), first description of female, first record from Chile; Toma and Guimarães (2002: 38, 66), in key to *Leschenaultia* species, figures, diagnosis.

#### Genus *PATELLOA* Townsend, 1916

***PATELLOA*** Townsend, 1916a: 619. Type species: *Phoroceraleucaniae* Coquillett, 1897, by original designation [United States]. **New record from Chile.**

*PATELLOAPSIS* Townsend, 1927a: 263. Type species: *Patelloapsissimilis* Townsend, 1927, by original designation [Brazil].

*YAHUARPHRYNO* Townsend, 1927a: 263. Type species: *Yahuarphrynopatelloides* Townsend, 1927, by original designation [Peru].

*MACROPATELLOA* Townsend, 1931d: 472. Type species: *Macropatelloatanumeana* Townsend, 1931, by original designation [Chile]. **Syn. nov.**

Note: *Patelloa* is currently known from 19 species that are widely distributed throughout the New World, including Argentina (three species) but not Chile (O’Hara et al. 2020: 491). *Macropatelloatanumeana* is a common species in Chile and Argentina and is well-represented in CNC. It is recognised here as a typical species of *Patelloa* based on the following diagnostic features of the genus: prosternum haired, parafacial bare, facial ridge with row of strong setae, first postsutural supra-alar seta well-developed, and setae on postpronotum arranged in a triangle (Wood 1987: 1206, Wood and Zumbado 2010: 1361).

References: Townsend (1936c: 218, 237), diagnosis of adults and immatures of Lydellini and key to genera (including *Macropatelloa*), diagnosis of adults and immatures of Trypherini and key to genera (including *Patelloa*, *Patelloapsis* and *Yahuarphryno*); Townsend (1941: 191, 307, 327), redescriptions of *Macropatelloa*, *Patelloa*, *Patelloapsis* and *Yahuarphryno*; Sabrosky and Arnaud (1965: 1104), synonymy of *Patelloapsis* with *Patelloa*; Guimarães (1971: 211), synonymy of *Yahuarphryno* with *Patelloa*.

***tanumeana*** (Townsend, 1931).—Neotropical: South America (Argentina, Chile). **Comb. nov.** (Fig. 5d)

*Macropatelloatanumeana* Townsend, 1931d: 472. Holotype female (USNM). Type locality: Chile, O’Higgins, Cardenal Caro, Tanumé [ca. 34°13′S, 71°55′W].

Note: Townsend (1931d: 472) described *Macropatelloatanumeana* from two males and one female from “Tanumé and Talagante, Chile”. The type locality of the female holotype was not given in the original description but was cited as Tanumé in Townsend (1941: 191).

References: Aldrich (1934: 69, 71), in key to Patagonian species of *Phorocera* Robineau-Desvoidy, 1830 (*s. lato*), redescription; Cortés (1945d: 158), in key to Chilean species of *Phorocera* (*s. lato*) and *Parasetigena* Brauer and Bergenstamm, 1891; Cortés (1950: 10), in key to Chilean species of *Phorocera* (*s. lato*); Guimarães (1971: 161), as unrecognised species of Exoristini; Cortés (1979: 80), first record from Argentina (as *Macropatelloatanumeana*); Cortés (1986: 144, 158), *Macropatelloa* in key to tachinid genera of Aysén and Magallanes regions, taxonomic notes on *M.tanumeana*; González (1992b: 179), survey data, as *M.tanumeana*.

#### Genus *PHILOCORUS* Cortés, 1976

***PHILOCORUS*** Cortés, 1976: 12. Type species: *Philocorusmontanum* Cortés, 1976, by original designation [Chile].

*PHILOCHORUS*. Incorrect subsequent spelling of *Philocorus* Cortés, 1976 (González and Vergés 2004: 41, 60).

References: Cortés (1986: 144), in key to tachinid genera of Aysén and Magallanes regions. González and Vergés (2004: 41, 60), in key to Chilean genera of Goniini, redescription.

***montanum*** Cortés, 1976.—Neotropical: South America (Chile).

*Philocorusmontanum* Cortés, 1976: 13. Holotype male (CNC). Type locality: Chile, Coquimbo, Elqui, Baños El Toro, 3300–4000 m [ca. 29°50′S, 70°1′W].

References: Cortés (1986: 158), first description of female; González and Vergés (2004: 61), redescription.

#### Genus *PROTOGONIOPS* Townsend, 1913

*PROTOGONIA* Townsend, 1912b: 347 (junior homonym of *Protogonia* Cope, 1881). Type species: *Protogoniaocellaris* Townsend, 1912, by original designation [Peru].

***PROTOGONIOPS*** Townsend, 1913a: 133 (*nomen novum* for *Protogonia* Townsend, 1912).

References: Townsend (1936c: 169, 280), diagnosis of adults and immatures of Goniini and key to genera (including *Protogoniops*), *Protogoniops* as valid name for *Protogonia*; Townsend (1941: 44), redescription of *Protogoniops* (with *Protogonia* in synonymy); Cortés and Campos (1974: 115) and Cortés (1984: 381), in keys to tachinid genera of Tarapacá and Antofagasta regions; González and Vergés (2004: 41, 61), in key to Chilean genera of Goniini, redescription.

***ocellaris*** (Townsend, 1912).—Neotropical: South America (Chile, Peru).

*Protogoniaocellaris* Townsend, 1912b: 348. Holotype male (USNM). Type locality: Peru, western base of Cordillera Occidental, Río Suyo, ca. 1500 ft.

References: Cortés and Campos (1974: 122), first description of female, first record from Chile; González and Vergés (2004: 61), redescription.

#### Genus *PSEUDOCHAETA* Coquillett, 1895

References: Coquillett (1910: 596, 615), type species of *Pseudochaeta*; Townsend (1936c: 237, 274), diagnosis of adults and immatures of Trypherini and key to genera (including *Metopiops*, *Phaenopsis* and *Pseudochaeta*), *Dimasicera* as synonym of *Phaenopsis*; Townsend (1941: 287, 309, 313), redescriptions of *Metopiops*, *Phaenopsis* (with *Dimasicera* in synonymy) and *Pseudochaeta*; Reinhard (1946), revision of North American species of *Phaenopsis* and *Pseudochaeta*, key to all New World species; Thompson (1964: 98), synonymy of *Phaenopsis* with *Pseudochaeta*, revision of Trinidad species; Wood (1987: 1210), synonymy of *Metopiops* and *Phaenopsis* with *Pseudochaeta*; O’Hara and Wood (1998: 756, 766), review of synonymy of Wood (1987).

### Subgenus METOPIOPS Townsend, 1912

*METOPIOPS* Townsend, 1912b: 338. Type species: *Metopiopsmirabilis* Townsend, 1912, by original designation [Peru].

There are no Chilean species in this subgenus.

### Subgenus PHAENOPSIS Townsend, 1912

*PHAENOPSIS* Townsend, 1912b: 362. Type species: *Phaenopsisarabella* Townsend, 1912, by original designation [Peru].

*DIMASICERA* Townsend, 1915c: 62. Type species: *Dimasiceranitida* Townsend, 1915 (= *Phaenopsisarabella* Townsend, 1912), by original designation [Peru].

References: Cortés and Campos (1971: 23, 1974: 114) and Cortés (1984: 380), *Phaenopsis* in keys to tachinid genera of Tarapacá and Antofagasta regions.

***arabella*** (Townsend, 1912).—Neotropical: South America (Chile, Peru).

*Phaenopsisarabella* Townsend, 1912b: 363. Holotype male (USNM). Type locality: Peru, Piura, Valle del Río Chira, Sullana.

*Dimasiceranitida* Townsend, 1915c: 64. Holotype female (USNM). Type locality: Peru, Piura, Valle del Río Chira, near Sullana.

References: Townsend (1941: 310), synonymy of *Dimasiceranitida* with *Phaenopsisarabella*; Reinhard (1946: 111), notes on synonymy, in key to New World species of *Phaenopsis* and *Pseudochaeta*; Cortés and Campos (1971: 97), first record from Chile; Guimarães (1971: 163), earlier synonymy of *Dimasiceranitida* with *Phaenopsisarabella* apparently overlooked and both names listed as valid.

### Subgenus PSEUDOCHAETA Coquillett, 1895

***PSEUDOCHAETA*** Coquillett, 1895a: 309. Type species: *Pseudochaetaargentifrons* Coquillett, 1895, by original designation [United States].

Reference: Townsend (1908: 96), comparison with new genus *Trepophrys*.

There are no Chilean species in this subgenus.

#### Unplaced species of Goniini

***leliae*** Cortés & Campos, 1971.—Neotropical: South America (Chile).

*Lespesialeliae* Cortés & Campos, 1971: 91. Holotype male (EEAM). Type locality: Chile, Arica y Parinacota, Arica, Valle de Lluta, Rosario, 352 m (18°26′S, 70°06′W) (coordinates and elevation given on p. 11).

Note: See note under *A.robusta* for comments on the tribal placement of this species.

***negrensis*** Aldrich, 1934.—Neotropical: South America (Argentina, Chile). (Fig. 5e)

*Phoroceranegrensis* Aldrich, 1934: 72. Holotype male (NHMUK). Type locality: Argentina, Río Negro, Lago Gutiérrez.

Notes: *Phoroceranegrensis* was recorded from both Argentina and Chile in the original description.

*Phoroceranegrensis* was listed as an unplaced species of Blondeliini by Guimarães (1971: 153) but it unquestionably belongs to the *Cyzenis* Robineau-Desvoidy, 1863–*Frontiniella* Townsend, 1918 clade of Goniini, a complex with additional but undescribed species in Chile. These species typically have a haired eye, setose facial ridge (on lower half or more), and lack a pair of apical scutellar setae (Wood and Zumbado 2010: 1363). DNA barcoding by JEOH suggests that *Chrysoexorista* Townsend, 1915 is also close to or part of this clade. The molecular phylogeny of Stireman et al. (2019: 13 [fig. 8]) did not include *Cyzenis* but found a close relationship between *P.negrensis* and *Frontiniella*. We could assign *Phoroceranegrensis* to *Cyzenis* here based on external morphology but we are reluctant to place it to genus without a proper study of the undescribed species related to it.

References: Aldrich (1934: 69, 72), in key to Patagonian species of *Phorocera* Robineau-Desvoidy, 1830 (*s. lato*); Cortés (1945d: 158), in key to Chilean species of *Phorocera* (*s. lato*) and *Parasetigena* Brauer & Bergenstamm, 1891; Cortés (1950: 10), in key to Chilean species of *Phorocera* (*s. lato*); Guimarães (1971: 153), as unplaced species of Blondeliini.

***nimia*** Cortés & Campos, 1971.—Neotropical: South America (Chile).

*Lespesianimia* Cortés & Campos, 1971: 95. Holotype male (EEAM). Type locality: Chile, Arica y Parinacota, Arica, Valle de Lluta, km 57.

Note: See note under *A.robusta* for comments on the tribal placement of this species.

***robusta*** Aldrich, 1934.—Neotropical: South America (Argentina, Chile).

*Achaetoneurarobusta* Aldrich, 1934: 91. Holotype male (USNM). Type locality: Chile, Metropolitana de Santiago, Santiago, Cerro San Cristóbal.

Note: Guimarães (1971: 194) assigned *Achaetoneurarobusta* Aldrich to “Unplaced Species of Sturmiini” (i.e., Goniini) without commenting on its placement. Later, Guimarães (1983: 23, 28) transferred two additional species to this category and commented that all three produce microtype eggs (i.e., belong to Goniini, not Eryciini), writing: “*Lespesia* deposits membranous eggs on the body of the host … Chilean species recorded to this complex, viz. *L.robusta* Aldrich, *L.leliae* Cortés and *L.nimiae* Cortés, definitely do not belong to *Lespesia*, and their correct placement have not yet been established. Dissections of females of the three Chilean species show the presence of microtype eggs, the male and female genitalia being differently shaped”.

Reference: Gramajo (1998: 96), first record from Argentina (as *Achaetoneurarobusta*).

### Tribe WINTHEMIINI

#### Genus *WINTHEMIA* Robineau-Desvoidy, 1830

***WINTHEMIA*** Robineau-Desvoidy, 1830: 173. Type species: *Muscaquadripustulata* Fabricius, 1794, by subsequent designation of Desmarest *in* d’Orbigny (1849: 301) (see Evenhuis and Thompson 1990: 239) [Germany].

*MICROTRICHODES* Macquart, 1846: 288 [also 1846: 160]. Type species: *Microtrichodesanalis* Macquart, 1846, by original designation [Brazil].

*MASIPODA* Brauer & Bergenstamm, 1889: 162 [also 1889: 94]. Type species: *Masipodageminata* Brauer & Bergenstamm, 1889, by monotypy [Mexico].

*HEMIMASIPODA* Townsend, 1927a: 267. Type species: *Hemimasipodabrasiliensis* Townsend, 1927, by original designation [Brazil].

*OKEOPSIS* Townsend, 1927a: 267. Type species: *Okeopsispalpalis* Townsend, 1927, by original designation [Brazil].

*PROWINTHEMIA* Townsend, 1928a: 151. Type species: *Prowinthemiaparaguayensis* Townsend, 1928 (= *Exoristatricolor* van der Wulp, 1890), by original designation [Paraguay].

*BICRUCIOSTURMIA* Townsend, 1932b: 106. Type species: *Bicruciosturmiabicrucis* Townsend, 1932, by original designation [Brazil].

*PROMASIPODA* Townsend, 1934b: 399. Type species: *Promasipodapinguioides* Townsend, 1934, by original designation [Brazil].

*PRONEMORILLA* Townsend, 1935: 229. Type species: *Pronemorillamima* Townsend, 1935 (junior secondary homonym of *Winthemiamima* Reinhard, 1931; = *Winthemiatrinitatis* Thompson, 1963), by original designation [Brazil].

*WINTHEMIOPSIS* Blanchard, 1963: 212. Type species: *Winthemiopsisgrioti* Blanchard, 1963, by original designation [Argentina].

*MICROTRICHOMODES*. Incorrect subsequent spelling of *Microtrichodes* Macquart, 1846 (Guimarães 1972: 42).

*WINTHEMYA*. Incorrect subsequent spelling of *Winthemia* Robineau-Desvoidy, 1830 (Robineau-Desvoidy 1863a: 206–216; Vimmer and Soukup 1940a: 207).

*WINTHEMYIA*. Incorrect subsequent spelling of *Winthemia* Robineau-Desvoidy, 1830 (e.g., Vimmer and Soukup 1940b: 370).

*WITHEMIA*. Incorrect subsequent spelling of *Winthemia* Robineau-Desvoidy, 1830 (Etcheverry 1957: 187).

Notes: There is much confusion in the literature regarding the valid names of *Winthemia* species and their synonyms in the New World. For practical purposes the synonymy proposed by Coelho et al. (1989) is followed here.

Macquart (1846: 289 [also 1846: 161]) noted about his new genus *Microtrichodes*, “Le type de ce genre est du Brésil” [“The type of this genus is from Brazil”]. This statement is accepted as a type species designation for *Microtrichodes* of the single included species, *Microtrichodesanalis* Macquart, from Brazil.

The species treated by many authors as *Winthemiaignobilis* (van der Wulp) was moved to the Ethillini by Cerretti et al. (2012: 34) and is treated here under the name *Neoethillaignobilis* (not recorded from Chile; see explanation under that name).

References: Coquillett (1910: 565, 620), type species of *Masipoda* and *Winthemia* (with former in synonymy with latter; type species of *Winthemia* given as “*Muscaquadripustulata* Fabricius … by designation of Desvoidy … vol. 1, 1863, p. 207”); Reinhard (1931), revision of New World species, synonymy of *Hemimasipoda*, *Masipoda* and *Microtrichodes* with *Winthemia*, key, descriptions; Aldrich (1934: 3, 43), in key to Patagonian genera, synonymy of *Prowinthemia* with *Winthemia*; Townsend (1936c: 190), diagnosis of adults and immatures of Sturmiini and key to genera (including *Bicruciosturmia*, *Hemimasipoda*, *Masipoda*, *Microtrichodes*, *Okeopsis*, *Promasipoda*, *Pronemorilla*, *Prowinthemia* and *Winthemia*); Townsend (1941: 90–138), redescriptions of the aforementioned genera; Thompson (1963b: 960), revision of Trinidad species; Cortés and Campos (1971: 101), synonymy including *Pronemorilla* with *Winthemia*; Cortés and Campos (1971: 23, 1974: 114) and Cortés (1984: 380), in keys to tachinid genera of Tarapacá and Antofagasta regions; Guimarães (1971: 196), synonymy of *Okeopsis*, *Promasipoda* and *Winthemiopsis* with *Winthemia*; Guimarães (1972), revision of species from north of Mexico; Coelho et al. (1989), synonymy of *Bicruciosturmia* with *Winthemia*, key and review of South American species.

*quadripustulata* (Fabricius, 1794).—Not Chile [Palaearctic; also Nearctic and Oriental].

*Muscaquadripustulata* Fabricius, 1794: 324.

Note: *Winthemiaquadripustulata* was recorded from Chile by Molina-Ochoa et al. (2003: 262) based on an earlier record by Etcheverry (1957: 187, as “*Withemia*” *quadripustulata*). This is undoubtedly a misidentification; *W.quadripustulata* is widely distributed in the Palaearctic, Nearctic and Oriental regions and though possibly a species complex is not reliably known from South America. It was not recognised from America south of United States by Guimarães (1971, 1972) or from South America by Coelho et al. (1989).

***singularis*** Reinhard, 1931.—Neotropical: southern Lesser Antilles (Trinidad & Tobago), South America (Argentina, Brazil, ?Chile, Colombia, Ecuador, Paraguay, Peru, Venezuela).

*Winthemiasingularis* Reinhard, 1931: 38. Holotype male (USNM). Type locality: Argentina, Tucumán [province or city].

*Hemimasipodaalabamae* Townsend, 1940b: 892. Lectotype male (MZSP), by fixation of Coelho et al. (1989: 280) (examination of “holótipo macho” in MZSP is regarded as a lectotype fixation). Type locality: Brazil, São Paulo, Ribeirão Preto.

*Winthemiaaureonigra* Thompson, 1963b: 978. Holotype male (CNC). Type locality: Trinidad, Maracas Valley.

*Winthemiaroblesi* Valencia, 1972b: 366. Holotype male (SENASA, Lozada et al. 2005: 460). Type locality: Peru, Ica, Huamaní.

Note: The only record of *Winthemiasingularis* from Chile was given in a table in Molina-Ochoa et al. (2003: 262), as *Winthemiaroblesi*. Due to the difficult nature of identifying *Winthemia* specimens, the synonymy of Coelho et al. (1989: 280) needs confirmation, as does the presence of *Winthemiasingularis* in Chile. *Winthemiaaureonigra* (holotype examined by DMW) is a particularly unlikely synonym.

References: Coelho et al. (1989: 275, 280), in key to South American species, synonymy of *Hemimasipodaalabamae*, *Winthemiaaureonigra* and *Winthemiaroblesi* with *Winthemiasingularis*, distribution; Nihei (2016: 935), in catalogue of Tachinidae of Colombia.

***trinitatis*** Thompson, 1963.—Neotropical: southern Lesser Antilles (Trinidad & Tobago), South America (Argentina, Bolivia, Brazil, ?Chile, Colombia, Paraguay, Peru, Venezuela).

*Pronemorillamima* Townsend, 1935: 230 (junior secondary homonym of *Winthemiamima* Reinhard, 1931). Holotype female (MZSP). Type locality: Brazil, São Paulo, São Vicente.

*Winthemiatrinitatis* Thompson, 1963b: 971. Holotype male (CNC). Type locality: Trinidad, Chaguanas.

*Winthemiareliqua* Cortés & Campos, 1971: 101 (*nomen novum* for *Pronemorillamima* Townsend, 1935).

*reliquia*. Incorrect subsequent spelling of *reliqua* Cortés & Campos, 1971 (Valencia 1972b: 365, etc.).

Note: *Winthemiatrinitatis* was recorded from Chile by Coelho et al. (1989: 275) but we are doubtful that this species, which was originally described from Trinidad, occurs there.

References: Coelho et al. (1989: 275, 281), in key to South American species, distribution, and synonymy of *Pronemorillamima* Townsend, 1935 with *Winthemiatrinitatis*; Nihei (2016: 935), in catalogue of Tachinidae of Colombia.

#### Unplaced species of Winthemiini

***bullocki*** Aldrich, 1934.—Neotropical: South America (Argentina, Chile). (Fig. 5f)

*Phorocerabullocki* Aldrich, 1934: 70. Syntypes, 4 females (NHMUK, USNM, according to databases of these collections). Type localities: Chile, Araucanía (Malleco, Angol) and Metropolitana de Santiago (Santiago, Cerro San Cristóbal).

Note: *Phorocerabullocki* and two related but undescribed species are each represented in CNC by specimens from Chile and Argentina. A new genus in the Winthemiini may be warranted for these species. The katepimeron is haired as in other members of the tribe. The parafacial is bare (haired in *Winthemia* species) and facial ridge is setose.

References: Aldrich (1934: 69, 70), in key to Patagonian species of *Phorocera* Robineau-Desvoidy, 1830 (*s. lato*), description; Cortés (1945d: 159), in key to Chilean species of *Phorocera* (*s. lato*) and *Parasetigena* Brauer & Bergenstamm, 1891; Cortés (1950: 10), in key to Chilean species of *Phorocera* (*s. lato*); Campos (1953: 25), first description of male (in *Phorocera*); Guimarães (1971), name missing from catalogue); Henry (1987: 206), first record from Argentina (in *Phorocera* but genus unplaced in Tachinidae).

### Unplaced genus of Exoristinae

#### Genus *CALTAGIRONEA* Cortés & Campos, 1974

***CALTAGIRONEA*** Cortés & Campos, 1974: 117. Type species: *Caltagironeavera* Cortés & Campos, 1974, by original designation [Chile].

Note: Cortés and Campos (1974: 117) placed their new genus *Caltagironea* in the “Sturmiini” *sensu*Crosskey (1973: 91), a group comprising genera that would later be assigned to the Eryciini or Goniini depending upon reproductive habit (with those producing microtype eggs being placed in the latter). Cortés and Campos (1974) recognised both the Goniini and Sturmiini and hence their sturmiines are generally eryciines in modern terminology. González and Vergés (2004) excluded *Caltagironea* from their revision of Chilean Goniini and although it likely belongs to the Eryciini we cannot rule out its placement elsewhere in the Tachinidae.

Reference: Cortés (1984: 381), in key to tachinid genera of Tarapacá and Antofagasta regions.

***scillina*** Cortés & Campos, 1974.—Neotropical: South America (Chile).

*Caltagironeascillina* Cortés & Campos, 1974: 120. Holotype male (MEUC). Type locality: Chile, Arica y Parinacota, Arica, Valle de Camarones, Taltape, 300–400 m.

***vera*** Cortés & Campos, 1974.—Neotropical: South America (Chile).

*Caltagironeavera* Cortés & Campos, 1974: 119. Holotype male (MEUC). Type locality: Chile, Tarapacá, Tamarugal, south of (or road to) Chiapa, 3400–3800 m.

### Subfamily PHASIINAE

#### Tribe CYLINDROMYIINI

##### Genus *CYLINDROMYIA* Meigen, 1803

References: Coquillett (1910: 529, 577), type species of *Cylindromyia* and *Ocyptera* (with latter in synonymy with former); Aldrich (1926: 2), revision of North American species, synonymy of *Apinocyptera* and *Odontocyptera* with *Cylindromyia*; Aldrich (1934: 2, 8), in key to Patagonian genera, synonymy of *Dolichocyptera*, *Glossidionophora* and *Melanocyptera* with *Cylindromyia*, taxonomic notes, key to three Chilean species; Townsend (1936b: 63), diagnosis of adults and immatures of Cylindromyiini and key to genera (including *Apinocyptera*, *Catocyptera*, *Cylindromyia*, *Dolichocyptera*, *Ecatocypterops*, *Glossidionophora*, *Melanocyptera*, *Ocypteryx* and *Odontocyptera*); Townsend (1938: 87–143), redescriptions of the aforementioned genera; Cortés (1944e), key and review of Chilean species; Guimarães (1971: 15), synonymy of *Ecatocypterops* with *Cylindromyia*; Guimarães (1976), revision of species from south of the United States, synonymy including *Catocyptera* with *Cylindromyia*, three subgenera recognised for New World *Cylindromyia*.

#### Subgenus APINOCYPTERA Townsend, 1915

*APINOCYPTERA* Townsend, 1915f: 94. Type species: *Apinocypterasignata* Townsend, 1915 (= *Ocypterasignatipennis* van der Wulp, 1892), by original designation [Guatemala].

*ODONTOCYPTERA* Townsend, 1915h: 233. Type species: *Odontocypteranana* Townsend, 1915, by original designation [Mexico].

There are no Chilean species in this subgenus (Guimarães 1976: 24).

#### Subgenus CYLINDROMYIA Meigen, 1803

***CYLINDROMYIA*** Meigen, 1803: 279. Type species: *Muscabrassicaria* Fabricius, 1775, by monotypy [Europe].

*OCYPTERA* Latreille, 1804: 195. Type species: *Muscabrassicaria* Fabricius, 1775, by subsequent designation of Curtis (1837: 629) [Europe].

*GLOSSIDIONOPHORA* Bigot, 1885a: 237. *Nomen nudum*.

*GLOSSIDIONOPHORA* Bigot, 1885c: lv [also 1885c: lv, *Bull. Soc. Ent. France*]. Type species: *Glossidionophoranigra* Bigot, 1885, by subsequent designation of Townsend (1916b: 7) [Argentina].

*CATOCYPTERA* Townsend, 1927a: 215. Type species: *Catocypterabrasiliana* Townsend, 1927, by original designation [Brazil].

*MELANOCYPTERA* Townsend, 1927a: 215. Type species: *Melanocypteracarinata* Townsend, 1927, by original designation [Brazil].

*DOLICHOCYPTERA* Townsend, 1931c: 325. Type species: *Dolichocypterapirioni* Townsend, 1931, by original designation [Chile].

*OCYPTERYX* Townsend, 1931c: 326. Type species: *Ocypteryxochrescens* Townsend, 1931 (= *Ocypteradorsalis* Wiedemann, 1830), by original designation [Paraguay].

*ECATOCYPTEROPS* Townsend, 1935: 217. Type species: *Ecatocypteropsater* Townsend, 1935 (junior secondary homonym of *Ocypteraatra* Röder, 1885; = *Melanocypteracarinata* Townsend, 1927), by original designation [Brazil].

***aldrichi*** Cortés, 1944.—Neotropical: South America (Chile).

*Cylindromyiaaldrichi* Cortés, 1944e: 178. Holotype male (USNM). Type locality: Chile, Metropolitana de Santiago, Santiago, Santiago.

Note: The type locality of *Cylindromyiaaldrichi* was given as “Santiago” in Chile, which could be interpreted as either the city or province of that name. Cortés and Hichins (1969: 28) cited the former as the type locality (as “Santiago (Santiago)”) and we follow this interpretation.

Reference: Guimarães (1976: 8, 9), in key, taxonomic notes, figures.

***apicalis*** (Bigot, 1878).—Neotropical: South America (Chile).

*Ocypteraapicalis* Bigot, 1878: 45. Lectotype male [original type(s) not female as published by Bigot] (NHMUK), by designation of Guimarães (1976: 10). Type locality: Chile.

References: Aldrich (1934: 10), redescription, taxonomic notes; Guimarães (1976: 8, 10), in key, redescription, taxonomic notes, figures.

***nigra*** (Bigot, 1885).—Neotropical: South America (Argentina, Chile).

*Glossidionophoranigra* Bigot, 1885c: lv [also 1885c: lv, *Bull. Soc. Ent. France*]. Holotype female [not male as published, Guimarães 1976: 19] (NHMUK). Type locality: Argentina, Buenos Aires, Buenos Aires.

*Glossidionophoracylindrica* Brauer, 1899: 499. Holotype female [not male as published, Guimarães 1976: 19] (NHMUK). Type locality: Argentina, Buenos Aires, Buenos Aires.

*Cylindromyiaatricauda* Aldrich, 1934: 10. Holotype female (NHMUK). Type locality: Chile, Valparaíso, San Felipe de Aconcagua, Llay-Llay [as “Llaillai”].

Note: The holotype of *Glossidionophoranigra* Bigot, 1885 is also the holotype of *Glossidionophoracylindrica* Brauer, 1899. Brauer described *Glossidionophoracylindrica* from a specimen in the Bigot collection labelled with that name but was unaware that Bigot had described *Glossidionophoranigra* from the same specimen a few years earlier (Guimarães 1976: 19–20).

Reference: Guimarães (1976: 7, 19), in key, synonymy of *Cylindromyiaatricauda* and *Glossidionophoracylindrica* with *Glossidionophoranigra*, redescription, taxonomic notes, figures, first record from Chile as *Cylindromyianigra*.

***pirioni*** (Townsend, 1931).—Neotropical: South America (Chile).

*Dolichocypterapirioni* Townsend, 1931c: 326. Holotype female (USNM). Type locality: Chile, Metropolitana de Santiago, Santiago, Cerro San Cristóbal.

Reference: Guimarães (1976: 7, 22), in key, taxonomic notes.

***porteri*** (Brèthes, 1925).—Neotropical: South America (Argentina, Chile).

*Ocypteraporteri* Brèthes, 1925: 208. Holotype female [not male as published, Mulieri et al. 2013: 169] (MACN). Type locality: Chile, Metropolitana de Santiago, Santiago, Las Condes.

References: Aldrich (1934: 9), redescription; Cortés (1963: 251), notes on name-bearing type in MACN, as male; Guimarães (1976: 8, 21), in key, redescription, taxonomic notes, figures; Gramajo (1998: 91), first record from Argentina; Mulieri et al. (2013: 169), notes on holotype in MACN.

### Tribe GYMNOSOMATINI

#### Genus *GYMNOSOMA* Meigen, 1803

*RHODOGYNE* Meigen, 1800: 39. Meigen (1800) suppressed by ICZN (1963: 339).

***GYMNOSOMA*** Meigen, 1803: 278. Type species: *Muscarotundata* Linnaeus, 1758 (as “*Muscarotundata* Fabr.”), by monotypy [Europe].

*RHODOGYNE* Meigen *in* Hendel, 1908: 66. Type species: *Muscarotundata* Linnaeus, 1758 (as “*M.rotundata* F.”), by monotypy (see Evenhuis and Pape 2017: 50) [Europe].

Note: The name *Rhodogyne* Meigen, 1800 became unavailable when the pamphlet of Meigen (1800) was suppressed by ICZN (1963: 339). *Rhodogyne* became available later when given by Meigen *in* Hendel (1908: 65), as explained in Evenhuis and Pape (2017: 50).

References: Coquillett (1910: 548, 600), type species of *Gymnosoma* and *Rhodogyne* Meigen, 1800 (with former in synonymy with latter); Townsend (1936b: 44), diagnosis of adults and immatures of Gymnosomatini and key to genera (including *Gymnosoma*); Townsend (1936c: 281), *Rhodogyne* as synonym of *Gymnosoma*; Townsend (1938: 7), redescription of *Gymnosoma* (with *Rhodogyne* in synonymy); Cortés and Campos (1971: 21, 1974: 112) and Cortés (1984: 378), in keys to tachinid genera of Tarapacá and Antofagasta regions.

***neotropicale*** Cortés & Campos, 1971.—Neotropical: South America (Chile, Peru).

*Gymnosomaneotropicale* Cortés & Campos, 1971: 27. Holotype male (EEAM). Type locality: Chile, Arica y Parinacota, Arica, Valle de Lluta, km 23.

Reference: Vergara de Sánchez and Raven (1990: 94), first record from Peru.

#### Genus *TRICHOPODA* Berthold, 1827

References: Coquillett (1897: 47), key to species of America north of Mexico; Townsend (1897: 273), key to species in Vera Cruz; van der Wulp (1903: 433), revision of Central American species; Townsend (1908: 129), key to genera of Trichopodini; Coquillett (1910: 546, 593, 616), type species of *Galactomyia*, *Polistomyia* and *Trichopoda* (with first two in synonymy with last, *Trichopoda* as “*Trichiopoda*Latreille … 1829”); Townsend (1936b: 47), diagnosis of adults and immatures of Trichopodini and key to genera (including *Polistomyia*, “*Trichiopoda*” and *Trichopodopsis*); Townsend (1936c: 275), synonymy of *Galactomyia* with “*Trichiopoda*”; Townsend (1938: 25, 28, 30), redescriptions of *Polistomyia*, “*Trichiopoda*” (with *Galactomyia* in synonymy) and *Trichopodopsis*; Sabrosky (1950: 361, 366), key to genera of Trichopodini, synonymy of *Trichopodopsis* with *Trichopoda*, taxonomic notes; Blanchard (1966a: 62, 81), *Trichopodopsis* as valid genus, *Eutrichopodopsis* as new genus; Guimarães (1971: 7, 10), *Eutrichopodopsis* as valid genus, *Trichopodopsis* as synonym of TrichopodasubgenusGalactomyia; Liljesthröm (1992: 51, 56), revision of Argentinian species, synonymy of *Eutrichopodopsis* with *Trichopoda*; Dios and Nihei (2020), revision of Neotropical species.

### Subgenus GALACTOMYIA Townsend, 1908

*GALACTOMYIA* Townsend, 1908: 135. Type species: *Trichopodaradiata* Loew, 1863 (= *Therevalanipes* Fabricius, 1805), by subsequent designation of Coquillett (1910: 546) [United States].

*TRICHOPODOPSIS* Townsend, 1913b: 148, 313. Type species: *Muscapennipes* Fabricius, 1781, by subsequent monotypy of Anonymous (1913: 313) (see Evenhuis et al. 2015: 268) [North America].

*ECTOPHASIOPSIS* Townsend, 1915e: 439. Type species: *Ectophasiopsischilensis* Townsend, 1915 (= *Trichopodaarcuata* Bigot, 1876), by original designation [Chile]. **Syn. nov.**

*EUTRICHOPODOPSIS* Blanchard, 1966a: 81. Type species: *Eutrichopodopsisfunebris* Blanchard, 1966 (= *Muscapennipes* Fabricius, 1781), by original designation [Argentina].

*ECTOPHASIOPS*. Incorrect subsequent spelling of *Ectophasiopsis* Townsend, 1915 (Sabrosky 1950: 361).

*TRICHOPODOSIS*. Incorrect subsequent spelling of *Trichopodopsis* Townsend, 1913 (Mallea et al. 1977: 21, 23).

Note: *Ectophasiopsis* was recognised as a valid genus with a single species until recently revised by Dios and Nihei (2017), resulting in an increase in the number of species to three (with only the original species, *E.arcuata*, known from Chile). The species of *Ectophasiopsis*, *Trichopoda* (*sensu*Dios and Nihei 2020) and *Eutrichopoda* Townsend, 1908 (*sensu*Dios and Nihei 2016) all share a common habitus of black or black and yellow bodies and wings, and a row of long and distinctive “feather-like” setae on the hind tibia. A key to separate these three genera was given in Dios and Nihei (2017: 4) but the phylogenetic relationships among them have yet to be studied. Possibly all three taxa should be combined under one genus, for which the name *Trichopoda* would apply, but we have only explored the relationship between *Ectophasiopsis* and *Trichopoda*. Single legs of more than 50 CNC specimens of *Trichopoda* species from Canada, United States and Costa Rica and one Chilean specimen of *E.arcuata* (CNC487602, figs 30–31 in Stireman et al. 2016: 37) were sent to the Biodiversity Institute of Ontario (BIO) at the University of Guelph for DNA barcoding of the COI gene. A neighbor-joining tree clustered *E.arcuata* among ca. 40 samples of *T.pennipes* (Fabricius, 1781) and undetermined Costa Rican *Trichopoda*. These were all assigned to the same BIN (Barcode Index Number), BOLD:AAD9027, except for a few Costa Rican specimens. Sister to this clade was one consisting of three *Trichopodaindivisa* Townsend, 1897 and five *Trichopodaplumipes* (Fabricius, 1805), with each species in its own BIN. These last two species are members of the subgenus Trichopoda, whereas *T.pennipes* belongs to subgenus Galactomyia. We accept that the members of the BIN to which *E.arcuata* and *T.pennipes* belong are closely related, but we suspect from the morphological diversity within the sampled group that several or more species are involved and more sensitive molecular analyses may be needed to resolve them. For the present we restrict our taxonomic changes to the synonymy of *Ectophasiopsis* with *Trichopoda* and the assignment of *E.arcuata* and its allies *E.gradata* and *E.ypiranga* to subgenus Galactomyia.

References: Aldrich (1934: 2, 11), *Ectophasiopsis* in key to Patagonian genera, taxonomic notes; Townsend (1936b: 53), diagnosis of adults and immatures of Phasiini and key to genera (including *Ectophasiopsis*); Townsend (1938: 47), redescription of *Ectophasiopsis*; Sabrosky (1950: 361), *Ectophasiopsis* (as “*Ectophasiops*”) moved to Trichopodini; Dios and Nihei (2017), revision of *Ectophasiopsis*.

***arcuata*** (Bigot, 1876).—Neotropical: South America (Argentina, Chile). Australasian & Oceanian: Polynesia (Easter Island, introduced). **Comb. revived.**

*Trichopodaarcuata* Bigot, 1876: 397. Lectotype male (NHMUK), by designation of Dios and Nihei (2017: 6). Type locality: Chile.

*Ectophasiopsischilensis* Townsend, 1915e: 440. Holotype, unspecified sex [female, see note] (USNM). Type locality: Chile.

Note: Townsend (1915e: 440) described *Ectophasiopsischilensis* from “one female and two males” and designated one of them as “Holotype.–Cat. No. 19460, U.S.N.M.”. This was a valid designation of a holotype even though Townsend did not specify which specimen it was. Townsend (1938: 47) later cited the holotype as the single female (as “Ht female”). The name-bearing type does not comprise all three of the original specimens as inferred by Dios and Nihei (2017: 5, as “Syntypes, two ♂♂ and one ♀”).

References: Aldrich (1934: 12), synonymy, redescription, taxonomic notes; Wolcott (1948: 471), introduced to Puerto Rico but not established; Verbeke (1962: 121, etc.), description of male terminalia; Cortés (1979: 79), first record from Argentina; Ripa et al. (1995: 432), introduced to Easter Island; Gramajo (1998: 92), cited as first record from Argentina but preceded by Cortés (1979); Stireman et al. (2016: 37), habitus images; Dios and Nihei (2017: 5, 7), in key, redescription, figures.

*gradata* Wiedemann, 1830.—Not Chile [Argentina, Brazil, Uruguay]. **Comb. revived.**

*Trichopodagradata* Wiedemann, 1830: 275. Lectotype female (NHMW), by fixation of Dios and Nihei (2017: 10) (examination of “Holotype ♀” from Brazil in NHMW is regarded as a lectotype fixation). Type locality: Brazil.

*Trichopodopsisincognita* Blanchard, 1966a: 62. Holotype female (probably lost, Dios and Nihei 2017: 10). Type locality: Argentina, La Rioja [province].

*Trichopodopsisargentinensis* Blanchard, 1966a: 65. Holotype male (INTA). Type locality: Argentina, Córdoba [province].

*Trichopodopsischristenseni* Blanchard, 1966a: 78. Holotype male (INTA). Type locality: Argentina, Buenos Aires, José C. Paz.

Note: The relative priority of *Trichopodopsisincognita* Blanchard, 1966, *Trichopodopsisargentinensis* Blanchard, 1966 and *Trichopodopsischristenseni* Blanchard, 1966, when the three are treated as synonyms, was established by Liljesthröm (1992: 57), as the First Reviser (Article 24.2.2 of the *Code*, ICZN 1999).

References: Liljesthröm (1992: 57), synonymy of *Trichopodopsischristenseni* and *T.incognita* with *T.argentinensis*, as species of *Trichopoda*; Dios and Nihei (2017: 10), synonymy of *T.argentinensis* and its two synonyms with *T.gradata*, reassigned to *Ectophasiopsis*, redescription, figures.

*ypiranga* (Dios & Nihei, 2017).—Not Chile [Argentina, Brazil]. **Comb. nov.**

*Ectophasiopsisypiranga* Dios & Nihei, 2017: 18. Holotype male (FIOC). Type locality: Brazil, São Paulo, São Paulo, Ipiranga [as “Ypiranga”].

Note: Information about an intended paratype of *Ectophasiopsisypiranga* was inadvertently removed from the manuscript of Dios and Nihei (2017) prior to publication (pers. comm., R. de V.P. Dios). It was the only specimen of *E.ypiranga* examined from Argentina and would have explained the inclusion of Argentina in the stated distribution and the dot on the distribution map. The specimen is a male in MNHN with the following data: “Museum Paris / Chaco de Santiago / del Estero / bords du rio Salado / la Palisa del Bracho / 25 kil. N. O. d’Icaño / E. R. Wagner 1909” (details courtesy of R. de V.P. Dios).

### Subgenus TRICHOPODA Berthold, 1827

***TRICHOPODA*** Berthold, 1827: 508 (as “*Trichopode*” (vernacular) by Latreille 1825: 498, name first latinised in Berthold’s German translation of Latreille (1825); see Sabrosky 1950: 366). Type species: *Therevaplumipes* Fabricius, 1805, by subsequent designation of Coquillett (1910: 616) [United States].

*POLISTOMYIA* Townsend, 1908: 132. Type species: *Trichopodatrifasciata* Loew, 1863 (= *Therevaplumipes* Fabricius, 1805), by original designation [United States].

*THICHOPODA*. Incorrect subsequent spelling of *Trichopoda* Berthold, 1827 (Guimarães 1971: 7).

*TRICHIOPODA*. Incorrect spelling of *Trichopoda* Berthold, 1827 (e.g., Latreille 1829: 512; Coquillett 1910: 616; Townsend 1913b: 147; see Sabrosky 1999: 313).

References: Townsend (1913b: 147), synonymy of *Polistomyia* with *Trichopoda* (as “*Trichiopoda*”), taxonomic notes; Townsend (1915g: 122), *Polistomyia* reinstated as valid genus, nomenclatural and taxonomic notes; Aldrich (1931: 3), synonymy of *Polistomyia* with “*Trichiopoda*”.

There are no Chilean species in this subgenus.

### Tribe LEUCOSTOMATINI

#### Genus *LEUCOSTOMA* Meigen, 1803

***LEUCOSTOMA*** Meigen, 1803: 279. Type species: *Ocypterasimplex* Fallén, 1815, by subsequent monotypy of Meigen (1824: 234) [Sweden].

*PSALIDA* Rondani, 1856: 76. Type species: *Psalidaleucostoma* Rondani, 1856 (as “*TachinaLeucostoma* Mgn.”) (= *Ocypterasimplex* Fallén, 1815), by original designation (see O’Hara et al. 2011: 152) [Italy].

*SIPHOPSALIDA* Townsend, 1915e: 439. Type species: *Siphopsalidameridionalis* Townsend, 1915, by original designation [Peru].

*CYCLODIONAEA* Townsend, 1915h: 233. Type species: *Cyclodionaeaacuminata* Townsend, 1915 (= *Muscaaterrima* Villers, 1789), by original designation [United States].

*PARADIONAEA* Townsend, 1916a: 631. Type species: *Leucostomaatra* Townsend, 1891 (= *Ocypterasimplex* Fallén, 1815), by original designation [United States].

*NEOPSALIDA* Townsend, 1916a: 632. Type species: *Leucostomaneomexicana* Townsend, 1892 (= *Muscaaterrima* Villers, 1789), by original designation [United States].

References: Coquillett (1910: 561, 595), type species of *Leucostoma* and *Psalida* (with latter in synonymy with former); Aldrich (1934: 3, 28), in key to Patagonian genera, synonymy, taxonomic notes; Townsend (1936b: 77), diagnosis of adults and immatures of Leucostomatini and key to genera (including *Cyclodionaea*, *Leucostoma*, *Neopsalida*, *Paradionaea* and *Siphopsalida*); Townsend (1936c: 280), *Psalida* as synonym of *Leucostoma*; Townsend (1938: 185, 189, 191, 192, 196), redescriptions of *Cyclodionaea*, *Leucostoma* (with *Psalida* in synonymy), *Neopsalida*, *Paradionaea* and *Siphopsalida*; Reinhard (1956), revision of New World species; Cortés and Campos (1971: 21, 1974: 113) and Cortés (1984: 379), in keys to tachinid genera of Tarapacá and Antofagasta regions.

***aterrimum*** (Villers, 1789).—Neotropical: Greater Antilles (Puerto Rico), Middle America (Mexico), South America (Argentina, Chile). Nearctic: Canada, United States. Palaearctic: Europe. Australasian & Oceanian: Hawaii (immigrant).

*Muscaaterrima* Villers, 1789: 548. Lectotype male (MNHN, see note), by fixation of Townsend (1932a: 33) (examination of “Male Ht” from Europe in MNHN is regarded as a lectotype fixation). Type locality: Europe.

*Leucostomaneomexicana* Townsend, 1892c: 169. Holotype male (SEMC, Byers et al. 1962: 175). Type locality: USA, New Mexico, Las Cruces.

*Cyclodionaeaacuminata* Townsend, 1915h: 234. Holotype female (USNM). Type locality: USA, California, Santa Clara County.

Notes: The lectotype of *Muscaaterrima* is not among the types currently listed in the online MNHN database but is assumed to be in the Muséum based on its study there by Townsend (1932a: 33).

Bezzi and Stein (1907: 327) listed both *L.aterrimum* and *L.simplex* as valid species in Europe and both species were recognised from the Americas in Reinhard’s (1956) revision of *Leucostoma*. Subsequent catalogues in the Americas (Sabrosky and Arnaud 1965: 976; Guimarães 1971: 17; O’Hara and Wood 2004: 224) also recognised both species. However, Herting (1984: 174) treated *M.aterrima* as a questionable synonym of *L.simplex* and later Herting and Dely-Draskovits (1993: 420) removed the questionable status and listed the names as synonyms but continued to treat *L.simplex* as the valid name even though it is the junior synonym. Subsequent authors in Europe have recognised only *L.simplex* but the two names are in use in the Americas for two broadly distributed species, both recorded here from Chile. Preliminary DNA barcoding by JEOH of seven specimens in CNC grouped four as *L.simplex* from Czech Republic (CNCDIPTERA 161997, CNCDIPTERA 161998) and New Mexico, USA (CNCDIPTERA 104203, CNCDIPTERA 104205) and three as *L.aterrimum* from Ontario, Canada (CNC602375) and Chile (CNC487609, CNC487610). What this signifies is not clear and further study is needed to determine if: 1) the two names are correctly applied in the Americas (and the names are thus not synonyms), 2) one or both names are misapplied in the Americas (and one or both of the species must be given a different available or new name), or 3) the names *L.aterrimum* and *L.simplex* are truly synonyms as currently treated in Europe and the valid name is *L.aterrimum* according to the *Code* (ICZN 1999).

References: Aldrich (1934: 29), synonymy, diagnosis, taxonomic notes, first records from Argentina and Chile; Reinhard (1956: 160), synonymy, redescription; Sabrosky and Arnaud (1965: 976), distribution including Mexico; Guimarães (1971: 17), distribution including Puerto Rico; Nishida (1992: 121), recorded from Hawaii as an immigrant (i.e., not purposely introduced).

***simplex*** (Fallén, 1815).—Neotropical: South America (Argentina, Chile). Nearctic: Canada, United States. Palaearctic: Central Asia, China [Pal.], Europe, Kazakhstan, Mongolia, Russia, Transcaucasia. Afrotropical: Cape Verde, Sierra Leone. Australasian & Oceanian: Australia, Hawaii (immigrant).

*Ocypterasimplex* Fallén, 1815: 240. Holotype female [not syntypes of both sexes as cited by Herting 1984: 174] (NHRS). Type locality: Sweden, Småland, Kalmar Län.

*Psalidaleucostoma* Rondani, 1856: 76 (as “*TachinaLeucostoma* Mgn.”, see O’Hara et al. 2011: 152). Type(s), female (not located). Type locality: Italy.

*Leucostomaatra* Townsend, 1891: 380. Holotype male (SEMC, Byers et al. 1962: 174). Type locality: USA, Illinois, Carlinville.

Note: The identity of *Leucostomasimplex* is discussed above under *L.aterrimum*.

References: Aldrich (1934: 28), diagnosis, taxonomic notes, first record from Chile (as *Leucostomaatra*); Reinhard (1956: 159), synonymy of *Leucostomaatra* with *Ocypterasimplex*, redescription; Cortés and Hichins (1969: 40), Chilean records (as *Leucostomaater*); Cortés and Campos (1971: 36), *Leucostomasimplex* accepted as the valid name with *Leucostomaatra* in synonymy; Cortés (1979: 80), first record from Argentina; Nishida (1992: 121), recorded from Hawaii as an immigrant (i.e., not purposely introduced).

#### Genus *PERIOSTOMA* Cortés, 1986

***PERIOSTOMA*** Cortés, 1986: 145. Type species: *Periostomaflabellatum* Cortés, 1986, by original designation [Chile].

Reference: Cortés (1986: 142), in key to tachinid genera of Aysén and Magallanes regions.

***flabellatum*** Cortés, 1986.—Neotropical: South America (Chile).

*Periostomaflabellatum* Cortés, 1986: 145. Holotype male (MEUC). Type locality: Chile, Magallanes y de la Antártica Chilena, Ultima Esperanza, Parque Nacional Torres del Paine, Laguna Amarga.

### Tribe PHASIINI

#### Genus *PHASIA* Latreille, 1804

***PHASIA*** Latreille, 1804: 195. Type species: *Conopssubcoleoptratus* Linnaeus, 1767, by subsequent monotypy of Latreille (1805: 379); see rulings by ICZN (1970, 2006) [Sweden].

*ALOPHORA* Robineau-Desvoidy, 1830: 293. Type species: *Syrphushemipterus* Fabricius, 1794, by subsequent designation of Robineau-Desvoidy (1863b: 226, as “*Therevahemiptera* de Fabricius”) [United Kingdom].

*HYALOMYA* Robineau-Desvoidy, 1830: 298. Type species: *Phasiasemicinerea* Meigen, 1824 (= *Phasiapusilla* Meigen, 1824), by subsequent designation of Westwood (1840: 140) [probably Germany].

*HYALOMYIA* Macquart, 1834: 69 [also 1834: 205]. Unjustified emendation of *Hyalomya* Robineau-Desvoidy, 1830 (see Evenhuis et al. 2010: 90).

*ALLOPHORA* Mik, 1894: 49. Unjustified emendation of *Alophora* Robineau-Desvoidy, 1830 (see Evenhuis et al. 2010: 36).

*PARAPHORANTHA* Townsend, 1915b: 20. Type species: *Alophoragrandis* Coquillett, 1897, by original designation [United States].

*PHORANTHELLA* Townsend, 1915b: 23. *Nomen nudum* (by ruling of ICZN 1954: 311).

*ALOPHORELLOPSIS* Townsend, 1927a: 209. Type species: *Alophorellopsiscapitata* Townsend, 1927, by original designation [Brazil].

*EPAULOPHASIA* Townsend, 1934a: 207. Type species: *Epaulophasiaofficialis* Townsend, 1934, by original designation [Brazil].

*HEYNEOPHASIA* Townsend, 1934a: 208. Type species: *Heyneophasiaheynei* Townsend, 1934, by original designation [Costa Rica].

*XANTHOTRICHIUS* Townsend, 1934a: 209. Type species: *Xanthotrichiusxenos* Townsend, 1934, by original designation [Brazil].

*XIPHOPHASIA* Townsend, 1937a: 116. Type species: *Xiphophasiaushpayacua* Townsend, 1937, by monotypy [Peru].

*TRICHOPHASIA* Townsend, 1939b: 447 (junior homonym of *Trichophasia* Swainson, 1839). Type species: *Trichophasiatransita* Townsend, 1939, by original designation [Brazil].

*PARAPHASIANA* Townsend, 1940b: 889. Type species: *Paraphasianadysderci* Townsend, 1940 (junior secondary homonym of *Euphoranthadysderci* Townsend, 1938; = *Phasiaaurodysderci* Nihei & Dios, 2016), by original designation [Brazil].

*ANDROEURYOPS* Beneway, 1961: 44. Type species: *Hyalomyiaecitonis* Townsend, 1897, by original designation [Mexico].

Note: Herting (1984: 168) designated *Conopssubcoleoptrata* Linnaeus, 1767 as the type species of *Thereva* Fabricius, 1798, a junior homonym of *Thereva* Latreille, 1796 (Diptera, Therevidae). Later, an application to the International Commission on Zoological Nomenclature (Holston et al. 2003) resulted in the placement of the name *Thereva* Fabricius, 1798 on the Official Index of Rejected and Invalid Generic Names in Zoology (ICZN 2006).

References: Coquillett (1910: 505, 553, 587), type species of *Alophora*, *Hyalomya* and *Phasia* (with first two in synonymy with *Phasia*); Aldrich (1934: 2, 13), in key to Patagonian genera, synonymy, taxonomic notes, key to four Patagonian species (as “*Hyalomyia*” Robineau-Desvoidy); Townsend (1936b: 53), diagnosis of adults and immatures of Phasiini and key to genera (including *Alophorellopsis*, *Epaulophasia*, *Heyneophasia*, *Hyalomya*, *Paraphorantha*, *Phasia*, *Phoranthella* and *Xanthotrichius*; Townsend (1936c: 271), *Alophora* as synonym of *Phasia*; Townsend (1938: 36, 50, 56, 57, 64, 65, 68, 73, 74), redescriptions of *Alophorellopsis*, *Epaulophasia*, *Heyneophasia*, *Hyalomya*, *Paraphorantha*, *Phasia* (with *Alophora* in synonymy), *Phoranthella*, *Xanthotrichius* and *Xiphophasia*; Sabrosky and Arnaud (1965: 968), synonymy of *Alophorellopsis* with *Hyalomya* Robineau-Desvoidy; Herting (1974: 37, 1984: 170), synonymy of *Hyalomya* with *Phasia* Latreille; Cortés (1986: 142), in key to tachinid genera of Aysén and Magallanes regions (as *Hyalomya*); Wood (1987: 1258), synonymy including *Hyalomya*, *Paraphorantha* and *Phoranthella* with *Phasia*; O’Hara and Wood (1998: 756, 765), review of synonymy of Wood (1987); Sun and Marshall (2003: 19), synonymy of *Androeuryops*, *Epaulophasia*, *Heyneophasia*, *Paraphasiana*, *Trichophasia*, *Xanthotrichius* and *Xiphophasia* with *Phasia* Latreille.

***chilensis*** (Macquart, 1851).—Neotropical: Middle America (Mexico), South America (Argentina, Brazil, Chile, Peru, Uruguay, Venezuela). Nearctic: United States.

*Hyalomyiachilensis* Macquart, 1851: 189 [also 1851: 216]. Lectotype male (MNHN, see note), by fixation of Aldrich (1934: 14) (examination of “type, a male” from Chile in MNHN is regarded as a lectotype fixation). Type locality: Chile.

*Paraphoranthaperuviana* Townsend, 1936a: 489. Syntypes, 2 males and 2 females (USNM). Type locality: Peru, La Libertad, Pacasmayo, Jequetepeque.

*Paraphoranthadimidiata* Townsend, 1937b: 318. Syntypes, 1 male and 1 female (USNM). Type localities: Brazil, São Paulo, Tietê and Campinas.

*Paraphoranthapollinosa* Brooks, 1945: 660. Holotype male (MCZ). Type locality: USA, Maryland, Chesapeake Beach.

*Paraphoranthaauricaudata* Brooks, 1945: 661. Holotype male (CNC). Type locality: USA, Oregon, Milton.

Note: The online MNHN database records a male holotype in the Macquart collection for *Hyalomyiachilensis* (number MNHN-ED-ED8382) based on a holotype determination label that DMW attached to the specimen in 1985. Macquart did not restrict the name-bearing type to a single specimen and the lectotype fixation of Aldrich (1934: 14) is accepted here [see Recommendation 73F of the *Code* (ICZN 1999), “Avoidance of assumption of holotype”].

References: Aldrich (1934: 14), redescription, taxonomic notes; Blanchard (1940: 224), first record from Argentina; Berry (1951: 339, 340), first record from Peru, figures of larval cephaloskeleton and puparium; Cortés (1963: 249), notes on name-bearing type of *Hyalomyiachilensis*; Sun and Marshall (2003: 158, 159, 165), in key, synonymy of *Paraphoranthaperuviana*, *Paraphoranthadimidiata*, *Paraphoranthapollinosa* and *Paraphoranthaauricaudata* with *Hyalomyiachilensis*, redescription, distribution.

***curvipes*** (Aldrich, 1934).—Neotropical: South America (Argentina, Chile).

*Hyalomyiacurvipes* Aldrich, 1934: 16. Holotype, unspecified sex [male, examined by DMW] (NHMUK). Type locality: Chile, Metropolitana de Santiago, Santiago, Santiago.

Note: The type locality of *Hyalomyiacurvipes* was given as “Santiago” in Chile, which could be interpreted as either the city or province of that name. Cortés and Hichins (1969: 37) cited the former as the type locality (as “Santiago (Santiago)”) and we follow this interpretation.

Reference: Cortés (1948: 122), first record from Argentina.

***glauca*** (Aldrich, 1934).—Neotropical: South America (Argentina, Chile).

*Hyalomyiaglauca* Aldrich, 1934: 15. Holotype male (USNM). Type locality: Argentina, Río Negro, San Carlos de Bariloche [as “Bariloche”].

Reference: Cortés (1946: 173), first record from Chile.

***metallica*** (Aldrich, 1934).—Neotropical: South America (Chile).

*Hyalomyiametallica* Aldrich, 1934: 15. Holotype female (NHMUK). Type locality: Chile, Los Lagos, Llanquihue, Casa Pangue.

### Tribe STRONGYGASTRINI

#### Genus *STRONGYGASTER* Macquart, 1834

***STRONGYGASTER*** Macquart, 1834: 75 [also 1834: 211]. Type species: *Tachinaglobula* Meigen, 1824, by monotypy [Europe].

*CLISTOMORPHA* Townsend, 1892a: 79. Type species: *Clistomorphahyalomoides* Townsend, 1892 (= *Hyalomyiatriangulifera* Loew, 1863), by original designation [United States].

*HYALOMYODES* Townsend, 1893: 429. Type species: *Hyalomyodesweedii* Townsend, 1893 (= *Hyalomyiatriangulifera* Loew, 1863), by monotypy [United States].

*HYALOMYIODES*. Incorrect subsequent spelling of *Hyalomyodes* Townsend, 1893 (Verbeke (1962: 118).

References: Coquillett (1910: 525, 553), type species of *Clistomorpha* (as synonym of *Eliozeta* Rondani, 1856) and *Hyalomyodes*; Curran (1927: 297), key, synonymy of *Hyalomyodes* with *Clistomorpha*; Aldrich (1934: 2, 17), in key to Patagonian genera, taxonomic notes (as *Clistomorpha* with *Hyalomyodes* in synonymy); Townsend (1936b: 80), diagnosis of adults and immatures of Strongygastrini and key to genera (including *Clistomorpha*, *Hyalomyodes* and *Strongygaster*); Townsend (1938: 200, 201, 205), redescriptions of *Clistomorpha*, *Hyalomyodes* and *Strongygaster*; Brooks (1942: 140, 142), revisions of *Clistomorpha* and *Hyalomyodes*; Cortés (1986: 142), in key to tachinid genera of Aysén and Magallanes regions (as *Hyalomyodes*); Wood (1987: 1260), synonymy of *Clistomorpha* and *Hyalomyodes* with *Strongygaster*; O’Hara and Wood (1998: 754, 755, 766), review of synonymy of Wood (1987).

***triangulifera*** (Loew, 1863).—Neotropical: Middle America (Mexico), South America (Argentina, Chile). Nearctic: Canada, United States.

*Hyalomyiatriangulifera* Loew, 1863: 319. Type(s), female [not male as published] (2 females in MCZ). Type locality: USA, New York.

*Clistomorphahyalomoides* Townsend, 1892a: 80. Holotype female [not male as published, Townsend 1938: 200 and verified by DMW] (SEMC, Byers et al. 1962: 173 [as male, in error]). Type locality: USA, New York, Ithaca.

*Hyalomyodesweedii* Townsend, 1893: 430. Lectotype male (SEMC, Byers et al. 1962: 174 [as “5 ♂? syntypes”]), by fixation of Townsend (1938: 201) (mention of “Ht male” from Hanover in SEMC is regarded as a lectotype fixation for the single male syntype from that locality). Type locality: USA, New Hampshire, Hanover.

*triangulifer*. Incorrect subsequent spelling of *triangulifera* Loew, 1863 (e.g., Cortés and Hichins 1969: 37; Guimarães 1971: 18; González 1992b: 179).

Note: The mention of a “Ht” for *Hyalomyiatriangulifera* from New York in MCZ by Townsend (1938: 201) is not accepted as a lectotype fixation because the specimen in question is not distinguishable from the other specimen in the type series.

References: Aldrich (1934: 18), synonymy, redescription, first record from Chile; Verbeke (1962: 118, pl. XIV fig. 9), description and figure of male terminalia; Cortés (1979: 79), taxonomic notes, first record from Argentina; O’Hara and Wood (2004: 306), synonymy including *Clistomorphahyalomoides* with *Hyalomyiatriangulifera*.

### Subfamily TACHININAE

#### Tribe ERNESTIINI

##### Genus *LINNAEMYA* Robineau-Desvoidy, 1830

References: Coquillett (1910: 515, 561, 565, 569), type species of *Bonnetia*, *Linnaemya*, *Marshamia* and *Micropalpis* (with *Marshamia* and *Micropalpis* in synonymy with *Bonnetia*); Townsend (1936b: 190, 197), diagnosis of adults and immatures of Cuphoceratini and key to genera (including *Marshamia*), diagnosis of adults and immatures of Linnaemyini and key to genera (including *Bonnetia*, *Gymnochaetopsis* and *Linnaemya*); Townsend (1936c: 106, 277), diagnosis of adults and immatures of Phoroceratini and key to genera (*Ophina* omitted from key), “*Micropalpus*” as synonym of *Bonnetia*; Townsend (1939a: 198, 221, 232, 238), redescriptions of *Marshamia*, *Bonnetia* (with “*Micropalpus*” in synonymy), *Gymnochaetopsis* and *Linnaemya*; Townsend (1940a: 140), redescription of *Ophina*.

#### Subgenus LINNAEMYA Robineau-Desvoidy, 1830

***LINNAEMYA*** Robineau-Desvoidy, 1830: 52. Type species: *Linnaemyasilvestris* Robineau-Desvoidy, 1830 (= *Tachinavulpina* Fallén, 1810), by subsequent designation of Robineau-Desvoidy (1863a: 131) (as *vulpina*, with *silvestris* in synonymy) [France].

*BONNETIA* Robineau-Desvoidy, 1830: 55. Type species: *Bonnetiaoenanthis* Robineau-Desvoidy, 1830 (= *Tachinacomta* Fallén, 1810), by subsequent designation of Townsend (1916b: 6) [France].

*MARSHAMIA* Robineau-Desvoidy, 1830: 57. Type species: *Marshamiaanalis* Robineau-Desvoidy, 1830 (junior secondary homonym of *Linnaemyaanalis* Robineau-Desvoidy, 1830; = *Tachinacomta* Fallén, 1810), by subsequent designation of Townsend (1916b: 7) [United States].

*MICROPALPIS* Macquart, 1834: 180 [also 1834: 316]. Type species: *Tachinavulpina* Fallén, 1810, by subsequent designation of d’Orbigny (1846: 200, as “*Micropalpus*”) (see Evenhuis and Thompson 1990: 237, as “*Micropalpus*”) [Sweden].

*LINNEMYIA* Macquart, 1835: 81. Unjustified emendation of *Linnaemya* Robineau-Desvoidy, 1830 (see Evenhuis et al. 2010: 100).

*LINNAEMYIA* Aldrich, 1905: 451. Unjustified emendation of *Linnaemya* Robineau-Desvoidy, 1830 (see Evenhuis et al. 2010: 100).

*MICROPALPUS*. Incorrect subsequent spelling of *Micropalpis* Macquart, 1834 (Macquart 1835: 80).

References: Cortés and Campos (1971: 24, 1974: 114) and Cortés (1984: 380), in keys to tachinid genera of Tarapacá and Antofagasta regions (as *Bonnetia* Robineau-Desvoidy).

***comta*** (Fallén, 1810).—Neotropical: Middle America (Honduras, Mexico), South America (Chile, Peru). Nearctic: Canada, United States. Palaearctic: Central Asia, China [Pal.], Europe, Kazakhstan, Korean Peninsula, Middle East, Russia, Transcaucasia. Oriental: China [Orien.], Taiwan. Misidentified from the Afrotropical Region (O’Hara and Cerretti 2016: 193–194).

*Tachinacomta* Fallén, 1810: 277. Lectotype female (NHRS), by fixation of Townsend (1939a: 222) (mention of “Ht male” from Sweden in NHRS is regarded as a lectotype fixation of the single type specimen, a female, in NHRS; examined by JEOH). Type locality: Sweden.

*Linnaemyadistincta* Robineau-Desvoidy, 1830: 54. Lectotype female (MNHN, see note), by fixation of O’Hara and Wood (2004: 242) (mention of “holotype female” from Philadelphia in MNHN is regarded as a lectotype fixation; examined by DMW). Type locality: USA, Pennsylvania, Philadelphia.

*Linnaemyaanalis* Robineau-Desvoidy, 1830: 54. Holotype, unspecified sex (MNHN or lost, see note). Type locality: France, Maine-et-Loire, Angers.

*Marshamiaanalis* Robineau-Desvoidy, 1830: 58 (junior secondary homonym of *Linnaemyaanalis* Robineau-Desvoidy, 1830; = *Micropalpuspiceus* Macquart, 1835). Lectotype female (MNHN, see note), by fixation of Townsend (1939a: 198) (mention of “Ht male” from “Carolina” in MNHN is regarded as a lectotype fixation of the single type specimen, a female, in MNHN; examined by DMW). Type locality: USA, “Caroline” (i.e., North and South Carolina).

*Marshamianigripes* Robineau-Desvoidy, 1830: 58. Lectotype female (MNHN, see note), by fixation of O’Hara and Wood (2004: 242) (mention of “holotype female” from “Carolina” in MNHN is regarded as a lectotype fixation; examined by DMW). Type locality: USA, “Caroline” (i.e., North and South Carolina).

*Micropalpuspiceus* Macquart, 1835: 84 (*nomen novum* for *Marshamiaanalis* Robineau-Desvoidy, 1830, see note).

*comta*. Incorrect subsequent spelling of *comta* Fallén, 1810 (Meigen 1824: 262; numerous subsequent authors).

Notes: The relative priority of *Linnaemyaanalis* Robineau-Desvoidy, 1830 and *Marshamiaanalis* Robineau-Desvoidy, 1830, when both are placed in the same genus, was established by Macquart (1835: 84), as the First Reviser (Article 24.2.2 of the *Code*, ICZN 1999). Macquart (1835: 84) gave the junior homonym, *Marshamiaanalis*, the new name *Micropalpuspiceus* Macquart, 1835.

The single specimen of *Tachinacomta* Fallén in NHRS (a female, examined by JEOH), was treated as the holotype by O’Hara and Wood (2004: 241).

The online MNHN database records a female holotype in the Macquart collection for *Linnaemyadistincta* (number MNHN-ED-ED7203, mistakenly recorded as *Micropalpusdistinctus*) based on a holotype determination label that DMW attached to the specimen in 1985. Robineau-Desvoidy did not restrict the name-bearing type to a single specimen and the lectotype fixation of O’Hara and Wood (2004: 242) is accepted here [see Recommendation 73F of the *Code* (ICZN 1999), “Avoidance of assumption of holotype”].

There is no record for *Linnaemyaanalis* Robineau-Desvoidy in the online MNHN database. There are also no records in the database for *Marshamiaanalis* Robineau-Desvoidy and *Marshamianigripes* Robineau-Desvoidy, but the lectotypes of both names are assumed to be in the Muséum based on their examination there by DMW.

References: Giglio-Tos (1894: 482), first record from Mexico; Coquillett (1897: 87), synonymy of *Linnaemyadistincta*, *Linnaemyaanalis*, *Marshamiaanalis*, *Marshamianigripes* and *Micropalpuspiceus* with *Tachinacomta*; Cortés and Campos (1971: 59), head figure, first records from Chile and Peru; Cave (1992: 595), record from Honduras.

#### Subgenus OPHINA Robineau-Desvoidy, 1863

*OPHINA* Robineau-Desvoidy, 1863a: 298. Type species: *Ophinafulvipes* Robineau-Desvoidy, 1863 (= *Tachinapicta* Meigen, 1824), by original designation [France].

*GYMNOCHAETOPSIS* Townsend, 1914a: 15. Type species: *Gymnochaetopsisanalis* Townsend, 1914 (junior secondary homonym of *Linnaemyaanalis* Robineau-Desvoidy, 1830, see note), by original designation (see Evenhuis et al. 2015: 136) [Peru].

Note: The type species of *Gymnochaetopsis*, *G.analis* Townsend, 1914, is a junior secondary homonym of *Linnaemyaanalis* Robineau-Desvoidy, 1830, described from France. It is not renamed while *Linnaemyaanalis* is in synonymy with *Linnaemyacomta* (Fallén, 1810).

References: Thompson (1963a: 433), redescription of *Gymnochaetopsisanalis*, first record from Trinidad; Mesnil (1971: 1006, 1018), synonymy of *Gymnochaetopsis* with *Linnaemya* and treatment of the former as a subgenus; Shima (1986), assignment of *Gymnochaetopsisanalis* to Linnaemya (Ophina).

There are no Chilean species in this subgenus.

### Tribe GRAPHOGASTRINI

Reference: Andersen (1988: 48), treatment of the Graphogastrini, key to Palaearctic genera.

#### Genus *CLASTONEURA* Aldrich, 1934

***CLASTONEURA*** Aldrich, 1934: 26. Type species: *Clastoneurabrevicornis* Aldrich, 1934, by original designation [Argentina].

Note: Wood and Zumbado (2010: 1412) suggested in their note about *Steleoneura* Stein that “apparently, it [*Steleoneura*] is the sister genus of two Chilean genera, *Clastoneura* Aldrich and *Embiomyia* Aldrich; the latter may be congeneric with *Steleoneura*”. *Steleoneura* is a blondeliine genus and we recognise it herein from Chile with *Embiomyia* in synonymy. We have examined males and females of *Clastoneurabrevicornis* in CNC and believe it to be a graphogastrine and not a blondeliine, as similarly interpreted by Guimarães (1971: 171) and Andersen (1988: 48–49, diagnosis and included genera of Graphogastrini).

References: Townsend (1936c: 129), diagnosis of adults and immatures of Actiini and key to genera (including *Clastoneura*); Townsend (1940a: 203), redescription.

**Figure 6.**
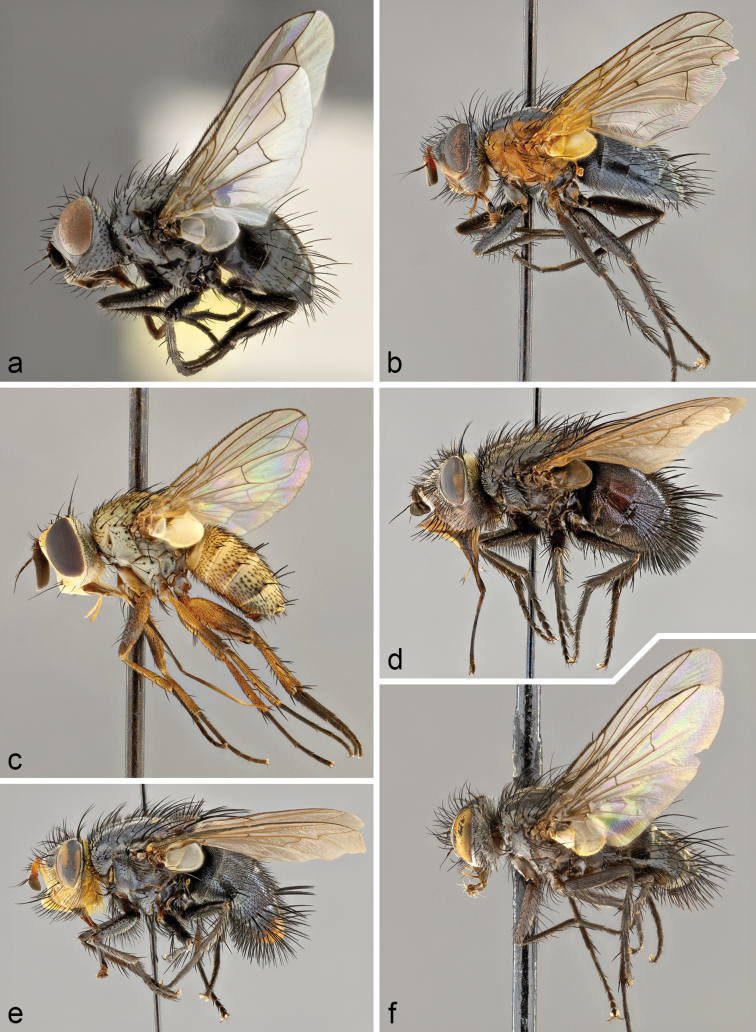
Habitus images **a***Clastoneurabrevicornis* Aldrich ♀ (Tachininae, Graphogastrini) (Chile) [CNC487612], 3.2 mm **b***Xanthopeltascutellaris* Aldrich ♀ (Tachininae, Polideini) (Chile) [CNC487642], 7.6 mm **c**Siphona (Siphona) sp. ♂ (Tachininae, Siphonini) (Chile) [CNC497678], 4.0 mm **d***Chaetoepalpuscoquilleti* Vimmer & Soukup (Tachininae, Tachinini) ♀ (Chile) [CNC1546963], 12.4 mm **e***Chiloepalpuscallipygus* (Bigot) (Tachininae, Tachinini) ♀ (Argentina) [CNC1546964], 12.9 mm **f***Marnefiamirifica* Cortés (Tachinidae, unplaced genus) ♂ (Chile) [CNC1546965], 4.2 mm.

***brevicornis*** Aldrich, 1934.—Neotropical: South America (Argentina, Chile). (Fig. 6a)

*Clastoneurabrevicornis* Aldrich, 1934: 27. Holotype male (NHMUK). Type locality: Argentina, Río Negro, eastern end of Lago Nahuel Huapí.

Reference: Cortés (1967b: 11), taxonomic notes, first record from Chile.

#### Genus *CLASTONEURIOPSIS* Reinhard, 1939

***CLASTONEURIOPSIS*** Reinhard, 1939: 68. Type species: *Clastoneuriopsismeralis* Reinhard, 1939, by original designation [United States].

References: Cortés (1986: 144, 156), in key to tachinid genera of Aysén and Magallanes regions, taxonomic affinities, diagnostic characters; Andersen (1988: 49), assigned to Graphogastrini.

***magallanica*** Cortés, 1986.—Neotropical: South America (Chile).

*Clastoneuriopsismagallanica* Cortés, 1986: 156. Holotype male (MEUC). Type locality: Chile, Magallanes y de la Antártica Chilena, Última Esperanza, Sierra de Los Baguales, 600 m [ca. 50°47′S, 72°24′W].

#### Genus *PHYTOMYPTERA* Rondani, 1845

***PHYTOMYPTERA*** Rondani, 1845: 32, 33. Type species: *Phytomypteranitidiventris* Rondani, 1845 (= *Tachinanigrina* Meigen, 1824), by monotypy [Italy].

*ELFIA* Robineau-Desvoidy, 1849: 158. *Nomen nudum* (no description or included species).

*ELFIA* Robineau-Desvoidy, 1850: 190. Type species: *Actiacingulata* Robineau-Desvoidy, 1830, by subsequent designation of Robineau-Desvoidy (1863a: 672) [France].

*LISPIDEA* Coquillett, 1895b: 51. Type species: *Lispideapalpigera* Coquillett, 1895, by original designation [United States].

*LISPIDEOSOMA* Reinhard, 1943: 164. Type species: *Lispideosomaflavipes* Reinhard, 1943, by original designation [United States].

*CAMPOSODES* Cortés, 1967a: 4. Type species: *Camposodesevanescens* Cortés, 1967, by original designation [Chile]. **Syn. nov.**

*IRWINIA* Cortés, 1967a: 7. Type species: *Irwiniapollinosa* Cortés, 1967, by original designation [Chile].

*LISPIDIA*. Incorrect subsequent spelling of *Lispidea* Coquillett, 1895 (Vimmer and Soukup 1940a: 214).

Note: Cortés (1967a: 4, 7) described *Camposodes* and *Irwinia* as monotypic genera, each characterised by reduced and distinctive wing venation. They are, however, simply apomorphic forms of *Phytomyptera*, a genus in which the loss of wing veins is not unusual and has likely occurred independently in several lineages. Mesnil (1973: 1192) recognised this and synonymised *Irwinia* with *Phytomyptera*. We have examined a specimen of *C.evanescens* in CNC and it is also fundamentally *Phytomyptera*. The diagnostic features of *Phytomyptera* include a haired prosternum, a single setula at the base of wing vein R_4+5_ and lower proepimeral seta directed downward (Andersen 1988: 49; Wood and Zumbado 2010: 1372).

References: Coquillett (1910: 537, 562), type species of *Elfia* (as synonym of *Actia* Robineau-Desvoidy) and *Lispidea*; Aldrich (1934: 4, 5, 75), in key to Patagonian genera, synonymy, taxonomic notes, key to six Patagonian species (as *Lispidea*, in part); Townsend (1936c: 129, 274), diagnosis of adults and immatures of Actiini and key to genera (including *Lispidea* and *Phytomyptera*), *Elfia* as synonym of *Actia* Robineau-Desvoidy, 1830; Townsend (1940a: 232, 248), redescriptions of *Lispidea* and *Phytomyptera*; Sabrosky and Arnaud (1965: 1065), synonymy of *Lispidea* with *Elfia*; Cortés and Campos (1971: 20, 26, 1974: 112, 115, as *Camposodes* and *Lispidea*) and Cortés (1984: 378, 381, as *Camposodes* and *Elfia*), in keys to tachinid genera of Tarapacá and Antofagasta regions; Mesnil (1973: 1192), synonymy of *Irwinia* with *Phytomyptera*; Cortés (1986: 144), in key to tachinid genera of Aysén and Magallanes regions (as *Elfia*); O’Hara (1985: 93), request for type species designation for *Actia* to separate the concepts of *Actia* and *Elfia*; ICZN (1987: 71), type species designation for *Actia*, thereby removing *Actia* as a senior synonym of *Elfia*; Wood (1987: 1220), synonymy of *Elfia*, *Lispidea* and *Lispideosoma* with *Phytomyptera*; Andersen (1988: 45, 49), in key to genera of Graphogastrini, synonymy, diagnosis; O’Hara and Wood (1998: 754, 755, 765), review of synonymy of Wood (1987).

***atra*** (Aldrich, 1934).—Neotropical: South America (Chile).

*Lispideaatra* Aldrich, 1934: 78. Holotype female (USNM). Type locality: Chile, Los Lagos, Llanquihue, Casa Pangue.

Reference: Cortés (1967b: 11), first description of male.

***evanescens*** (Cortés, 1967).—Neotropical: South America (Argentina, Chile). **New record from Argentina. Comb. nov.**

*Camposodesevanescens* Cortés, 1967a: 4. Holotype male (EEAM). Type locality: Chile, Metropolitana de Santiago, Santiago, Maipú, Universidad de Chile, Estación Experimental Agronómica, Quebrada de La Plata, 510–550 m.

Note: The new combination for *Camposodesevanescens* is explained under the genus heading above. The new record from Argentina is based on four CNC specimens from three localities with the following data: [Santa Cruz], southeast of Lago Viedma, ca. 50°S, 72°W, 22.xii.1960, L. Peña (2 specimens); Jujuy, La Quiaca, 23.x.1968, 3500 m, L. Peña (1 specimen); Jujuy, 3 km north of Humahuaca, 3300 m, 22.x.1968, L. Peña (1 specimen).

***frontalis*** (Aldrich, 1934).—Neotropical: South America (Argentina, Chile).

*Lispideafrontalis* Aldrich, 1934: 80. Holotype female (NHMUK). Type locality: Argentina, Tierra del Fuego, Río Grande, Estancia Viamonte.

Reference: Cortés and Campos (1971: 97), first record from Chile.

***interrupta*** (Aldrich, 1934).—Neotropical: South America (Chile).

*Lispideainterrupta* Aldrich, 1934: 79. Holotype female (USNM). Type locality: Chile, Los Lagos, Chiloé, Ancud.

Note: The suggestion by Andersen (1988: 46) that *Lispideainterrupta* “seems more likely belonging in Leskiini” was probably based on misidentified specimens.

***pollinosa*** (Cortés, 1967).—Neotropical: South America (Chile).

*Irwiniapollinosa* Cortés, 1967a: 7. Holotype male (EEAM). Type locality: Chile, Coquimbo, Limarí, Pachingo, Parque Nacional Bosque Fray Jorge.

***triangularis*** (Aldrich, 1934).—Neotropical: South America (Argentina, Chile).

*Lispideatriangularis* Aldrich, 1934: 76. Holotype male (NHMUK). Type locality: Argentina, Río Negro, Lago Correntoso.

Reference: Cortés and Hichins (1969: 41), first record from Chile.

#### Genus *PLANOMYIA* Aldrich, 1934

***PLANOMYIA*** Aldrich, 1934: 129. Type species: *Planomyiabrowni* Aldrich, 1934, by original designation [Chile].

*PLANOMYA*. Incorrect subsequent spelling of *Planomyia* Aldrich, 1934 (Cortés 1967b: 11).

References: Townsend (1936c: 129), diagnosis of adults and immatures of Actiini and key to genera (including *Planomyia*); Townsend (1940a: 249), redescription; Cortés (1986: 144), in key to tachinid genera of Aysén and Magallanes regions; Andersen (1988: 49), in key to genera of Graphogastrini, characters given to separate this genus from the externally similar *Phytomyptera* Rondani.

***browni*** Aldrich, 1934.—Neotropical: South America (Argentina, Chile).

*Planomyiabrowni* Aldrich, 1934: 129. Holotype female (NHMUK). Type locality: Chile, Biobío, Concepción, Concepción.

Note: The type locality of *Planomyiabrowni* was given as “Concepción” in Chile, which could be interpreted as either the city or province of that name. Cortés and Hichins (1969: 53) cited the former as the type locality (as “Concepción (Concepción)”) and we follow this interpretation. Seven paratypes of *P.browni* were collected from “So. Patagonia” by “B. Brown” (Aldrich 1934: 130). The country of origin of these paratypes is interpreted here as Argentina based on the travels of the collector, paleontologist Barnum Brown.

Reference: Cortés (1967b: 11), first description of male.

***vibrissata*** (Aldrich, 1934).—Neotropical: South America (Argentina, Chile).

*Lispideavibrissata* Aldrich, 1934: 78. Holotype male (NHMUK). Type locality: Argentina, Río Negro, Lago Correntoso.

References: Andersen (1988: 46), moved to *Planomyia*; González (1992b: 183), first record from Chile (in *Elfia* Robineau-Desvoidy).

### Tribe LESKIINI

#### Genus *CLAUSICELLA* Rondani, 1856

***CLAUSICELLA*** Rondani, 1856: 61. Type species: *Clausicellasuturata* Rondani, 1856 (as “*Claus: Suturata Mihi*”), by original designation (see O’Hara et al. 2011: 61) [Italy].

*SIPHOACTIA* Townsend, 1927a: 212. Type species: *Siphoactiacharapensis* Townsend, 1927, by original designation [Peru]. **Syn. nov.**

Note: It is evident from the holotype of *Siphoactiacharapensis* (examined by DMW) and head figure of *Siphoactiaperegrina* (Cortés and Campos 1971: 68) that these two species are typical members of *Clausicella*, a genus not previously reported from South America. The genus can be recognised in part by the long proboscis and “membrane between lower facial margin and clypeus with pair of convex subtriangular sclerites” (Wood and Zumbado 2010: 1396).

References: Coquillett (1910: 524), type species of *Clausicella*; Townsend (1936c: 129, 146), diagnosis of adults and immatures of Actiini and key to genera (including *Clausicella*), diagnosis of adults and immatures of Siphonini and key to genera (including *Siphoactia*); Townsend (1940a: 204, 290), redescription of *Clausicella* and *Siphoactia*; Cortés and Campos (1971: 25, 1974: 115) and Cortés (1984: 381), *Siphoactia* in keys to tachinid genera of Tarapacá and Antofagasta regions.

*charapensis* (Townsend, 1927).—Not Chile [Peru]. **Comb. nov.**

*Siphoactiacharapensis* Townsend, 1927a: 357. Holotype female (USNM). Type locality: Peru, Cajamarca, Río Charapi [as “Rio Charape”, ca. 5°25′S, 78°59′W].

***peregrina*** (Cortés & Campos, 1971).—Neotropical: South America (Chile). **Comb. nov.**

*Siphoactiaperegrina* Cortés and Campos, 1971: 67. Holotype female (EEAM). Type locality: Chile, Arica y Parinacota, Arica, Valle de Lluta, km 31.

#### Genus *EPICORONIMYIA* Blanchard, 1940

***EPICORONIMYIA*** Blanchard, 1940: 245. Type species: *Epigrimyiamundelli* Blanchard, 1935 (as “*Epigrymiamundelli*”), by original designation [Argentina].

***mundelli*** (Blanchard, 1935).—Neotropical: South America (Argentina, Chile).

*Epigrymiamundelli* Blanchard, 1935: 8. Holotype male (not located). Type locality: Argentina, Santiago del Estero [province or city].

Reference: Cortés (1976: 4), additional characters, first record from Chile.

#### Genus *ORAEOSOMA* Cortés, 1976

***ORAEOSOMA*** Cortés, 1976: 8. Type species: *Oraeosomaproboscideum* Cortés, 1976, by original designation [Chile].

***proboscideum*** Cortés, 1976.—Neotropical: South America (Chile).

*Oraeosomaproboscideum* Cortés, 1976: 10. Holotype male (MEUC). Type locality: Chile, Metropolitana de Santiago, Santiago, Pudahuel.

#### Genus *SPATHIPALPUS* Rondani, 1863

***SPATHIPALPUS*** Rondani, 1863: 20 [also 1864: 20]. Type species: *Spathipalpusphilippii* Rondani, 1863, by subsequent designation of Brauer and Bergenstamm (1893: 44 [also 1893: 132], as “*Spatipalpus* Rdi. Type: *Philippi* Rdi.”) (see O’Hara et al. 2011: 166) [Chile].

*MACROPALPUS* Rondani, 1863: 20 [also 1864: 20]. *Nomen nudum* (proposed in synonymy [with *Spathipalpus* Rondani, 1863] and not made available by subsequent usage before 1961) (see O’Hara et al. 2011: 111).

References: Aldrich (1934: 3, 31), in key to Patagonian genera, taxonomic notes; Townsend (1936c: 62, 277), diagnosis of adults and immatures of Leskiini and key to genera (including *Spathipalpus*), *Spathipalpus* as valid name for *Macropalpus*; Townsend (1940a: 237), redescription of *Spathipalpus* (with *Macropalpus* in synonymy); Cortés (1986: 143), in key to tachinid genera of Aysén and Magallanes regions.

***philippii*** Rondani, 1863.—Neotropical: South America (Argentina, Chile).

*Spathipalpusphilippii* Rondani, 1863: 21 [also 1864: 21]. Lectotype female (probably MZUF or lost), by fixation of Townsend (1939c: 237) (mention of “Ht female” from Valdivia in “Naples or Genoa” is regarded as a lectotype fixation). Type locality: Chile, Los Ríos, Valdivia, Valdivia.

? *Spathipalpusflavifrons* Rondani, 1863: 21 [also 1864: 21]. Type(s), ?male [described as female but possibly male, Aldrich 1934: 32 and Townsend 1939c: 239] (probably MZUF or lost). Type locality: Chile, Los Ríos, Valdivia, Valdivia.

*philipii*. Incorrect subsequent spelling of *philippii* Rondani, 1863 (Henry 1987: 204).

*philippi*. Incorrect subsequent spelling of *philippii* Rondani, 1863 (Brauer and Bergenstamm 1893: 44 [also 1893: 132]).

Note: The relative priority of *Spathipalpusphilippii* Rondani, 1863 and *Spathipalpusflavifrons* Rondani, 1863, when the two are treated as synonyms, was established by Aldrich (1934: 32), as the First Reviser (Article 24.2.2 of the *Code*, ICZN 1999). The synonymy of these two names was questioned by Townsend (1939c: 239), Cortés (1951a: 65) and Guimarães (1971: 118) and has not been conclusively established.

References: Aldrich (1934: 32), taxonomic notes, head figure, first record from Argentina; Cortés (1951a: 62), first description of male.

### Tribe MEGAPROSOPINI

References: Cortés (1945e: 150), key to the three Chilean genera here assigned to the Megaprosopini, treated as genera allied to *Trichoprosopus* Macquart; Cortés (1983), “Trichoprosopini” proposed as the sister group of the New Zealand tribe Proscissionini (as Occisorini) based in particular on the study of *Trichoceronia* Cortés and *Trichoprosopus* Macquart.

#### Genus *STUARDOMYIA* Cortés, 1945

***STUARDOMYIA*** Cortés, 1945e: 157. Type species: *Stuardomyiacrassiseta* Cortés, 1945, by original designation [Chile].

***crassiseta*** Cortés, 1945.—Neotropical: South America (Argentina, Chile).

*Stuardomyiacrassiseta* Cortés, 1945e: 158. Holotype male (USNM). Type locality: Chile, Araucanía, Malleco, Angol.

Reference: Cortés (1980: 105), first record from Argentina.

#### Genus *TRICHOCERONIA* Cortés, 1945

***TRICHOCERONIA*** Cortés, 1945e: 150. Type species: *Trichoceroniathermitana* Cortés, 1945, by original designation [Chile].

*THRICHOCERONIA*. Incorrect subsequent spelling of *Trichoceronia* Cortés, 1945 (González (1992b: 183).

Reference: Cortés (1986: 143), in key to tachinid genera of Aysén and Magallanes regions.

***latifrons*** (Aldrich, 1934).—Neotropical: South America (Argentina, Chile).

*Trichoprosopuslatifrons* Aldrich, 1934: 20. Holotype female (AMNH). Type locality: “South Patagonia” (interpreted as Argentina by Cortés and Hichins 1969: 60).

References: Cortés (1945e: 150), moved to *Trichoceronia* and partial redescription of female holotype; Cortés and Hichins (1969: 60), first record from Chile.

***thermitana*** Cortés, 1945.—Neotropical: South America (Chile).

*Trichoceroniathermitana* Cortés, 1945e: 151. Holotype male (USNM). Type locality: Chile, Araucanía, Malleco, Curacautín, Termas de Río Blanco.

#### Genus *TRICHOPROSOPUS* Macquart, 1844

***TRICHOPROSOPUS*** Macquart, 1844: 70 [also 1844: 227]. Type species: *Trichoprosopusdurvillei* Macquart, 1844, by original designation [Chile].

*THRICHOPROSOPUS*. Incorrect subsequent spelling of *Trichoprosopus* Macquart, 1844 (González 1992b: 183).

*TRICHOPROSOPA*. Incorrect subsequent spelling of *Trichoprosopus* Macquart, 1844 (Cortés 1963: 249, with note “*erratum pro Trichoprosopus*”).

References: Aldrich (1934: 2, 19), in key to Patagonian genera, taxonomic notes; Townsend (1936b: 121), diagnosis of adults and immatures of Trichoprosopini and key to genera (including *Trichoprosopus*); Townsend (1938: 300), redescription; Cortés (1986: 143), in key to tachinid genera of Aysén and Magallanes regions; Stireman et al. (2016: 38), habitus images of *Trichoprosopus* sp.

***durvillei*** Macquart, 1844.—Neotropical: South America (Chile).

*Trichoprosopusdurvillei* Macquart, 1844: 71 [also 1844: 228]. Lectotype male (MNHN, see note), by fixation of Townsend (1931a: 97) (examination of “Male Ht” from “Chile” in MNHN is regarded as a lectotype fixation). Type locality: Chile, Biobío, Concepción, Concepción.

Note: The online MNHN database records a male holotype in the Macquart collection for *Trichoprosopusdurvillei* (number MNHN-ED-ED8374) based on a holotype determination label that DMW attached to the specimen in 1982. Macquart did not restrict the name-bearing type to a single specimen and the lectotype fixation of Townsend (1931a: 97) is accepted here [see Recommendation 73F of the *Code* (ICZN 1999), “Avoidance of assumption of holotype”].

The type locality of *Trichoprosopusdurvillei* was given as “Conception” in Chile, which could be interpreted as either the city or province of that name. Cortés and Hichins (1969: 61) cited the former as the type locality (as “Concepción (Concepción)”) and we follow this interpretation.

References: Aldrich (1934: 20), redescription, taxonomic notes; Cortés (1945e: 154), redescription; Cortés (1963: 249), notes on name-bearing type in MNHN.

### Tribe NEMORAEINI

#### Genus *XANTHOPHYTO* Townsend, 1916

***XANTHOPHYTO*** Townsend, 1916a: 627. Type species: *Nemoraealabis* Coquillett, 1895, by original designation [United States].

References: Aldrich (1934: 4, 82), in key to Patagonian genera, synonymy, taxonomic notes; Townsend (1936b: 203), diagnosis of adults and immatures of Ernestiini and key to genera (including *Xanthophyto*); Townsend (1939a: 269), redescription.

***erythropyga*** (van der Wulp, 1882).—Neotropical: South America (Argentina, Chile).

*Nemoraeaerythropyga* van der Wulp, 1882: 83. Holotype male (RMNH). Type locality: Chile.

References: Aldrich (1934: 83), redescription, first description of female; Cortés (1973a: 101), taxonomic notes; Gramajo (1998: 96), first record from Argentina.

### Tribe POLIDEINI

The concept of the Polideini and the North American members of the tribe were revised by O’Hara (2002). The Neotropical Polideini are not well understood and require significant revision at the generic and specific levels to better classify the fauna along phylogenetic lines and to accommodate numerous new species. The traditional classification of the Polideini is followed here pending a revision of the tribe.

#### Genus *ANDICESA* Koçak & Kemal, 2010

*TRICHOPHOROPSIS* Townsend, 1914a: 11. *Nomen nudum* (see Evenhuis et al. 2015: 267).

*TRICHOPHOROPSIS* Townsend, 1914b: 42 (junior homonym of *Trichophoropsis* Bonaparte, 1854). Type species: *Trichophoropsispuna* Townsend, 1914, by original designation [Peru].

***ANDICESA*** Koçak & Kemal, 2010: 158 (*nomen novum* for *Trichophoropsis* Townsend, 1914).

*ANICESA*. Incorrect subsequent spelling of *Andicesa* Koçak & Kemal, 2010 (Evenhuis et al. 2015: 267).

References: Townsend (1936b: 190), diagnosis of adults and immatures of Cuphoceratini and key to genera (including *Trichophoropsis*), Townsend (1939a: 217), redescription of *Trichophoropsis*; Cortés and Campos (1971: 24, 1974: 114) and Cortés (1984: 380), in keys to tachinid genera of Tarapacá and Antofagasta regions (as *Trichophoropsis*); González (1992a: 55, 63), in key to Chilean genera of “Cuphocerini”, diagnosis, two new species (as *Trichophoropsis*).

***bicolor*** (González, 1992).—Neotropical: South America (Chile).

*Trichophoropsisbicolor* González, 1992a: 64. Holotype male (UMCE). Type locality: Chile, Antofagasta, Antofagasta, Geyser del Tatio.

***coscaroni*** (González, 1992).—Neotropical: South America (Chile).

*Trichophoropsiscoscaroni* González, 1992a: 65. Holotype male (UMCE). Type locality: Chile, Ñuble, Diguillín, Termas de Chillán.

***nitens*** (Townsend, 1914).—Neotropical: South America (Chile, Peru).

*Trichophoropsisnitens* Townsend, 1914b: 44. Syntypes, 3 males (USNM). Type locality: Peru, Junín, La Oroya, 12,250 ft.

Reference: Cortés and Campos (1971: 70), first record from Chile.

***sabroskyi*** (Cortés & Campos, 1971).—Neotropical: South America (Argentina, Chile).

*Trichophoropsissabroskyi* Cortés & Campos, 1971: 71. Holotype male (EEAM). Type locality: Chile, Arica y Parinacota, Arica, Valle de Lluta, Rosario, 352 m (18°26′S, 70°06′W) (coordinates and elevation given on p. 11).

Reference: Cortés (1980: 106), first record from Argentina.

#### Genus *COMOPS* Aldrich, 1934

***COMOPS*** Aldrich, 1934: 40. Type species: *Comopsruficornis* Aldrich, 1934, by original designation [Argentina].

References: Townsend (1936b: 190), diagnosis of adults and immatures of Cuphoceratini and key to genera (including *Comops*), Townsend (1939a: 177), redescription; Cortés (1986: 143), in key to tachinid genera of Aysén and Magallanes regions; González (1992a: 55, 58), in key to Chilean genera of “Cuphocerini”, diagnosis, notes.

***ruficornis*** Aldrich, 1934.—Neotropical: South America (Argentina, Chile).

*Comopsruficornis* Aldrich, 1934: 40. Holotype, unspecified sex [male, examined by DMW] (NHMUK). Type locality: Argentina, Río Negro, eastern end of Lago Nahuel Huapí.

*Enchomyiapenai* Cortés, 1967b: 16 (as “*peñai*”). Holotype female (EEAM). Type locality: Chile, Coquimbo, Choapa, Illapel, Hacienda Illapel, 2500–2800 m.

References: Guimarães (1971: 75), synonymy of *Enchomyiapenai* with *Comopsruficornis*, citing “R. Cortés, *in litt*.”; Cortés (1973a: 99), taxonomic notes.

#### Genus *DELOBLEPHARIS* Aldrich, 1934

***DELOBLEPHARIS*** Aldrich, 1934: 74. Type species: *Deloblepharisnigra* Aldrich, 1934, by original designation [Chile].

References: Townsend (1936b: 218), diagnosis of adults and immatures of Germariini and key to genera (including *Deloblepharis*); Townsend (1939a: 325), redescription; Cortés (1986: 143), in key to tachinid genera of Aysén and Magallanes regions.

***nigra*** Aldrich, 1934.—Neotropical: South America (Argentina, Chile).

*Deloblepharisnigra* Aldrich, 1934: 74. Holotype female (NHMUK). Type locality: Chile, Los Lagos, Llanquihue, Casa Pangue.

Reference: Gramajo (1998: 95), first record from Argentina.

#### Genus *DESANTISODES* Cortés, 1973

***DESANTISODES*** Cortés, 1973a: 102. Type species: *Desantisodesconcinnum* Cortés, 1973, by original designation [Chile].

***concinnum*** Cortés, 1973.—Neotropical: South America (Argentina, Chile).

*Desantisodesconcinnum* Cortés, 1973a: 103. Holotype female (MEUC). Type locality: Chile, Maule, Curicó, Río Vergara, 2000–2300 m.

Reference: Cortés (1976: 5), first description of male, first record from Argentina.

#### Genus *DOLICHOSTOMA* Townsend, 1912

***DOLICHOSTOMA*** Townsend, 1912b: 325. Type species: *Dolichostomaalpina* Townsend, 1912, by original designation [Peru].

*ERIGONOPSIS* Townsend, 1912b: 326. Type species: *Erigonopsisarequipae* Townsend, 1912, by original designation [Peru].

*EPIDOLICHOSTOMA* Townsend, 1927a: 238. Type species: *Epidolichostomaandina* Townsend, 1927, by original designation [Peru].

Note: The relative priority of *Dolichostoma* Townsend, 1912 and *Erigonopsis* Townsend, 1912, when the two are treated as synonyms, was established by Aldrich (1934: 37), as the First Reviser (Article 24.2.2 of the *Code*, ICZN 1999).

References: Aldrich (1934: 3, 37), in key to Patagonian genera, synonymy of *Erigonopsis* with *Dolichostoma*, taxonomic notes; Townsend (1936b: 190), diagnosis of adults and immatures of Cuphoceratini and key to genera (including *Dolichostoma*, *Epidolichostoma* and *Erigonopsis*), Townsend (1939a: 184, 187, 188), redescriptions of *Dolichostoma*, *Epidolichostoma* and *Erigonopsis*; Cortés and Campos (1971: 24, 1974: 114) and Cortés (1984: 380), in keys to tachinid genera of Tarapacá and Antofagasta regions; Guimarães (1971: 77), earliest synonymy we have found of *Epidolichostoma* with *Dolichostoma*; Cortés (1986: 143), in key to tachinid genera of Aysén and Magallanes regions; González (1992a: 55, 58), in key to Chilean genera of “Cuphocerini”, diagnosis, notes.

***arequipae*** (Townsend, 1912).—Neotropical: South America (Chile, Peru).

*Erigonopsisarequipae* Townsend, 1912b: 326. Holotype female (USNM). Type locality: Peru, Arequipa, Arequipa.

Reference: Cortés and Campos (1971: 61), first record from Chile.

***nigricaudum*** (Blanchard, 1963).—Neotropical: South America (Argentina, Chile).

*Erigonopsisnigricauda* Blanchard, 1963: 178. Holotype male (MACN). Type locality: Argentina, Tucumán [province or city].

References: González (1992a: 59), head figure, first record from Chile; Mulieri et al. (2013: 167), notes on type series in MACN.

***puntarenensis*** (Townsend, 1928).—Neotropical: South America (Argentina, Chile).

*Erigonopsispuntarenensis* Townsend, 1928b: 163. Holotype female (USNM). Type locality: Chile, Magallanes y de la Antártica Chilena, Magallanes, Punta Arenas.

References: Aldrich (1934: 38), redescription, head figure, first record from Argentina; Cortés (1986: 149), taxonomic notes.

#### Genus *ERNESTIOPSIS* Townsend, 1931

***ERNESTIOPSIS*** Townsend, 1931d: 454. Type species: *Ernestiopsiserigonopsidis* Townsend, 1931, by original designation [Chile].

References: Aldrich (1934: 51), in synonymy with *Lypha* Robineau-Desvoidy; Townsend (1936b: 203), diagnosis of adults and immatures of Ernestiini and key to genera (including *Ernestiopsis*); Townsend (1939a: 256), redescription.

***erigonopsidis*** Townsend, 1931.—Neotropical: South America (Argentina, Chile).

*Ernestiopsiserigonopsidis* Townsend, 1931d: 454. Holotype male (USNM). Type locality: Chile, Valparaíso, Marga Marga, Bosque Los Perales [as “Perales”, ca. 33°9′S, 71°18′W].

*erygonopsidis*. Incorrect subsequent spelling of *erigonopsidis* Townsend, 1931 (Cortés and Hichins 1969: 41).

Note: *Ernestiopsiserigonopsidis* was treated in *Lypha* Robineau-Desvoidy by Aldrich (1934: 57), Cortés (1946: 176), Cortés and Hichins (1969: 41) and Cortés (1986: 150), and in *Ernestiopsis* by Guimarães (1971: 83), Gramajo (1998: 95) and O’Hara (2002: 10).

References: Aldrich (1934: 57), redescription; Gramajo (1998: 95), first record from Argentina.

#### Genus *GANOPROCTUS* Aldrich, 1934

***GANOPROCTUS*** Aldrich, 1934: 36. Type species: *Ganoproctusargentifer* Aldrich, 1934, by original designation [Argentina].

References: Aldrich (1934: 36), key to the two species; Townsend (1936b: 190), diagnosis of adults and immatures of Cuphoceratini and key to genera (including *Ganoproctus*), Townsend (1939a: 193), redescription; Cortés (1986: 144), in key to tachinid genera of Aysén and Magallanes regions; González (1992a: 55, 59), in key to Chilean genera of “Cuphocerini”, diagnosis, notes.

***argentifer*** Aldrich, 1934.—Neotropical: South America (Argentina, Chile).

*Ganoproctusargentifer* Aldrich, 1934: 36. Holotype male (NHMUK). Type locality: Argentina, Río Negro, Lago Correntoso.

References: Cortés (1967b: 11), first record from Chile; Cortés (1973a: 98), first description of female.

***longicornis*** Aldrich, 1934.—Neotropical: South America (Chile).

*Ganoproctuslongicornis* Aldrich, 1934: 37. Holotype male (USNM). Type locality: Chile, Araucanía, Malleco, Lonquimay, Reserva Nacional Alto Biobío [as “Alto Biobio”, ca. 38°36′S, 70°58′W].

#### Genus *LYGAEOMYIA* Aldrich, 1934

***LYGAEOMYIA*** Aldrich, 1934: 143. Type species: *Lygaeomyiatristis* Aldrich, 1934, by original designation [Argentina].

References: Townsend (1936c: 129), diagnosis of adults and immatures of Actiini and key to genera (including *Lygaeomyia*); Townsend (1940a: 233), redescription.

***tristis*** Aldrich, 1934.—Neotropical: South America (Argentina, Chile).

*Lygaeomyiatristis* Aldrich, 1934: 144. Holotype male (USNM). Type locality: Argentina, Río Negro, Lago Correntoso.

Note: *Lygaeomyiatristis* was recorded from both Argentina and Chile in the original description.

#### Genus *LYPHA* Robineau-Desvoidy, 1830

***LYPHA*** Robineau-Desvoidy, 1830: 141. Type species: *Tachinadubia* Fallén, 1810, by subsequent designation of Robineau-Desvoidy (1863a: 196) [Sweden].

*APOROMYA* Rondani, 1859: 90. Type species: *Tachinadubia* Fallén, 1810, by original designation [Sweden].

*LYPHE*. Incorrect subsequent spelling of *Lypha* Robineau-Desvoidy, 1830 (Coquillett 1910: 563).

Note: Preliminary study of some of the species below suggests they do not belong to the same lineage as the *Lypha* of North America and need to be reclassified under one or more other genera.

References: Coquillett (1910: 509, 563), type species of *Aporomya* and *Lypha* (as “*Lyphe*”, with *Aporomya* in synonymy); Townsend (1936b: 216), diagnosis of adults and immatures of Lyphini and key to genera (including *Lypha*); Townsend (1936c: 271), *Aporomya* as synonym of *Lypha*; Townsend (1939a: 306), redescription of *Lypha* (with *Aporomya* in synonymy); Aldrich (1934: 4, 51), in key to Patagonian genera, synonymy, taxonomic notes, key to Patagonian species; Cortés (1986: 143), in key to tachinid genera of Aysén and Magallanes regions.

***angolensis*** Aldrich, 1934.—Neotropical: South America (Argentina, Chile).

*Lyphaangolensis* Aldrich, 1934: 58. Holotype male (USNM). Type locality: Chile, Araucanía, Malleco, Angol.

Reference: Gramajo (1998: 95), first record from Argentina.

***chaetosa*** Aldrich, 1934.—Neotropical: South America (Argentina, Chile).

*Lyphachaetosa* Aldrich, 1934: 59. Holotype male (USNM). Type locality: Argentina, Río Negro, Lago Nahuel Huapí.

***corax*** Aldrich, 1934.—Neotropical: South America (Argentina, Chile).

*Lyphacorax* Aldrich, 1934: 61. Holotype male (NHMUK). Type locality: Argentina, Río Negro, San Carlos de Bariloche [as “Bariloche”].

***edwardsi*** Aldrich, 1934.—Neotropical: South America (Argentina, Chile).

*Lyphaedwardsi* Aldrich, 1934: 53. Holotype male (NHMUK). Type locality: Argentina, Río Negro, Lago Correntoso.

Reference: Cortés and Hichins (1969: 41), first record from Chile.

***longicornis*** Aldrich, 1934.—Neotropical: South America (Chile).

*Lyphalongicornis* Aldrich, 1934: 62. Holotype male (NHMUK). Type locality: Chile, Los Lagos, Llanquihue, Casa Pangue.

***orbitalis*** Aldrich, 1934.—Neotropical: South America (Argentina, Chile).

*Lyphaorbitalis* Aldrich, 1934: 60. Holotype male (NHMUK). Type locality: Chile, Los Lagos, Llanquihue, Ensenada.

Reference: Gramajo (1998: 95), first record from Argentina.

***ornata*** Aldrich, 1934.—Neotropical: South America (Argentina, Chile). **New record from Chile.**

*Lyphaornata* Aldrich, 1934: 56. Holotype female (NHMUK). Type locality: Argentina, Tierra del Fuego, Río Grande, Estancia Viamonte.

Note: *Lyphaornata* is newly recorded from Chile based on four CNC specimens from two localities in the Ultima Esperanza Province of the Magallanes y de la Antártica Chilena Region, with the following data: “Natales East of Mount Payne”, Laguna Amarga, 200 m, 14–20.xii.1960, L. Peña (CNC1546165–CNC1546167); and “110 km north of Pto Natales”, Laguna Amarga, 28.i.1994, M. Wood (CNC1546168).

***triangulifera*** (Jacobs, 1900).—Neotropical: South America (Argentina, Chile).

*Hystriciatriangulifera* Jacobs, 1900: 107. Holotype female (RBINS). Type locality: Argentina, Tierra del Fuego, Canal Beagle, Puerto Harberton.

References: Aldrich (1934: 54), redescription; Cortés (1968c: 142), first record from Chile, southernmost record of a tachinid in the Americas.

***truncata*** Aldrich, 1934.—Neotropical: South America (Argentina, Chile).

*Lyphatruncata* Aldrich, 1934: 55. Holotype male (NHMUK). Type locality: Argentina, Tierra del Fuego, Lago Yehuin [as “Lake Yuvin”].

Reference: Cortés (1986: 150), first record from Chile.

#### Genus *NOTODERUS* Cortés, 1986

***NOTODERUS*** Cortés, 1986: 150. Type species: *Notoderusmaculatus* Cortés, 1986, by original designation [Chile].

Reference: Cortés (1986: 143), in key to tachinid genera of Aysén and Magallanes regions.

***maculatus*** Cortés, 1986.—Neotropical: South America (Chile).

*Notoderusmaculatus* Cortés, 1986: 150. Holotype male (MEUC). Type locality: Chile, Magallanes y de la Antártica Chilena, Magallanes, northeast of Punta Arenas, Punta Delgada.

#### Genus *OLLACHERYPHE* Townsend, 1927

***OLLACHERYPHE*** Townsend, 1927a: 256. Type species: *Ollacheryphefacialis* Townsend, 1927, by original designation [Peru].

*AEGLOPS* Aldrich, 1934: 47. Type species: *Aeglopsaenea* Aldrich, 1934, by original designation [Argentina].

References: Townsend (1936b: 218), diagnosis of adults and immatures of Germariini and key to genera (including *Aeglops* and *Ollacheryphe*); Townsend (1939a: 310, 347), redescriptions of *Aeglops* and *Ollacheryphe*; Cortés (1945c: 30), synonymy of *Aeglops* with *Ollacheryphe*; Cortés (1984: 380), in key to tachinid genera of Tarapacá and Antofagasta regions; O’Hara (2002: 105), taxonomic notes.

***aenea*** (Aldrich, 1934).—Neotropical: South America (Argentina, Chile).

*Aeglopsaenea* Aldrich, 1934: 47. Holotype male (NHMUK). Type locality: Argentina, Río Negro, Lago Correntoso.

Note: *Aeglopsaenea* was recorded from both Argentina and Chile in the original description.

***facialis*** Townsend, 1927.—Neotropical: South America (Argentina, Brazil, Chile, Peru).

*Ollacheryphefacialis* Townsend, 1927a: 339. Holotype male (USNM). Type locality: Peru, Puno, Ollachea.

*fascialis*. Incorrect subsequent spelling of *facialis* Townsend, 1927 (Cortés and Hichins 1979: 114).

References: Guimarães (1971: 88), first record from Argentina; Cortés and Hichins (1979: 114), first record from Chile; Cortés (1980: 107), first record from Brazil.

#### Genus *TELODYTES* Aldrich, 1934

***TELODYTES*** Aldrich, 1934: 50. Type species: *Telodytesanalis* Aldrich, 1934, by original designation [Argentina].

References: Townsend (1936b: 203), diagnosis of adults and immatures of Ernestiini and key to genera (including *Telodytes*); Townsend (1939a: 268), redescription.

***analis*** Aldrich, 1934.—Neotropical: South America (Argentina, Chile).

*Telodytesanalis* Aldrich, 1934: 50. Holotype male (NHMUK). Type locality: Argentina, Río Negro, Lago Gutiérrez.

Reference: Henry (1987: 205), first record from Chile.

#### Genus *XANTHOPELTA* Aldrich, 1934

***XANTHOPELTA*** Aldrich, 1934: 48. Type species: *Xanthopeltascutellaris* Aldrich, 1934, by original designation [Argentina].

References: Townsend (1936b: 218), diagnosis of adults and immatures of Germariini and key to genera (including *Xanthopelta*); Townsend (1939a: 362), redescription.

***scutellaris*** Aldrich, 1934.—Neotropical: South America (Argentina, Chile). (Fig. 6b)

*Xanthopeltascutellaris* Aldrich, 1934: 49. Holotype female (NHMUK). Type locality: Argentina, Río Negro, San Carlos de Bariloche [as “Bariloche”].

Note: *Xanthopeltascutellaris* was recorded from both Argentina and Chile in the original description.

### Tribe SIPHONINI

Reference: O’Hara (1989), revision of the genera of the Siphonini.

#### Genus *CEROMYA* Robineau-Desvoidy, 1830

***CEROMYA*** Robineau-Desvoidy, 1830: 86. Type species: *Ceromyatestacea* Robineau-Desvoidy, 1830 (= *Tachinabicolor* Meigen, 1824), by subsequent designation of Coquillett (1910: 520) (see Evenhuis et al. 2010: 54) [France].

*CEROMYIA* Agassiz, 1846: 7. Unjustified emendation of *Ceromya* Robineau-Desvoidy, 1830 (see Evenhuis et al. 2010: 54).

*ACTINACTIA* Townsend, 1927a: 248. Type species: *Actinactialutea* Townsend, 1927, by original designation [Brazil].

Note: O’Hara (1989: 63) in his “List of examined, undescribed, species included in *Ceromyasensu stricto*” listed two undescribed species from Chile as “*Ceromya* Chile sp. 1: One male and one female from Magellanes (CNC)” and “*Ceromya* Chile sp. 2: One male from Isla de Chiloe (CNC)”. These species are still undescribed.

References: Coquillett (1910: 520), type species of *Ceromya* (as synonym of *Ceranthia* Robineau-Desvoidy, 1830); Aldrich (1934: 5, 131), in key to Patagonian genera, synonymy, key to four Patagonian species (as *Actia* Robineau-Desvoidy, 1830); Townsend (1936c: 129, 146), diagnosis of adults and immatures of Actiini and key to genera (including *Ceromya*), diagnosis of adults and immatures of Siphonini and key to genera (including *Actinactia*); Townsend (1940a: 200, 274), redescriptions of *Ceromya* and *Actinactia*; O’Hara (1989: 38, 52), in key to genera of the Siphonini, synonymy including *Actinactia* with *Ceromya*, redescription.

***amblycera*** (Aldrich, 1934).—Neotropical: South America (Argentina, Chile).

*Actiaamblycera* Aldrich, 1934: 132. Holotype male (USNM). Type locality: Argentina, Río Negro, San Carlos de Bariloche [as “Bariloche”].

References: Cortés (1967b: 10), first description of female, first record from Chile; O’Hara (1989: 60), moved to *Ceromya* Robineau-Desvoidy.

***cornuta*** (Aldrich, 1934).—Neotropical: South America (Argentina, Chile).

*Actiacornuta* Aldrich, 1934: 131. Holotype male (USNM). Type locality: Chile, Araucanía, Malleco, Angol.

Note: *Actiacornuta* was recorded from both Argentina and Chile in the original description.

Reference: O’Hara (1989: 61), moved to *Ceromya* Robineau-Desvoidy.

#### Genus *SIPHONA* Meigen, 1803

References: Coquillett (1910: 528, 606), type species of *Crocuta* Meigen and *Siphona* (with latter in synonymy with former); Townsend (1936c: 146, 273), diagnosis of adults and immatures of Siphonini and key to genera (including *Phantasiosiphona*, *Siphona* and *Siphonopsis*), *Crocuta* as synonym of *Siphona*; Townsend (1940a: 286, 292, 294), redescriptions of *Phantasiosiphona*, *Siphona* (with *Crocuta* in synonymy) and *Siphonopsis*; O’Hara (1989: 39, 84), in key to genera of the Siphonini, recognition, key to subgenera.

### Subgenus JIMIMYIA Evenhuis, Pont & Whitmore, 2015

*SIPHONOPSIS* Townsend, 1916a: 622 (junior homonym of *Siphonopsis* Agassiz, 1846). Type species: *Siphonaplusiae* Coquillett, 1895, by original designation [United States].

*JIMIMYIA* Evenhuis, Pont & Whitmore, 2015: 249 (*nomen novum* for *Siphonopsis* Townsend, 1916).

Note: O’Hara (1989: 123) in his “List of examined, undescribed, species included in New World Siphona (Siphonopsis)” listed one undescribed species from Chile as “S. (Siphonopsis) sp. N: One male from Ecuador, males and two females from Chile (CNC)”. This species is still undescribed.

Reference: O’Hara (1989: 86, 120), in key to subgenera of *Siphona*, first treatment as subgenus of *Siphona*, redescription (as *Siphonopsis*).

***brasiliensis*** (Townsend, 1929).—Neotropical: South America (Brazil, Chile).

*Siphonopsisbrasiliensis* Townsend, 1929: 374. Lectotype female (USNM), by fixation of O’Hara (1989: 123) (examination of “Holotype female” from São Paulo in USNM is regarded as a lectotype fixation). Type locality: Brazil, São Paulo, Itaquaquecetuba.

Note: Records of *Siphonabrasiliensis* from Chile (e.g., Cortés 1944d: 142; Cortés 1946: 180; Cortés 1948: 123; Cortés and Hichins 1969: 57; Guimarães 1977c: 74) are possibly based on misidentifications (O’Hara 1989: 122).

Reference: Cortés (1944d: 142), first record from Chile.

### Subgenus SIPHONA Meigen, 1803

*CROCUTA* Meigen, 1800: 39. Meigen (1800) suppressed by ICZN (1963: 339).

***SIPHONA*** Meigen, 1803: 281. Type species: *Muscageniculata* De Geer, 1776, by designation under the Plenary Powers of ICZN (1974: 157) [Sweden].

*CROCUTA* Bezzi, 1907: 414. First usage of *Crocuta* (*sensu* Meigen, 1800) as a valid name after Meigen, 1800; no type species designated originally or subsequently (see note).

*PHANTASIOSIPHONA* Townsend, 1915f: 93. Type species: *Phantasiosiphonatropica* Townsend, 1915, by original designation [Mexico].

Notes: The name *Crocuta* Meigen, 1800 became unavailable when the pamphlet of Meigen (1800) was suppressed by ICZN (1963: 339). *Crocuta* became available later when used by Bezzi (1907: 414), as explained in Evenhuis and Pape (2017: 30). This last work cited the type species of *Crocuta* Bezzi, 1907 as *Muscageniculata* De Geer, 1776 by designation of Coquillett (1910: 528) but this is incorrect; Coquillett (1910) designated a type species for *Crocuta* Meigen, 1800 (at the time an available name) not *Crocuta* Bezzi, 1907.

O’Hara (1989: 119) in his “List of examined, undescribed, species included in Siphona (Siphona)” listed one undescribed species from Chile as “S. (Siphona) nr. tropica: One female from Coquimbo, Chile (CNC)”. This species is still undescribed. Stireman et al. (2016) recorded specimens of unidentified (and presumably undescribed) Siphona (Siphona) species from several sites in Chile (Fig. 6c). One specimen of S. (Pseudosiphona Townsend, 1916) was also reported in Stireman et al. (2016: 27) but we have not reexamined it to confirm its subgenus placement; it is in the Stireman collection at Wright State University, Dayton, USA.

References: Aldrich (1934: 5, 108), in key to Patagonian genera, synonymy, taxonomic notes; Cortés and Campos (1971: 22, 1974: 113) and Cortés (1984: 379), in keys to tachinid genera of Tarapacá and Antofagasta regions; O’Hara (1983: 275), synonymy of *Phantasiosiphona* with *Siphona*; Cortés (1986: 144), in key to tachinid genera of Aysén and Magallanes regions; O’Hara (1989: 85, 108), in key to subgenera of *Siphona*, redescription; Stireman et al. (2016: 27, 28, 29, 33, 35), records of unidentified specimens of S. (Pseudosiphona) and S. (Siphona) species.

*geniculata* (De Geer, 1776).—Not Chile [Nearctic (introduced), Palaearctic].

*Muscageniculata* De Geer, 1776: 38.

Note: *Siphonageniculata* is native to the Palaearctic Region and was introduced into North America for biological control purposes (Wilkinson 1984). There are many records of *S.geniculata* from South America but we believe they are based on misidentifications, as suggested by O’Hara (1983: 299). Among the records are those of Aldrich (1934: 109), Cortés (1946: 180), Cortés and Hichins (1969: 57), Cortés and Campos (1971: 98), Cortés (1979: 81), Cortés (1986: 158 [as possible misidentification based on pers. comm. with JEOH]), Avalos (1989: 49 [also citing possibility of misidentification]), González (1992b: 179) and Gramajo (1998: 98). Guimarães (1971: 170) did not recognise *S.geniculata* from America south of United States. We have not seen *S.geniculata* among examined specimens of S. (Siphona) from the Neotropical Region. Recent DNA barcoding of the COI gene for two S. (Siphona) specimens collected by JEOH in Chile in 2015 (CNC497684, CNC497688) confirmed that they are not *S.geniculata*.

***kuscheli*** (Cortés, 1952).—Neotropical: South America (Chile [Juan Fernández Islands]). Not known from mainland Chile.

*Phantasiosiphonakuscheli* Cortés, 1952: 110. Holotype male (MEUC). Type locality: Chile, Valparaíso, Valparaíso, Juan Fernández Islands, Isla Robinson Crusoe [as “Masatierra”], Cerro Alto, 600 m.

Reference: O’Hara (1983: 279), moved to *Siphona* Meigen.

### Tribe TACHININI

Reference: Cortés (1951b: 250), key to Chilean genera of Tachinini with strong setae on the lower parafacial.

#### Genus *ACROCERONIA* Cortés, 1951

***ACROCERONIA*** Cortés, 1951b: 251. Type species: *Acroceroniaelquiensis* Cortés, 1951, by original designation [Chile].

Reference: González (1992a: 56, 57), in key to Chilean genera of “Cuphocerini”, diagnosis, notes.

***elquiensis*** Cortés, 1951.—Neotropical: South America (Chile).

*Acroceroniaelquiensis* Cortés, 1951b: 252. Holotype male (MNNC). Type locality: Chile, Coquimbo, Elqui, Gualliguaica, 600 m.

Reference: Cortés (1951b: 251), in key to Chilean genera of Tachinini with strong setae on the lower parafacial.

#### Genus *AGICUPHOCERA* Townsend, 1915

***AGICUPHOCERA*** Townsend, 1915e: 430. Type species: *Agicuphoceranigra* Townsend, 1915, by original designation [Peru].

References: Townsend (1936b: 190), diagnosis of adults and immatures of Cuphoceratini and key to genera (including *Agicuphocera*), Townsend (1939a: 169), redescription; Cortés and Campos (1974: 116) and Cortés (1984: 382), in keys to tachinid genera of Tarapacá and Antofagasta regions; González (1992a: 56, 57), in key to Chilean genera of “Cuphocerini”, diagnosis, notes.

***nigra*** Townsend, 1915.—Neotropical: South America (Chile, Peru).

*Agicuphoceranigra* Townsend, 1915e: 430. Holotype female (USNM). Type locality: Peru, Lima, Chosica, ca. 2800 ft.

Reference: Cortés and Campos (1974: 116), first record from Chile.

#### Genus *ALLELOMYIA* González, 1992

***ALLELOMYIA*** González, 1992a: 56. Type species: *Allelomyiadiscalis* González, 1992, by original designation [Chile].

***discalis*** González, 1992.—Neotropical: South America (Chile).

*Allelomyiadiscalis* González, 1992a: 56. Holotype male (UMCE). Type locality: Chile, Metropolitana de Santiago, Cordillera, Reserva Nacional de Río Clarillo.

#### Genus *ANDROSOMA* Cortés & Campos, 1971

***ANDROSOMA*** Cortés & Campos, 1971: 50. Type species: *Androsomaperhirsutum* Cortés & Campos, 1971, by original designation [Chile].

References: Cortés and Campos (1971: 24, 1974: 114) and Cortés (1984: 380), in keys to tachinid genera of Tarapacá and Antofagasta regions.

***perhirsutum*** Cortés & Campos, 1971.—Neotropical: South America (Chile).

*Androsomaperhirsutum* Cortés & Campos, 1971: 52. Holotype male (EEAM). Type locality: Chile, Antofagasta, El Loa, Ojo Hécar, 4500 m (23°11′S, 68°01′W) (coordinates given on p. 12, locality as “Ojo Hécar (Láscar)”).

#### Genus *ARCHYTAS* Jaennicke, 1867

Note: Subgenera of *Archytas* Jaennicke are not recognised here because the subgeneric placements of the Neotropical species require more study.

***ARCHYTAS*** Jaennicke, 1867: 392 [also 1868: 84]. Type species: *Archytasbicolor* Jaennicke, 1867 (= *Tachinadiaphana* Fabricius, 1805), by monotypy [Venezuela].

*NEMOCHAETA* van der Wulp, 1888: 38. Type species: *Nemochaetadissimilis* van der Wulp, 1888, by monotypy [Costa Rica].

*TACHINODES* Brauer & Bergenstamm, 1889: 133 [also 1889: 65]. Type species: hereby fixed under Article 70.3.2 of the *Code* (ICZN 1999) as *Juriniametallica* Robineau-Desvoidy, 1830, misidentified as *Muscahystrix* Fabricius, 1775 in the fixation by monotypy of Brauer and Bergenstamm (1889) [United States].

*PARAFABRICIA* Brauer & Bergenstamm, 1894: 612 [also 1895: 76] (as subgenus of *Archytas* Jaennicke, 1867). Type species: hereby fixed under Article 70.3.2 of the *Code* (ICZN 1999) as *Parafabriciaperplexa* Townsend, 1931, misidentified as *Tachinabicolor* Wiedemann, 1830 in the subsequent designation of Coquillett (1910: 513) [Brazil].

*EUFABRICIA* Townsend, 1908: 111. Type species: *Eufabriciaflavicans* Townsend, 1908 (= *Tachinadiaphanus* Fabricius, 1805), by original designation [Brazil].

*PSEUDOARCHYTAS* Townsend, 1915a: 185. Type species: *Pseudoarchytasmarmorata* Townsend, 1915, by original designation [Peru].

*NEOARCHYTAS* Townsend, 1915e: 430. Type species: *Neoarchytasinambarica* Townsend, 1915, by original designation [Peru].

*MAKASINOCERA* Townsend, 1915e: 431. Type species: *Makasinoceraunguis* Townsend, 1915, by original designation [Peru].

*PSEUDOARCHYTOPSIS* Townsend, 1927a: 252. Type species: *Pseudoarchytopsisbrasiliensis* Townsend, 1927 (= *Goniaincerta* Macquart, 1851), by original designation [Brazil].

*PROARCHYTAS* Townsend, 1931c: 351. Type species: *Tachinadaemon* Wiedemann, 1830, by original designation [Brazil].

*MAKASINOCEROPS* Townsend, 1935: 219. Type species: *Makasinoceropsfulviventris* Townsend, 1935 (junior secondary homonym of *Juriniafulviventris* Robineau-Desvoidy, 1830; = *Archytasshannoni* Guimarães, 1960), by original designation [Brazil].

*ITARCHYTAS* Blanchard, 1940: 225. Type species: *Itarchytaspseudodaemon* Blanchard, 1940, by original designation [Argentina].

*ARCHYNEMOCHAETA* Blanchard, 1941: 345. Type species: *Archynemochaetafrenguellii* Blanchard, 1941, by original designation [Argentina].

*ARCHYTODEJEANIA* Blanchard, 1941: 348. Type species: *Archytodejeaniabruchi* Blanchard, 1941, by original designation [Argentina].

*PROARCHYTOIDES* Blanchard, 1941: 365. Type species: *Proarchytoidesgiacomellii* Blanchard, 1941, by original designation [Argentina].

References: Brauer and Bergenstamm (1893: 58 [also 1893: 146], synonymy of *Nemochaeta* and *Tachinodes* with *Archytas*; Coquillett (1897: 141), synonymy including *Parafabricia* with *Archytas*; Coquillett (1910: 509, 574, 584, 611), type species of *Archytas*, *Nemochaeta*, *Parafabricia* and *Tachinodes* (with last three in synonymy with *Archytas*); Curran (1928b–e), revision of New World species, synonymy including *Makasinocera*, *Neoarchytas*, *Proarchytas* and *Pseudoarchytas* with *Archytas* (*Proarchytas* not mentioned by name but type species included in *Archytas*); Aldrich (1934: 5, 133), in key to Patagonian genera, synonymy including *Eufabricia* and *Pseudoarchytopsis* with *Archytas*, redescription; Townsend (1936b: 167, 174), diagnosis of adults and immatures of Tachinini and key to genera (including *Makasinocera*, *Makasinocerops*, *Nemochaeta*, *Neoarchytas*, *Pseudoarchytas* and *Pseudoarchytopsis*), diagnosis of adults and immatures of Dejeaniini and key to genera (including *Archytas*, *Parafabricia* and *Proarchytas*); Townsend (1936c: 275, 282), *Eufabricia* and *Tachinodes* as synonyms of *Archytas*; Townsend (1939a: 46–58, 70–97), redescriptions of the aforementioned genera of Tachinini and Dejeaniini (with *Eufabricia* and *Tachinodes* in synonymy with *Archytas*); Cortés (1944c), notes on Chilean species; Sabrosky (1955), notes on *Archytas* species; Guimarães (1960, 1961a, 1961b, 1963b, 1963c), series of papers revising *Archytas* species, synonymy including *Makasinocerops* with *Archytas* (Guimarães 1960: 116, 122), synonymy including *Archynemochaeta*, *Archytodejeania*, *Itarchytas* and *Proarchytoides* with *Archytas* (Guimarães 1961b: 356); Thompson (1963a: 361), revision of Trinidad species; Cortés and Campos (1971: 27, 1974: 116) and Cortés (1984: 382), in keys to tachinid genera of Tarapacá and Antofagasta regions; Ravlin and Stehr (1984), revision of species from America north of Mexico.

***incasanus*** Townsend, 1912.—Neotropical: South America (Bolivia, Chile, Peru).

*Archytasincasana* Townsend, 1912b: 331. Holotype female (USNM). Type locality: Peru, Piura, Piura.

*incansanus*. Incorrect subsequent spelling of *incasanus* Townsend, 1912 (Silva et al. 2008: 493, 496).

Note: The identity of *Archytasincasanus* is currently confused in the literature with that of *Archytasdivisus* (Walker, 1853), a non-Chilean species. *Archytasdivisus* was treated as a tentative synonym of *A.analis* (Fabricius, 1805) by Guimarães (1961b: 374) but was later recognised as a valid name by the same author (Guimarães 1971: 49), with *A.incasanus* in synonymy. As a result of this synonymy, *A.incasanussensu*Guimarães (1961b: 370) equals *A.divisussensu*Guimarães (1971). Some authors have continued to use the name *A.incasanus*, see references below and additionally Cortés and Campos (1974: 117) [Chile], Cortés (1984: 385) [Chile], Henry (1987: 200) [Chile], Silva et al. (2008: 496) [Brazil], Nihei (2016: 918) [not Colombia, “but is likely to occur in the country”] and Zetina et al. (2018: 31) [Mexico]. Other authors have used the name *A.divisus*; e.g., Terán (1974: 20) [Venezuela], Avalos (1989: 48) [Argentina] and Vergara de Sánchez and Raven (1990: 95) [Peru, det. Cortés]. There are undoubtedly misidentifications of both *A.incasanus* and *A.divisus* throughout the literature and the only countries that can be conclusively recorded for each is Peru for *A.incasanus* and Brazil for *A.divisus*, based on the type localities. Pending further study of these species we conservatively record the distribution of *A.incasanus* as Peru, Chile and Bolivia and for present purposes record *A.divisus* from the other countries from which *A.incasanus* or *A.divisus* has been reported, see aforementioned references plus Curran (1928e: 275) [Peru, Ecuador, Costa Rica, Mexico] and Thompson (1963a: 383) [Trinidad].

References: Etcheverry (1957: 186), first record from Chile; Cortés and Campos (1971: 55), first record from Bolivia, notes on Chilean specimens (as *A.incasanus*).

*incertus* (Macquart, 1851).—Not Chile [Argentina, Brazil, Paraguay, Uruguay].

*Goniaincerta* Macquart, 1851: 152 [also 1851: 179].

Note: *Archytasincertus* was recorded from Chile only once, in a paper on cutworm control in northern Chile by Caltagirone (1953: 88). Although this record was cited much later in the host-parasite catalogue of Guimarães (1977c: 20), *A.incertus* was not mentioned in the Chilean literature after Caltagirone (1953) (e.g., Cortés and Hichins 1969; Cortés and Campos 1971) and is deemed here to have been misidentified from Chile.

***marmoratus*** (Townsend, 1915).—Neotropical: Greater Antilles (Cuba, Haiti, Jamaica, Puerto Rico), eastern Lesser Antilles (Barbados, Grenada, Guadeloupe, Montserrat, Virgin Islands), southern Lesser Antilles (Trinidad & Tobago), Middle America (Costa Rica, El Salvador, Guatemala, Honduras, Mexico, Nicaragua, Panama), South America (Argentina, Bolivia, Brazil, Chile, Colombia, Ecuador, Guyana, Peru, Suriname, Venezuela). Nearctic: United States.

*Pseudoarchytasmarmorata* Townsend, 1915a: 186. Holotype female (USNM). Type locality: Peru, Lima, Chosica (3000 ft according to label data).

*Echinomyiapiliventris* of Coquillett (1897: 142, as “*Archytaspiliventris*”) and Curran (1928c: 222, as “*Archytaspiliventris*”), not van der Wulp, 1883. Misidentification (Sabrosky 1955: 78) (see note).

Note: The distributions of *Archytasmarmoratus* and *A.incertus* (Macquart) are confused in the literature, in part because the name *Echinomyiapiliventris* van der Wulp, that is currently accepted as a synonym of *A.incertus*, had also been used for misidentified specimens of *A.marmoratus* (e.g., Coquillett 1897: 142; Curran 1928c: 222). Authors who followed the concept of Curran (1928c) for *Archytaspiliventris* were using the name in the current sense of *A.marmoratus*. It is likely that the early Argentinian records of “*Archytaspiliventris*” by Blanchard (1935: 12, 1937: 47) and of “*Pseudoarchytopsispiliventris*” by Blanchard (1941: 348, 1963: 165) apply to *A.marmoratus* because both “*Pseudoarchytopsispiliventris*” and “*Pseudoarchytasincerta*” were redescribed in the same work by Blanchard (1963). A closer study of Blanchard’s descriptions is needed to determine with certainty the identities of the species he redescribed.

References: Curran (1928c: 202, 222), in key, redescription (as “*Archytaspiliventris*” with *Pseudoarchytasmarmorata* in synonymy); Sabrosky (1955: 78), modern interpretation of *A.marmoratus*, distribution given as most of the countries listed here with the notable exceptions of Brazil and Argentina; Guimarães (1961a: 168), redescription, distribution including first record from Brazil; Thompson (1963a: 375), redescription; Ravlin and Stehr (1984: 18), redescription; Avalos (1989: 48), first record from Argentina using the name *Archytasmarmoratus* (see note above); Maes (1999: 1605), distribution, references; Nihei (2016: 918), in catalogue of Tachinidae of Colombia.

***nigriventris*** (van der Wulp, 1882).—Neotropical: South America (Argentina, Chile).

*Jurinianigriventris* van der Wulp, 1882: 81 (junior primary homonym of *Jurinianigriventris* Robineau-Desvoidy, 1863). Syntypes, 2 females (RMNH). Type localities: Chile and Argentina.

Note: *Jurinianigriventris* van der Wulp, 1882 is a junior primary homonym of *Jurinianigriventris* Robineau-Desvoidy, 1863, a valid name for a Mexican species of *Jurinia* Robineau-Desvoidy, 1830. Junior primary homonyms are permanently invalid according to Article 70.3.2 of the *Code* (ICZN 1999), but Article 23.9.5 states: “the author must not automatically replace the junior homonym; the case should be referred to the Commission for a ruling under the plenary power and meanwhile prevailing usage of both names is to be maintained [Art. 82]”. Cortes (1944c: 140) suggested that *J.nigriventris* van der Wulp might be a synonym of *Tachinainfirma* Walker, 1849, a name later synonymised by Cortés (1963: 242) with *Juriniascutellata* Macquart, 1844, treated here as *Archytasscutellatus*. In light of this possible synonymy and the instructions of Article 23.9.5, no action is taken at this time to replace the name of the junior homonym *Jurinianigriventris* van der Wulp.

***peruanus*** Curran, 1928.—Neotropical: South America (Bolivia, Chile, Peru).

*Archytasperuanus* Curran, 1928d: 249. Holotype male (USNM). Type locality: Peru, Junín, La Oroya.

References: Guimarães (1961b: 392), redescription, first record from Bolivia; Cortés and Campos (1971: 58), first record from Chile.

***pilifrons*** (Schiner, 1868).—Neotropical: South America (Argentina, Chile).

*Echinomyiapilifrons* Schiner, 1868: 331. Holotype male (NHMW). Type locality: Chile.

*Jurinianudigaena* Brauer, 1898: 500. Lectotype female (NHMUK), by designation of Sabrosky (1955: 83). Type locality: Chile (see note).

*Archytaspollinosus* Curran, 1928d: 251. Holotype male (SDEI, Rohlfien and Ewald 1974: 142). Type locality: Chile.

*Juriniascutellata* of Aldrich (1934: 135), Cortés (1944c: 139), Cortés (1946: 182) and Guimarães (1961a: 170) (all as “*Archytasscutellatus*”), not Macquart, 1844. Misidentification (e.g., Cortés 1963: 247; Cortés and Hichins 1969: 17; Guimarães 1971: 51).

Note: Sabrosky (1955: 83) reported that Aldrich saw three syntypes of *Jurinianudigaena* in the Bigot collection and had written in his notes: “2 are *Archytaspiliventris* V.d.W., the other is ♀ of *Archytaspilifrons* Sch.”. Sabrosky (1955) designated the last as lectotype of *J.nudigaena* and synonymised *J.nudigaena* with *Echinomyiapilifrons*. The lectotype of *J.nudigaena* is from Chile and the two paralectotypes, both males of a second species [*Archytasincertus* (Macquart, 1851), as *Archytaspiliventris* (van der Wulp, 1883) in notes of Aldrich] are from Montevideo in Uruguay (all three examined by DMW). Brauer (1898: 500) cited the type locality of *Jurinianudigaena* as “Chili, Montevideo” but the latter locality applies to the two males of the second species, *Archytasincertus*. The true *Archytaspilifrons* has not been recorded from Uruguay.

References: Aldrich (1929a: 27), taxonomic notes on holotype of *Echinomyiapilifrons*; Aldrich (1934: 135), synonymy of *Archytaspollinosus* with “*Archytasscutellatus*” (misidentification), records from Argentina and Chile (as *A.scutellatus*); Cortés (1963: 247), notes on synonymy and misidentifications.

***platonicus*** Cortés & Campos, 1971.—Neotropical: South America (Chile, Peru).

*Archytasplatonicus* Cortés & Campos, 1971: 58. Holotype male (EEAM). Type locality: Chile, Arica y Parinacota, Arica, Valle de Lluta, km 57.

Reference: Vergara de Sánchez and Raven (1990: 95), first record from Peru.

***scutellatus*** (Macquart, 1844).—Neotropical: South America (Chile).

*Juriniascutellata* Macquart, 1844: 41 [also 1844: 198]. Lectotype female (MNHN, see note), by fixation of Aldrich (1934: 135) (examination of “type” in MNHN is regarded as a lectotype fixation). Type locality: Chile.

*Tachinainfirma* Walker, 1849: 719. Lectotype male (NHMUK), by fixation of Cortés (1963: 242) (examination of “type” from Chile in NHMUK is regarded as a lectotype fixation). Type locality: Chile.

*Juriniaandana* Robineau-Desvoidy, 1863a: 657. Type(s), female (lost, Cortés 1963: 247). Type locality: Chile.

*Echinomyaignobilis* Rondani, 1863: 15 [also 1864: 15]. Type(s), unspecified sex (lost, Cortés 1963: 247). Type locality: Chile.

*Archytaschilensis* Curran, 1928c: 222. Holotype male (USNM). Type locality: Chile, Valparaíso, Valparaíso, Valparaíso.

Note: Macquart (1844: 41) described *Juriniascutellata* from an unspecified number of specimens from “Chili” and “Santa-Fe de Bogota, en Colombie”. The online MNHN database records a female lectotype from Chile in the Macquart collection for *Juriniascutellata* (number MNHN-ED-ED8299) based on a lectotype determination label that DMW attached to the specimen in 1982. This specimen is presumed to be the same one examined earlier by Aldrich (1934: 135, as “type”) and Cortés (1963: 246, as “tipo”). The paralectotypes from Colombia are not listed in the MNHN database and have not been discussed by subsequent authors. They are here presumed to have been misidentified because there is no corroborating evidence that *Archytasscutellatus* occurs in Colombia and it was not listed from Colombia by Nihei (2016).

References: Aldrich (1934: 135), redescription (as *Archytaschilensis*); Cortés (1963: 242, 246), notes on name-bearing types of *Tachinainfirma* (in NHMUK), *Juriniascutellata* (in MNHN) and *Archytaschilensis* (in USNM), synonymy of *Tachinainfirma*, *Juriniaandana*, *Echinomyaignobilis* and *Archytaschilensis* with *Juriniascutellata*.

*seminiger* (Wiedemann, 1830).—Not Chile [Brazil, Colombia].

*Tachinaseminigra* Wiedemann, 1830: 296.

Note: *Archytasseminiger* was described from Brazil and was later recorded from Chile and Colombia by Schiner (1868: 331). Reed (1888: 305) listed *A.seminiger* from Chile but this was likely based on Schiner’s earlier record. *Archytasseminiger* was considered a doubtful species in Chile by Cortés (1944c: 140, 1946: 172) and it has not been reported from Chile since. Reports of *A.seminiger* from Mexico and/or Puerto Rico by such authors as Giglio-Tos (1894: 484), Aldrich (1905: 487), Curran (1928a: 117), Wolcott (1948: 483), Nihei (2016: 918) and Zetina et al. (2018: 32) are presumed to have been based on misidentifications. *Archytasseminiger* was redescribed by Guimarães (1963b: 155).

#### Genus *CHAETOEPALPUS* Vimmer & Soukup, 1940

*CHAETOEPALPUS* Vimmer, 1940: 101. *Nomen nudum* (proposed after 1930 without designation of type species; no included species).

***CHAETOEPALPUS*** Vimmer & Soukup, 1940a: 218. Type species: *Chaetoepalpuscoquilleti* Vimmer & Soukup, 1940, by monotypy [Peru]. **New record from Chile.**

*RUIZIELLA* Cortés, 1951b: 254. Type species: *Ruiziellafrontosa* Cortés, 1951, by original designation [Chile]. **Syn. nov.**

*CHAETOPALPUS*. Incorrect subsequent spelling of *Chaetoepalpus* Vimmer & Soukup, 1940 (Vimmer and Soukup 1940b: 371; Guimarães 1971: 264).

Note: The new synonymy of *Ruiziella* with *Chaetoepalpus* is explained under *C.coquilleti*.

References: Cortés (1951b: 251), *Ruiziella* in key to Chilean genera of Tachinini with strong setae on the lower parafacial; Cortés and Campos (1971: 26, 1974: 116) and Cortés (1984: 381), *Ruiziella* in keys to tachinid genera of Tarapacá and Antofagasta regions; Stireman et al. (2016: 38), habitus images of *Ruiziella* sp.

***coquilleti*** Vimmer & Soukup, 1940.— Neotropical: South America (Argentina, Chile, Peru). **New records from Argentina and Chile.** (Fig. 6d)

*Chaetoepalpuscoquilleti* Vimmer & Soukup, 1940a: 218. Type(s), unspecified sex (1 female in NMPC, examined by DMW). Type locality: Peru, Puno [region or city].

*Ruiziellafrontosa* Cortés, 1951b: 255. Holotype male (MNNC). Type locality: Chile, Metropolitana de Santiago, Cordillera, Cerro Punta de Damas, 3200 m [ca. 33°31′S, 70°26′W]. **Syn. nov.**

Note: *Chaetoepalpuscoquilleti* was described from an unspecified number of specimens of unspecified sex. One type specimen, a female, was examined in NMPC by DMW in 2005 and matched a conspecific CNC female bearing the following data: Argentina, Jujuy, Río Seco, 5 km south of Station Catalina, 3500 m, 25.x.1968, L. Peña. There are additional specimens in CNC collected by Peña from various localities at high elevations in Jujuy Province of Argentina. There is a series of specimens from Chile in CNC with the following data: Coquimbo, La Laguna, 130 km east of Vicuña, 3200 m, 20.i.1994, G. & M. Wood (including CNC_Diptera162434–CNC_Diptera162436). Images of the specimen from Argentina that was compared to the type of *C.coquilleti* in NMPC and a specimen from Vicuña, Chile, were compared with the types of *R.frontosa* and *R.luctuosa* in MNNC by CRG. The type of *R.frontosa* is a match for *C.coquilleti* (i.e., palpus shorter and postpedicel less broad at apex than in type of *R.luctuosa*), and on this basis *Ruiziella* is synonymised with *Chaetoepalpus* and *R.frontosa* is synonymised with *C.coquilleti*.

The specific epithet “*coquilleti*” is not a misspelling or printer’s error that could be “corrected” to “*coquilletti*” to correspond with the proper spelling of the surname of dipterist Daniel W. Coquillett. Vimmer and Soukup (1940a) mentioned *coquilleti* only once in their paper (p. 218) but mentioned the dipterist’s name as well in the same paper, as “Coquillet” (p. 221), and thus the specific epithet was spelled as intended.

References: Guimarães (1971: 217), *C.coquilleti* listed as an unrecognised species of Tachinidae; Cortés (1979: 81), first record of *R.frontosa* from Argentina.

***luctuosus*** (Cortés, 1951).—Neotropical: South America (Argentina, Chile). **Comb. nov.**

*Ruiziellaluctuosa* Cortés, 1951b: 257. Holotype male (MNNC). Type locality: Chile, Ñuble, Diguillín, Termas de Chillán.

Reference: Cortés (1980: 106), first record from Argentina.

#### Genus *CHILOEPALPUS* Townsend, 1927

***CHILOEPALPUS*** Townsend, 1927c: 281. Type species: *Chiloepalpusaurifacies* Townsend, 1927 (= *Juriniacallipyga* Bigot, 1857), by original designation [Chile].

*EUHELIOPROSOPA* Reinhard, 1964: 123. Type species: *Euhelioprosopapactilis* Reinhard, 1964 (= *Cuphoceraaurea* Aldrich, 1926), by original designation [Chile].

References: Aldrich (1934: 5, 122), in key to Patagonian genera, redescription; Townsend (1936b: 182), diagnosis of Juriniini and key to genera (including *Chiloepalpus*); Townsend (1939a: 108), redescription of *Chiloepalpus*; Cortés (1951b: 250), in key to Chilean genera of Tachinini with strong setae on the lower parafacial; Cortés (1986: 144), in key to tachinid genera of Aysén and Magallanes regions; Cortés (1992: 236), synonymy of *Euhelioprosopa* with *Chiloepalpus*.

***aureus*** (Aldrich, 1926).—Neotropical: South America (Chile).

*Cuphoceraaurea* Aldrich, 1926b: 25. Holotype male (USNM). Type locality: Chile, Araucanía, Malleco, Angol.

*Euhelioprosopapactilis* Reinhard, 1964: 124. Holotype male (CAS). Type locality: Chile, Coquimbo, Elqui, 50 km south of La Serena (misspelled as “LaSorena” in original description, see citation of label data in Arnaud 1979: 400).

References: Aldrich (1934: 124), redescription in *Chiloepalpus*, taxonomic notes; Cortés (1992: 236), synonymy of *Euhelioprosopapactilis* with *Cuphoceraaurea*.

***callipygus*** (Bigot, 1857).—Neotropical: South America (Argentina, Chile).

*Juriniacallipyga* Bigot, 1857: 299. Lectotype female (NHMUK), by fixation of Townsend (1939a: 108) (mention of “Ht female” from Chile in NHMUK [as “Newmarket”] is regarded as a lectotype fixation). Type locality: Chile.

*Chiloepalpusaurifacies* Townsend, 1927c: 281. Holotype female (SDEI, Rohlfien & Ewald 1974: 133). Type locality: Chile, Biobío, Concepción, Concepción.

Note: The type locality of *Chiloepalpusaurifacies* was given as “Concepcion” in Chile, which could be interpreted as either the city or province of that name. Cortés & Hichins (1969: 26) cited the former as the type locality (as “Concepción (Concepción)”) and we follow this interpretation.

Reference: Aldrich (1934: 123), synonymy, redescription, first record from Argentina.

#### Genus *COMOPSIS* Cortés, 1986

***COMOPSIS*** Cortés, 1986: 148. Type species: *Comopsisregale* Cortés, 1986, by original designation [Chile].

References: Cortés (1986: 143), in key to tachinid genera of Aysén and Magallanes regions; González (1992a: 55, 58), in key to Chilean genera of “Cuphocerini”, diagnosis, notes.

***regale*** Cortés, 1986.—Neotropical: South America (Chile).

*Comopsisregale* Cortés, 1986: 148. Holotype male (MEUC). Type locality: Chile, Aysén, General Carrera, 5.8 km west of Chile Chico.

#### Genus *DEOPALPUS* Townsend, 1908

***DEOPALPUS*** Townsend, 1908: 110. Type species: *Deopalpushirsutus* Townsend, 1908, by original designation [Mexico].

*SPANIPALPUS* Townsend, 1908: 110. Type species: *Trichophoramiscelli* Coquillett, 1897, by monotypy [United States].

*PROCYANOPSIS* Townsend, 1934a: 209. Type species: *Procyanopsispictipennis* Townsend, 1934, by original designation [Brazil].

*SPANIPALPIS*. Incorrect subsequent spelling of *Spanipalpus* Townsend, 1908 (Coquillett 1910: 606).

Notes: The relative priority of *Deopalpus* Townsend, 1908 and *Spanipalpus* Townsend, 1908, when the two are treated as synonyms, was established by Sabrosky and Arnaud (1965: 1003), as the First Reviser (Article 24.2.2 of the *Code*, ICZN 1999).

References: Coquillett (1910: 531, 606) type species of *Deopalpus* (as synonym of *Cuphocera* Macquart) and *Spanipalpus* (as “*Spanipalpis*”); Aldrich (1934: 126), as *Cuphocera* Macquart, in part; Townsend (1936b: 190), diagnosis of adults and immatures of Cuphoceratini and key to genera (including *Deopalpus*, *Procyanopsis* and *Spanipalpus*), Townsend (1939a: 183, 207, 212), redescriptions of *Deopalpus*, *Procyanopsis* and *Spanipalpus*; Cortés (1951b: 251), *Spanipalpus* in key to Chilean genera of Tachinini with strong setae on the lower parafacial; Guimarães (1963a: 76), synonymy of *Procyanopsis* with *Deopalpus*; Sabrosky and Arnaud (1965: 1003), synonymy of *Spanipalpus* with *Deopalpus*; Cortés (1967b: 16), key to separate *Spanipalpus*, *Vibrissomyia* Townsend and *Epalpodes* Townsend; Cortés (1984: 382), *Spanipalpus* in key to tachinid genera of Tarapacá and Antofagasta regions; Cortés (1986: 144), *Spanipalpus* in key to tachinid genera of Aysén and Magallanes regions; González (1992a: 56, 59), *Spanipalpus* in key to Chilean genera of “Cuphocerini”, key to species, diagnosis, notes, two new species.

***australis*** (Townsend, 1928).—Neotropical: South America (Argentina, Chile).

*Spanipalpusaustralis* Townsend, 1928b: 164. Holotype female (USNM). Type locality: Chile, Magallanes y de la Antártica Chilena, Magallanes, Punta Arenas.

*Helioprosopafinita* Reinhard, 1964: 121. Holotype male (CAS). Type locality: Chile, Coquimbo, Elqui, 5 miles north of Laguna Dam, 8000 ft.

Note: The records of *Helioprosopafinita* from Mexico and Colombia given in Guimarães (1971: 78) were likely based on misidentifications; see also Nihei (2016: 922).

References: Aldrich (1934: 127), redescription; Reinhard (1934: 57), redescription; Cortés (1992: 236), synonymy of *Helioprosopafinita* with *Spanipalpusaustralis*; Gramajo (1998: 95), first record from Argentina.

***conspiciendum*** (Cortés, 1976).—Neotropical: South America (Chile).

*Spanipalpusconspiciendum* Cortés, 1976: 6. Holotype male (CNC). Type locality: Chile, Biobío, Arauco, Cordillera de Nahuelbuta, Cerro Pichinahuel [ca. 37°48′S, 73°2′W].

***hiemalis*** (Cortés, 1983).—Neotropical: South America (Chile).

*Spanipalpushiemalis* Cortés, 1984: 384. Holotype female (MEUC). Type locality: Chile, Tarapacá, Tamarugal, Zapahuira, ca. 2500 m.

***ochricornis*** (Bigot, 1888).—Neotropical: South America (Chile).

*Epalpusochricornis* Bigot, 1888b: 95. Holotype female (NHMUK). Type locality: Chile.

References: Brauer (1898: 503), taxonomic notes on *Epalpusochricornis*; Aldrich (1930: 26), notes on type of *Epalpusochricornis*; Guimarães (1971: 76), moved to *Deopalpus* based on “R. Cortés, *in litt*.”); Cortés (1976: 6), partial redescription (as *Spanipalpusochricornis*).

***picturatus*** (González, 1992).—Neotropical: South America (Chile).

*Spanipalpuspicturatus* González, 1992a: 61. Holotype male (UMCE). Type locality: Chile, Tarapacá, Tamarugal, 1250 m (located at ca. 20°24′S, 69°56′W, as determined by CRG).

***pulchriceps*** (Aldrich, 1934).—Neotropical: South America (Argentina, Chile).

*Cuphocerapulchriceps* Aldrich, 1934: 128. Holotype male (NHMUK). Type locality: Argentina, Río Negro, San Carlos de Bariloche [as “Bariloche”].

Reference: Cortés and Hichins (1979: 27), first record from Chile.

***rubidus*** (González, 1992).—Neotropical: South America (Chile).

*Spanipalpusrubidus* González, 1992a: 62. Holotype female (UMCE). Type locality: Chile, Magallanes y de la Antártica Chilena, Última Esperanza, Sierra de Los Baguales, 600 m [ca. 50°47′S, 72°24′W].

##### *Nomina dubia* of *DEOPALPUS* Townsend, 1908

***pruinosus*** (Rondani, 1863).—Neotropical: South America (Chile).

*Ciphocerapruinosa* Rondani, 1863: 16 [also 1864: 16]. Type(s), female (probably MZUF or lost). Type locality: Chile.

References: Guimarães (1971: 77), unrecognised species of *Deopalpus*; González (1992a: 61), not included in key to Chilean species of *Spanipalpus*.

***ratzeburgii*** (Jaennicke, 1867).—Neotropical: South America (Chile).

*Demoticusratzeburgii* Jaennicke, 1867: 386 [also 1868: 78]. Type(s), female (SMF). Type locality: Chile.

*ratzeburgi*. Incorrect subsequent spelling of *ratzeburgii* Jaennicke, 1867 (e.g., Cortés and Hichins 1969: 90; Guimarães 1971: 77, 315).

References: Guimarães (1971: 77), unrecognised species of *Deopalpus*; González (1992a: 61), not included in key to Chilean species of *Spanipalpus*.

#### Genus *EDWYNIA* Aldrich, 1930

*REEDIA* Aldrich, 1928b: 17 (junior homonym of *Reedia* Ashmead, 1904). Type species: *Reediarobusta* Aldrich, 1928, by original designation [Chile].

***EDWYNIA*** Aldrich, 1930: 26 (*nomen novum* for *Reedia* Aldrich, 1928).

References: Aldrich (1934: 5, 125), in key to Patagonian genera, synonymy, taxonomic notes; Townsend (1936b: 182), diagnosis of adults and immatures of Juriniini and key to genera (including *Edwynia*); Townsend (1936c: 281), *Edwynia* as valid name for *Reedia*; Townsend (1939a: 116), redescription of *Edwynia* (with *Reedia* in synonymy); Cortés (1951b: 250), in key to Chilean genera of Tachinini with strong setae on the lower parafacial; Cortés (1986: 144), in key to tachinid genera of Aysén and Magallanes regions.

***robusta*** (Aldrich, 1928).—Neotropical: South America (Argentina, Chile).

*Reediarobusta* Aldrich, 1928b: 18. Holotype female (USNM). Type locality: Chile, Biobío, Concepción, Concepción.

Note: The type locality of *Reediarobusta* was given as “Concepcion” in Chile, which could be interpreted as either the city or province of that name. Cortés and Hichins (1969: 32) cited the former as the type locality (as “Concepción (Concepción)”) and we follow this interpretation.

Reference: Aldrich (1934: 126), redescription, first record from Argentina.

#### Genus *EPALPODES* Townsend, 1912

***EPALPODES*** Townsend, 1912b: 330. Type species: *Epalpodesequatorialis* Townsend, 1912, by original designation [Ecuador].

References: Townsend (1936b: 182), diagnosis of adults and immatures of Juriniini and key to genera (including *Epalpodes*); Townsend (1939a: 119), redescription; Cortés (1951b: 251), in key to Chilean genera of Tachinini with strong setae on the lower parafacial; Cortés (1967b: 16), key to separate *Epalpodes*, *Vibrissomyia* Townsend and *Deopalpus* Townsend (as *Spanipalpus* Townsend); Cortés and Campos (1974: 116) and Cortés (1984: 382), in keys to tachinid genera of Tarapacá and Antofagasta regions; González (1992a: 56, 59), in key to Chilean genera of “Cuphocerini”, diagnosis, notes.

***chillanensis*** Cortés, 1951.—Neotropical: South America (Argentina, Chile).

*Epalpodeschillanensis* Cortés, 1951b: 258. Holotype male (MNNC). Type locality: Chile, Ñuble, Diguillín, Termas de Chillán.

Reference: Liljesthröm (1980: 135), first record from Argentina.

***malloi*** Cortés & Campos, 1971.—Neotropical: South America (Chile).

*Epalpodesmalloi* Cortés & Campos, 1971: 62. Holotype male (EEAM). Type locality: Chile, Tarapacá, Tamarugal, Mamiña, 2700 m (20°06′S, 69°16′W) (coordinates given on p. 11).

#### Genus *EPALPUS* Rondani, 1850

***EPALPUS*** Rondani, 1850: 168, 169. Type species: *Micropalpusrufipennis* Macquart, 1846, by subsequent designation of Coquillett (1910: 538) (see O’Hara et al. 2011: 81) [Colombia].

*EUSIGNOSOMA* Townsend, 1914b: 44. *Nomen nudum* (see Evenhuis et al. 2015: 122).

*EUSIGNOSOMA* Townsend, 1914c: 123. Type species: *Eusignosomaaureum* Townsend, 1914, by original designation [Peru].

*ARGENTOEPALPUS* Townsend, 1919a: 178. Type species: *Epalpusniveus* Townsend, 1914, by original designation [Peru].

Note: The name *Eusignosomaaureum* Townsend, 1914, type species of *Eusignosoma* Townsend, is a junior secondary homonym of *Saundersiaaurea* Giglio-Tos, 1893 when the two names are placed together in the genus *Epalpus* Rondani, as in Guimarães (1971: 64). We have assessed the placement of *Saundersiaaurea* and move it to “Unplaced species of Tachinini” herein. See under that heading for further details.

References: Coquillett (1910: 538), type species of *Epalpus*; Townsend (1936b: 182), diagnosis of adults and immatures of Juriniini and key to genera (including *Argentoepalpus*, *Epalpus* and *Eusignosoma*); Townsend (1939a: 105, 120, 128), redescriptions of *Argentoepalpus*, *Epalpus* and *Eusignosoma*; Sabrosky and Arnaud (1965: 1002), synonymy of *Argentoepalpus* with *Epalpus*; Guimarães (1971: 63), synonymy including *Eusignosoma* with *Epalpus*.

***porteri*** Brèthes, 1918.—Neotropical: South America (Chile).

*Epalpusporteri* Brèthes, 1918: 50. Type(s), unspecified sex (1 female in MACN, Mulieri et al. 2013: 169). Type locality: Chile, Valparaíso, Petorca, La Ligua.

References: Cortés (1963: 250), notes on a specimen with data of name-bearing type in MACN (with no mention of “type” and hence not a lectotype fixation); Mulieri et al. (2013: 169), notes on syntype in MACN.

#### Genus *PELETERIA* Robineau-Desvoidy, 1830

Note: Subgenera of *Peleteria* Robineau-Desvoidy are not recognised here because the subgeneric placements of the Neotropical species require more study.

***PELETERIA*** Robineau-Desvoidy, 1830: 39. Type species: *Peleteriaabdominalis* Robineau-Desvoidy, 1830, by subsequent designation of Coquillett (1910: 586) (see Evenhuis et al. 2010: 129) [Italy].

*CUPHOCERA* Macquart, 1845: 267. Type species: *Micropalpusruficornis* Macquart, 1835, by original designation [France].

*PELETERIOPSIS* Townsend, 1916a: 630. Type species: *Echinomyiaflaviventris* van der Wulp, 1888, by original designation [Mexico].

*APHRIOSPHYRIA* Townsend, 1927a: 238. Type species: *Aphriosphyriacommunis* Townsend, 1927 (= *Tachinarobusta* Wiedemann, 1830), by original designation [Brazil].

*CUPHOCEROPSIS* Townsend, 1935: 220. Type species: *Cuphoceropsisfacialis* Townsend, 1935 (= *Echinomyiapygmaea* Macquart, 1851), by original designation [Brazil].

*APHRIOSPHYRIOPSIS* Blanchard, 1943c: 134. Type species: *Aphriosphyriopsisnemochaetoides* Blanchard, 1943, by original designation [Argentina].

*CUPHOCEROMYIA* Blanchard, 1943c: 136. Type species: *Cuphoceromyiaaldrichi* Blanchard, 1943 (junior secondary homonym of *Peleteriaaldrichi* Curran, 1925; = *Peleteriablanchardi* Guimarães, 1971), by original designation [Argentina].

*PROSTEATOSOMA* Blanchard, 1943c: 150. Type species: *Prosteatosomalineata* Blanchard, 1943, by original designation [Argentina].

*APHRYOSPHYRIA*. Incorrect original spelling of *Aphriosphyria* Townsend, 1927 (Townsend 1927a: 287).

*PELETIERIA*. Incorrect subsequent spelling of *Peleteria* Robineau-Desvoidy, 1830 (Thompson 1963a: 341, 412).

Note: There are two original spellings of *Aphriosphyria* in Townsend (1927a): *Aphriosphyria* (p. 238) and *Aphryosphyria* (p. 287). The correct original spelling was selected as *Aphriosphyria* by Townsend (1927b, see entry for “page 287, line 7 [from] top” in the unpaginated errata of Townsend 1927a), as the First Reviser (Article 24.2.3 of the *Code*, ICZN 1999).

References: Coquillett (1910: 529, 586), type species of *Cuphocera* and *Peleteria*; Curran (1925), revision of New World species; Aldrich (1934: 5, 119, 126), in key to Patagonian genera, synonymy including *Aphriosphyria* and *Peleteriopsis* with *Peleteria*, taxonomic notes (as both *Peleteria* and *Cuphocera*); Townsend (1936b: 167, 190), diagnosis of adults and immatures of Tachinini and key to genera (including *Peleteria* and *Peleteriopsis*), diagnosis of adults and immatures of Cuphoceratini and key to genera (including *Aphriosphyria*, *Cuphocera* and *Cuphoceropsis*; Townsend (1939a: 54, 55, 172, 179, 180), redescriptions of *Peleteria*, *Peleteriopsis*, *Aphriosphyria*, *Cuphocera* and *Cuphoceropsis*; Cortés (1951b: 250), in key to Chilean genera of Tachinini with strong setae on the lower parafacial; Guimarães (1962), revision of Brazilian species; Cortés and Campos (1971: 27, 1974: 116) and Cortés (1984: 382), in keys to tachinid genera of Tarapacá and Antofagasta regions; Guimarães (1971: 43), synonymy of *Aphriosphyriopsis*, *Cuphoceromyia*, *Cuphoceropsis* and *Prosteatosoma* with *Peleteria*.

***filipalpis*** (Rondani, 1863).—Neotropical: South America (Argentina, Chile).

*Echinomyafilipalpis* Rondani, 1863: 15 [also 1864: 15]. Type(s), female (probably MZUF or lost). Type locality: Chile (likely the commune of Valdivia in southern Chile [in Los Ríos Region, Valdivia Province] according to Cortés and Campos 1971: 65).

Note: *Peleteriafilipalpis* is restricted here to southern South America following Cortés and Campos (1971: 65).

References: Curran (1925: 257), redescription [but misidentified, see *Peleteriasordida* Aldrich under *P.pygmaea* (Macquart)]; Aldrich (1934: 120), synonymy, redescription, taxonomic notes, first records from Argentina; Cortés and Campos (1971: 65), distribution in Chile and Argentina, comments on type locality of *E.filipalpis*.

***pygmaea*** (Macquart, 1851).—Neotropical: South America (Argentina, Brazil, Chile, Paraguay).

*Echinomyiapygmaea* Macquart, 1851: 143 [also 1851: 170]. Lectotype female (MNHN), by designation herein (see Lectotype Designations section). Type locality: Chile.

*Peleteriasordida* Aldrich, 1934: 122 (named for *Echinomyafilipalpis* of Curran, 1925, not Rondani, 1863). Holotype male (USNM). Type locality: Chile, Araucanía, Malleco, Angol.

*Cuphoceropsisfacialis* Townsend, 1935: 220. Holotype female (USNM). Type locality: Brazil, Pernambuco, Tapéra.

*Echinomyafilipalpis* of Curran (1925: 257, as “*Peleteriafilipalpis*”), not Rondani, 1863. Misidentification (Aldrich 1934: 122).

*pygmaea*. Incorrect subsequent spelling of *pygmaea* Macquart, 1851 (Vergara de Sánchez and Raven 1990: 95).

References: Aldrich (1934: 122), first record from Argentina (as *Peleteriasordida*); Guimarães (1962: 488), redescription (as *Peleteriasordida*), distribution as Argentina, Brazil, Chile and Paraguay; Cortés (1963: 248), *Echinomyiapygmaea* moved to *Peleteria*, synonymy of *P.sordida* with *P.pygmaea*.

***robusta*** (Wiedemann, 1830).—Neotropical: South America (Argentina, Brazil, Chile, Peru, Uruguay).

*Tachinarobusta* Wiedemann, 1830: 290. Lectotype female (NHMW), by fixation of Aldrich (1929a: 28) (examination of female “type” from Montevideo in NHMW is regarded as a lectotype fixation). Type locality: Uruguay, Montevideo, Montevideo.

*Fabriciaandicola* Bigot, 1888b: 86. Holotype female (NHMUK). Type locality: Chile. **Syn. revived.**

*Peleteriarobustamarmorata* Townsend, 1915a: 185. Syntypes, 8 males and 8 females (USNM). Type locality: Peru, Lima, Chosica, ca. 2700–3000 ft.

*Peleteriainca* Curran, 1925: 247. Holotype male (CUIC). Type locality: Peru, Lima, Matucana. **Syn. revived.**

*Aphriosphyriacommunis* Townsend, 1927a: 287 (genus as “*Aphryosphyria*”, see note above under generic synonyms). Syntypes, many males and females (USNM). Type locality: Brazil, São Paulo, Itaquaquecetuba.

Notes: The mention of a “female Ht” of *Aphriosphyriacommunis* by Townsend (1931b: 158) or “Ht female” by Townsend (1939a: 172) is not accepted as a lectotype fixation because the specimen in question is not distinguishable from the other females in the type series.

The distribution of *Peleteriarobusta* is confused in the literature due to numerous misidentifications and is recognised here only from South America. Aldrich (1929a: 28) and Guimarães (1962: 484) treated *P.robusta* as widespread in the Americas with the northern portion of the distribution attributable to their treatment of *Peleteriatexensis* Curran, 1925 as a junior synonym of *P.robusta*. Guimarães (1971: 45) later treated this synonymy as questionable and Richards (1973: 80) confirmed *P.texensis* as a separate species. *Peleteriatexensis* was recorded from United States to Costa Rica by O’Hara and Wood (2004: 322). The South American distribution of *P.robusta* was given as Argentina, Brazil, Chile, Peru and Uruguay by Guimarães (1962: 488) and this distribution is followed here.

*Fabriciaandicola* and *Peleteriainca* were treated as junior synonyms of *Peleteriarobusta* by Guimarães (1962: 484) but were moved into synonymy with *Peleteriafilipalpis* by Guimarães (1971: 44) based on their earlier placement there by Aldrich (1934: 120). However, Aldrich (1934) misinterpreted the type locality of *P.filipalpis* and misidentified the species from the Santiago area according to Cortés and Campos (1971: 65), thereby making the synonymy of Guimarães (1962: 484) more probable than that of Guimarães (1971: 44).

References: Brauer (1898: 495), taxonomic notes on *Fabriciaandicola*; Aldrich (1929a: 28), synonymy (not entirely followed here, see note above), taxonomic notes; Parker (1953: 62), figures of egg, first instar larva and puparium; Guimarães (1962: 484), redescription (see note above); Blanchard (1963: 155), redescription (as *Aphriosphyriarobusta*); Cortés and Campos (1971: 64), distribution of *P.robusta* vs. *P.filipalpis*.

#### Genus *PYRRHOTACHINA* Townsend, 1931

***PYRRHOTACHINA*** Townsend, 1931d: 447. Type species: *Pyrrhotachinaproboscidea* Townsend, 1931, by original designation [Argentina].

References: Townsend (1936b: 190), diagnosis of adults and immatures of Cuphoceratini and key to genera (including *Pyrrhotachina*); Townsend (1939a: 209), redescription; Cortés (1984: 382), in key to tachinid genera of Tarapacá and Antofagasta regions; González (1992a: 55, 59), in key to Chilean genera of “Cuphocerini”, diagnosis.

***proboscidea*** Townsend, 1931.—Neotropical: South America (Argentina, Chile).

*Pyrrhotachinaproboscidea* Townsend, 1931d: 448. Holotype female (SDEI, Rohlfien and Ewald 1974: 142). Type locality: Argentina, Mendoza [province or city].

Reference: Cortés (1984: 387), taxonomic notes, first record from Chile.

#### Genus *SAUNDERSIOPS* Townsend, 1914

***SAUNDERSIOPS*** Townsend, 1914d: 138. Type species: *Saundersiopsconfluens* Townsend, 1914, by original designation [Peru].

*SIGNOEPALPUS* Townsend, 1931d: 446. Type species: *Signoepalpusspinosus* Townsend, 1931, by original designation [Peru].

References: Townsend (1936b: 182), diagnosis of Juriniini and key to genera (including *Saundersiops* and *Signoepalpus*); Townsend (1939a: 152, 153), redescriptions of *Saundersiops* and *Signoepalpus*; Curran (1947: 94), revision, key to species, synonymy of *Signoepalpus* with *Saundersiops*; Cortés (1984: 382), in key to tachinid genera of Tarapacá and Antofagasta regions.

***cruciatus*** Townsend, 1914.—Neotropical: South America (Chile, Peru).

*Saundersiopscruciata* Townsend, 1914d: 140. Holotype female (USNM). Type locality: Peru, Lima, Matucana, ca. 8000 ft.

Reference: Cortés and Hichins (1979: 114), first record from Chile.

#### Genus *STEATOSOMA* Aldrich, 1934

***STEATOSOMA*** Aldrich, 1934: 112. Type species: *Steatosomarufiventris* Aldrich, 1934, by original designation [Argentina].

References: Townsend (1936b: 167), diagnosis of adults and immatures of Tachinini and key to genera (including *Steatosoma*; Townsend (1939a: 63), redescription; Cortés (1951b: 251), in key to Chilean genera of Tachinini with strong setae on the lower parafacial. Cortés (1986: 144), in key to tachinid genera of Aysén and Magallanes regions. González (1992a: 56, 63), in key to Chilean genera of “Cuphocerini”, diagnosis, notes.

***nigriventris*** Aldrich, 1934.—Neotropical: South America (Argentina, Chile).

*Steatosomanigriventris* Aldrich, 1934: 115. Holotype male (NHMUK). Type locality: Argentina, Tierra del Fuego, Lago Yehuin [as “Lake Yuvin”].

*nigripentris*. Incorrect subsequent spelling of *nigriventris* Aldrich, 1934 (Cortés 1963: 246).

References: Cortés (1963: 246), notes on type series in NHMUK; Cortés (1973a: 98), taxonomic notes, first record from Chile.

***rufiventris*** Aldrich, 1934.—Neotropical: South America (Argentina, Chile).

*Steatosomarufiventris* Aldrich, 1934: 112. Holotype male (NHMUK). Type locality: Argentina, Tierra del Fuego, Río Grande, Estancia Viamonte.

Note: *Steatosomarufiventris* was recorded from both Argentina and Chile in the original description.

Reference: Cortés (1963: 246), notes on holotype and paratype in NHMUK.

#### Genus *VIBRISSOMYIA* Townsend, 1912

***VIBRISSOMYIA*** Townsend, 1912b: 327. Type species: *Vibrissomyialineata* Townsend, 1912 (= *Epalpuslineolata* Bigot, 1888), by original designation [Peru].

*VIBRISSOMYA*. Incorrect subsequent spelling of *Vibrissomyia* Townsend, 1912 (Cortés 1980: 106).

References: Townsend (1936b: 190), diagnosis of adults and immatures of Cuphoceratini and key to genera (including *Vibrissomyia*); Townsend (1939a: 218), redescription; Cortés (1951b: 251), in key to Chilean genera of Tachinini with strong setae on the lower parafacial; Cortés (1967b: 16), key to separate *Vibrissomyia*, *Epalpodes* Townsend, and *Deopalpus* Townsend (as *Spanipalpus* Townsend); Cortés and Campos (1971: 26, 1974: 116) and Cortés (1984: 382), in keys to tachinid genera of Tarapacá and Antofagasta regions; Cortés (1986: 144), in key to tachinid genera of Aysén and Magallanes regions; González (1992a: 56, 66), in key to Chilean genera of “Cuphocerini”, key to species, diagnosis, notes, one new species.

***concinnata*** González, 1992.—Neotropical: South America (Chile).

*Vibrissomyiaconcinnata* González, 1992a: 66. Holotype male (UMCE). Type locality: Chile, Ñuble, Diguillín, Termas de Chillán.

***erythrostoma*** (Bigot, 1888).—Neotropical: South America (Chile).

*Epalpuserythrostoma* Bigot, 1888b: 95. Holotype female (NHMUK). Type locality: Chile.

Reference: Brauer (1898: 504), taxonomic notes.

***lineolata*** (Bigot, 1888).—Neotropical: South America (Argentina, Chile, Peru).

*Epalpuslineolatus* Bigot, 1888b: 94. Holotype male (NHMUK). Type locality: Chile.

*Vibrissomyialineata* Townsend, 1912b: 328. Holotype female (USNM). Type locality: Peru, Puno, high puna of Lake Titicaca Region, Tirapata, ca. 13,000 ft.

*Vibrissomyiaalbilineata* Blanchard, 1943c: 152. Syntypes, unspecified number and sex (MLPA). Type locality: Argentina, Santa Cruz, Parque Nacional Los Glaciares, Valle del Río Túnel [as “Valle Tunel, Santa Cruz”, ca. 49°23′S, 72°56′W].

References: Brauer (1898: 503), taxonomic notes on *Epalpuslineolatus*; Cortés (1945c: 29), notes, synonymy of *Vibrissomyialineata* and *Vibrissomyiaalbilineata* with *Epalpuslineolatus*, first records from Argentina and Peru.

***notata*** Cortés, 1967.—Neotropical: South America (Argentina, Chile).

*Vibrissomyianotata* Cortés, 1967b: 14. Holotype male (EEAM). Type locality: Chile, Metropolitana de Santiago, Cordillera, Cajón del Río Maipo, El Yeso, 2200–2500 m.

Reference: Cortés (1979: 81), first record from Argentina.

***pullata*** Cortés, 1951.—Neotropical: South America (Chile).

*Vibrissomyiapullata* Cortés, 1951b: 260. Holotype male (MNNC). Type locality: Chile, Metropolitana de Santiago, Cordillera, El Canelo [ca. 33°35′S, 70°27′W].

#### Unplaced species of Tachinini

*aurea* Giglio-Tos, 1893.—Not Chile [Mexico]. **Comb. nov.**

*Saundersiaaurea* Giglio-Tos, 1893: 3. Type(s), male (MZUT). Type locality: Mexico.

Note: Guimarães (1971: 64) listed *Saundersiaaurea* as an unrecognised species of *Epalpus* Rondani. This species name is a senior secondary homonym of *Eusignosomaaureum* Townsend, 1914 (a Peruvian species) when the two names are placed together in *Epalpus*. Guimarães (1971: 64) was aware of this homonymy but chose not to rename the junior homonym “at present”. DMW examined a male in MZUT labelled as lectotype by P. Arnaud Jr., but unpublished, and called it a “*Trichoepalpus* with bristles on pfc [parafacial]”. This was a preliminary determination in a lineage that has yet to be satisfactorily delineated. We remove *Saundersiaaurea* from *Epalpus* and leave it unplaced in Tachinini until it can be placed to genus with more confidence.

References: Giglio-Tos (1894: 492), redescription, number of specimens in original type series (2 males and 1 ?female) and type localities (Mexico and “Angang” [Mexico, Michoacán, Angangueoven]) given; Guimarães (1971: 64), as unrecognised species of *Epalpus* Rondani; Papavero and Ibáñez-Bernal (2001: 144), notes on type series and current name; Zetina et al. (2018: 33), erroneous listing of *Saundersiaaurea* Giglio-Tos and *Eusignosomaaureum* Townsend as synonyms instead of homonyms with combined distribution of Mexico and Peru (the latter in error).

### Unplaced tribe of Tachinidae

#### Tribe MYIOPHASIINI

The New World tribe Myiophasiini currently comprises nine genera and ca. 40 species, with most species in *Gnadochaeta* Macquart (O’Hara et al. 2020). Myiophasiines are mostly or entirely parasitoids of larval weevils (Curculionidae*s. lato*) (Guimarães 1977; Arnaud 1978) and have been assigned to the Dexiinae by some authors (e.g., Sabrosky and Arnaud 1965; Guimarães 1977) and the Tachininae by others (e.g., Mesnil 1966; Tschorsnig 1985; O’Hara and Wood 2004). Mesnil (1966: 882) treated the group as a basal lineage of Tachininae and the phylogenetic analyses of Cerretti et al. (2014) and Stireman et al. (2019) placed it as an early branch of the Tachinidae, basal to the four recognised subfamilies. It likely shares this basal position with a sister lineage, the Macquartini (Stireman et al. 2019). If this basal lineage is recognised as a subfamily then the name Macquartinae will apply (Sabrosky 1999) but we are hesitant to take this step without further comfirmatory evidence. For the present we regard Myiophasiini as unplaced in the Tachinidae.

##### Genus *GNADOCHAETA* Macquart, 1851

***GNADOCHAETA*** Macquart, 1851: 200 [also 1851: 227] (see note). Type species: *Gnadochaetacoerulea* Macquart, 1851, by original designation [Brazil].

*ANGIORHINA* Brauer & Bergenstamm, 1889: 163 [also 1890: 95]. Type species: *Tachinacrudelis* Wiedemann, 1830, by monotypy [West Indies].

*MYIOPHASIA* Brauer & Bergenstamm, 1891: 362 [also 1891: 58]. Type species: *Tachinaaenea* Wiedemann, 1830 (junior primary homonym of *Tachinaaenea* Meigen, 1824; = *Myiophasiaaustralis* Townsend, 1916), by monotypy [Uruguay].

*PSEUDOCLISTA* Brauer & Bergenstamm, 1893: 104 [also 1893: 192]. Type species: *Pseudoclistaatra* Brauer & Bergenstamm, 1893, by original designation [Brazil].

*ANGIORRHINA*. Incorrect subsequent spelling of *Angiorhina* Brauer & Bergenstamm, 1889 (Guimarães 1971: 23, 253, 269).

*GNADOCHOETA*. Incorrect original spelling of *Gnadochaeta* Macquart, 1851 (Macquart 1851: 200, see note).

Notes: There are two original spellings for *Gnadochaeta* in Macquart (1851): *Gnadochoeta* in the text (pp. 200–201) and index (p. 291) and on plate 21, and *Gnadochaeta* in the figure explanation (p. 286). O’Hara and Wood (2004: 277) were unaware of the latter spelling in the original publication and treated it as an incorrect subsequent spelling in prevailing usage. As explained in Evenhuis et al. (2016: 66): “They [O’Hara and Wood 2004] used ICZN*Code* Art. 33.3.1 to treat *Gnadochaeta* as the original spelling in prevailing usage, but by doing so they implicitly acted as First Reviser in selecting *Gnadochaeta* as the correct original spelling”.

Macquart (1851: 201) noted about his new genus *Gnadochaeta*, “Le type est du Brésil” [“The type is from Brazil”]. This statement is accepted as a type species designation for *Gnadochaeta* of the single included species, *Gnadochaetacoerulea* Macquart, from Brazil.

References: Coquillett (1910: 506, 572), type species of *Angiorhina* and *Myiophasia*; Aldrich (1934: 6, 165), in key to Patagonian genera, synonymy, taxonomic notes (as *Myiophasia*); Townsend (1936b: 116, 121, 124), diagnosis of adults and immatures of Dexillini and key to genera (including *Angiorhina*); diagnosis of adults and immatures of Trichoprosopini and key to genera (including *Gnadochaeta*); diagnosis of adults and immatures of Myiophasiini and key to genera (including *Myiophasia* and *Pseudoclista*); Townsend (1938: 274, 297, 307, 309), redescriptions of *Angiorhina*, *Gnadochaeta*, *Myiophasia* and *Pseudoclista*.

***antennalis*** (Aldrich, 1934).—Neotropical: South America (Argentina, Chile).

*Myiophasiaantennalis* Aldrich, 1934: 167. Holotype male (NHMUK). Type locality: Argentina, Río Negro, Lago Nahuel Huapí, Puerto Blest.

Note: *Myiophasiaantennalis* was recorded from both Argentina and Chile in the original description.

***solitaria*** (Aldrich, 1934).—Neotropical: South America (Chile).

*Myiophasiasolitaria* Aldrich, 1934: 168. Holotype female (USNM). Type locality: Chile, Araucanía, Malleco, Angol.

#### Unplaced genus of Tachinidae

##### Genus *MARNEFIA* Cortés, 1982

***MARNEFIA*** Cortés, 1982: 142. Type species: *Marnefiamirifica* Cortés, 1982, by original designation [Chile].

***mirifica*** Cortés, 1982.—Neotropical: South America (Chile). (Fig. 6f)

*Marnefiamirifica* Cortés, 1982: 143. Holotype (MEUC). Type locality: Chile, Valparaíso, Valparaíso, Viña del Mar, El Salto, Jardín Botánico Nacional.

### Unplaced species of Tachinidae

***casanuevai*** Cortés, 1945.—Neotropical: South America (Chile).

*Phoroceracasanuevai* Cortés, 1945d: 160. Holotype male (MEUC). Type locality: Chile, Valparaíso, Marga Marga, Limache.

References: Cortés (1945d: 159), in key to Chilean species of *Phorocera* Robineau-Desvoidy, 1830 (*s. lato*) and *Parasetigena* Brauer & Bergenstamm; Cortés (1950: 10), in key to Chilean species of *Phorocera* (*s. lato*); Guimarães (1971: 161), listed as an unrecognised species of Exoristini.

***porteri*** Reed, 1907.—Neotropical: South America (Chile).

*Tachinaporteri* Reed, 1907: 1046. Syntypes, males and females (not located). Type locality: Chile, Biobío, Concepción [province].

Note: C.S. Reed (1907: 1046) referred to *Tachinaporteri* as a manuscript name of E.C. Reed (his father, see history section) but gave descriptive details that made the name available from his paper. Brèthes (1910: 67) gave a more formal description of the species under the name “*Exoristaporteri* (Reed) Brèthes”. The name was correctly attributed to C.S. Reed by Cortés and Hichins (1969: 90) but was attributed to Brèthes by Guimarães (1971: 215).

Reference: Guimarães (1971: 215), treated as *Exoristaporteri* Brèthes, 1910 and listed as an unplaced species of Exoristinae (as “Goniinae”).

### *Nomina dubia* of Tachinidae

***albomaculata*** Robineau-Desvoidy, 1863.—Neotropical: South America (Chile).

*Peleteriaalbomaculata* Robineau-Desvoidy, 1863: 622 (as “*albo-maculata*, Macq.”). Type(s), female (MNHN, 1 specimen with number MNHN-ED-ED8298, see note). Type locality: Chile.

Note: Robineau-Desvoidy (1863: 622) noted that this species was listed as *Echinomyaalbo-maculata* Macquart in the catalogue of the Muséum and was labelled as such in the collection. Macquart did not publish a description of it and hence the name *Peleteriaalbomaculata* dates from Robineau-Desvoidy’s (1863: 622) description of it. The single specimen in MNHN is coded with the name “*Echinomyiaalbomaculata* Macquart” and has not been recognised and photographed as the name-bearing type.

References: Cortés (1946: 185), listed under “Species *incertae sedis*” at end of Tachinidae as “*Peleteriaalbomaculata* Macquart [*nomen nudum*]; Robineau-Desvoidy … 1863”; Cortés and Hichins (1969: 90), listed under “Especies excluidas de la lista (*incertae sedis*)” as “*Peleteriaalbomaculata* Macquart *nomen nudum*” followed by “*Peleteriaalbomaculata* Robineau-Desvoidy 1863”; Guimarães (1971: 215), listed as an unrecognised species of Tachinidae.

***lateralis*** Robineau-Desvoidy, 1863.—Neotropical: South America (Chile).

*Faurellalateralis* Robineau-Desvoidy, 1863: 664. Type(s), female (no specimens listed in MNHN database). Type locality: Chile.

References: Cortés (1946: 185), listed under “Species *incertae sedis*” at end of Tachinidae; Cortés and Hichins (1969: 90), listed under “Especies excluidas de la lista (*incertae sedis*)”; Guimarães (1971: 216), listed as an unrecognised species of Tachinidae.

## Lectotype designations

In the interests of nomenclatural stability, DMW designates below four lectotypes for Macquart species based on his examination and labelling of the specimens in the 1980s. The lectotypes are housed in MNHN and their data and images can currently be accessed by searching the Diptera collection database at: https://science.mnhn.fr/institution/mnhn/collection/ed/item/search?lang=en_US.

### *Echinomyiapygmaea* Macquart, 1851: 143 [also 1851: 170].

Described from an unspecified number of females from “Chili” [Chile] collected by “M. Pissis” [Monsieur Pissis; i.e., Pierre Joseph Aimé Pissis, see history section] and deposited in “Muséum” [MNHN].

The online MNHN database records a female holotype in the Macquart collection for *Echinomyiapygmaea* (number MNHN-ED-ED8307) based on a holotype determination label that DMW attached to the specimen in 1980. However, Macquart did not restrict the name-bearing type to a single specimen and no lectotype fixation has been published subsequently. Cortés (1963: 248) examined a single female in MNHN, presumably the same specimen later examined by DMW, but did not explicitly refer to it as a name-bearing type and therefore did not fix it as lectotype.

In the interests of nomenclatural stability and to restrict the name to a single specimen, female syntype MNHN-ED-ED8307 in MNHN is hereby designated by DMW as lectotype of *Echinomyiapygmaea* Macquart, 1851.

The current combination for this species is *Peleteriapygmaea* (Macquart, 1851).

### *Goniachilensis* Macquart, 1844: 50 [also 1844: 207].

Described from an unspecified number of females from “Chili” [Chile] collected by “M. Gay” [Monsieur Gay; i.e., Claude Gay, see history section] and deposited in “Muséum” [MNHN] and from Cuba collected by “M. de la Sagra” [Monsieur de la Sagra; i.e., Ramón de la Sagra] in “Muséum” [MNHN].

The online MNHN database records a female lectotype (from Chile, number MNHN-ED-ED8332) and three female paralectotypes (two from Chile [MNHN-ED-ED8333 and MNHN-ED-ED8334] and one from Cuba [MNHN-ED-ED8335]) for *Goniachilensis* in the Macquart collection based on labels that DMW attached to these specimens in 1982 (the lectotype) and 1985 (the paralectotypes). However, the lectotype designation was not published.

In the interests of nomenclatural stability and to restrict the name to a single specimen, female syntype MNHN-ED-ED8332 from Chile in MNHN is hereby designated by DMW as lectotype of *Goniachilensis* Macquart, 1844.

The current combination for this species is *Goniapallens* Wiedemann, 1830.

### *Masiceraauriceps* Macquart, 1844: 59 [also 1844: 216].

Described from an unspecified number of males from “Brésil ou du Chili” [Brazil or Chile] collected by “M. Gaudichand” [Monsieur Gaudichaud (as “Gaudichand”, typesetter error); i.e., Charles Gaudichaud-Beaupré, see history section] and deposited in “Muséum” [MNHN].

Guimarães (1983: 23) reported that the “type” of *Masiceraauriceps* is “presumably lost” and treated the species as unrecognised. However, the online MNHN database records four male type specimens in the Macquart collection with numbers MNHN-ED-ED8355 to MNHN-ED-ED8358. The database has the first male as lectotype and the other three males as paralectotypes based on labels that DMW attached to these specimens in 1982 (the lectotype) and 1985 (the paralectotypes). However, the lectotype designation was not published. The database has Brazil as the country of origin but we have been unable to verify the restriction of the type locality to either of the two cited countries, Brazil or Chile.

In the interests of nomenclatural stability and to restrict the name to a single specimen, male syntype MNHN-ED-ED8355 in MNHN is hereby designated by DMW as lectotype of *Masiceraauriceps* Macquart, 1844.

The current combination for this species is *Lespesiaauriceps* (Macquart, 1844).

### *Prosopochoetanitidiventris* Macquart, 1851: 184 [also 1851: 211].

Described from an unspecified number of males from “Chili” [Chile] collected by “M. Gay” [Monsieur Gay; i.e., Claude Gay, see history section] and deposited in “Muséum” [MNHN].

Aldrich (1934: 118) reported that the “types” of *Prosopochoetanitidiventris* “apparently are lost”, but this was in error. Townsend (1938: 299) mentioned a “Ht male” for *P.nitidiventris* from Coquimbo in “Lille or lost” but this is not accepted as a lectotype fixation because the specimen in question is not distinguishable from the other males in the type series (in MNHN, not Lille). Cortés (1963: 249) reported examining five (type) specimens in poor condition in MNHN.

The online MNHN database records three male type specimens in the Macquart collection with numbers MNHN-ED-ED8367 to MNHN-ED-ED8369. The database has specimen MNHN-ED-ED8367 as lectotype based on a lectotype label that DMW attached to this specimen in 1982. The other two specimens were labelled as paralectotypes by DMW in 1985. However, the lectotype designation was not published.

In the interests of nomenclatural stability and to restrict the name to a single specimen, male syntype MNHN-ED-ED8367 in MNHN is hereby designated by DMW as lectotype of *Prosopochoetanitidiventris* Macquart, 1851.

The current combination for this species is *Prosopochaetanitidiventris* Macquart, 1851.

## References

[B1] Agassiz L (1846) Nomina systematica generum dipterorum, tam viventium quam fossilium, secundum ordinem alphabeticum disposita, adjectis auctoribus, libris in quibus reperiuntur, anno editionis, etymologia et familiis ad quas pertinent. [Pt. 4], [vi] + 42 pp. In: Agassiz L, Nomenclator zoologicus, continens nomina systematica generum animalium tam viventium quam fossilium, secundum ordinem alphabeticum disposita, adjectis auctoribus, libris, in quibus reperiuntur, anno editionis, etymologia et familias, ad quas pertinent, in singulis classibus. Fasc. IX/X: Titulum et praefationem operis, Mollusca, Lepidoptera, Strepsiptera, Diptera, Myriapoda, Thysanura, Thysanoptera, Suctoria, Epizoa et Arachnidas. Jent & Gassman, Soloduri [= Solothurn, Switzerland]. [before 9 October, Evenhuis 1997: 50] 10.5962/bhl.title.15763

[B2] Aldrich JM (1905) A catalogue of North American Diptera (or two-winged flies). Smithsonian Miscellaneous Collections 46 (2) [= pub. 1444]: 1–680. [before 25 May, Thompson et al. 1999: 365] 10.5962/bhl.title.1681

[B3] Aldrich JM (1924) Notes on some types of American muscoid Diptera in the collection of the Vienna Natural History Museum. Annals of the Entomological Society of America 17: 209–218. [25 July, OS (vol. 17, p. 476)] 10.1093/aesa/17.2.209

[B4] Aldrich JM (1925) Notes on some types of American muscoid Diptera in the collection of the Vienna Natural History Museum. [Concl.] Annals of the Entomological Society of America 18: 456–469. [30 December, OS (vol. 18, p. iv)] 10.1093/aesa/18.4.456

[B5] Aldrich JM (1926a) Notes on muscoid flies (Diptera) with retracted hind crossvein, with key and several new genera and species. Transactions of the American Entomological Society 52: 7–28. [14 April, OS (List of Papers of vol. 52)]

[B6] Aldrich JM (1926b) Descriptions of new and little known Diptera or two-winged flies. Proceedings of the United States National Museum 69 (No. 2648) [1927]: 1–26. [27 December, OS (Table of Contents of vol. 69)] 10.5479/si.00963801.69-2648.1

[B7] Aldrich JM (1927a) Notes on muscoid synonymy. Bulletin of the Brooklyn Entomological Society 22: 18–25. [30 March, OS (wrapper of vol. 22, no. 1)]

[B8] Aldrich JM (1927b) Redescription of types of American muscoid flies in the collection of the Vienna Natural History Museum with incidental notes. Proceedings of the United States National Museum 72 (No. 2703) [1928]: 1–35. [31 December, OS (Table of Contents of vol. 72)] 10.5479/si.00963801.72-2703.1

[B9] Aldrich JM (1928a) A revision of the American parasitic flies belonging to the genus *Belvosia*. Proceedings of the United States National Museum 73 (No. 2729) [1929]: 1–45. [17 May, OS (Table of Contents of vol. 73)] 10.5479/si.00963801.73-2729.1

[B10] Aldrich JM (1928b) New Diptera or two-winged flies from South America. Proceedings of the United States National Museum 74 (No. 2746) [1929]: 1–25. [1 December, OS (Table of Contents of vol. 74)] 10.5479/si.00963801.74-2746.1

[B11] Aldrich JM (1929a) Further studies of types of American muscoid flies in the collection of the Vienna Natural History Museum. Proceedings of the United States National Museum 74 (No. 2764): 1–34. [25 February, OS (Table of Contents of vol. 74)] 10.5479/si.00963801.74-2764.1

[B12] Aldrich JM (1929b) New genera and species of muscoid flies. Proceedings of the United States National Museum 76 (No. 2812) [1930]: 1–13. [16 November, OS (Table of Contents of vol. 76)] 10.5479/si.00963801.76-2812.1

[B13] Aldrich JM (1930) Notes on synonymy of Diptera, No. 4. Proceedings of the Entomological Society of Washington 32: 25–28. [8 March, OS (vol. 32, p. 36)]

[B14] Aldrich JM (1931) Notes on Francis Walker’s types of North American flies of the family Tachinidae. Proceedings of the United States National Museum 80 (No. 2910) [1932]: 1–16. [10 November, OS (Table of Contents of vol. 80)] 10.5479/si.00963801.80-2910.1

[B15] Aldrich JM (1934) Tachinidae. Diptera of Patagonia and South Chile based mainly on material in the British Museum (Natural History). Part VII.—Fascicle 1. British Museum (Natural History), London, 170 pp. [24 March, OS]

[B16] Andersen S (1988) Revision of European species of *Phytomyptera* Rondani (Diptera: Tachinidae). Entomologica Scandinavica 19: 43–80. [20 May, OS] 10.1163/187631289X00050

[B17] Anonymous (1913) A correction. Proceedings of the New York Entomological Society 21: 313. [December, Evenhuis et al. 2015: 310]

[B18] Anonymous (1995) Supplements and Memoirs. The Canadian Entomologist 127: 987–993. [December, Evenhuis et al. 2015: 310]

[B19] Arnaud PH Jr (1958) The entomological publications of Charles Henry Tyler Townsend [1863–1944]; with lists of his new generic and specific names. Microentomology 23: 1–63. [30 May, OS]

[B20] Arnaud PH Jr. (1978) A host-parasite catalog of North American Tachinidae (Diptera). United States Department of Agriculture. Miscellaneous Publication 1319: 1–860. [June, OS]

[B21] Arnaud PH Jr (1979) A catalog of the types of Diptera in the collection of the California Academy of Sciences. Myia 1: v + 505 pp.

[B22] Artigas JN Papavero N (1991) The American genera of Asilidae (Diptera): keys for identification with an atlas of female spermathecae and other morphological details. V11.7. Subfamily Stenopogoninae Hull. Tribe Cyrtopogonini with descriptions of four new genera and one new species. Boletín de la Sociedad de Biología de Concepción 62: 55–81.

[B23] Ashley TR (1979) Classification and distribution of fall armyworm parasites. Florida Entomologist 62: 114–123. [15 June, OS (wrapper of vol. 62, no. 2)] 10.2307/3494087

[B24] Austen EE (1907) The synonymy and generic position of certain species of Muscidae (sens. lat.) in the collection of the British Museum, described by the late Francis Walker. Annals and Magazine of Natural History (Ser. 7) 19: 326–347. [30 March, Evenhuis 2003b: 37] 10.1080/00222930709487273

[B25] Avalos DS (1989) Moscas Tachinidae de la Provincia de Córdoba (Argentina). Revista Peruana de Entomología 31 [1988]: 48–50. [30 June, OS]

[B26] Barendrecht G (1957) A propos du centenaire du *Tijdschrift voor Entomologie*. Tijdschrift voor Entomologie 100: 1–4. [17 April, OS (wrapper of vol. 100, no. 1)]

[B27] Beneway DF (1961) *Androeuryops*, a new genus of Tachinidae (Diptera) from Central America. Journal of the Kansas Entomological Society 34: 44–47. [28 February, OS (wrapper of vol. 34, no. 1)]

[B28] Beneway DF (1963) A revision of the flies of the genus *Lespesia* (= *Achaetoneura*) in North America (Diptera: Tachinidae). University of Kansas Science Bulletin 44: 627–686. [13 September, OS]

[B29] Berry PA (1951) Biology and habits of cotton stainers (Hemiptera: *Dysdercus* spp.), their natural enemies in South America and two parasitic flies imported into Puerto Rico. Revista de Entomologia 22: 329–342. [31 December, OS (vol. 22, p. iv)]

[B30] Berthold AA (1827) Latreille’s Natürliche Familien der Thierreichs. Aus dem Französischen. Mit Anmerkungen und Zusätzen. Landes-Industrie Comptoir, Weimar. x + 606 pp. [This is a German translation of Latreille (1825) (see Evenhuis 1997: 86).] [8 November, Evenhuis 1997: 86] 10.5962/bhl.title.11652

[B31] Bezzi M (1907) Die Gattungen der blutsaugenden Musciden (Dipt.). Zeitschrift für Systematische Hymenopterologie und Dipterologie 7: 413–416. [1 September, Evenhuis and Pape 2019: 154] 10.5962/bhl.title.5144

[B32] Bezzi M Stein P (1907) CyclorrhaphaAschiza. CyclorrhaphaSchizophora: Schizometopa, pp. 1–747. In: Becker T, Bezzi M, Kertész K, Stein P (Eds), Katalog der paläarktischen Dipteren. Band III. Budapest, 828 pp. [17 December, Thompson et al. 1999: 378]

[B33] Bigot JMF (1857a) Diptères nouveaux provenant du Chili. Annales de la Société Entomologique de France (Sér. 3) 5: 277–308 + pls. 6–7. [9 September, Evenhuis 2003a: 6]

[B34] Bigot JMF (1857b) Dipteros, pp. 328–349, pl. 20. In: Sagra DR de la (Ed), Historia fisica, politica y natural de la isla de Cuba. Segunda parte. Historia natural. Tomo VII. Crustaceos, aragnides é insectos. Arthus Bertrand, Paris. xxxii + 371 pp. [after 10 October, Evenhuis 2003a: 6]

[B35] Bigot JMF (1876) Diptères nouveaux ou peu connus. 6^e^ partie. VIII. Curie des Phasides (Phasidae, mihi). G^res^*Trichopoda* (Macq.) et *Bogosia* (Rond.). Annales de la Société Entomologique de France (Sér. 5) 6: 389–400. [27 December, Evenhuis 2003a: 12]

[B36] Bigot JMF (1878) [Diptères nouveaux ou peu connus. 9^e^ partie.] XIII. Genres *Ocyptera* (Latr.), *Ocypterula*, *Exogaster* (Rond.). Annales de la Société Entomologique de France (Sér. 5) 8: 40–47. [26 June, Evenhuis 2003a: 14]

[B37] Bigot JMF (1880) [Diagnose d’un nouveau genre de diptères de la tribu des Tachinidi.] Bulletin Bimensuel de la Société Entomologique de France 1880(7): 69–70. [Also published in 1880, Bulletin de la Société Entomologique de France (Sér. 5) 10: liii.] [24 April (Bulletin Bimensuel), 25 August (Bulletin), Evenhuis 2003a: 17]

[B38] Bigot JMF (1885a) [Diagnoses de 11 genres de diptères exotiques nouveaux.] Bulletin Bimensuel de la Société Entomologique de France 1884(24): 237. [8 January, Evenhuis 2003a: 23]

[B39] Bigot JMF (1885b) [Diagnoses de deux genres nouveaux de diptères du groupe des tachinides.] Bulletin Bimensuel de la Société Entomologique de France 1885 (5): xliv–xlvi. [Also published in 1885, Bulletin de la Société Entomologique de France (Sér. 6) 5: xliv–xlvi.] [20 March (Bulletin Bimensuel), 15 July (Bulletin), Evenhuis 2003a: 24]

[B40] Bigot JMF (1885c) [Diagnoses génériques de deux genres nouveaux de diptères du groupe des tachinides.] Bulletin Bimensuel de la Société Entomologique de France 1885 (5): liv–lvi. [Also published in 1885, Bulletin de la Société Entomologique de France (Sér. 6) 5: liv–lvi.] [20 March (Bulletin Bimensuel), 15 July (Bulletin), Evenhuis 2003a: 24]

[B41] Bigot JMF (1888a) Diptères [sect. v.]. In: Ministères de la Marine et de l’Instruction Publique, Mission scientifique du Cap Horn. 1882–1883. Tome VI. Zoologie. Deuxième partie. Insectes. Gauthier-Villars et Fils, Paris, 45 pp. + 4 pls. [15 February, Evenhuis 2003a: 28]

[B42] Bigot JMF (1888b) Diptères nouveaux ou peu connus. 33^e^ partie. XLI. Tachinidae. Annales de la Société Entomologique de France (Sér. 6) 8: 77–101. [30 June, Evenhuis 2003a: 28]

[B43] Bigot JMF (1891) [Notes diptérologiques.] Bulletin Bimensuel de la Société Entomologique de France 1891 (14): cxxxv–cxxxvi. [Also published in 1891, Bulletin de la Société Entomologique de France, 60: cxxxv–cxxxvi.] [24 October (Bulletin Bimensuel), 23 December (Bulletin), Evenhuis 2003a: 33]

[B44] Blanchard CE (1854) Orden IX. Dipteros, pp. 327–468. In: Gay C (Ed), Historia física y política de Chile según documentos adquiridos en esta república durante doce años de residencia en ella y publicada bajo los auspicios del supremo gobierno. Zoologia. Tomo sétimo. Paris, 471 pp. [Cover of volume bears date of 1852.] [26 April, Evenhuis 2015: 44]

[B45] Blanchard EE (1935) Apuntes sobre dípteros argentinos. Revista Argentina de Entomología 1: 5–12.

[B46] Blanchard EE (1937) Dipteros argentinos nuevos o poco conocidos. Revista de la Sociedad Entomológica Argentina 9: 35–58. [31 December, OS (wrapper of vol. 9, p. 1)]

[B47] Blanchard EE (1940) Apuntes sobre muscoideos argentinos. Anales de la Sociedad Científica Argentina 129: 224–246.

[B48] Blanchard EE (1941) Los dípteros muscoideos del Museo de La Plata. Primera parte: Tachinidae. Revista del Museo de La Plata (Nueva Serie), Sección Zoología 2: 341–379. [6 December, OS]

[B49] Blanchard EE (1943a) Un nuevo exoristido, importante parasito del gorgojo de las hortalizas (*Listroderesobliquus*, Klug). Revista de la Sociedad Entomológica Argentina 11: 450–454. [1 February, OS (vol. 11, p. 385)]

[B50] Blanchard EE (1943b) Un nuevo dexiido, *Ceraciasubandina*, parasito de la tucura, *Dichroplusarrogans*, Stal. Revista de la Sociedad Entomológica Argentina 12: 19–21. [30 September, OS (vol. 12, p. 3)]

[B51] Blanchard EE (1943c) Los dípteros muscoídeos del Museo de La Plata. Primera parte: Tachinidae. [Concl.] Revista del Museo de La Plata (Nueva Serie), Sección Zoología 3: 123–161. [30 December, OS]

[B52] Blanchard EE (1954) Contribucion al conocimiento de los oestromuscarios entomofagos argentinos. I. Los belvosiinos (Dipt. Exoristidae). Instituto de Sanidad Vegetal, Ministerio de Agricultura y Ganadería (Serie A) 10(57): 1–53.

[B53] Blanchard EE (1959) Dípteros parásitos de orugas de Arctiidae (“gatas peludas”). Revista de Investigaciones Agrícolas 13: 157–182.

[B54] Blanchard EE (1963) Dípteros parásitos de Noctuidae argentinos. Revista de Investigaciones Agrícolas 17: 129–254.

[B55] Blanchard EE (1966a) Nuevos triquiopodinos argentinos, parásitos de hemípteros nocivos. (Dipt. Gymnosomatidae). Revista de Investigaciones Agropecuarias. Serie 5. Patología Vegetal 3: 59–95 + 2 pls.

[B56] Blanchard EE (1966b) Dípteros parásitos de escarabaeoideos argentinos. Revista de Investigaciones Agropecuarias. Serie 5. Patología Vegetal 3: 175–229. [after 27 June, OS (date journal issue sent for publication)]

[B57] Blanchard EE De Santis L (1975) Primera lista anotada de Oestromuscarios entomófagos argentinos. Revista de Investigaciones Agropecuarias. Serie 5. Patología Vegetal 7: 7–76.

[B58] Boldt PE Cordo HA Gandolfo D (1991) Life history of Stolas (Anacassis) fuscata (Klug) (Coleoptera: Chrysomelidae) on seepwillow, *Baccharissalicifolia* (R. & P.) Pers. (Asteraceae). Proceedings of the Entomological Society of Washington 93: 839–844. [6 December, OS (wrapper of vol. 93, no. 4)]

[B59] Brauer F (1893) Vorarbeiten zu einer Monographie der Muscaria Schizometopa (exclusive Anthomyidae) von Prof. Dr. Fr. Brauer und Julius Edl. v. Bergenstamm. Verhandlungen der Kaiserlich-Königlichen Zoologisch-Botanischen Gesellschaft in Wien 43 (Abhandlungen): 447–525. [“Ende December”, OS (wrapper of vol. 43, p. ii)]

[B60] Brauer F (1898) Beiträge zur Kenntniss der Muscaria Schizometopa. I. Bemerkungen zu den Originalexemplaren der von Bigot, Macquart und Robineau-Desvoidy beschriebenen Muscaria Schizometopa aus Sammlung des Herrn G.H. Verrall. Zweite Folge. Sitzungsberichte der Mathematisch-Naturwissenschaftlichen Classe der Kaiserlichen Akademie der Wissenschaften in Wien. Abteilung I 107: 493–546. [31 December+, Evenhuis et al. 2016: 166]

[B61] Brauer F (1899) Beiträge zur Kenntniss der Muscaria Schizometopa. Bemerkungen zu den Originalexemplaren der von Bigot, Macquart und Robineau-Desvoidy beschriebenen Muscaria Schizometopa aus der Sammlung des Herrn G.H. Verrall. Dritte Folge. Sitzungsberichte der Mathematisch-Naturwissenschaftliche Classe der Kaiserlichen Akademie der Wissenschaften in Wien. Abteilung I 108: 495–529. [31 December+ (not June as given in Evenhuis et al. 2016: 166)]

[B62] Brauer F Bergenstamm JE von (1889) Die Zweiflügler des Kaiserlichen Museums zu Wien. IV. Vorarbeiten zu einer Monographie der Muscaria Schizometopa (exclusive Anthomyidae). Pars I. Denkschriften der Kaiserlichen Akademie der Wissenschaften. Wien. Mathematisch-Naturwissenschaftliche Classe 56: 69–180 + 11 pls. [Also published separately in Wien by F. Tempsky, 1890, 112 pp. + 11 pls.] [after 19 December (journal), February 1890 (separate), Evenhuis 2014: 497. Evenhuis (1997: 121) gave the same order for journal and separate and same year of publication and was followed by O’Hara and Wood 2004, O’Hara et al. 2009, and other works.]

[B63] Brauer F Bergenstamm JE von (1891) Die Zweiflügler des Kaiserlichen Museums zu Wien. V. Vorarbeiten zu einer Monographie der Muscaria Schizometopa (exclusive Anthomyidae). Pars II. Denkschriften der Kaiserlichen Akademie der Wissenschaften. Wien. Mathematisch-Naturwissenschaftliche Classe 58: 305–446. [Also published separately in Wien by F. Tempsky, 1891, 142 pp.] [17 December (journal), December (separate), Evenhuis 2014: 497. Evenhuis (1997: 121) dated the separate from 1891 and journal from 1892 and this order and dates were followed by O’Hara and Wood 2004, O’Hara et al. 2009, and other works.]

[B64] Brauer F Bergenstamm JE von (1893) Die Zweiflügler des Kaiserlichen Museums zu Wien. VI. Vorarbeiten zu einer Monographie der Muscaria Schizometopa (exclusive Anthomyidae). Pars III. F. Tempsky, Wien, 152 pp. [Also published in 1893, Denkschriften der Kaiserlichen Akademie der Wissenschaften. Wien. Mathematisch-Naturwissenschaftliche Classe 60: 89–240.] [August (separate), 14 December (journal), Evenhuis 2014: 497. Evenhuis (1997: 121) gave the same order but dated the journal from 1894 and this order and these dates were followed by O’Hara and Wood 2004, O’Hara et al. 2009, and other works.]

[B65] Brauer F Bergenstamm JE von (1894) Die Zweiflügler des Kaiserlichen Museums zu Wien. VII. Vorarbeiten zu einer Monographie der Muscaria Schizometopa (exclusive Anthomyidae). Pars IV. Denkschriften der Kaiserlichen Akademie der Wissenschaften. Wien. Mathematisch-Naturwissenschaftliche Classe 61: 537–624. [Also published separately in Wien by F. Tempsky, 1895, 88 pp.] [13 December (journal), February 1895 (separate), Evenhuis 2014: 497. Evenhuis (1997: 121) gave the separate as published first and dated both separate and journal from 1895 and this order and date were followed by O’Hara and Wood 2004, O’Hara et al. 2009, and other works.]

[B66] Brèthes J (1910) Dos insectos nuevos chilenos. Revista Chilena de Historia Natural 14: 67–69. [after 3 February, OS (date of manuscript)]

[B67] Brèthes J (1917) Sur quelques diptères de Lima (Perou). Anales de Zoología Aplicada (Agrícola, Médica, Veterinaria) 4: 16–18.

[B68] Brèthes J (1918) Quelques diptères du Chili. Revista Chilena de Historia Natural 22: 49–50. [30 June, OS]

[B69] Brèthes J (1920a) Cueillette d’insectes au Rio Blanco. III. Diptères. Revista Chilena de Historia Natural 23 [1919]: 40–44.

[B70] Brèthes J (1920b) Description d’un nouveau diptère chilien, parasite de *Laoravariabilis*. Anales de Zoología Aplicada (Agrícola, Médica, Veternaria) 7: 12–13.

[B71] Brèthes J (1925) Coléopteres et diptères chiliens. Revista Chilena de Historia Natural 29: 198–208.

[B72] Brooks AR (1942) *Clistomorpha*, *Psalidopteryx* and allies (Diptera, Tachinidae). Canadian Entomologist 74: 140–150. [28 September, OS (vol. 74, p. 156)] 10.4039/Ent74140-8

[B73] Brooks AR (1945) A revision of the North American species of the *Phasia* complex (Diptera, Tachinidae). Scientific Agriculture 25: 647–679. [after 8 March, OS (date manuscript received for publication)]

[B74] Burke HR (1977) Henry Jonathan Reinhard 1892–1976. Journal of Economic Entomology 70: 272. 10.1093/jee/70.2.272 323323

[B75] Byers GW Blank F Hanson WJ Beneway DF Fredrichson RW (1962) Catalogue of the types in the Snow Entomological Museum. Part III (Diptera). University of Kansas Science Bulletin 43: 131–181. [20 August, OS] 10.5962/bhl.part.13345

[B76] Caltagirone L (1954) Observaciones sobre *Incamyiachilensis* Aldrich y su multiplicacion en laboratorio. Revista Chilena de Entomología 3 [1953]: 87–99.

[B77] Caltagirone L (1966) Una nueva especie chilena de *Opsophagus* Aldrich, 1926 (Diptera, Tachinidae). Publicaciones del Centro de Estudios Entomológicos, Facultad de Filosofía y Educación, Universidad de Chile 8: 61–67.

[B78] Camousseight A (2005) La contribución entomológica de R.A. Philippi entre 1859 y 1875 y el estado actual de sus especies. Boletín del Museo Nacional de Historia Natural, Chile 54: 81–106.

[B79] Campos L (1953) Notas sobre taquinidos chilenos I (Dipt., Tachinidae). Agricultura Técnica (Chile) 13: 24–31.

[B80] Campos LE (1975) Insects – men and environment in Chile. Revista Chilena de Entomología 8 [1974]: 6–10. [February, OS (wrapper at end of vol. 8)]

[B81] Cerretti P (2010) I tachinidi della fauna italiana (DipteraTachinidae) con chiave interattiva dei generi ovest-paleartici. Volumes I & II. Centro Nazionale Biodiversità Forestale, Verona, 573 pp. (Vol. I) + 339 pp. (Vol. II) + CD ROM.

[B82] Cerretti P O’Hara JE Wood DM Shima H Inclán DJ Stireman JO III (2014) Signal through the noise? Phylogeny of the Tachinidae (Diptera) as inferred from morphological evidence. Systematic Entomology 39: 335–353. [12 March (online), journal website] 10.1111/syen.12062

[B83] Cerretti P Stireman JO III Pape T O’Hara JE Marinho MAT Rognes K Grimaldi DA (2017) First fossil of an oestroid fly (Diptera: Calyptratae: Oestroidea) and the dating of oestroid divergences. PLoS ONE 12(8): e0182101. [23 August, OS] 10.1371/journal.pone.0182101 PMC5568141 28832610

[B84] Cerretti P Wood DM O’Hara JE (2012) *Neoethilla*, a new genus for the first record of the Ethillini from the New World (Diptera, Tachinidae, Exoristinae). ZooKeys 242: 25–41. [15 November, OS] 10.3897/zookeys.242.3974 PMC3561682 23378795

[B85] Coelho SMP Carvalho CJB de Guimarães JH (1989) Chave e sinonímias para as espécies sul-americanas de *Winthemia* Robineau-Desvoidy (Diptera, Tachinidae) com descrição de três espécies novas. Revista Brasileira de Zoologia 6: 271–296. [15 August, OS] 10.1590/S0101-81751989000200014

[B86] Cooper BE O’Hara JE (1996) Diptera types in the Canadian National Collection of Insects. Part 4. Tachinidae. Agriculture and Agri-Food Canada, Publication A53-1918/B. Ottawa, 94 pp.

[B87] Coquillett DW (1895a) Descriptions of new genera and species, pp. 307–319. In: Johnson CW (Ed.) , Diptera of Florida.Proceedings of the Academy of Natural Sciences of Philadelphia 1895: 303–340. [20 August, Evenhuis 2018a: 88]

[B88] Coquillett DW (1895b) Notes and descriptions of Tachinidae. Journal of the New York Entomological Society 3: 49–58. [September, Evenhuis 2018a: 88]

[B89] Coquillett DW (1897) Revision of the Tachinidae of America north of Mexico. A family of parasitic two-winged insects. United States Department of Agriculture. Division of Entomology. Technical Series 7: 1–156. [Some copies have 154 pp., differing from the longer version in containing a less complete index and no Errata.] [after 2 June, OS (date of Letter of Transmittal on p. 3)] 10.5962/bhl.title.87236

[B90] Coquillett DW (1910) The type-species of the North American genera of Diptera. Proceedings of the United States National Museum 37 (No. 1719): 499–647. [4 August, OS (Table of Contents of vol. 37)] 10.5479/si.00963801.37-1719.499

[B91] Cortés R (1944a) El Dr. Charles H.T. Townsend. Revista Universitaria (Universidad Católica de Chile) 29: 109–111. [after 21 April, OS (date of manuscript)]

[B92] Cortés R (1944b) Sinopsis historica de los estudios sobre taquinidos chilenos (Dipt., Tachinidae). Boletín de Sanidad Vegetal (Chile) 3 [1943]: 5–11. [14 June, date stamp on reprint]

[B93] Cortés R (1944c) Las especies chilenas del genero *Archytas* Jaen. (Dipt., Tachinidae). Boletín de Sanidad Vegetal (Chile) 3 [1943]: 139–141.

[B94] Cortés R (1944d) Taquinidos nuevos para Chile (Dipt., Tachinidae). Boletín de Sanidad Vegetal (Chile) 3 [1943]: 142.

[B95] Cortés R (1944e) Las especies chilenas del genero *Cylindromyia* Meigen (Dipt., Tachinidae). Boletín de Sanidad Vegetal (Chile) 3 [1943]: 177–179.

[B96] Cortés R (1944f) *Trichodischia* Bigot y *Dischotrichia* n. gen., generos nuevos de taquinidos para Chile (Dipt. Tachinidae). Revista Universitaria (Universidad Católica de Chile) 29: 49–58.

[B97] Cortés R (1944g) Las especies chilenas del genero *Sturmia* R. D. (Dipt., Tachinidae). Boletín del Museo Nacional de Historia Natural, Chile 22: 159–167. [after November, OS (date of manuscript)]

[B98] Cortés R (1945a) Algunos generos goniinos de taquinidos chilenos (Dipt., Tachinidae). Agricultura Técnica (Chile) 4 [1944]: 115–124. 10.1590/S0074-02761989000800025

[B99] Cortés R (1945b) Nuevo nombre generico para un taquinido de la Republica Argentina (Dipt., Tachinidae). Acta Zoológica Lilloana 2 [1944]: 255–257.

[B100] Cortés R (1945c) Taquinidos chilenos nuevos o poco conocidos (Dipt., Tachinidae). Agricultura Técnica (Chile) 5: 24–30.

[B101] Cortés R (1945d) Especies chilenas de los generos *Phorocera* R. D. y *Parasetigena* B. B. (Dipt., Tachinidae). Acta Zoológica Lilloana 3: 157–164.

[B102] Cortés R (1945e) Nuevos generos de taquinidos chilenos aliados a *Trichoprosopus* Macquart (Dipt., Tachinidae). Revista Chilena de Historia Natural 48 [1944]: 149–160. [after July, OS (date of manuscript)]

[B103] Cortés R (1946) Tachinidae, pp. 172–185. In: Stuardo [Ortíz] C, Catálogo de los dípteros de Chile. Santiago. 250 + [1 (errata)] pp.

[B104] Cortés R (1948) Sobre algunos taquinidos chilenos y sus huespedes (Dipt., Tachinidae). Revista Universitaria (Universidad Católica de Chile) 33: 119–125.

[B105] Cortés R (1950) Nueva especie chilena del genero *Phorocera* R. D. (Dipt., Tachinidae). Agricultura Técnica (Chile) 9 [1949]: 5–10.

[B106] Cortés R (1951a) Sobre tres especies de taquinidos chilenos (Dipt., Tachinidae). Agricultura Técnica (Chile) 10 [1950]: 59–65.

[B107] Cortés R (1951b) Nuevos generos de Tachininae chilenos con cerdas facio-orbitales (DipteraTachinidae). Revista Chilena de Entomología 1: 249–262. [27 December, OS]

[B108] Cortés R (1952) Los insectos de las Islas Juan Fernández. 9. Tachinidae (Diptera). Revista Chilena de Entomología 2: 109–111. [19 December, OS]

[B109] Cortés R (1963) Tipos de Tachinidae (Diptera) chilenos en algunos museos extranjeros. Revista Universitaria (Universidad Católica de Chile) 47 [1962]: 241–252.

[B110] Cortés R (1967a) Primeros generos de taquinidos chilenos con la cuarta vena longitudinal evanescente (Diptera: Tachinidae). Boletín Técnico, Estación Experimental Agronómica, Universidad de Chile 26: 3–9. [August, OS (wrapper of bulletin)]

[B111] Cortés R (1967b) Taquinidos chilenos nuevos o poco conocidos – II (Diptera: Tachinidae). Boletín Técnico, Estación Experimental Agronómica, Universidad de Chile 26: 10–29. [August, OS (wrapper of bulletin)]

[B112] Cortés R (1968a) Taquinidos chilenos (Dipt., Tachinidae) parasitos de phasmidos (Phasmoidea). Boletín de la Sociedad de Biología de Concepción 40[1965–1966]: 101–111. [20 June, OS (wrapper at end of bulletin)]

[B113] Cortés R (1968b) Nueva especie chilena del genero *Incamyia* Townsend 1912 (Diptera: Tachinidae). Revista Chilena de Entomología 6: 17–20. [23 December, OS (wrapper at end of vol. 6)]

[B114] Cortés R (1968c) El taquinido mas austral de Chile (Diptera: Tachinidae). Revista Chilena de Entomología 6: 142. [23 December, OS (wrapper at end of vol. 6)]

[B115] Cortés R (1969) Rehabilitacion de una especie bigotiana. *Trichodischiacaerulea* Bigot 1885, diferente de *T.soror* Bigot 1885 (Diptera: Tachinidae). Revista Universitaria (Universidad Católica de Chile) 53: 97–99. [after 30 May, OS (date read at meeting of the Academia Chilena de Ciencias Naturales)]

[B116] Cortés R (1973a) Taquinidos chilenos nuevos o poco conocidos III (Diptera, Tachinidae). Revista Chilena de Entomología 7: 97–105. [15 July, OS (wrapper at end of vol. 7)]

[B117] Cortés R (1973b) Dr. Everard E. Blanchard. Revista Chilena de Entomología 7: 261–262. [15 July, OS (wrapper at end of vol. 7)]

[B118] Cortés R (1975) Sobre cuatro generos de dexiinos chilenos (Diptera, Tachinidae) con los femures posteriores de los machos modificados. Revista Chilena de Entomología 8 [1974]: 35–38. [February, OS (wrapper at end of vol. 8)]

[B119] Cortés R (1976) Taquinidos chilenos nuevos o poco conocidos, IV – (Diptera, Tachinidae). Boletín Técnico, Facultad de Agronomía, Universidad de Chile 40: 3–14.

[B120] Cortés R (1979) Taquinidos chileno – argentinos (Diptera, Tachinidae). Investigación Agricola (Chile) 5: 75–82.

[B121] Cortés R (1980) Neotropical Tachinidae (Diptera) I. Notes, records, distribution and descriptions. Revista Brasileira de Entomologia 24: 105–110. [29 August, OS]

[B122] Cortés R (1982) *Marnefiamirifica* n. gen. et sp. – con insólita venación alar (Diptera, Tachinidae, Actiini). Phegea 10: 137–144.

[B123] Cortés R (1983) First record of transantarctic relationships in the Tachinidae (Diptera, Muscoidea, Calyptratae). Revista Brasileira de Zoologia 1: 419–420. [7 September, OS] 10.1590/S0101-81751982000400003

[B124] Cortés R (1984) Tachinid flies (Diptera: Tachinidae) from Tarapacá and Antofagasta provinces, Chile. III. Addendum. Florida Entomologist 66 [1983]: 377–389. [24 February, OS (wrapper of vol. 66, no. 4)] 10.2307/3494010

[B125] Cortés R (1986) Taquinidos de Aysen (XI Region) y Magallanes (XII Region) Chile (Diptera: Tachinidae). Acta Entomológica Chilena 13: 133–160. [June, OS (wrapper of vol. 13)]

[B126] Cortés R (1989) Algunos caracteres no genericos en taquinidos chilenos (Diptera: Tachinidae). Acta Entomológica Chilena 15: 271–274. [June, OS (wrapper of vol. 15)]

[B127] Cortés R (1992) Nuevas sinonimias de taquinidos chilenos (Diptera: Tachinidae). Acta Entomológica Chilena 17: 235–236. [before 8 December, CAL date stamp]

[B128] Cortés R Campos L (1971) Taquinidos de Tarapacá y Antofagasta (Diptera: Tachinidae). Anales de la Universidad del Norte 8 [1970]: 1–104. [31 January, date stamp on reprint]

[B129] Cortés R Campos L (1974) Taquínidos de Tarapacá y Antofagasta. Addenda I. (Diptera, Tachinidae). Idesia 3: 111–125. [December, Evenhuis et al. 2015: 311]

[B130] Cortés R González CR (1989) Generos voriinos de taquinidos chilenos (Diptera: Tachinidae, Voriini). Memórias do Instituto Oswaldo Cruz 84 (Suppl. IV): 115–123. 10.1590/S0074-02761989000800025

[B131] Cortés R Herrera J (1989) Antecedentes historicos y bibliograficos para una historia de la entomologia en Chile. Acta Entomológica Chilena 15: 297–321. [June, OS (wrapper of vol. 15)]

[B132] Cortés R Hichins N (1969) Distribución geográfica y huéspedes conocidos de los taquinidos de Chile (Diptera: Tachinidae). Ediciones de la Universidad de Chile, Santiago, 92 pp. + [98] pls.

[B133] Cortés R Hichins N (1979) Taquínidos de Tarapacá y Antofagasta (Diptera: Tachinidae), addenda II. Idesia 5: 111–116.

[B134] Cortés R Valencia L (1972) Nueva especie chileno-peruana del género *Ateloglutus* Aldrich, 1934, con descripción de un nuevo subgénero (Diptera, Tachinidae, Voriini). Idesia 2: 65–70.

[B135] Crosskey RW (1973) A conspectus of the Tachinidae (Diptera) of Australia, including keys to the supraspecific taxa and taxonomic and host catalogues. Bulletin of the British Museum (Natural History). Entomology Supplement 21: 221 pp. [7 December, OS] 10.5962/p.193226

[B136] Crosskey RW (Ed) (1980) Catalogue of the Diptera of the Afrotropical Region. British Museum (Natural History), London, 1437 pp. [10 July, Evenhuis 2008b: 401]

[B137] Curran CH (1925) The American species of the tachinid genus *Peleteria* Desv. (Diptera). Proceedings and Transactions of the Royal Society of Canada (Ser. 3) 19 (5): 225–257 + [1] p. + 2 pls. [after May, OS (date read at meeting of the Royal Society of Canada)]

[B138] Curran CH (1926) Appendix. New Diptera from Jamaica, pp. 102–114. In: Gowdey CC Catalogus insectorum jamaicensis. Department of Agriculture, Jamaica.Entomological Bulletin 4: 1–114.

[B139] Curran CH (1927) Some new North American Diptera. Canadian Entomologist 59: 290–303. [31 December, OS (vol. 59, p. 304)] 10.4039/Ent59290-12

[B140] Curran CH (1928a) Insects of Porto Rico and the Virgin Islands. Diptera or two-winged flies. Scientific Survey of Porto Rico and the Virgin Islands. Volume XI—Part 1. New York Academy of Sciences, New York, 118 pp. [before 17 February, Evenhuis 1997: 162]

[B141] Curran CH (1928b) Revision of the American species of *Archytas* (Tachinidae, Diptera). [Cont.] Canadian Entomologist 60: 201–208. [4 September, OS] 10.4039/Ent60201-8

[B142] Curran CH (1928c) Revision of the American species of *Archytas* (Tachinidae, Diptera). [Cont.] Canadian Entomologist 60: 218–226. [6 October, OS (vol. 60, p. 232)] 10.4039/Ent60218-9

[B143] Curran CH (1928d) Revision of the American species of *Archytas* (Tachinidae, Diptera). [Cont.] Canadian Entomologist 60: 249–256. [29 October, OS] 10.4039/Ent60249-10

[B144] Curran CH (1928e) Revision of the American species of *Archytas* (Tachinidae, Diptera). [Concl.] Canadian Entomologist 60: 275–282. [1 December, OS] 10.4039/Ent60275-11

[B145] Curran CH (1934) The families and genera of North American Diptera. Ballou Press, New York, 512 pp. + 2 pls. [25 August, Thompson et al. 1999: 378] 10.5962/bhl.title.6825

[B146] Curran CH (1947) New and little known American Tachinidae. Bulletin of the American Museum of Natural History 89: 41–122. [30 September, OS]

[B147] Curtis J (1836) [Foreword], p. 315. In: Curtis J, Haliday AH, Walker F, Descriptions, &c. of the insects collected by Captain P.P. King, R.N., F.R.S., in the survey of the Straits of Magellan. Transactions of the Linnean Society of London 17 [1837]: 315–359. 10.1111/j.1095-8339.1834.tb00026.x

[B148] Curtis J (1837) British Entomology; being illustrations and descriptions of the genera of insects found in Great Britain and Ireland: containing coloured figures from nature of the most rare and beautiful species, and in many instances of the plants upon which they are found. Vol. 14. Privately published, London. [2 (index, errata and addenda)] pp. + pls. 626–629. [Each plate is accompanied by two unnumbered pages of text.] [1 January, Evenhuis 1997: 166]

[B149] Dallas ED (1928) Dr. Juan Brèthes. Bio-bibliografía. Revista de la Sociedad Entomológica Argentina 2: 103–112 + 1 pl. [30 December, OS (wrapper of vol. 2, no. 2)]

[B150] Dios R de VP Nihei SS (2016) A remarkable new species of *Eutrichopoda* Townsend, 1908 (Diptera: Tachinidae: Phasiinae). Zootaxa 4121: 194–200. [8 June, OS] 10.11646/zootaxa.4121.2.10 27395220

[B151] Dios R de VP Nihei SS (2017) Taxonomic revision of the Neotropical genus *Ectophasiopsis* Townsend, 1915 (Diptera: Tachinidae: Phasiinae). European Journal of Taxonomy 334: 1–27. [4 July, OS] 10.5852/ejt.2017.334

[B152] Dios R de VP Nihei SS (2020) Taxonomic revision of the genus *Trichopoda* Berthold, 1827 (Diptera: Tachinidae: Phasiinae), with emphasis on the Neotropical fauna. Zootaxa 4870: 1–104. [5 November, OS] 10.11646/zootaxa.4870.1.1 33311339

[B153] d’Orbigny CVD. See Orbigny CVD d’

[B154] Dugdale JS (1969) A classification of the New Zealand genera of Tachinidae (Diptera: Cyclorrhapha). New Zealand Journal of Science 12: 606–646.

[B155] Edmundson W (2009) A history of the British presence in Chile. From Bloody Mary to Charles Darwin and the decline of British influence. Palgrave Macmillan, New York, 276 pp. 10.1057/9780230101210

[B156] Edwards FW (1929) Introduction, pp. vii–xiv. In: Alexander CP, Diptera of Patagonia and South Chile based mainly on material in the British Museum (Natural History). Part I.—Crane-flies. British Museum (Natural History), London. xvi + 240 pp. + XII pls. [January, Evenhuis 1997: 126]

[B157] Emden FI van (1949) The scientific name of the common tachinid parasite of *Diatraea* spp. (Lep. Pyral.) in Central and South America, with notes on related species. (Dipt.) Revista de Entomologia 20: 499–508. [31 August, OS (vol. 20, p. iv)]

[B158] Etcheverry M (1957) *Laphygmafrugiperda* (Abbot & Smith) en Chile (LepidopteraNoctuidae). Revista Chilena de Entomología 5: 183–192. [10 October, OS]

[B159] Etcheverry M (1993) Los naturalistas de la familia Reed en Chile: Edwyn Charles (1841–1910), Edwyn Pastor (1880–1966) y Carlos Samuel (1888–1949). Boletín de la Sociedad de Biología de Concepción 64: 85–95.

[B160] Evenhuis NL (Ed) (1989) Catalog of the Diptera of the Australasian and Oceanian regions. Bishop Museum Special Publication 86. Bishop Museum Press, Honolulu and E.J. Brill, Leiden, 1155 pp. [23 August, Evenhuis 2008b: 402]

[B161] Evenhuis NL (1990) Dating of livraisons and volumes of d’Orbigny’s *Dictionnaire Universel d’Histoire Naturelle*. Bishop Museum Occasional Papers 30: 219–225. [6 June, Evenhuis 2008a: 63]

[B162] Evenhuis NL (1995) Dating of the “Proceedings of the Hawaiian Entomological Society” (1906–1993). Proceedings of the Hawaiian Entomological Society 32: 39–44. [10 August, Thompson et al. 1999: 414]

[B163] Evenhuis NL (1997) Litteratura taxonomica dipterorum (1758–1930). Being a selected list of the books and prints of Diptera taxonomy from the beginning of Linnaean zoological nomenclature to the end of the year 1930; containing information on the biographies, bibliographies, types, collections, and patronymic genera of the authors listed in this work; including detailed information on publication dates, original and subsequent editions, and other ancillary data concerning the publications listed herein. Backhuys Publishers, Leiden, 871 pp. [Published in two volumes simultaneously: Vol. I, A–K (pp. 1–426); Vol. II, L–Z (pp. 427–871).] [10 October, Evenhuis et al. 2015: 312]

[B164] Evenhuis NL (2003a) The complete bibliography of scientific works of Jacques-Marie-Frangille Bigot. Zootaxa 210: 1–36. [6 June, OS] 10.11646/zootaxa.210.1.1

[B165] Evenhuis NL (2003b) Publication and dating of the journals forming the Annals and Magazine of Natural History and the Journal of Natural History. Zootaxa 385: 1–68. [16 December, OS] 10.11646/zootaxa.385.1.1

[B166] Evenhuis NL (2008a) Celebrating a centuria of volumes of the *Bishop Museum Occasional Papers*: history, contents, dates of publication, and author index. Bishop Museum Occasional Papers 101: 1–76. [3 October, O’Hara et al. 2011: 222]

[B167] Evenhuis NL (2008b) Dates of publication of regional and world Diptera catalogs. Studia Dipterologica 14 [2007]: 379–403. [29 October, OS]

[B168] Evenhuis NL (2014) Publication and dating of parts IV–VII of Brauer & Bergenstamm’s *Die Zweiflügler des Kaiserlichen Museums zu Wien* (1889–1894). Zootaxa 3790: 495–499. [22 April, OS] 10.11646/zootaxa.3790.3.8 24869882

[B169] Evenhuis NL (2015) Dates of the Diptera in Volume 7 of the *Zoología* of Gay’s “*Historia Física y Política de Chile*”. Fly Times 55: 40–45. [22 December (OS, title page of issue 55)]

[B170] Evenhuis NL (2018a) Nomenclatural studies toward a world list of Diptera genus-group names. Part VI: Daniel William Coquillett. Zootaxa 4381: 1–95. [19 February, OS] 10.11646/zootaxa.4381.1.1 29689947

[B171] Evenhuis NL (2018b) The life and work of Francis Walker (1809–1874). Fly Times, Supplement 2: 101 pp. [27 September, OS]

[B172] Evenhuis NL O’Hara JE Pape T Pont AC (2010) Nomenclatural studies toward a world catalog of Diptera genus-group names. Part I. André-Jean-Baptiste Robineau-Desvoidy. Zootaxa 2373: 1–265. [26 February, OS]

[B173] Evenhuis NL Pape T (2017) Battling the un-dead: the status of the Diptera genus-group names originally proposed in Johann Wilhelm Meigen’s 1800 pamphlet. Zootaxa 4275: 1–74. [8 June, OS] 10.11646/zootaxa.4275.1.1 28610225

[B174] Evenhuis NL Pape T (2019) Nomenclatural studies toward a world list of Diptera genus-group names. Part VII: Johann Wilhelm Meigen. Zootaxa 4703: 1–193. [5 December, OS] 10.11646/zootaxa.4703.1.1 32229894

[B175] Evenhuis NL Pape T Pont AC (2008) The problems of subsequent typification in genus-group names and use of the *Zoological Record*: a study of selected post-1930 Diptera genus-group names without type species designations. Zootaxa 1912: 1–44. [22 October, OS] 10.11646/zootaxa.1912.1.1

[B176] Evenhuis NL Pape T Pont AC (2016) Nomenclatural studies toward a world list of Diptera genus-group names. Part V: Pierre-Justin-Marie Macquart. Zootaxa 4172: 1–211. [30 September, OS] 10.11646/zootaxa.4172.1.1 27701208

[B177] Evenhuis NL Pont AC (2013) Nomenclatural studies toward a world catalog of Diptera genus-group names. III. Christian Rudolph Wilhelm Wiedemann. Zootaxa 3638: 1–75. [16 April, OS] 10.11646/zootaxa.3638.1.1 25325087

[B178] Evenhuis NL Pont AC Whitmore D (2015) Nomenclatural studies toward a world list of Diptera genus-group names. Part IV: Charles Henry Tyler Townsend. Zootaxa 3978: 1–362. [25 June, OS] 10.11646/zootaxa.3978.1.1 26249934

[B179] Evenhuis NL Thompson FC (1990) Type designations of genus-group names of Diptera given in d’Orbigny’s *Dictionnaire Universel d’Histoire Naturelle*. Bishop Museum Occasional Papers 30: 226–258. [6 June, Evenhuis 2008a: 63]

[B180] Evenhuis NL Thompson FC Pont AC Pyle BL (1989) Literature cited, pp. 809–991. In: Evenhuis NL (Ed), Catalog of the Diptera of the Australasian and Oceanian regions. Bishop Museum Special Publication 86. Bishop Museum Press, Honolulu and E.J. Brill, Leiden, 1155 pp. 10.5962/bhl.title.49897

[B181] Fabricius JC (1781) Species insectorum exhibentes eorum differentias specificas, synonyma, auctorum, loca natalia, metamorphosin. Vol. 2. C.E. Bohnii, Hamburg and Kiel, 494 pp. [An appendix and index were published later, pp. 495–517.] [March, Evenhuis 1997: 247] 10.5962/bhl.title.36509

[B182] Fabricius JC (1805) Systema antliatorum secundum ordines, genera, species adiectis synonymis, locis, observationibus, descriptionibus. C. Reichard, Brunsvigae [= Brunswick]. xiv + 15–372 + [1 (errata)] + 30 pp. [31 December+, Evenhuis et al. 2016: 250] 10.5962/bhl.title.15806

[B183] Fallén CF (1810) Försök att bestämma de i Sverige funne flugarter, som kunna föras till slägtet *Tachina*. Kongliga Vetenskaps Academiens Nya Handlingar (Ser. 2) 31: 253–287.

[B184] Fallén CF (1815) Beskrifning öfver några Rot-fluge Arter, hörande till slägterna *Thereva* och *Ocyptera*. Kongliga Vetenskaps Academiens Nya Handlingar (Ser. 3) 1815: 229–240.

[B185] Fleming AJ Wood DM Smith MA Dapkey T Hallwachs W Janzen DH (2017) A new species of *Voria* Robineau-Desvoidy (Diptera: Tachinidae) from Area de Conservación Guanacaste in northwestern Costa Rica. Biodiversity Data Journal 5 (e20123): 1–19. [1 December, OS] 10.3897/BDJ.5.e20123 PMC5784236 29391853

[B186] Giglio-Tos E (1893) Diagnosi di nuovi generi e di nuove specie di Ditteri. IX. Bollettino dei Musei di Zoologia ed Anatomia Comparata della Reale Università di Torino 8 (No. 158): 1–14. [1 July, OS] 10.5962/bhl.part.27226

[B187] Giglio-Tos E (1894) Ditteri del Messico. Parte Terza. Muscidae Calypteratae. Ocypterinae, Gymnosominae, Phasinae, Phaninae, Tachininae, Dexinae, Sarcophaginae. C. Clausen, Torino, 76 pp. + 1 pl. [Also published later in 1894 with same title in Memorie della Reale Accademia della Scienze di Torino (Ser. 2) 44: 473–546 + 1 pl. Only this later version was seen by the authors and this version is cited in the text.] [2 April, Evenhuis 1997: 300]

[B188] González CR (1989) El genero *Ateloglutus* Aldrich, 1934 con una nueva especie para la Republica Argentina (Diptera: Tachinidae: Voriini). Acta Entomológica Chilena 15: 225–228. [June, OS (wrapper of vol. 15)]

[B189] González CR (1992a) Generos cuphocerinos de taquinidos chilenos (Diptera: Tachinidae: Cuphocerini). Acta Entomológica Chilena 17: 53–68. [before 8 December, CAL date stamp]

[B190] González CR (1992b) Taquinidos de la Reserva Nacional de Río Clarillo (Diptera: Tachinidae). Acta Entomológica Chilena 17: 175–185. [before 8 December, CAL date stamp]

[B191] González CR (2001) En Memoria. Profesor Raúl E. Cortés Peña, 1915–2001. Acta Entomológica Chilena 25: 85–87.

[B192] González CR Henry A (1992) El genero Neotropical *Incamyia* Townsend, 1912 en Chile, con una clave para sus especies (Diptera: Tachinidae: Blondeliini). Anales del Museo de Historia Natural de Valparaíso 20 [1989]: 35–39.

[B193] González CR Vergés X (2004) Revisión de las especies de la tribu Goniini de distribución Chilena (Diptera: Tachinidae). Acta Entomológica Chilena 28: 39–62. [before 21 January 2005, CAL date stamp]

[B194] Gramajo MC (1998) Lista preliminar de las Tachinidae (Diptera) de la Patagonia argentina. Revista de la Sociedad Entomológica Argentina 57: 91–99. [January, OS (vol. 57, p. 140)]

[B195] Gramajo MC (2011) Una especie nueva de *Dasyuromyia* (Diptera: Tachinidae) de la Patagonia Argentina. Acta Zoológica Lilloana 55: 171–176.

[B196] Guimarães JH (1960) Contribuição ao conhecimento do gênero *Archytas* Jaennicke, 1867 (Diptera, Tachinidae). Memórias do Instituto Oswaldo Cruz 58: 115–124. [July, OS] 10.1590/S0074-02761960000100006 13709659

[B197] Guimarães JH (1961a) Segunda contribuição ao conhecimento do gênero *Archytas* Jaennecke, 1867 (Diptera, Tachinidae). Memórias do Instituto Oswaldo Cruz 59: 163–179. [July, OS] 10.1590/S0074-02761961000200004 13902852

[B198] Guimarães JH (1961b) Terceira contribuição ao conhecimento do gênero *Archytas* Jaennecke, 1867 (Diptera, Tachinidae). Memórias do Instituto Oswaldo Cruz 59: 355–396. [September, OS] 10.1590/S0074-02761961000200004 13902853

[B199] Guimarães JH (1962) Espécies brasileiras do gênero *Peleteria* Desvoidy, 1830 (Diptera, Tachinidae). Anais da Academia Brasileira de Ciências 34: 483–495. [31 December, OS]

[B200] Guimarães JH (1963a) Primeira contribuição ao conhecimento da tribu Cuphoceratini (Diptera, Tachinidae). Memórias do Instituto Oswaldo Cruz 61: 41–72. [June, OS] 10.1590/S0074-02761963000100003 14092245

[B201] Guimarães JH (1963b) Quarta contribuição ao conhecimento do gênero *Archytas* Jaennecke, 1867 (Diptera, Tachinidae). Memórias do Instituto Oswaldo Cruz 61: 153–164. [June, OS] 10.1590/S0074-02761963000100012

[B202] Guimarães JH (1963c) Fifth contribution to the knowledge of the genus *Archytas* Jaennecke, 1867 (Diptera, Tachinidae). Memórias do Instituto Oswaldo Cruz 61: 329–340. [August, OS] 10.1590/S0074-02761963000200004 14089868

[B203] Guimarães JH (1971) Family Tachinidae (Larvaevoridae). A catalogue of the Diptera of the Americas south of the United States 104: 1–333. [6 December, OS]

[B204] Guimarães JH (1972) A revision of the genus *Winthemia* Robineau-Desvoidy in America north of Mexico (Diptera, Tachinidae). Arquivos de Zoologia 22: 27–112. [29 February, OS] 10.11606/issn.2176-7793.v22i2p27-112

[B205] Guimarães JH (1976) A revision of the genus *Cylindromyia* Meigen in the Americas south of the United States (Diptera, Tachinidae). Arquivos de Zoologia 27: 1–50. [30 January, OS] 10.11606/issn.2176-7793.v27i1p1-50

[B206] Guimarães JH (1977a) A review of the tribe Oestrophasiini Brauer & Bergenstamm (Diptera, Tachinidae). Papéis Avulsos de Zoologia 30: 215–238. [20 January, OS]

[B207] Guimarães JH (1977b) A revision of the genus *Paratheresia* Townsend (Diptera: Tachinidae, Theresiini). Papéis Avulsos de Zoologia 30: 267–288. [20 January, OS]

[B208] Guimarães JH (1977c) Host-parasite and parasite-host catalogue of South American Tachinidae (Diptera). Arquivos de Zoologia 28: 1–131. [20 January, OS] 10.11606/issn.2176-7793.v28i3p1-131

[B209] Guimarães JH (1983) Taxonomy of Brazilian flies of the genus *Lespesia* Robineau-Desvoidy (Diptera, Tachinidae). Papéis Avulsos de Zoologia 35: 11–30. [20 August, OS]

[B210] Hansen DE Toma R (2004) Visit to Itaquaquecetuba (Brazil) and the old homestead of C.H.T. Townsend. The Tachinid Times 17: 3–8. [3 March, newsletter website]

[B211] Henry A (1987) Los taquinidos (Diptera: Tachinidae) de la coleccion del Instituto de Entomologia de la Universidad Metropolitana de Ciencias de la Educacion. Acta Entomológica Chilena 14: 189–207. [December, OS (wrapper of vol. 14)]

[B212] Herting B (1974) Revision der von Robineau-Desvoidy beschriebenen europäischen Tachiniden und Rhinophorinen (Diptera). Stuttgarter Beiträge zur Naturkunde. Serie A (Biologie) 264: 1–46. [1 June, OS]

[B213] Herting B (1984) Catalogue of Palearctic Tachinidae (Diptera). Stuttgarter Beiträge zur Naturkunde. Serie A (Biologie) 369: 1–228. [30 November, OS]

[B214] Herting B Dely-Draskovits Á (1993) Family Tachinidae, pp. 118–458. In: Soós Á, Papp L (Eds), Catalogue of Palaearctic Diptera. Vol. 13. Anthomyiidae—Tachinidae. Hungarian Natural History Museum, Budapest, 624 pp. [15 December, Evenhuis 2008b: 402]

[B215] Hichins N (1969) Los Taquinidos de la Quebrada de La Plata. Noticiario Mensual del Museo Nacional de Historia Natural 157: 3–8.

[B216] Holston KC Irwin ME Thompson FC (2003) Case 3251. *Thereva* Latreille, 1797 and *Phasia* Latreille, 1804 (Insecta, Diptera): proposed conservation of usage by designation of *Muscaplebeja* Linnaeus, 1758 as the type species of *Thereva*. Bulletin of Zoological Nomenclature 60: 198–202. [30 September, OS (wrapper of vol. 60, pt. 3)]

[B217] ICZN. See International Commission on Zoological Nomenclature 10.3897/zookeys.931.51583 PMC7205856 32405237

[B218] International Commission on Zoological Nomenclature (1954) Opinion 205. Rejection of the generic name *Phoranthella* Townsend (Class Insecta, Order Diptera), as published in 1915, as a *nomen nudum*, pp. 311–317. In: Hemming F (ed), Opinions and declarations rendered by the International Commission on Zoological Nomenclature. Vol. 3. London, 448 pp. [27 January, OS (vol. 3, p. 447)]

[B219] International Commission on Zoological Nomenclature (1963) Opinion 678. The suppression under the Plenary Powers of the pamphlet published by Meigen, 1800. Bulletin of Zoological Nomenclature 20: 339–342. [21 October, OS (wrapper of vol. 20, pt. 5)]

[B220] International Commission on Zoological Nomenclature (1964) Opinion 712. Forty-seven genera of decapod Crustacea: placed on the Official List. Bulletin of Zoological Nomenclature 21: 336–351. [26 November, OS (wrapper of vol. 21, pt. 5)]

[B221] International Commission on Zoological Nomenclature (1970) Opinion 896. *Phasia* Latreille, 1804, (Insecta, Diptera): addition to the Official List. Bulletin of Zoological Nomenclature 26 [1969]: 196–199. [7 April, OS (wrapper of vol. 26, pts. 5/6)]

[B222] International Commission on Zoological Nomenclature (1974) Opinion 1008. *Siphona* Meigen, 1803 and *Haematobia* Lepeletier and Serville, 1828 (Insecta: Diptera): designations under the Plenary Powers. Bulletin of Zoological Nomenclature 30: 157–158. [28 June, OS (wrapper of vol. 30, pts. 3/4)]

[B223] International Commission on Zoological Nomenclature (1983) Opinion 1255. *Lespesia* Robineau-Desvoidy, 1863 (Diptera, Tachinidae): designation of type species. Bulletin of Zoological Nomenclature 40: 97–101. [15 July, OS (wrapper of vol. 40, pt. 2)]

[B224] International Commission on Zoological Nomenclature (1987) Opinion 1432. *Actia* Robineau-Desvoidy, 1830 (Insecta, Diptera): *Roeselialamia* Meigen, 1838, designated as type species. Bulletin of Zoological Nomenclature 44: 71–72. [23 March, OS (wrapper of vol. 44, pt. 1)]

[B225] International Commission on Zoological Nomenclature (1999) International Code of Zoological Nomenclature. Fourth edition adopted by the International Union of Biological Sciences. International Trust for Zoological Nomenclature, London. xxix + 306 pp. [p. iii: “The provisions of this Code supersede those of the previous editions with effect from 1 January 2000”.]

[B226] International Commission on Zoological Nomenclature (2006) Opinion 2142 (Case 3251). *Thereva* Latreille, 1797 and *Phasia* Latreille, 1804 (Insecta, Diptera): usage conserved by the designation of *Muscaplebeja* Linnaeus, 1758 as the type species of *Thereva*. Bulletin of Zoological Nomenclature 63: 72–73. [31 March, OS (wrapper of vol. 63, pt. 1)]

[B227] International Commission on Zoological Nomenclature (2012) Opinion 2307 (Case 3539). *Sturmia* Robineau-Desvoidy, 1830, *Senometopia* Macquart, 1834 and *Drino* Robineau-Desvoidy, 1863 (Insecta, Diptera, Tachinidae): usage conserved. Bulletin of Zoological Nomenclature 69: 242–243. [1 September, BioOne Complete website] 10.21805/bzn.v69i3.a7

[B228] Jaennicke F (1867) Neue exotische Dipteren. Abhandlungen, herausgegeben von der Senckenbergischen Naturforschenden Gesellschaft 6: 311–408 + pls. 43–44. [Also published separately as his “Neue exotische Dipteren aus den Museen zu Frankfurt a. M. und Darmstadt”, 99 + [1] pp. + 2 pls., C. Winter, Frankfurt, 1868 (as 1867).] [November, Evenhuis 1997: 390]

[B229] Jones TH (1913) Some notes on *Laphygmafrugiperda* S. and A. in Porto Rico. Journal of Economic Entomology 6: 230–236. 10.1093/jee/6.2.230

[B230] Kabat AR Coan EV (2017) The life and work of Rudolph Amandus Philippi (1808–1904). Malacologia 60: 1–30. [1 June, journal website] 10.4002/040.060.0103

[B231] Koçak AÖ Kemal M (2010) Nomenclatural notes on the genus group names of some families (Diptera). Priamus 12: 156–160. [19 March, OS (vol. 12, p. 151)]

[B232] Lamas CJE Nihei SS Papavero N (2009) Necrológio. José Henrique Guimarães (08.10.1937–14.10.2008). Revista Brasileira de Entomologia 52 [2008]: 674–676. 10.1590/S0085-56262008000400022

[B233] Latreille PA (1804) Tableau méthodique des insectes, pp. 129–200. In: Société de Naturalistes et d’Agriculteurs, Nouveau dictionnaire d’histoire naturelle, appliquée aux arts, principalement à l’agriculture et à l’économie rurale et domestique. Tome XXIV [Section 3]: Tableaux méthodiques d’histoire naturelle. Déterville, Paris. 84 + 4 + 85 + 238 + 18 + 34 pp. [21 March, Thompson et al. 1999: 456]

[B234] Latreille PA (1805) Histoire naturelle, genérale et particuliere, des crustacés et des insectes. Ouvrage faisant suite aux oeuvres de Leclerc de Buffon, et partie du cours complet d’histoire naturelle rédigé par C.S. Sonnini, membre de plusieurs Sociétés savantes. Tome quatorzième. F. Dufart, Paris, 432 pp. + pls. CIV–CXII. [20 February to 21 March, Thompson et al. 1999: 457]

[B235] Latreille PA (1825) Familles naturelles du règne animal, exposées succinctement et dans un ordre analytique, avec l’indication de leurs genres. J.-B. Baillière, Paris, 570 pp. [16 May, Evenhuis 1997: 441] 10.5962/bhl.title.16094

[B236] Latreille PA (1829) Suite et fin des insectes. In: Cuvier GCLD, Le règne animal distribué d’après son organisation, pour servir de base a l’histoire naturelle des animaux et d’introduction a l’anatomie comparée. Avec figures dessinees d’après nature. Nouvelle édition, revue et augmentée. Tome V. Déterville & Crochard, Paris. xxiv + 556 pp. [11 April, Evenhuis 1997: 174]

[B237] LeConte JL (Ed) (1859) The complete writings of Thomas Say on the entomology of North America. Bailliere, New York. Vol. II, 814 pp. [before 13 December, Evenhuis 1997: 446]

[B238] Liljesthröm G (1980) Nuevos citas de dipteros taquinidos para la Republica Argentina (Insecta). Revista de la Sociedad Entomológica Argentina 39: 135–136. [before 24 November 1981, CAL date stamp]

[B239] Liljesthröm G (1992) Revision de las especies de los generos *Trichopoda* Berthold, *Trichopodopsis* Townsend y *Eutrichopodopsis* Blanchard descriptas para la Republica Argentina. Revista de la Sociedad Entomológica Argentina 50 [1991]: 51–71.

[B240] Loew H (1863) Diptera Americae septentrionalis indigena. Centuria quarta. Berliner Entomologische Zeitschrift 7: 275–326. [Also published in 1864 with other papers by Loew, pp. 159–210.] [December, Thompson et al. 1999: 464]

[B241] Lozada PW Valencia L Díaz-B W Burckhardt D (2005) Material tipo de insectos en el Laboratorio de Sanidad Vegetal, SENASA, Lima, Perú. Revista Peruana de Biología 12: 457–461. 10.15381/rpb.v12i3.2422

[B242] Macquart PJM (1834) Insectes diptères du nord de la France. Athéricères: créophiles, oestrides, myopaires, conopsaires, scénopiniens, céphalopsides. [Vol. 5.] L. Danel, Lille, 232 pp. + 6 pls. [Also published in 1834, Mémoires de la Société Royale des Sciences, de l’Agriculture et des Arts de Lille 1833: 137–368 + 6 pls.] [21 July (separate), October (journal), Evenhuis et al. 2016: 201]

[B243] Macquart PJM (1835) Histoire naturelle des insectes. Diptères. Tome deuxième. Roret, Paris, 703 pp. + [2 or more pages depending on copy (errata)]. [February, Evenhuis et al. 2016: 201] 10.5962/bhl.title.14274

[B244] Macquart PJM (1844) Diptères exotiques nouveaux ou peu connus. Tome deuxième. 3.^e^ partie [1843]. Roret, Paris, 304 pp. + 36 pls. [Also published in 1844, Mémoires de la Société Royale des Sciences, de l’Agriculture et des Arts de Lille 1842: 162–460 + 36 pls. Both the journal version and separate were generally thought to have been published in 1843 until the dating was revised by Evenhuis (1997: 513).] [February (separate), 5 August (journal), Evenhuis et al. 2016: 204]

[B245] Macquart PJM (1845) Nouvelles observations sur les insectes diptères de la tribu des tachinaires. Annales de la Société Entomologique de France (Sér. 2) 3: 237–296 + pls. 4–6. [The first part of this paper comprising pp. 237–280 was published in October 1845 and the second part comprising pp. 281–296 was published in December 1845 (Crosskey 1980: 1066). The entire paper is continuous and the only indication within the paper itself that publication was split was the placement of pls. 3–5 after p. 280 (pl. 3 belonging to a paper by a different author; Macquart’s pl. 6 was placed after p. 416).] [22 October (pp. 237–280), 22 December (pp. 281–296), Evenhuis et al. 2016: 204]

[B246] Macquart PJM (1846) Diptères exotiques nouveaux ou peu connus. [1.^er^] Supplément. Mémoires de la Société Royale des Sciences, de l’Agriculture et des Arts de Lille 1844: 133–364 + 20 pls. [Also published separately as his “Diptères exotiques nouveaux ou peu connus. Supplément” [I], pp. 5–238 + 20 pls., Roret, Paris, 1846.] [22 July (journal), 7 November (separate), Evenhuis et al. 2016: 205]

[B247] Macquart PJM (1851) Diptères exotiques nouveaux ou peu connus. Suite du 4.^e^ supplément publié dans les Mémoires de 1849. Mémoires de la Société Royale des Sciences, de l’Agriculture et des Arts de Lille 1850: 134–294 + pls. 15–28. [Also published separately as his “Diptères exotiques nouveaux ou peu connus. Supplément IV” [part], pp. 161–336 (including the combined index of the two parts of this supplement) + pls. 15–28, Roret, Paris, 1851.] [5 April (journal), later date (separate), Evenhuis et al. 2016: 206]

[B248] Maes JM (1999) Familia Tachinidae, pp. 1603–1612. In: Insectos de Nicaragua. Catálogo de los insectos y artrópodos terrestres de Nicaragua. Vol. III. Setab, Marena, Managua, Nicaragua, 700 pp.

[B249] Mallea AR Mácola GS García JG Bahamondes LA Suárez JH Lanatl SJ (1977) *Trichopodopsisgustavoi* n. sp. (Diptera – Tachinidae – Gymnosomatidae – Trichopodini) parasito de *Nezaraviridula* (L.) Stal. Revista de la Facultad de Ciencias Agrarias, Universidad Nacional de Cuyo 21: 21–31.

[B250] Marnef L (1965) *Lafuentemyiayanezi* nov. gen., nov. sp. de taquinido (Diptera) de Chile. Bulletin et Annales de la Société Royale d’Entomologie de Belgique 101: 243–250.

[B251] Marshall SA Wood DM González CR (2009) What’s in a kiss? An unusual oral exchange between a tachinid fly (*Myiopharuspirioni* Aldrich) and a larval leaf beetle (*Procalus* spec.) (Diptera: Tachinidae, Coleoptera: Chrysomelidae). Studia Dipterologica 15 [2008]: 242–244. [14 September, OS]

[B252] McAtee WL Wade JS (1951) Raymond Corbett Shannon 1894–1945. Proceedings of the Entomological Society of Washington 53: 211–222. [28 August, OS (Table of Contents of vol. 53)]

[B253] Meigen JW (1800) Nouvelle classification des mouches à deux ailes (Diptera L.) d’après un plan tout nouveau. J.J. Fuchs, Paris, 40 pp. [Publication suppressed by ICZN (1963). Author given as “J.G. Meigen” on title page.] [before 22 September, Evenhuis and Pape 2019: 187] 10.5962/bhl.title.119764

[B254] Meigen JW (1803) Versuch einer neuen GattungsEintheilung der europäischen zweiflügligen Insekten. Magazin für Insektenkunde 2: 259–281. [after 17 May, Evenhuis and Pape 2019: 187]

[B255] Meigen JW (1824) Systematische Beschreibung der bekannten europäischen zweiflügeligen Insekten. Vierter Theil. Schulz-Wundermann, Hamm. xii + 428 pp. + pls. 33–41. [after 24 September, Evenhuis and Pape 2019: 188]

[B256] Meigen JW (1838) Systematische Beschreibung der bekannten europäischen zweiflügeligen Insekten. Siebenter Theil oder Supplementband. Schulz, Hamm. xii + 434 + [1 (errata)] pp. + pls. 67–74. [21 September, Evenhuis and Pape 2019: 190]

[B257] Mesnil LP (1939) Essai sur les tachinaires (Larvaevoridae). Monographies publiées par les Stations et Laboratoires de Recherches Agronomiques 7: 67 + v pp.

[B258] Mesnil LP (1946) Revision des Phorocerini de l’Ancien Monde (Larvaevoridae). Encyclopédie Entomologique. Série B. Mémoires et Notes. II. Diptera 10: 37–80. [15 December, Evenhuis et. al. 2008: 41 (“Date of reprint; full volume was published on 26 March 1947”)]

[B259] Mesnil LP (1950) 64g. Larvaevorinae (Tachininae). Die Fliegen der Palaearktischen Region 10 (Lieferung 164): 105–160 + pls. VI–VII. [12 June, OS (wrapper of vol. 10, pt. 1)]

[B260] Mesnil LP (1966) 64g. Larvaevorinae (Tachininae). Die Fliegen der palaearktischen Region 10 (Lieferung 263): 881–928. [1 February, OS (wrapper of vol. 10, pt. 3)]

[B261] Mesnil LP (1971) 64g. Larvaevorinae (Tachininae). Die Fliegen der Palaearktischen Region 10 (Lieferung 286): 977–1024. [21 July, OS (wrapper of vol. 10, pt. 3)]

[B262] Mesnil LP (1973) 64g. Larvaevorinae (Tachininae). Die Fliegen der Palaearktischen Region 10 (Lieferung 299): 1169–1232. [25 September, OS (wrapper of vol. 10, pt. 3)]

[B263] Mesnil LP (1974) 64g. Larvaevorinae (Tachininae). Die Fliegen der Palaearktischen Region 10 (Lieferung 304): 1233–1304. [19 July, OS (wrapper of vol. 10, pt. 3)]

[B264] Mik J (1894) Dipterologische Miscellen. (2. Serie.) IV. Wiener Entomologische Zeitung 13: 49–54. [28 February, OS] 10.5962/bhl.part.13242

[B265] Molina-Ochoa J Carpenter JE Heinrichs EA Foster JE (2003) Parasitoids and parasites of *Spodopterafrugiperda* (Lepidoptera: Noctuidae) in the Americas and Caribbean Basin: an inventory. Florida Entomologist 86: 254–289. [30 September, OS (wrapper of vol. 86, no. 3)] 10.1653/0015-4040(2003)086[0254:PAPOSF]2.0.CO;2

[B266] Mulieri PR Patitucci LD Bachmann AO O’Hara JE (2013) The type specimens of Tachinidae (Diptera) housed in the Museo Argentino de Ciencias Naturales “Bernardino Rivadavia”, Buenos Aires. Zootaxa 3670: 157–176. [12 June, OS] 10.11646/zootaxa.3670.2.3 26438932

[B267] Nihei SS (2015) Revision of the Neotropical Exoristini (Diptera, Tachinidae): the status of the genera *Epiplagiops* and *Tetragrapha*. Journal of Insect Science 15 (35): 9 pp. [5 April, journal website] 10.1093/jisesa/iev023 PMC4535485 25843588

[B268] Nihei SS (2016) Family Tachinidae, pp. 904–949. In: Wolff M Nihei SS Carvalho CJB de (Eds) , Catalogue of Diptera of Colombia.Zootaxa 4122: 1–949. [14 June, OS] 10.11646/zootaxa.4122.1.76 27395249

[B269] Nihei SS Dios RVP (2016) Nomenclatural acts for some Neotropical Tachinidae (Diptera). Papéis Avulsos de Zoologia 56: 177–181. [17 November, OS] 10.11606/0031-1049.2016.56.16

[B270] Nishida GM (Ed) (1992) Hawaiian terrestrial arthropod checklist. Bishop Museum Press, Honolulu. viii + 262 pp. [10 September, OS]

[B271] O’Hara JE (1983) Classification, phylogeny and zoogeography of the North American species of *Siphona* Meigen (Diptera: Tachinidae). Quaestiones Entomologicae 18 [1982]: 261–380. [before 1 February, date received by JEOH]

[B272] O’Hara JE (1985) *Actia* Robineau-Desvoidy, 1830 (Insecta, Diptera): request for designation of type species. Z.N.(S.) 2491. Bulletin of Zoological Nomenclature 42: 93–97. [2 April, OS (wrapper of vol. 42, pt. 1)] 10.5962/bhl.part.872

[B273] O’Hara JE (1989) Systematics of the genus group taxa of the Siphonini (Diptera: Tachinidae). Quaestiones Entomologicae 25: 1–229. [May, OS (wrapper of vol. 25, nos. 1/2)]

[B274] O’Hara JE (2002) Revision of the Polideini (Tachinidae) of America north of Mexico. Studia Dipterologica. Supplement 10: 1–170. [30 April, OS]

[B275] O’Hara JE (2013a) A visit to the Vienna Museum with a brief history of the tachinid collection. The Tachinid Times 26: 30–38. [28 February, newsletter website]

[B276] O’Hara JE (2013b) History of tachinid classification (Diptera, Tachinidae). ZooKeys 316: 1–34. [11 July, OS] 10.3897/zookeys.316.5132 PMC3713332 23878512

[B277] O’Hara JE Cerretti P (2016) Annotated catalogue of the Tachinidae (Insecta, Diptera) of the Afrotropical Region, with the description of seven new genera. ZooKeys 575: 1–344. [31 March, OS] 10.3897/zookeys.575.6072 PMC4829880 27110184

[B278] O’Hara JE Cerretti P Pape T Evenhuis NL (2011) Nomenclatural studies toward a world list of Diptera genus-group names. Part II: Camillo Rondani. Zootaxa 3141: 1–268. [23 December, OS] 10.11646/zootaxa.3141.1.1

[B279] O’Hara JE Henderson SJ Wood DM (2020) Preliminary checklist of the Tachinidae (Diptera) of the world. Version 2.1. PDF document, 1039 pp. Available at: http://www.nadsdiptera.org/Tach/WorldTachs/Checklist/Tachchlist_ver2.1.pdf. [5 March, OS]

[B280] O’Hara JE Shima H Zhang C-t (2009) Annotated catalogue of the Tachinidae (Insecta: Diptera) of China. Zootaxa 2190: 1–236. [6 August, OS] 10.11646/zootaxa.2190.1.1

[B281] O’Hara JE Wood DM (1998) Tachinidae (Diptera): nomenclatural review and changes, primarily for America north of Mexico. Canadian Entomologist 130: 751–774. [before 13 January 1999, CAL date stamp] 10.4039/Ent130751-6

[B282] O’Hara JE Wood DM (2004) Catalogue of the Tachinidae (Diptera) of America north of Mexico. Memoirs on Entomology, International 18: iv + 410 pp. [3 January, OS]

[B283] Orbigny CVD d’ (Ed) (1846) [Livraisons 85–92], pp. 1–640. Dictionnaire universel d’histoire naturelle résumant et complétant tous les faits présentés par les encyclopédies, les anciens dictionnaires scientifiques, les oeuvres complètes de Buffon, et les meilleurs traités spéciaux sur les diverses branches des sciences naturelles; — donnant la description des êtres et des divers phénomènes de la nature, l’étymologie et la définition des noms scientifiques, les principales applications des corps organiques et inorganiques à l’agriculture, à la médecine, aux arts industriels, etc.; dirigé par M. Charles d’Orbigny, et enrichi d’un magnifique atlas de planches gravées sur acier. Tome huitième. C. Renard, Paris, 766 pp. [21 September (livr. 85–86), 19 October (livr. 87–88), 9 November (livr. 89–90), 14 December (livr. 91–92), Evenhuis 1990: 224]

[B284] Orbigny CVD d’ (Ed) (1849) [Livraisons 148–149], pp. 193–320. Dictionnaire universel d’histoire naturelle résumant et complétant tous les faits présentés par les encyclopédies, les anciens dictionnaires scientifiques, les oeuvres complètes de Buffon, et les meilleurs traités spéciaux sur les diverses branches des sciences naturelles; — donnant la description des êtres et des divers phénomènes de la nature, l’étymologie et la définition des noms scientifiques, les principales applications des corps organiques et inorganiques à l’agriculture, à la médecine, aux arts industriels, etc.; dirigé par M. Charles d’Orbigny, et enrichi d’un magnifique atlas de planches gravées sur acier. Tome treizième. C. Renard, Paris, 384 pp. [10 September (livr. 148–149), Evenhuis 1990: 225]

[B285] Papavero N Ibáñez-Bernal S (2001) Contributions to a history of Mexican dipterology.– Part 1. Entomologists and their works before the *Biologia Centrali-Americana*. Acta Zoológica Mexicana (nueva serie) 84: 65–173.

[B286] Parker HL (1953) Miscellaneous notes on South American dipterous parasites. Bollettino del Laboratorio di Entomologia Agraria “Filippo Silvestri” di Portici 12: 45–73. [10 April, OS]

[B287] Parker HL Berry A Guido AS (1951) Host-parasite and parasite-host lists of insects reared in the South American Parasite Laboratory during the period 1940–1946. Revista de la Asociación de Ingenieros Agrónomos de Montevideo 23: 15–112. [Also published separately with same title, 100 + [1] pp., Montevideo, 1953. Only this later version was seen by the authors.]

[B288] Patitucci LD Mulieri PR Domínguez MC Mariluis JC (2015) The type specimens of Calyptratae (Diptera) housed in non-traditional institutions in Argentina. Zootaxa 3905: 557–572. [14 January, OS] 10.11646/zootaxa.3905.4.8 25661231

[B289] Persson PI (1971) “Eugenies resa”. Localities, dates and labels of the insects collected during the voyage around the world by the Swedish frigate “Eugenie” in the years 1851–1853. Entomologisk Tidskrift 92: 164–172.

[B290] Philippi RA (1865) Aufzählung der chilenischen Dipteren. Verhandlungen der Kaiserlich-Königlichen Zoologisch-Botanischen Gesellschaft in Wien 15 (Abhandlungen): 595–782. [31 December+, O’Hara et al. 2011: 230] 10.5962/bhl.title.9295

[B291] Pirán AA (1973) Everardo E. Blanchard. Revista de la Sociedad Entomológica Argentina 34: 29–30.

[B292] Ravlin FW Stehr FW (1984) Revision of the genus *Archytas* (Diptera: Tachinidae) from America north of Mexico. Miscellaneous Publications of the Entomological Society of America 58: 1–59. [before 22 June, CAL date stamp]

[B293] Reed CS (1907) Entomología económica. Biología de la *LaoraVariabilis* F. Ph. y noticias acerca de un díptero cuya larva vive como parásito en su oruga. Boletín de la Sociedad Agrícola del Sur (Concepción) 7: 1008–1014, 1045–1051.

[B294] Reed EC (1888) Catálogo de los insectos dípteros de Chile. Anales de la Universidad de Chile 73: 271–316. 10.5962/bhl.title.8562

[B295] Reinhard HJ (1931) Revision of the American parasitic flies belonging to the genus *Winthemia*. Proceedings of the United States National Museum 79 (No. 2886) [1932]: 1–54 + 1 pl. [24 November, OS (Table of Contents of vol. 79)] 10.5479/si.00963801.79-2886.1

[B296] Reinhard HJ (1934) Revision of the American two-winged flies belonging to the genus *Cuphocera*. Proceedings of the United States National Museum 83 (No. 2974) [1937]: 45–70. [25 October, OS (Table of Contents of vol. 83)] 10.5479/si.00963801.2974.45

[B297] Reinhard HJ (1939) New genera and species of muscoid Diptera. Bulletin of the Brooklyn Entomological Society 34: 61–74. [29 April, OS (wrapper of vol. 34, no. 2)]

[B298] Reinhard HJ (1943) New genera of North American muscoid Diptera. Canadian Entomologist 75: 163–169. [21 October, OS (vol. 75, p. 178)] 10.4039/Ent75163-9

[B299] Reinhard HJ (1946) The tachinid genera *Pseudochaeta* and *Phaenopsis* in North America (Diptera). Canadian Entomologist 78: 111–121. [6 December, OS (vol. 78, p. 130)] 10.4039/Ent78111-6

[B300] Reinhard HJ (1956) A synopsis of the tachinid genus *Leucostoma* (Diptera). Journal of the Kansas Entomological Society 29: 155–168. [20 December, OS (wrapper of vol. 29, no. 4)]

[B301] Reinhard HJ (1964) Parasitic flies of the genera *Helioprosopa* and *Euhelioprosopa* (Diptera: Tachinidae). Pan-Pacific Entomologist 40: 117–124. [26 June, OS (wrapper of vol. 40, no. 2)]

[B302] Richards LLE (1973) A revision of the *Peleteria* (Diptera: Tachinidae) of America north of Mexico. Unpublished Ph. D. thesis, Washington State University, Pullman. xvii + 255 pp.

[B303] Riley CV (1871) Third annual report on the noxious, beneficial and other insects, of the State of Missouri. Annual Report of the State Board of Agriculture, Missouri 6 [1870]: 1–175. [Also published separately in 1871 with the same pagination, Jefferson City.]

[B304] Ripa SR Rojas PS Velasco G (1995) Releases of biological control agents of insect pests on Easter Island (Pacific Ocean). Entomophaga 40: 427–440. 10.1007/BF02373730

[B305] Robineau-Desvoidy J-B (1830) Essai sur les myodaires. Mémoires présentés par divers savans a l’Académie Royale des Sciences de l’Institut de France. Sciences Mathématiques et Physiques (Sér. 2) 2: 813 pp. [6 June, Evenhuis et al. 2010: 225]

[B306] Robineau-Desvoidy J-B (1849) [Sociétés Savantes: M. Robineau-Desvoidy lit une nouvelle suite de son ouvrage sur les myodaires des environs de Paris.] Revue et Magasin de Zoologie Pure et Appliquée (Sér. 2) 1: 158. [3 April, Evenhuis et al. 2010: 229]

[B307] Robineau-Desvoidy J-B (1850) Myodaires des environs de Paris. (Suite.) Annales de la Société Entomologique de France (Sér. 2) 8: 183–209. [June, Evenhuis et al. 2010: 229]

[B308] Robineau-Desvoidy J-B (1863a) Histoire naturelle des diptères des environs de Paris. Oeuvre posthume du D^r^ Robineau-Desvoidy publiée par les soins de sa famille, sous la direction de M. H. Monceaux. Tome premier. V. Masson et fils, Paris; F. Wagner, Leipzig; and Williams & Norgate, London. xvi + 1143 pp. [11 January, Evenhuis et al. 2010: 232] 10.5962/bhl.title.8550

[B309] Robineau-Desvoidy J-B (1863b) Histoire naturelle des diptères des environs de Paris. Oeuvre posthume du D^r^ Robineau-Desvoidy publiée par les soins de sa famille, sous la direction de M. H. Monceaux. Tome second. Victor Masson et fils, Paris; Franz Wagner, Leipzig; and Williams & Norgate, London, 920 pp. [11 January, Evenhuis et al. 2010: 232] 10.5962/bhl.title.8550

[B310] Rohlfien K Ewald B (1974) Katalog der in den Sammlungen des ehemaligen Deutschen Entomologischen Institutes aufbewahrten Typen — XI. Beiträge zur Entomologie 24: 107–147.

[B311] Rondani C (1845) Descrizione di due generi nuovi di insetti ditteri. Memoria duodecima per servire alla ditterologia italiana. Nuovi Annali delle Scienze Naturali e Rendiconto delle Sessioni della Società Agraria e dell’Accademia delle Scienze dell’Istituto di Bologna (Ser. 2) 3: 25–36 + 1 pl. [15 February, O’Hara et al. 2011: 243]

[B312] Rondani C (1850) Osservazioni sopra alquante specie di esapodi ditteri del Museo Torinese. Nuovi Annali delle Scienze Naturali e Rendiconto delle Sessioni della Società Agraria e dell’Accademia delle Scienze dell’Istituto di Bologna (Ser. 3) 2: 165–197 + 1 pl. [14 November, O’Hara et al. 2011: 245]

[B313] Rondani C (1856) Dipterologiae italicae prodromus. Vol: I. Genera italica ordinis dipterorum ordinatim disposita et distincta et in familias et stirpes aggregata. A. Stocchi, Parmae [= Parma]. 226 + [2] pp. [10 September, O’Hara et al. 2011: 246] 10.5962/bhl.title.8160

[B314] Rondani C (1857) Dipterologiae italicae prodromus. Vol: II. Species italicae ordinis dipterorum in genera characteribus definita, ordinatim collectae, methodo analitica distinctae, et novis vel minus cognitis descriptis. Pars prima. Oestridae: Syrpfhidae: Conopidae. A. Stocchi, Parmae [= Parma], 264 pp. + 1 pl. [after 20 September, O’Hara et al. 2011: 246]

[B315] Rondani C (1859) Dipterologiae italicae prodromus. Vol: III. Species italicae ordinis dipterorum in genera characteribus definita, ordinatim collectae, methodo analitica distinctae, et novis vel minus cognitis descriptis. Pars secunda. Muscidae. Siphoninae et (partim) Tachininae. A. Stocchi, Parmae [= Parma]. 243 + [1] pp. + 1 pl. [31 December+, O’Hara et al. 2011: 246]

[B316] Rondani C (1863) Diptera exotica revisa et annotata novis nonnullis descriptis. Eredi Soliani, Modena, 99 pp. + 1 pl. [The title page has “nonnullis” but page 1 has “nonullis”. This is the separate that came out before the journal version with a different title, here as Rondani (1864).] [31 December+, O’Hara et al. 2011: 249]

[B317] Rondani C (1864) Dipterorum species et genera aliqua exotica revisa et annotata novis nonullis descriptis. Archivio per la Zoologia, l’Anatomia e la Fisiologia 3: 1–99 + pl. V. [This is the journal version that came out after the separate with a different title, here as Rondani (1863).] [May, O’Hara et al. 2011: 250]

[B318] Rondani C (1865) Diptera italica non vel minus cognita descripta vel annotata observationibus nonnullis additis. Fasc. II. Muscidae. Atti della Società Italiana di Scienze Naturali 8: 193–231. [May, O’Hara et al. 2011: 250]

[B319] Rossi Belgrano A Rossi Belgrano M (2018) Juan Brèthes (Frère Judulien Marie). Primer entomólogo del Museo Nacional. Colaborador de Florentino Ameghino. Vida, obra y familia del ilustre naturalista. A.A. Rossi, Buenos Aires, 158 pp.

[B320] Sabrosky CW (1950) Notes on Trichopodini (Diptera, Larvaevoridae), with description of a new parasite of cotton stainers in Puerto Rico. Journal of the Washington Academy of Sciences 40: 361–371. [15 November, OS]

[B321] Sabrosky CW (1952) A new larvaevorid fly parasitic on tortoise beetles in South America (Diptera). Journal of the Washington Academy of Sciences 42: 325–327. [15 October, OS (reprint date; journal version published in issue 10, comprising pp. 313–344, on 20 October according to wrapper of vol. 42)]

[B322] Sabrosky CW (1955) The taxonomic status of the armyworm parasite known as *Archytaspiliventris* (van der Wulp) (Diptera: Larvaevoridae). Florida Entomologist 38: 77–83. [before 20 June, CAL date stamp] 10.2307/3492821

[B323] Sabrosky CW (1980) A revised key to the Nearctic species of *Lespesia* (Diptera: Tachinidae). Annals of the Entomological Society of America 73: 63–73. [15 January, OS (vol. 73, p. i)] 10.1093/aesa/73.1.63

[B324] Sabrosky CW (1981) A partial revision of the genus *Eucelatoria* (Diptera, Tachinidae), including important parasites of *Heliothis*. United States Department of Agriculture. Technical Bulletin 1635: iv + 18 pp. [July, OS]

[B325] Sabrosky CW (1999) Family-group names in Diptera. An annotated catalog. Myia 10: 1–360. [before 7 April, Evenhuis et al. 2015: 318]

[B326] Sabrosky CW Arnaud PH Jr (1965) Family Tachinidae (Larvaevoridae), pp. 961–1108. In: Stone A, Sabrosky CW, Wirth WW, Foote RH, Coulson JR (Eds), A catalog of the Diptera of America north of Mexico. United States Department of Agriculture. Agriculture Handbook 276: iv + 1696 pp. [23 August, Evenhuis 2008b: 397]

[B327] Sauer HFG (1946) Constatação de himenópteros e dipteros entomófagos no Estado de Sao Paulo. Boletím fitossanitário 3: 7–23. [Paper not seen but tachinid host data cited in Guimarães (1977c).]

[B328] Schiner JR (1868) Diptera, vi + 388 pp. + 4 pls. In: Reise der österreichischen Fregatte *Novara* um die Erde in den Jahren 1857, 1858, 1859 unter den Befehlen des Commodore B. von Wüllerstorf-Urbair. Zoologischer Theil. Zweiter Band. 1. Abtheilung. K. Gerold’s Sohn, Wien [= Vienna]. [30 April, Evenhuis 1997: 696]

[B329] Shima H (1986) A systematic study of the genus *Linnaemya* Robineau-Desvoidy from Japan and the Oriental Region (Diptera: Tachinidae). Sieboldia 5: 1–96. [31 March, date on reprint]

[B330] Silva TC Lemos RNS Moreira AA Araujo JRG Medeiros FR Castellani MA (2008) Parasitoids associated with *Spodopterafrugiperda* (Lepidoptera: Noctuidae) in corn in the state of Maranhão, Brazil. Boletín de Sanidad Vegetal, Plagas 34: 493–500.

[B331] Smith MA Wood DM Janzen DH Hallwachs W Hebert PDN (2007) DNA barcodes affirm that 16 species of apparently generalist tropical parasitoid flies (Diptera, Tachinidae) are not all generalists. Proceedings of the National Academy of Sciences of the United States of America 104: 4967–4972. [20 March, OS] 10.1073/pnas.0700050104 17360352 PMC1821123

[B332] Stein P (1924) Die verbreitetsten Tachiniden Mitteleuropas nach ihren Gattungen und Arten. Archiv für Naturgeschichte. Abteilung A 90(6): 1–271.

[B333] Stireman JO III Cerretti P O’Hara JE Blaschke JD Moulton JK (2019) Molecular phylogeny and evolution of world Tachinidae (Diptera). Molecular Phylogenetics and Evolution 139: 106358. [22 December 2018 (online version), final version published in vol. 139 (October 2019 issue)] 10.1016/j.ympev.2018.12.002 30584917

[B334] Stireman JO III O’Hara JE Cerretti P Inclan DJ (2016) Tachinid collecting in temperate South America. Expeditions of the World Tachinidae Project. Part III: Chile. The Tachinid Times 29: 20–40. [16 March, newsletter website]

[B335] Strobl G (1910) Die Dipteren von Steiermark. II. Nachtrag. Mitteilungen des Naturwissenschaftlichen Vereines für Steiermark 46 [1909]: 45–293. [before 19 October, date stamp on p. 3 of vol. 46 in Biodiversity Heritage Library]

[B336] Sun X-k Marshall SA (2003) Systematics of *Phasia* Latreille (Diptera: Tachinidae). Zootaxa 276: 1–320. [29 August, OS] 10.11646/zootaxa.276.1.1

[B337] Terán JB (1974) Lista preliminar de dipteros parasiticos de otros insectos en Venezuela. Revista de la Facultad de Agronomía de la Universidad Central de Venezuela 23: 1–85. [July, OS (“impreso durante julio de 1974”, unnumbered page after p. 85)]

[B338] Thompson FC (1974) Corrections and restrictions of the type localities of some Neotropical Syrphidae (Diptera). Revista Brasileira de Entomologia 18: 1–7. [20 May, OS]

[B339] Thompson FC Evenhuis NL Sabrosky CW (1999) Bibliography of the family-group names of Diptera. Myia 10: 361–574. [before 7 April, Evenhuis et al. 2015: 319]

[B340] Thompson WR (1961) The tachinids (Diptera) of Trinidad. I. The voriines. Transactions of the American Entomological Society 87: 21–44 + 5 pls. [9 September, OS]

[B341] Thompson WR (1963a) The tachinids of Trinidad. II. Echinomyiines, dexiines, and allies. Canadian Journal of Zoology 41: 335–576. 10.1139/z63-029

[B342] Thompson WR (1963b) The tachinids of Trinidad. IV. Winthemiines. Canadian Entomologist 95: 953–995. [28 October, OS (vol. 95, p. 1120)] 10.4039/Ent95953-9

[B343] Thompson WR (1964) The tachinids of Trinidad. VI. The larviparous goniines of the carceliine type (Diptera, Tachinidae). Studia Entomologica 7: 97–151. [10 December, OS (vol. 7, p. iv)]

[B344] Thompson WR (1966) The tachinids of Trinidad. VII. The larviparous goniines with broad cheeks (Dipt. Tachinidae). Studia Entomologica 8 [1965]: 353–434. [28 February, OS (vol. 8, p. iv)]

[B345] Thompson WR (1968) The tachinids of Trinidad. VIII. Phorocerines. Memoirs of the Entomological Society of Canada 56: 1–207. [13 September, Anonymous 1995: 989] 10.4039/entm10056fv

[B346] Thomson CG (1869) Diptera. Species novas descripsit, pp. 443–614 + pl. 9. In: Kongliga svenska fregatten Eugenies resa omkring jorden under befäl af C.A. Virgin, åren 1851–1853. Vetenskapliga iakttagelser på H.M. konung Oscar den förstes befallning utgifna af K. Svenska Vetenskaps-Akademien. Vol. II. Zoologi. 1. Insecta. P.A. Norstedt & Söner, Stockholm. [1868], 617 pp. + 9 pls. [10 February–10 March, Evenhuis 1997: 770]

[B347] Times Books (2007) The Times comprehensive atlas of the world. 12th edition. Times Books, London. 67 + [2] pp. + 125 pls. + 223 (Glossary and Index) + [1 (Acknowledgements)] pp. [Each of the 125 plates spans 2 pages.]

[B348] Toma R (2010) Contribuição ao conhecimento de espécies venezuelanas de *Lespesia* Robineau-Desvoidy (Diptera, Tachinidae, Exoristinae), com descrições de novas espécies. Revista Brasileira de Entomologia 54: 165–172. 10.4039/entm10056fv

[B349] Toma R Guimarães JH (2002) Estudo taxonômico de *Leschenaultia* Robineau-Desvoidy (Diptera, Tachinidae). Revista Brasileira de Entomologia 46: 33–70. [31 March, OS] 10.1590/S0085-56262002000100006

[B350] Toma R Nihei SS (2006) Catálogo do material-tipo de Tachinidae (Diptera) depositado no Museu de Zoologia da Universidade de São Paulo. Revista Brasileira de Entomologia 50: 240–256. 10.1590/S0085-56262006000200006

[B351] Townsend CHT (1891) Notes on North American Tachinidae sens. str. with descriptions of new genera and species. Paper II. Transactions of the American Entomological Society 18: 349–382. [December, Evenhuis et al. 2015: 333]

[B352] Townsend CHT (1892a) Notes on North American Tachinidae, with descriptions of new genera and species.—Paper V. [Concl.] Canadian Entomologist 24: 77–82. [2 April, Evenhuis et al. 2015: 334] 10.4039/Ent2477-4

[B353] Townsend CHT (1892b) Notes on North American Tachinidae sens. str. with descriptions of new genera and species. Paper III. Transactions of the American Entomological Society 19: 88–132. [June, Evenhuis et al. 2015: 334]

[B354] Townsend CHT (1892c) Notes on North American Tachinidae, with descriptions of new genera and species.—Paper VI. Canadian Entomologist 24: 165–172. [4 July, OS (vol. 24, p. 184)] 10.4039/Ent24165-7

[B355] Townsend CHT (1893) Description of a new and interesting phasiid-like genus of Tachinidae s. str. Psyche 6: 429–430. [1 April, Evenhuis et al. 2015: 335] 10.1155/1893/25860

[B356] Townsend CHT (1897) Contributions from the New Mexico Biological Station.—No II. (continued). On a collection of Diptera from the lowlands of the Rio Nautla, in the state of Vera Cruz. II. [Concl.] Annals and Magazine of Natural History, Ser. 6, 20: 272–291. [31 August, Evenhuis 2003b: 34] 10.1080/00222939708680625

[B357] Townsend CHT (1908) The taxonomy of the muscoidean flies, including descriptions of new genera and species. Smithsonian Miscellaneous Collections 51 (2) [= pub. 1803]: 1–138. [before 30 June, Evenhuis et al. 2015: 342] 10.1007/s11606-014-3121-5

[B358] Townsend CHT (1909) Descriptions of some new Tachinidae. Annals of the Entomological Society of America 2: 243–250. [30 December, Evenhuis et al. 2015: 342] 10.1093/aesa/2.4.243

[B359] Townsend CHT (1911) Announcement of further results secured in the study of muscoid flies. Annals of the Entomological Society of America 4: 127–152. [13 July, Evenhuis et al. 2015: 343] 10.1093/aesa/4.2.127

[B360] Townsend CHT (1912a) Foundation of some new genera and species of muscoid flies mainly on reproductive and early-stage characters. Journal of the New York Entomological Society 20: 107–119. [June, Evenhuis et al. 2015: 343]

[B361] Townsend CHT (1912b) Descriptions of new genera and species of muscoid flies from the Andean and Pacific Coast regions of South America. Proceedings of the United States National Museum 43 (No. 1935) [1913]: 301–367. [22 November, OS (Table of Contents of vol. 43] 10.5479/si.00963801.1935.301

[B362] Townsend CHT (1913a) Two new generic names in Muscoidea (Dip.). Entomological News 24: 133. [28 February, Evenhuis et al. 2015: 343]

[B363] Townsend CHT (1913b) On *Trichiopoda* Latreille, *Polistomyia* Townsend and *Trichopodopsis* new genus. Journal of the New York Entomological Society 21: 147–148 (313, a correction). [June, Evenhuis et al. 2015: 344]

[B364] Townsend CHT (1914a) New muscoid flies, mainly Hystriciidae and Pyrrhosiinae from the Andean Montanya. [Cont.] Insecutor Inscitiae Menstruus 2: 10–16. [31 January, Evenhuis et al. 2015: 344]

[B365] Townsend CHT (1914b) New muscoid flies, mainly Hystriciidae and Pyrrhosiinae from the Andean Montanya. [Cont.] Insecutor Inscitiae Menstruus 2: 42–48. [30 March, Evenhuis et al. 2015: 345]

[B366] Townsend CHT (1914c) New muscoid flies, mainly Hystriciidae and Pyrrhosiinae from the Andean Montanya. [Cont.] Insecutor Inscitiae Menstruus 2: 123–128. [31 August, Evenhuis et al. 2015: 345]

[B367] Townsend CHT (1914d) New muscoid flies, mainly Hystriciidae and Pyrrhosiinae from the Andean Montanya. [Cont.] Insecutor Inscitiae Menstruus 2: 133–144. [2 October, Evenhuis et al. 2015: 345]

[B368] Townsend CHT (1915a) New muscoid flies, mainly Hystriciidae and Pyrrhosiinae from the Andean Montanya. [Concl.] Insecutor Inscitiae Menstruus 2 [1914]: 183–187. [14 January, Evenhuis et al. 2015: 345] 10.5962/bhl.part.9593

[B369] Townsend CHT (1915b) Proposal of new muscoid genera for old species. Proceedings of the Biological Society of Washington 28: 19–23. [12 February, Evenhuis et al. 2015: 345]

[B370] Townsend CHT (1915c) New Masiceratidae and Dexiidae from South America. Journal of the New York Entomological Society 23: 61–68. [March, Evenhuis et al. 2015: 345]

[B371] Townsend CHT (1915d) New Andean spallanzaniine flies. Insecutor Inscitiae Menstruus 3: 63–69. [20 July, Evenhuis et al. 2015: 346] 10.5962/bhl.part.8936

[B372] Townsend CHT (1915e) New Neotropical muscoid flies. Proceedings of the United States National Museum 49 (No. 2115) [1916]: 405–440. [27 November, OS (Table of Contents of vol. 49)] 10.5479/si.00963801.2115.405

[B373] Townsend CHT (1915f) Nine new tropical American genera of Muscoidea. Insecutor Inscitiae Menstruus 3: 91–97. [11 December, Evenhuis et al. 2015: 346] 10.5962/bhl.part.8939

[B374] Townsend CHT (1915g) Synonymical notes on Muscoidea. Insecutor Inscitiae Menstruus 3: 115–122. [11 December, Evenhuis et al. 2015: 346]

[B375] Townsend CHT (1915h) New western and southwestern Muscoidea. Journal of the New York Entomological Society 23: 216–234. [December, Evenhuis et al. 2015: 346]

[B376] Townsend CHT (1916a) Diagnoses of new genera of muscoid flies founded on old species. Proceedings of the United States National Museum 49 (No. 2128): 617–633. [14 January, OS (Table of Contents of vol. 49)] 10.5479/si.00963801.2128.617

[B377] Townsend CHT (1916b) Designations of muscoid genotypes, with new genera and species. Insecutor Inscitiae Menstruus 4: 4–12. [31 March, Evenhuis et al. 2015: 346]

[B378] Townsend CHT (1916c) New muscoid genera (Dip.). Entomological News 27: 178. [31 March, Evenhuis et al. 2015: 346]

[B379] Townsend CHT (1916d) Some new North American muscoid forms. Insecutor Inscitiae Menstruus 4: 73–78. [23 October, Evenhuis et al. 2015: 347] 10.5962/bhl.part.8952

[B380] Townsend CHT (1916e) New genera and species of muscoid flies. Proceedings of the United States National Museum 51 (No. 2125) [1917]: 299–323. [28 October, OS (Table of Contents of vol. 51)] 10.5479/si.00963801.2152.299

[B381] Townsend CHT (1917) Miscellaneous muscoid notes and descriptions. Insecutor Inscitiae Menstruus 4 [1916]: 121–128. [12 January, Evenhuis et al. 2015: 347]

[B382] Townsend CHT (1919a) New muscoid genera, species and synonymy (Diptera). [Concl.] Insecutor Inscitiae Menstruus 6 [1918]: 157–182. [11 January, Evenhuis et al. 2015: 347]

[B383] Townsend CHT (1919b) New genera and species of muscoid flies. Proceedings of the United States National Museum 56 (No. 2301) [1920]: 541–592. [15 December, OS (Table of Contents of vol. 56)] 10.5479/si.00963801.2301.541

[B384] Townsend CHT (1927a) Synopse dos generos muscideos da região humida tropical da America, com generos e especies novas. Revista do Museu Paulista 15: 203–385 + 4 pls. + [4 (errata)] pp. [20 January, Evenhuis et al. 2015: 350]

[B385] Townsend CHT (1927b) Errata to “Synopse dos generos muscoideos da regiao humida tropical da America, com generos e especies novos” in volume XV of the Revista do Museu Paulista. Privately published, Lima. [12] pp. [10 March, Evenhuis et al. 2015: 351]

[B386] Townsend CHT (1927c) New muscoid flies in the collection of the Deutsches Entomologisches Institut in Berlin. Entomologische Mitteilungen 16: 277–287. [1 July, Evenhuis et al. 2015: 351]

[B387] Townsend CHT (1928a) New Muscoidea from humid tropical South America. Wiener Entomologische Zeitung 44: 143–154. [15 February, Evenhuis et al. 2015: 351]

[B388] Townsend CHT (1928b) New muscoid genera and species of the coasts of Perú and Chile. Revista Chilena de Historia Natural 31 [1927]: 158–164. [early 1928, Evenhuis et al. 2015: 352]

[B389] Townsend CHT (1929) New species of humid tropical American Muccoidea. Revista Chilena de Historia Natural 32 [1928]: 365–382. [“Muccoidea” in title is a misspelling of “Muscoidea”.] [early 1929, Evenhuis et al. 2015: 352]

[B390] Townsend CHT (1931a) Notes on American oestromuscoid types. [Cont.] Revista de Entomologia 1: 65–104. [25 April, OS (vol. 1, p. iv)]

[B391] Townsend CHT (1931b) Notes on American oestromuscoid types. [Concl.] Revista de Entomologia 1: 157–183. [15 July, OS (vol. 1, p. iv)]

[B392] Townsend CHT (1931c) New genera and species of American oestromuscoid flies. [Cont.] Revista de Entomologia 1: 313–354. [5 September, OS (vol. 1, p. iv)]

[B393] Townsend CHT (1931d) New genera and species of American oestromuscoid flies. [Concl.] Revista de Entomologia 1: 437–479. [14 November, OS (vol. 1, p. iv)]

[B394] Townsend CHT (1932a) Notes on Old-World oestromuscoid types.—Part II. Annals and Magazine of Natural History (Ser. 10) 9: 33–57. [1 January, Evenhuis et al. 2015: 352] 10.1080/00222933208673463

[B395] Townsend CHT (1932b) Five new Brazilian oestromuscoid genera. Revista de Entomologia 2: 105–107. [23 January, OS (vol. 2, p. iv)]

[B396] Townsend CHT (1932c) A remarkable new genus and species of two-winged flies related to the Oestridae. Proceedings of the United States National Museum 82 (No. 2942) [1934]: 1–4. [11 October, OS (Table of Contents of vol. 82)] 10.5479/si.00963801.82-2942.1

[B397] Townsend CHT (1933) Two new generic names in Oestroidea. Revista de Entomologia 3: 527. [13 December, OS (vol. 3, p. iv)]

[B398] Townsend CHT (1934a) New Neotropical oestromuscoid flies. Revista de Entomologia 4: 201–212. [27 June, OS (vol. 4, p. iv)]

[B399] Townsend CHT (1934b) New Neotropical oestromuscoid flies. Revista de Entomologia 4: 390–406. [5 September, OS (vol. 4, p. iv)]

[B400] Townsend CHT (1934–1942) Manual of myiology in twelve parts. Privately published by Charles Townsend & Filhos, Itaquaquecetuba, São Paulo. [Each part individually paginated for a total of ca. 3760 pp.]

[B401] Townsend CHT (1935) New South American Oestroidea (Dipt.). Revista de Entomologia 5: 216–233. [26 June, OS (vol. 5, p. iii)]

[B402] Townsend CHT (1936a) A new phasiine parasite of *Dysdercus*. Revista de Entomologia 6: 489. [30 October, OS (vol. 6, p. iv)]

[B403] Townsend CHT (1936b) Manual of myiology in twelve parts. Part III. Oestroid classification and habits. Gymnosomatidae to Tachinidae. Privately published, Itaquaquecetuba, São Paulo, 249 pp. [An “Addenda and corrigenda” of four pages, numbered as pp. 251–255, was published later. Thompson et al. (1999: 537) noted that “Sabrosky received these addenda in March 1937”.] [31 December+, Evenhuis et al. 2015: 353]

[B404] Townsend CHT (1936c) Manual of myiology in twelve parts. Part IV. Oestroid classification and habits. Dexiidae and Exoristidae. Privately published, Itaquaquecetuba, São Paulo, 303 pp. [An “Addenda and corrigenda” of five pages, numbered as pp. 305–309, was published sometime in 1937 (Evenhuis et al. 2015: 354).] [31 December+, Evenhuis et al. 2015: 354]

[B405] Townsend CHT (1937a) Two new South American oestromuscarian genera (Dipt.). Revista de Entomologia 7: 115–117. [30 March, OS (vol. 7, p. iv)]

[B406] Townsend CHT (1937b) New fly parasites of *Dysdercus*. Revista de Entomologia 7: 316–318. [24 July, OS (vol. 7, p. iv)]

[B407] Townsend CHT (1938) Manual of myiology in twelve parts. Part VII. Oestroid generic diagnoses and data. Gymnosomatini to Senostomatini. Privately published, Itaquaquecetuba, São Paulo, 428 pp. [An “Addenda and corrigenda” of six pages, numbered as pp. 429–434, was published later. Thompson et al. (1999: 537) noted that “Sabrosky received these addenda on 1 September 1939”.] [31 December+, Evenhuis et al. 2015: 354]

[B408] Townsend CHT (1939a) Manual of myiology in twelve parts. Part VIII. Oestroid generic diagnoses and data. Microtropesini to Voriini. Privately published, Itaquaquecetuba, São Paulo, 405 pp. [An “Addenda and corrigenda” of two pages, numbered as pp. 407–408, was published later. Thompson et al. (1999: 537) noted that “Sabrosky received these addenda on 22 March 1940”.] [before 1 September, Evenhuis et al. 2015: 354]

[B409] Townsend CHT (1939b) Seven new genera of Brazilian oestromuscarian flies. Revista de Entomologia 10: 446–452. [4 September, OS (vol. 10, p. iv)]

[B410] Townsend CHT (1939c) Manual of myiology in twelve parts. Part IX. Oestroid generic diagnoses and data. Thelairini to Clythoini. Privately published, Itaquaquecetuba, São Paulo, 268 pp. [An “Addenda and corrigenda” of two pages, numbered as pp. 269–270, was published later.] [31 December+, Evenhuis et al. 2015: 355]

[B411] Townsend CHT (1940a) Manual of myiology in twelve parts. Part X. Oestroid generic diagnoses and data. Anacamptomyiini to Frontinini. Privately published, Itaquaquecetuba, São Paulo, 334 pp. [An “Addenda and corrigenda” of one page, numbered as p. 335, was published later. Thompson et al. (1999: 538) noted that “Sabrosky received this page on 23 October 1941 with part 11”.] [31 December+, Evenhuis et al. 2015: 355]

[B412] Townsend CHT (1940b) New oestroid flies from Brazil. Revista de Entomologia 11: 889–894. [31 December, OS (vol. 11, p. iv)]

[B413] Townsend CHT (1941) Manual of myiology in twelve parts. Part XI. Oestroid generic diagnoses and data. Goniini to Trypherini. Privately published, Itaquaquecetuba, São Paulo, 330 pp. [An “Addenda and corrigenda” of twelve pages, numbered as pp. 331–342, was published later. Thompson et al. (1999: 538) noted that “Sabrosky received these addenda on 22 June 1943”.] [before 23 October, Evenhuis et al. 2015: 355]

[B414] Tschorsnig H-P (1985) Taxonomie forstlich wichtiger Parasiten: Untersuchungen zur Struktur des männlichen Postabdomens der Raupenfliegen (Diptera, Tachinidae). Stuttgarter Beiträge zur Naturkunde. Serie A (Biologie) 383: 1–137. [29 November, OS]

[B415] Valencia LA (1972a) *Velardemyia*, nuevo genero de Tachinidae – Voriini (Diptera) del Peru. Revista Peruana de Entomología 15: 363–365.

[B416] Valencia LA (1972b) Nueva especie del genero *Winthemia* Robineau-Desvoidy, 1830 de Ica – Peru (Diptera – Tachinidae). Revista Peruana de Entomología 15: 365–367.

[B417] van der Wulp FM. See Wulp FM van der

[B418] Van Dine DL (1913) The insects affecting sugar cane in Porto Rico. Journal of Economic Entomology 6: 251–257. 10.1093/jee/6.2.251

[B419] van Emden FI. See Emden FI van

[B420] Verbeke J (1962) Contribution a l’étude des Tachinidae africains (Diptera). Exploration Hydrobiologique des Lacs Kivu, Édouard et Albert (1952–1954). Résultats scientifiques 3 (Fasc. 4): 77–187 + 25 pls. [31 December+, Evenhuis et al. 2008: 43]

[B421] Vergara de Sánchez C (1987) Terga y genitalia de tres especies de *Eucelatoria* (Dipt.: Tachinidae) en el Perú. Revista Peruana de Entomología 28 [1985]: 9–12. [20 October, OS]

[B422] Vergara de Sánchez C Raven KG (1990) Tachinidae (Diptera) registrados en el Museo de Entomología de la Universidad Nacional Agraria La Molina. Revista Peruana de Entomología 32 [1989]: 93–101. [30 August, OS]

[B423] Villers C de (1789) Caroli Linnaei entomologia, faunae Suecicae descriptionibus aucta; DD. Scopoli, Geoffroy, de Geer, Fabricii, Schrank, &c. speciebus vel in systemate non enumeratis, vel nuperrime detectis, vel speciebus Galliae australis locupletata, generum specierumque rariorum iconibus ornata. Tomus tertius. Piestre et Delamolliere, Lugduni [= Lyon], 657 pp. + pls. 7–10. [31 December+, Evenhuis and Pape 2017: 72]

[B424] Vimmer A (1940) Přehled rodů jihoamerických hystriciid (Tachinidae, Dipt.). [Übersicht der Gattungen der südamerikanischen hystriciiden (Tachinidae, Dipt.).] Časopis České Společnosti Entomologické [also as Acta Societatis Entomologicae Bohemiae] 37: 100–113.

[B425] Vimmer A Soukup J (1940a) Los dípteros de los alrededores de Puno. Tachinoidea. [Cont.] Boletín del Museo de Historia Natural “Javier Prado” 4: 205–223.

[B426] Vimmer A Soukup J (1940b) Los dípteros de los alrededores de Puno. (Conclusión.) Boletín del Museo de Historia Natural “Javier Prado” 4: 360–372.

[B427] Walker F (1836) Descriptions, &c. of the Diptera, pp. 331–359. In: Curtis J, Haliday AH, Walker F, Descriptions, &c. of the insects collected by Captain P.P. King, R.N., F.R.S., in the survey of the Straits of Magellan. Transactions of the Linnean Society of London 17 [1837]: 315–359. [21 June, Evenhuis 2018b: 77] 10.1111/j.1095-8339.1834.tb00026.x

[B428] Walker F (1849) List of the specimens of dipterous insects in the collection of the British Museum. Part IV. British Museum, London. [3] + 689–1172 pp. [8 December, Evenhuis 2018b: 82]

[B429] Walton WR (1914) A new tachinid parasite of *Diapheromerafemorata* Say. Proceedings of the Entomological Society of Washington 16: 129–132. [26 September, OS (vol. 16, p. 142)]

[B430] Webber RT (1930) A revision of the North American tachinid files of the genus *Achaetoneura*. Proceedings of the United States National Museum 78 (No. 2853) [1931]: 1–37. [10 December, OS (Table of Contents of vol. 78)] 10.5479/si.00963801.78-2853.1

[B431] Westwood JO (1840) Order XIII. Diptera Aristotle. (Antliata Fabricius. Halteriptera Clairv.), pp. 125–154. In his: Synopsis of the genera of British insects, 158 pp. In his: An introduction to the modern classification of insects; founded on the natural habits and corresponding organisation of the different families. Longman, Orme, Brown, Green & Longmans, London. [Westwood’s “An introduction ...” consists of two volumes comprising 16 parts issued 1838–1840. Westwood’s “Synopsis ...” was separately paginated and issued in parts, one part with each part of “An introduction ...”. For further details see Evenhuis (1997: 813–814).] 10.5962/bhl.title.12455

[B432] Wiedemann CRW (1830) Aussereuropäische zweiflügelige Insekten. Als Fortsetzung des Meigenschen Werkes. Zweiter Theil. Schulz, Hamm. xii + 684 pp. + 5 pls. [1 September, Evenhuis and Pont 2013: 74]

[B433] Wilkinson ATS (1984) *Tipulapaludosa* Meigen, European cranefly (Diptera: Tipulidae), pp. 85–88. In: Kelleher JS, Hulme MA (Eds), Biological control programmes against insects and weeds in Canada 1969–1980. Commonwealth Agricultural Bureaux, Slough, England, 410 pp.

[B434] Williston SW (1893) *Belvosia*—a study. Insect Life 5: 238–240 + pl. 1. [April, OS (wrapper of vol. 5, no. 4)]

[B435] Williston SW (1896) On the Diptera of St. Vincent (West Indies). Transactions of the Entomological Society of London 1896: 253–446 + pls. 8–14. [30 September, OS (wrapper of vol. for 1896)] 10.1111/j.1365-2311.1896.tb00965.x

[B436] Williston SW (1908) Manual of North American Diptera. Third edition. Illustrated. J.T. Hathaway, New Haven, 405 pp. [28 August, Evenhuis et al. 2015: 328]

[B437] Wolcott GN (1948) [The insects of Puerto Rico.] Diptera. The Journal of Agriculture of the University of Puerto Rico 32: 417–536. 10.46429/jaupr.v32i3.13612

[B438] Wood DM (1972) A revision of the New World Exoristini (Diptera: Tachinidae). I. PhorocerasubgenusPseudotachinomyia. Canadian Entomologist 104: 471–503. 10.4039/Ent104471-4

[B439] Wood DM (1985) A taxonomic conspectus of the Blondeliini of North and Central America and the West Indies (Diptera: Tachinidae). Memoirs of the Entomological Society of Canada 132: 1–130. [December, Anonymous 1995: 991] 10.4039/entm117132fv

[B440] Wood DM (1987) Tachinidae, pp. 1193–1269. In: McAlpine JF, Peterson BV, Shewell GE, Teskey HJ, Vockeroth JR, Wood DM (Eds), Manual of Nearctic Diptera. Vol. 2. Agriculture Canada Monograph 28: vi + 675–1332. [15 March, Evenhuis et al. 2010: 223]

[B441] Wood DM Zumbado MA (2010) Tachinidae (tachinid flies, parasitic flies), pp. 1343–1417. In: Brown BV, Borkent A, Cumming JM, Wood DM, Woodley NE, Zumbado MA (Eds), Manual of Central American Diptera. Vol. 2. NRC Research Press, Ottawa. xvi + 715–1442. [15 December, Evenhuis and Pont 2013: 60]

[B442] Wulp FM van der (1882) Remarks on certain American Diptera in the Leyden Museum and description of nine new species. Notes from the Leyden Museum 4: 73–92. [April, OS (wrapper of vol. 4)]

[B443] Wulp FM van der (1883) Amerikaansche Diptera. [Concl.] Tijdschrift voor Entomologie 26: 1–60 + 2 pls. [January, Barendrecht 1957: 3]

[B444] Wulp FM van der (1888) Fam. Muscidae, pp. 2–40 + pls. 1–2. [Cont.] In: Godman FD, Salvin O (Eds), Biologia Centrali-Americana, or, contributions to the knowledge of the fauna and flora of Mexico and Central America. Zoologia. Class Insecta. Order Diptera. Vol. II. [1888–1903.] Taylor & Francis, London, 489 pp. + 13 pls. [April, Evenhuis 1997: 315]

[B445] Wulp FM van der (1890a) Fam. Muscidae, pp. 41–56. [Cont.] In: Godman FD, Salvin O (Eds), Biologia Centrali-Americana, or, contributions to the knowledge of the fauna and flora of Mexico and Central America. Zoologia. Class Insecta. Order Diptera. Vol. II. [1888–1903.] Taylor & Francis, London, 489 pp. + 13 pls. [24 January, Evenhuis 1997: 315]

[B446] Wulp FM van der (1890b) Fam. Muscidae, pp. 57–88. [Cont.] In: Godman FD, Salvin O (Eds), Biologia Centrali-Americana, or, contributions to the knowledge of the fauna and flora of Mexico and Central America. Zoologia. Class Insecta. Order Diptera. Vol. II. [1888–1903.] Taylor & Francis, London, 489 pp. + 13 pls. [February, Evenhuis 1997: 315]

[B447] Wulp FM van der (1890c) Fam. Muscidae, pp. 89–112. [Cont.] In: Godman FD, Salvin O (Eds), Biologia Centrali-Americana, or, contributions to the knowledge of the fauna and flora of Mexico and Central America. Zoologia. Class Insecta. Order Diptera. Vol. II. [1888–1903.] Taylor & Francis, London, 489 pp. + 13 pls. [March, Evenhuis 1997: 315]

[B448] Wulp FM van der (1890d) Fam. Muscidae, pp. 113–144 + pl. 3. [Cont.] In: Godman FD, Salvin O (Eds), Biologia Centrali-Americana, or, contributions to the knowledge of the fauna and flora of Mexico and Central America. Zoologia. Class Insecta. Order Diptera. Vol. II. [1888–1903.] Taylor & Francis, London, 489 pp. + 13 pls. [22 May, Evenhuis 1997: 315]

[B449] Wulp FM van der (1890e) Fam. Muscidae, pp. 201–208. [Cont.] In: Godman FD, Salvin O (Eds), Biologia Centrali-Americana, or, contributions to the knowledge of the fauna and flora of Mexico and Central America. Zoologia. Class Insecta. Order Diptera. Vol. II. [1888–1903.] Taylor & Francis, London, 489 pp. + 13 pls. [November, Evenhuis 1997: 315]

[B450] Wulp FM van der (1892) Diagnoses of new Mexican Muscidae. Tijdschrift voor Entomologie 35: 183–195. [“1892.??.??”, Thompson et al. 1999: 553; Barendrecht (1957: 4) gave a date of 17 June 1893 for the entire vol. 35 but we consider a date within 1892 as more likely.]

[B451] Wulp FM van der (1903) Fam. Muscidae, pp. 433–436 + pl. 13. In: Godman FD, Salvin O (Eds), Biologia Centrali-Americana, or, contributions to the knowledge of the fauna and flora of Mexico and Central America. Zoologia. Class Insecta. Order Diptera. Vol. II. [1888–1903.] Taylor & Francis, London, 489 pp. + 13 pls. [30 April, Evenhuis 1997: 316]

[B452] Zetina DH Romero-Napoles J Contreras-Ramos A Carrillo-Sánchez JL (2018) Checklist of Tachinidae (Insecta, Diptera) in Mexico. Transactions of the American Entomological Society 144: 1–89. [25 January, OS] 10.3157/061.144.0113

